# Identification and evolution of nuclear receptors in Platyhelminths

**DOI:** 10.1371/journal.pone.0250750

**Published:** 2021-08-13

**Authors:** Wenjie Wu, Philip T. LoVerde

**Affiliations:** Departments of Biochemistry and Structural Biology and Pathology and Laboratory Medicine, University of Texas Health Sciences Center, San Antonio, Texas, United States of America; Institute of Cytology and Genetics, RUSSIAN FEDERATION

## Abstract

Since the first complete set of Platyhelminth nuclear receptors (NRs) from *Schistosoma mansoni* were identified a decade ago, more flatworm genome data is available to identify their NR complement and to analyze the evolutionary relationship of Platyhelminth NRs. NRs are important transcriptional modulators that regulate development, differentiation and reproduction of animals. In this study, NRs are identified in genome databases of thirty-three species including in all Platyhelminth classes (Rhabditophora, Monogenea, Cestoda and Trematoda). Phylogenetic analysis shows that NRs in Platyhelminths follow two different evolutionary lineages: 1) NRs in a free-living freshwater flatworm (*Schmidtea mediterranea*) and all parasitic flatworms share the same evolutionary lineage with extensive gene loss. 2) NRs in a free-living intertidal zone flatworm (*Macrostomum lignano*) follow a different evolutionary lineage with a feature of multiple gene duplication and gene divergence. The DNA binding domain (DBD) is the most conserved region in NRs which contains two C4-type zinc finger motifs. A novel zinc finger motif is identified in parasitic flatworm NRs: the second zinc finger of parasitic Platyhelminth HR96b possesses a CHC2 motif which is not found in NRs of all other animals studied to date. In this study, novel NRs (members of NR subfamily 3 and 6) are identified in flatworms, this result demonstrates that members of all six classical NR subfamilies are present in the Platyhelminth phylum. NR gene duplication, loss and divergence in Platyhelminths are analyzed along with the evolutionary relationship of Platyhelminth NRs.

## 1. Introduction

Platyhelminths (flatworms) are one of the largest animal phyla, which includes more than 20,000 species [1, 2]. Some flatworms are important disease-causing parasites of humans and livestock, e.g. Schistosomiasis, Paragonimiasis and Cestodiasis. Platyhelminths are bilaterally symmetrical, non-segmented acoelomates without an anus. Although they have an excretory system, they lack respiratory and circulatory systems. In addition, all flatworms are hermaphroditic, undergoing both asexual and sexual reproduction, with the exception of members in the Schistosomatoidae [3]. Platyhelminths are traditionally divided into four classes: Rhabditophora, Monogenea, Cestoda (tapeworms) and Trematoda (flukes). The class Rhabditophora includes all free-living flatworms, while all members in classes of Monogenea, Trematoda, and Cestoda are parasitic flatworms. From an evolutionary point of view, the parasitic classes arose from a primitive free-living flatworm [3].

Nuclear receptors (NRs) are important transcriptional modulators that regulate development, differentiation and reproduction of animals. Most of NRs share a common tertiary structure: A/B-C-D-E domains. The A/B domain is highly variable, the C domain is the DNA-binding domain (DBD) which is the most conserved region containing two zinc finger motifs, the D domain a flexible hinge between the C and E domains and is poorly conserved, the E domain contains the ligand binding domain (LBD) which is involved in transcriptional activation. Atypical NRs are found in some animals, e.g. NRs with a DBD but no LBD are found in arthropods and nematodes, members without a DBD but with a LBD are present in some vertebrates, and NRs with two DBDs and a single LBD (2DBD-NRs) are identified in protostomes. A phylogenetic analysis of the NRs divides them into six classical subfamilies by alignment of the conserved DBD [4]. The early study of the complete genome sequence of ecdysozoan Arthropods (*Drosophila melanogaster*) [5] the mosquito (*Anopheles gambiae*) [6], free-living nematodes (*Caenorhabditis elegans* and *C*. *briggsae*) [7, 8], tunicata (*Ciona intestinalis*) [9], mammalians: rat (*Rattus norvegicus*), mouse (*Mus musculus*) [10] and human (*Homo sapiens*) [11]) revealed that NRs in insects, tunicata and mammalians share the same evolutionary lineage with extensive gene loss/duplication, while NRs in nematodes follow a different evolutionary lineage with a feature of multiple duplication of SupNRs and gene loss.

Since we identified the first complete set of Platyhelminths nuclear receptors (NRs) from *Schistosoma mansoni* a decade ago [12], more flatworm genome data has become available to identify their NR complement. Analysis of the NR complement of the blood fluke *Schistosoma mansoni* [12–14] and tapeworm *Echinococcus multilocularis* [13–15] shows that NRs in *S*. *mansoni* and *E*. *multilocularis* share the same evolutionary lineage as that of Deuterostomia and the arthropods in the Ecdysozoan clade of the Protostomia, but some divergent NRs in flatworms are not present in Deuterostomia or/and arthropods, for example 2DBD-NRs [16]. In this study, we analyzed genome databases of Platyhelminth Wormbase ParaSite including species from all of the four classes in Platyhelminths [17, 18]. Identification of the NR complement will contribute to a better understanding of the evolution of NRs in Platyhelminths.

**Fig 1:**
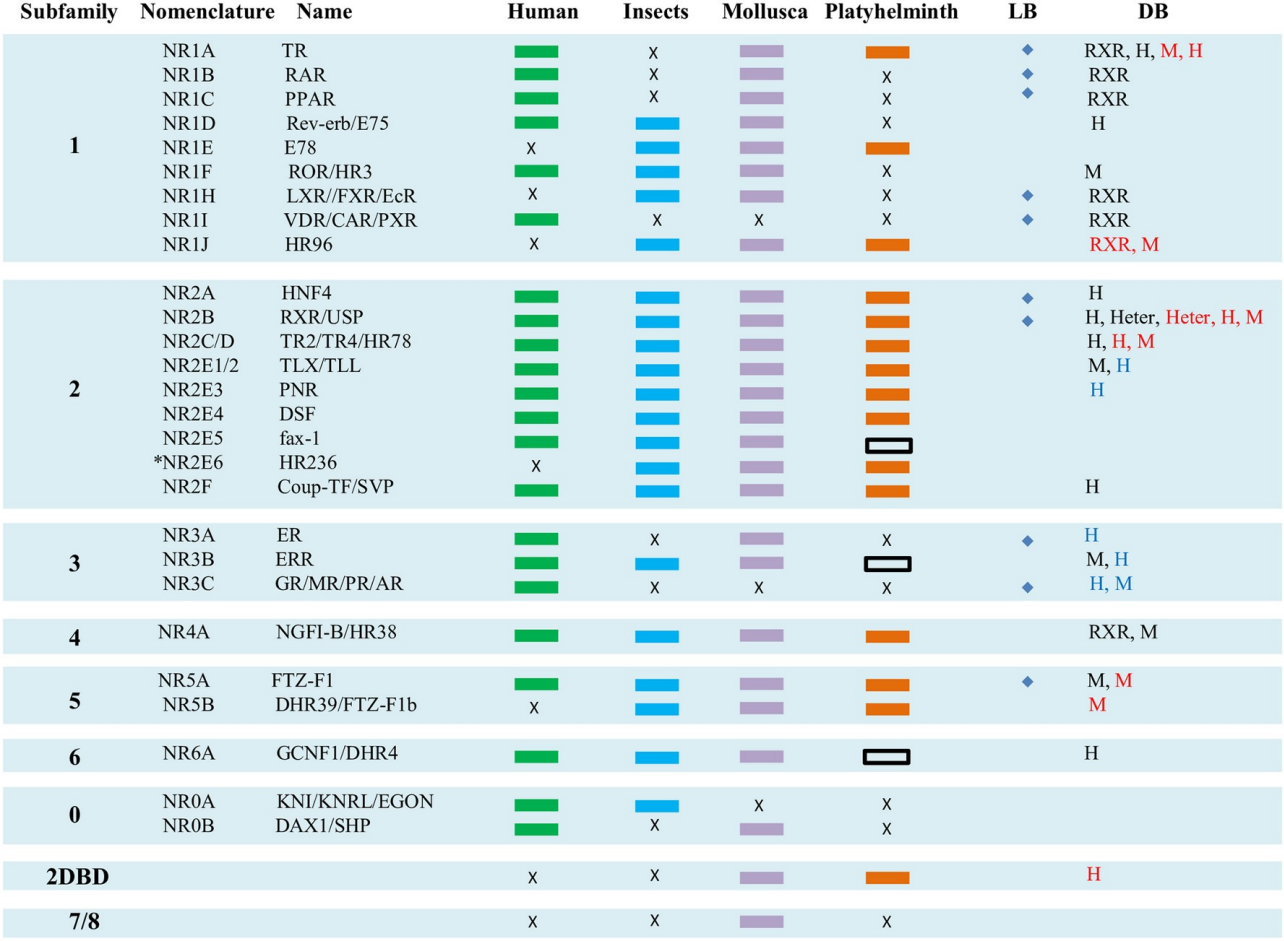
A schematic illustrates the different NR subfamilies and NRs in human, insects, Mollusca and Platyhelminths. Solid color box shows NRs in different animals, black box shows the new NRs found in Platyhelminths in this study and X indicates NRs loss in different animals. LB: Ligand binding, DB: DNA binding, diamond on ligand binding column indicates the NR was known to bind its ligand. On a DNA binding column, RXR indicates the NR can form a heterodimer with RXR, H: Homodimer, Heter: Heterodimer with other NRs, M: Monomer. On DNA binding column, the red character indicates DNA binding of Platyhelminth NRs studied *in vitro* [5, 6, 11, 16, 19–35].

A schematic that illustrates the different NR subfamilies and NRs in human, insects, Mollusca and Platyhelminths are shown in Fig 1.

## 2. Materials and methods

### 2.1. Data mining

Nuclear receptors in Platyhelminths were mined from the genome databases in Wormbase ParaSite (version: WBPS15 (WS276)) [17, 18]. Amino acid sequence of DBD of SmTRa (AY395038), SmHR96b (AY688259) and Sm2DBDα (AH013462) were used as the query to tblastn (with E-value threshold: 1e-1) against all available databases. Any sequence that contains a zinc finger structure of the NR DBD (Cys-X2-Cys-X13-Cys-X2-Cys or Cys-X5-Cys-X9-Cys-X2-Cys) was retained, and the deduced amino acid sequence of the DBD was obtained by the conserved junction position (JP) and GT-AG rule if there was an intron in the DBD coding region. The analyzed Platyhelminth genome databases are listed in Table 1. The classification of Platyhelminths and a short description of each Platyhelminth species can be found in Table 1.

**Table 1:** Platyhelminth species analyzed in Wormbase ParaSite (version: WBPS15 (WS276)).

CLASS	ORDER	FAMILY	SPECIES REFERENCES	SHOR DESCRIPTION
Rhabditophora	Rhabditophora	Rhabditophorae	*Macrostomum lignano (Ml)* [40, 41]	Free-living, intertidal flatworm that can regenerate most of its body parts.
	Tricladida	Dugesidae	*Schmidtea mediterranea (Sme)* [42–44]	Free-living, freshwater flatworm that can regenerate an entire organism.
Monogenea	Monopisthocotylea	Gyrodactylidae	*Gyrodactylus salaris (Gs)* [45]	Salmon fluke that lives on the body surface of freshwater fish.
	Polyopisthocotylea	Polystomatidae	*Protopolystoma xenopodis (Px)* [46]	Fluke of African clawed frogs where the adult worms live in the host’s urinary bladder.
Cestoda	Cyclophyllidea	Hymenolepididae	*Hymenolepis diminuta (Hd)* [46]	Rat tapeworm. The intermediate hosts: arthropod; the definitive hosts: rodent.
			*H*. *microstoma (Hm)* [47]	Rodent tapeworm. The intermediate hosts: arthropod; the definitive hosts: rodent.
			*H*. *nana (Hn)* [46]	Rodent tapeworm. The intermediate hosts: arthropod; the definitive hosts: human and rodent.
		Taeniidae	*Echinococcus Canadensis (Ec)* [48]	Canid tapeworm. The intermediate hosts: cervid, camels, cattle etc.; the definitive host: dogs and other canids.
			*E*. *granulosus (Eg)* [47, 49]	Dog tapeworm. The intermediate hosts: human, horse; the definitive host: dogs.
			*E*. *multilocularis (Em)* [47]	Fox tapeworm. The intermediate hosts: wild rodents; the definitive hosts: foxes, coyotes, dogs and other canids.
			*Taenia asiatica (Ta)* [50]	Human tapeworm. The intermediate hosts: pigs; the definitive host: human.
			*T*. *multiceps (Tm)* [51]	Dog tapeworm. The intermediate hosts: sheep, goat and cattle; the definitive hosts: Canids.
			*T*. *saginata (Ts)* [50]	Beef tapeworm. The intermediate hosts: Cattle; the definitive hosts: human.
			*T*. *solium (Tso)* [47]	Pork tapeworm. The intermediate host: pigs; the definitive host: human.
			*Hydatigera taeniaeformis (Ht)* [46]	Cat tapeworm. The intermediate hosts: rodents; the definitive hosts: cats.
		Mesocestoididae	*Mesocestoides corti (Mc)* [46]	Mouse tapeworm. The intermediate host: birds and small mammals; the definitive host: cats and dogs.
	Diphyllobotridea	Diphyllobothriidae	*Diphyllobothrium latum (Dl)* [46]	Human tapeworm. The intermediate hosts: fish; the definitive hosts: human.
			*Schistocephalus solidus (Ss)* [46]	Bird tapeworm. The first intermediate hosts: cyclopoid copepod; the second intermediate hosts: fish; the definitive hosts: fish-eating water bird.
			*Spirometra erinaceieuropaei (Se)* [52]	Tapeworm. The first intermediate hosts: cyclopoid copepod; the second intermediate hosts: fish, reptiles, or other amphibians; the definitive hosts: cats and dogs.
Trematoda	Opistorchida	Opistorchiidae	*Clonorchis sinensis (Cs)* [53]	Human liver fluke. The first intermediate hosts: freshwater snails; the second intermediate hosts: freshwater fish; the definitive hosts: human and other mammals.
			*Opisthorchis felineus (Of)* [54]	Cat liver fluke. The first intermediate hosts: freshwater snails; the second intermediate hosts: freshwater fish; the definitive hosts: human and other mammals.
			*O*. *viverrini (Ov)* [55]	Human liver fluke. The first intermediate hosts: freshwater snails; the second intermediate hosts: freshwater fish; the definitive hosts: human and other mammals.
	Echinostomida	Echinostomatidae	*Echinostoma caproni (Eca)* [46]	Intestinal fluke. The first intermediate hosts: freshwater snails; the second intermediate hosts: freshwater snails or frogs. Humans can be the definitive host.
		Fasciolidae	*Fasciola hepatica (Fh)* [56, 57]	Liver fluke. The intermediate hosts: freshwater snails; The definitive hosts: cattle, sheep and other mammals including human.
	Strigeidida	Schistosomatidae	*Schistosoma bovis (Sb)* [58]	Blood fluke. The intermediate hosts: freshwater snails; the definitive host: ruminants (cattle, goats, sheep, etc).
			*S*. *curassoni (Sc)* [46]	Blood fluke. The intermediate hosts: freshwater snails; the definitive hosts: ruminants (cattle, goat, sheep, etc).
			*S*. *haematobium (Sh)* [59]	Blood fluke. The intermediate hosts: freshwater snails; the definitive hosts: cattle, goat or sheep.
			*S*. *japonicum (Sj)* [60]	Zoonotic blood fluke. The intermediate hosts: freshwater snails; the definitive hosts: mammals including humans.
			*S*. *mansoni (Sm)* [61]	Human blood fluke. The intermediate hosts: freshwater snails; the definitive hosts: human.
			*S*. *margrebowiei (Sma)* [46]	Blood fluke. The intermediate hosts: freshwater snails; the definitive hosts: antelope, buffalo and waterbuck.
			*S*. *mattheei (Sma)* [46]	Blood fluke. The intermediate hosts: freshwater snails; the definitive hosts: bovid.
			*S*. *rodhaini (Sr)* [46]	Blood fluke. The intermediate hosts: freshwater snails; the definitive host: rodent.
			*Trichobilharzia regenti (Tr)* [46]	Neuropathogenic fluke. The intermediate host: freshwater snail; the definitive host: avian.

### 2.2. Phylogenetic analysis

Phylogenetic trees were constructed from deduced amino acid sequences of the DBD and/or ligand binding domain (LBD), the sequences are aligned with ClustalW [36], phylogenetic analysis of the data set is carried out using Bayesian inference (MrBAYES v3.1.1) [37] with a mix amino acid replacement model + gamma rates. The trees were started randomly; four simultaneous Markov chains were run for 5 million generations, the trees were sampled every 100 generations, the Bayesian posterior probabilities (BPPs) were calculated using a Markov chain Monte Carlo (MCMC) sampling approach implemented in MrBAYES v3.1.1, with a burn-in value setting at 12,500. The same data set was also tested by Maximum Likelihood method using PhyML 3.0 [38] with support values obtained by bootstrapping a 1000 replicates. The most optimal evolutionary models for phylogenetic reconstruction of NRs in each data set were determined by AIC criteria with Smart Model Selection in PhyML (SMS) [39] and the tested model for each data set was shown in each figure legend. For GenBank accession numbers of published NR sequences used in this study see S1 File.

## 3. Results

### 3.1. NR complements in Platyhelminths

NRs are identified in the genomes of 33 Platyhelminth species including 2 species (*Schistosoma mansoni* and *Echinococcus multilocularis*) that have had their NR complement reported [12, 15]. The analyzed flatworm species shown in Table 1, include 2 species from class Rhabditophora, 2 species from class Monogenea, 15 species from class Cestoda and 14 species from class Trematoda. The number of NRs varies from 15 to 61 in Platyhelminths, 18–23 NRs are present in Monogenea, 15–20 NRs are present in Cestoda, 21–22 NRs are found in Trematoda and 27–61 members are identified in Rhabditophora. For the amino acid sequences of the DBD identified in each Platyhelminth species see S2 File.

### 3.2. Phylogenetic analysis of NRs

Phylogenetic analysis of NRs using DBD sequences were carried out with Bayesian inference and Maximum Likelihood method. The DBD sequences of NRs from human, *Drosophila* and other animals were used as controls. Both methods place control NRs in mono groups and in correct families. Comparison of Bayesian inference and Maximum Likelihood method indicates Bayesian inference highly supports the analysis but Maximum Likelihood sometimes shows a lower support value (S3 File). This result indicates that Bayesian inference supports phylogenetic analysis of NRs using only DBD sequences.

The amino acid sequence of the P-box in the DBD is unique to NR members, groups or subfamilies. This sequence is helpful for identification of NRs when the phylogenetic analysis support value is low. For example, P-box sequence ESCKG is unique to NR subfamily 5 members (FTZ-F1 and DHR 39 orthologues). In this study, phylogenetic analysis of *G*. *salaris* NRs shows a lower support value for GsFTZ-F1 in subfamily 5 (S3 File), but the P-box sequence of GsFTZ-F1 (ESCKG) clearly demonstrated that it belongs to subfamily 5. The P-box sequence of all analyzed Platyhelminths NRs were checked to make sure that they share the same sequence with that of same group members. NRs in each Platyhelminth species are phylogenetic analyzed and the phylogenetic trees are reconstructed (S1–S17 Figs). NRs in Platyhelminths are summarized in Table 2.

**Table 2:** Nuclear receptors identified in Platyhelminths.

				**Platyhelminths**			
**CLASS**				**Rhabditophora**		**Monogenea**	
**ORDER**				Macrostomida	Tricladida	Polyopisthocotylea	Monopisthocotylea
**FAMILY**				Macrostomidae	Dugesidae	Polystomatidae	Gyrodactylidae
**Genus/**		*H*. *sapiens*	*D*. *melanogaster*	*Macrostomum*	*Schmidtea*	*Protopolystoma*	*Gyrodactylus*
**Species**				*lignano*	*mediterranea*	*xenopodis*	*salaris*
NR1A	TR	2		2	3	1	1
NR1B	RAR	3					
NR1C	PPAR	3					
NR1D	Rev-erb/E75	2	1				
NR1E	E78		1	2		1	1
NR1F	ROR	3	1				
NR1H	ECR/LXR/ FXR	3	1				
NR1I	VDR/PXR/CAR	3					
NR1J	DHR96		1	16	3	3	2
NR1a				1	1	1	
NR1b					1	1	
NR2A	HNF4	2	1	1	1	1	1
NR2B	RXR/USP	3	1	2	2	2	1
NR2C/D	TR2/TR4/DHR78	2	1	2	1	1	1
NR2E1/2	TLX/TLL	1	1	1	1	1	1
NR2E3	PNR	1	1	1	1	1	1
NR2E4	DSF		1	1	1	1	1
NR2E5	fax-1		1	1			
NR2E new	NHR236			2	1	1	
NR2F	COUP-TF/SVP	3	1	2	2	2	2
NR3A	ER	2					
NR3B	ERR	3	1	4			
NR3C	MR/PR/AR/GR	4					
NR4A	NGFI-B/DHR38	3	1	2	2	1	1
NR5A	FTZ-F1	2	1		1	1	
NR5B	DHR39		1	1	1	1	1
NR6A	GCNF1	1	1	1			
NR0A/B	Knirps/KNRL/ EGON/DAX1/SHP	2	3				
2DBD-NR	2DBD-NR			2	4	3	3
Other				17	1		1
**Total**		**48**	**21**	**61**	**27**	**23**	**18**
**Platyhelminths**							
**Cestoda**						**Trematoda**	
**Cyclophyllidea**			**Diphyllobothriidea**			**Opistorchida**	**Strigeidida**
						**Echinostomida**	
Hymenolepididae	Mesocestoididae	Taeniidae	Diphyllobothriidae				
*Hymenolepis*	*Mesocestoides*	*Echinococcu*	*Diphyllobothrium*	*Schistocephalus*	*Spirometra*		
	*corti*	*Hydatigera*	*latum*	*solidus*	*erinaceieuropaei*		
		*Taenia*					
1	2	1	1	1	1	2	2
1	1	1	1	1	1	1	1
3	3	3	3	3	3	3	2
	1			1	1	1	1
1	1	1	1	1	1		
1	1	1	1	1	1	1	1
			1	2	1	2	2
1	1	1	1	1	1	1	1
1	1	1	1	1	1	1	1
			1		1	1	1
						1	1
		1	1	1	1	2	2
1	1	1	1	1	1	1	1
		1	1	1	1	1	1
1	1	1	1	1	1	1	1
3	3	3	3	3	3	3	3
1	5	1	1	1	1		
**15**	**21**	**17**	**19**	**20**	**20**	**22**	**21**

### 3.3. A novel zinc finger motif (CHC2) exists in DBD of parasitic Platyhelminth NRs

DBD is the most conserved region in NRs, it contains two C4-type zinc finger motifs. In each motif, four cysteine residues chelate one Zn^2+^ ion. The first zinc finger (CI) contains a sequence element, the P-box [62, 63] which is responsible for binding the target gene, and the second zinc finger (CII) contains a sequence element the D-box which is responsible for dimerization [62].

**Fig 2:**
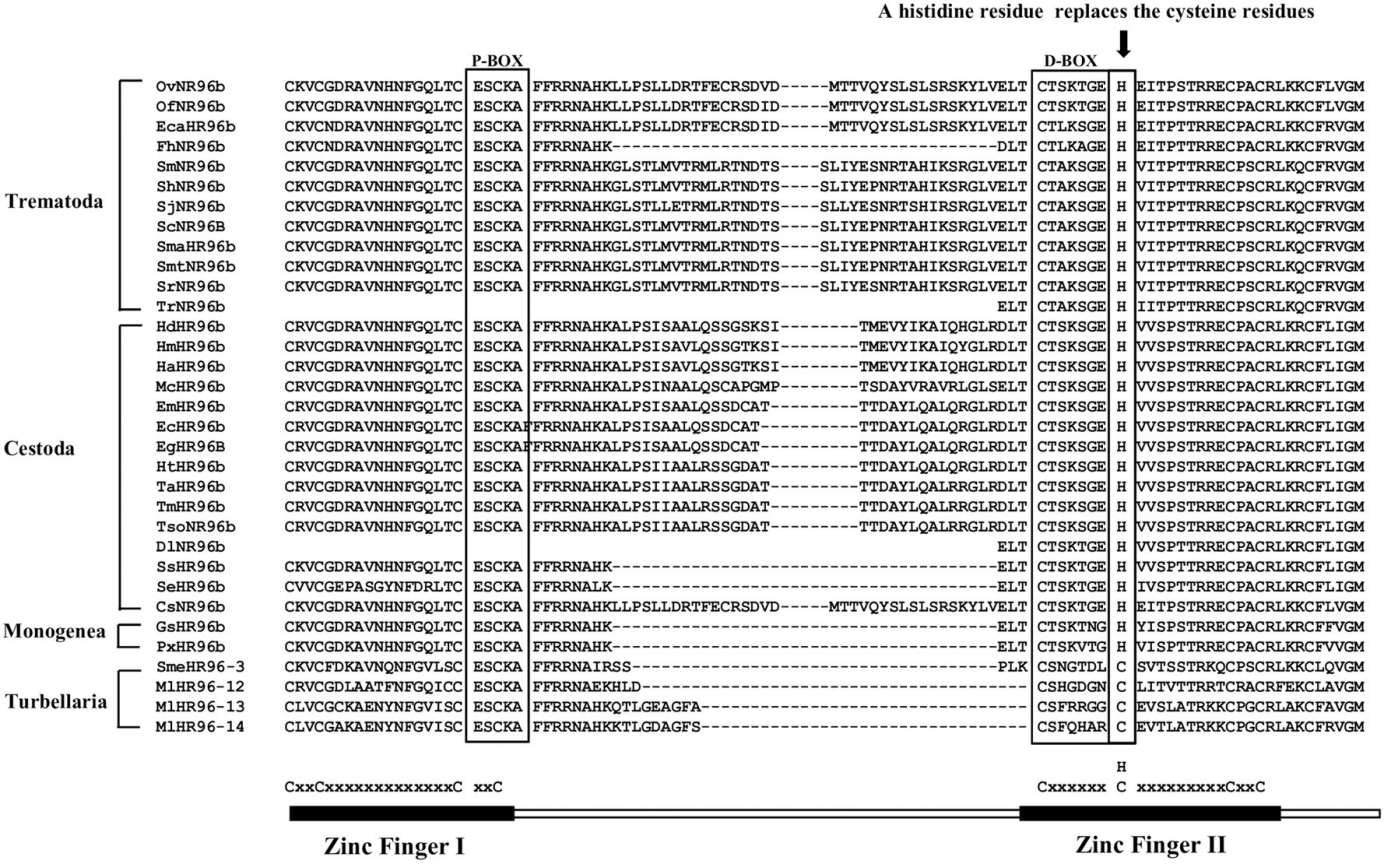
A novel zinc finger motif (CHC2) in the DBD of parasitic Platyhelminth HR96b. Amino acid sequence alignment of the DBD of the Platyhelminth HR96b shows a novel NR zinc finger motif (CHC2) is present in parasitic Platyhelminth NRs. A histidine residue replaces the second cysteine residues in the D-Box of zinc finger II forming a novel CHC2 motif. Cs: *Clonorchis sinensis*, Dl: *Dibothriocephalus latus*, Ec: *Echinococcus canadensis*, Eca: *Echinostoma caproni*, Eg: *Echinococcus granulosus*, Em: *Echinococcus multilocularis*, Fh: *Fasciola hepatica*, Gs: *Gyrodactylus salaris*, Hd: *Hymenolepis diminuta*, Hn: *Hymenolepis nana*, Ht: *Hydatigera taeniaeformis*, Lg: *Lottia gigantean*, Mc: *Mesocestoides corti*, Ml: *Macrostomum lignano*, Of: *Opisthorchis felineus*, Ov: *Opisthorchis viverrini*, Px: *Protopolystoma xenopodis*, Sb: *Schistosoma bovis*, Sc: *Schistosoma curassoni*, Se: *Spirometra erinaceieuropaei*, Sh: *Schistosoma haematobium*, Sj: *Schistosoma japonicum*, Sm: *Schistosoma mansoni*, Sma: *Schistosoma margrebowiei*, Smt: *Schistosoma mattheei*, Sme: *Schmidtea mediterranea*, Sr: *Schistosoma rodhaini*, Ss: *Schitocephalus solidus*, Ta: *Taenia asiatica*, Tm: *Taenia multiceps*, Ts: *Taenia saginata*, Tr: *Trichobilharzia regent*, Tso: *Taenia solium*.

Previously, we isolated a partial cDNA of a trematode *Schistosoma mansoni* HR96b (SmNR96b) [12], unlike other NRs, SmNR96b contains a long amino acid sequence in the DBD with two introns located in this region. DBD of SmHR96b has a histidine residue replaced by a second cysteine residue in the D-Box in CII and it forms a novel zinc finger motif (CHC2). Recently, CHC2 zinc finger motif has been demonstrated to be present in HR96b of Cestoda and other Trematoda species [64, 65]. In this study, we determined that all SmHR96b orthologues in parasitic Platyhelminths contain this novel CHC2 zinc finger motif (Fig 2). Thus, CHC2 type zinc finger motif represents a novel NR CII which has diverged in ancient HR96b in a common ancestor of parasitic Platyhelminths. The function of this novel CHC2 type motif in NRs is unknown. Recent study shows that SmHR96b (named Vitellogenic Factor 1 in [64, 65]) is essential for female sexual development.

### 3.4. New NRs identified in Platyhelminths

**Fig 3:**
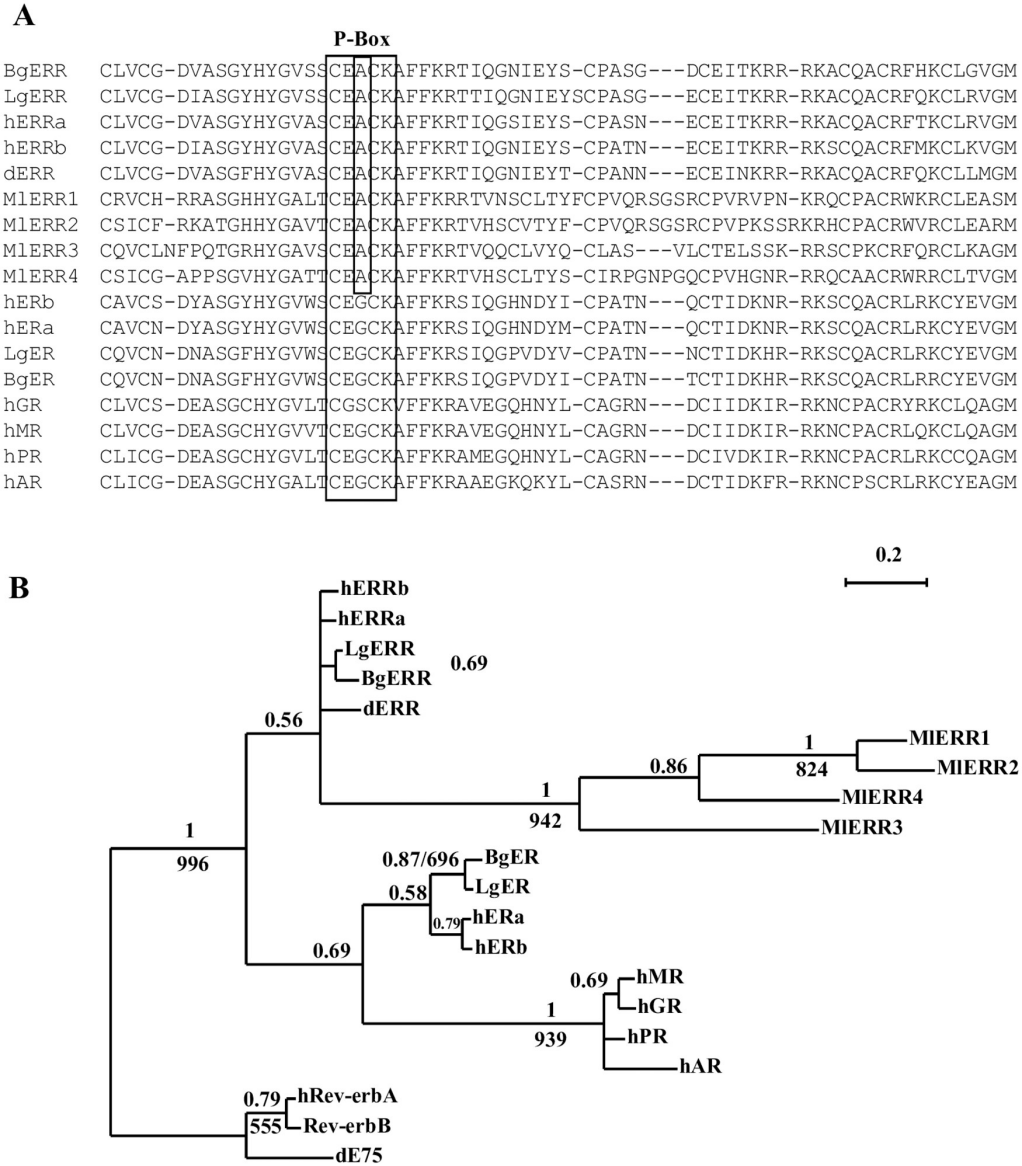
ERRs in Platyhelminths. **A)** Amino acid sequence alignment of DBD of MlERRs. **B)** Bayesian phylogenetic tree of MlERRs, the BPPs values are shown above each branch. Branches under the PPs 0.5 are shown as polytomies. The same data set was also tested by ML method using PHYML (v2.4.4) (under JTT +G model (equilibrium frequencies model, proportion of invariable sites was estimated as fixed 0.0, number of substitution rate categories: 4, gamma shape parameter was estimated as 0.547). Support values for the tree were obtained by bootstrapping a 1,000 replicates and bootstrap values above 500 are indicated below each branch (or after MrBAYES BPPs separated by Slash). Bg: *Biomphalaria glabrata*, d: *Drosophila melanogaster*, h: *Homo sapiens*, Lg: *Lottia gigantean*, Ml: *Macrostomum lignano*.

Four members of NR subfamily 3 (MlERRs), one member of subfamily 6 (MlNR6) and an orthologue of fax-1 (Mlfax-1) are identified in Rhabditophora *Macrostomum lignano* (S1 Fig), this result indicates that members of NR subfamily 3and subfamily 6 are present in Platyhelminths. The identification of fax-1 adds a new member of NR2E super-group in Platyhelminths NRs. Phylogenetic analysis of the four *M*. *lignano* NR3 (MlERRs) shows that all of them are clustered together as a separate group within NR3 subfamily with a high ML/Bayesian support value (Fig 3). Though MlERRs clustered within ERR group with a low support value (BPP = 0.56), all of the MlERRs possess a P-box (EACKA) that is unique to ERR (Fig 3), thus the MlERRs may represent a new group related human/Drosophila ERRs.

Previously, we demonstrated that a novel NR2E member (orthologue of nematode *Caenorhabditis elegans* NHR236) was present in Cnidaria, Arthropoda, free-living Platyhelminths, Mollusca and Echinodermata [14]. In this study, an orthologue of NHR236 was identified in *P*. *xenopodis* (Table 2 and S3 Fig). Amino acid sequence alignment shows that NHR236 orthologues have a unique P-box sequence (CDGCRG) and they form a new NR subgroup in NR2E super group and its progenitor gene was present in a common ancestor of Porifera and Bilateral [14]. It is the first time that an orthologue of NHR236 has been shown to exist in parasitic Platyhelminths.

Two divergent NRs from subfamily 1 are identified in different species of Platyhelminths. Phylogenetic analysis shows that Platyhelminths NR1a is an orthologue of *Schistosoma mansoni* NR1 (SmNR1) [44], which is present in different species of the four Platyhelminth classes. The other NR1 divergent member (NR1b) is an orthologue of the Cestode *Echinococcus granulosus* HR3 (EgHR3) [45], it is present in all analyzed tapeworms and also exists in the free-living Platyhelminth S. *mediterranea* (nhr10, [46]) (S18 Fig and Table 2). Phylogenetic analysis of the DBD sequences shows that Platyhelminth NR1b group clustered together with *Drosophila* HR3/human ROR/Mollusca HR3 group,but is outside of the HR3/ROR group **(**S18 Fig). To further demonstrate whether Platyhelminth NR1b is an orthologue of HR3/ROR, DBD with LBD sequences or only LBD sequence of NR1b orthologue (EgHR3 and SmeNR1b) were analyzed. Phylogenetic analysis shows that EgHR3 and SmeNR1b are clustered with the E75 group if both DBD and LBD sequences are analyzed, but they are clustered with E78 group if only LBD sequences are analyzed. These results suggest that the orthologues of Platyhelminths NR1b are a group of divergent NRs that were present in a common Platyhelminth ancestor **(**S19 and S20 Figs).

### 3.5. NR genes lost in Platyhelminths

Comparison of NRs in Platyhelminths, orthologues of *RAR*, *PPAR*, *E75*, *ROR*, *ER*, *MR*, *PR*, *GR*, *AR*, *Knir* and *NR7/8* (a new identified subfamily [26, 29]) are missing in all analyzed Platyhelminth species. *Fax-1* is only identified in Rhabditophora *M*. *lignano*, it was lost in Rhabditophora *S*. *mediterranea* and all analyzed parasitic Platyhelminths and NHR236 orthologue is not identified in Cestoda and trematode species. In Cestoda, *RXR* and *PNR* are present in Diphyllobothriidae Order but they are missing in Cyclophyllidea Order. NR gene lost in Platyhelminths is shown in Table 2.

### 3.6. Divergent NRs

Divergent NR refers to a NR which has a typical P-box sequence in the DBD but does not fall into any ‘typical’ NR groups, for example Platyhelminths NR1a and NR1b with a typical P-box of ‘CEGCKGFFRR’ belonging to NR subfamily 1 but they do not fit into any groups within the NR1 subfamily. It also refers to a NR which has an atypical P-box sequence in the DBD and does not fall into the present NR nomenclature [66].

Divergent NRs exist in various lineages of Platyhelminths. One divergent NR with a typical P-box (CEGCKGFFKR) which is the same as that of RXR/TR4/NR4A is found in Rhabditophora *S*. *mediterranea* and in Cestoda *Mesocestoides corti*. Most of the Platyhelminth divergent NRs possess an ‘atypical’ P-box sequence. For example, a NR with a P-box of ‘CEPCKVFFKR’ is identified in Monogenea *G*. *salaris*, a NR with a P-box of ‘CEACKAFFQQ’ is found in all analyzed species of Cestoda Hymenolepis family, a NR which has a P-box of ‘CDSCRAFFEM’ exists in Cestoda Taenia family, a NR with a P-box of ‘CEACKSFFKR’ is found in Cestoda Diphyllobothriidea order and seventeen NRs with various ‘atypical’ P-box sequences were identified in Rhabditophora *M*. *lignano* (S21 Fig).

A phylogenetic tree of the divergent NRs including those of Platyhelminths and Mollusca was constructed (S21 Fig). Phylogenetic analysis shows no orthologues exist among Rhabditophora *M*. *lignano*, Cestoda and Mollusca. The result suggests that these divergent NRs diverged and duplicated independently in different animal lineages.

### 3.7. NR gene duplication in Platyhelminths

#### 3.7.1. TR

One TR is identified in Monogenea; two are identified in Rhabditophora *M*. *lignano* and three are identified in *S*. *mediterranea*; and two are identified in Cestoda and Trematoda, respectively. Our previous study showed that two TR homologues are present in Platyhelminths [12–14, 34]. Phylogenetic analysis suggested that Platyhelminth TR gene duplicated after the split of the trematodes and the turbellarians [34], thus the paralogue of the trematode TR was not present in a turbellarian and in turn the paralogue of turbellarian TR was not present in trematode species. Phylogenetic analysis in this study shows that all TRs of parasitic Platyhelminths are clustered in a group, two *M*. *lignano* TRs are clustered in a group and three *S*. *mediterranea* TRs are clustered in another group. This result suggests that TRs duplicated independently in *M*. *lignano*, *S*. *mediterranea* and parasitic Platyhelminths. In parasitic Platyhelminth TR groups, trematode TRa (orthologues of *Schistosoma* TRa, SmTRa) are clustered together with those of Monogenea and Cestoda, but trematode TRb (orthologues of Schistosoma TRb, SmTRb) group only contain trematode TRs. This result suggested that one TR was present in a common ancestor of Platyhelminths and trematode TRa was an ancient TR gene. Phylogenetic analysis shows that the two Cestode *M*. *corti* TRs are clustered together in Cestoda TR group, this result suggests that TRs in Cestoda and Trematoda underwent duplication independently (S22 Fig). Since TR duplicated independently in different Platyhelminths lineage, Platyhelminths TRb/TRc are paralogous genes.

#### 3.7.2. HR96

**Fig 4:**
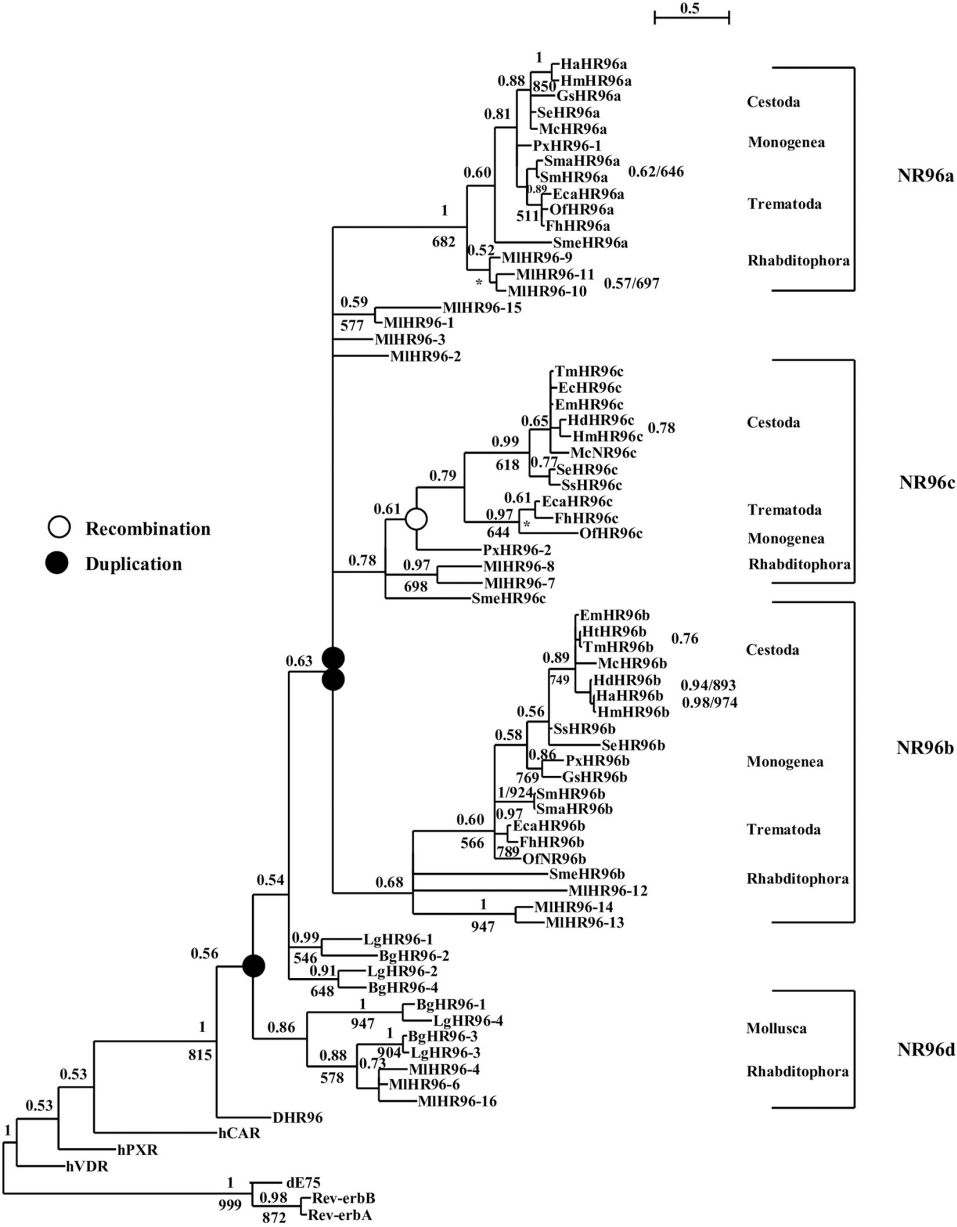
Bayesian phylogenetic tree of Platyhelminth HR96s. ML model tested as JTT+G+I (Equilibrium frequencies: Model, Proportion of invariable sites: estimated (0.149), Number of substitution rate categories: 4, Gamma shape parameter: estimated (0.727). Bg: *Biomphalaria glabrata*, Cs: *Clonorchis sinensis*, d: *Drosophila melanogaster*, Dl: *Dibothriocephalus latus*, Ec: *Echinococcus Canadensis*, Eca: *Echinostoma caproni*, Eg: *Echinococcus granulosus*, Em: *Echinococcus multilocularis*, Fh: *Fasciola hepatica*, Gs: *Gyrodactylus salaris*, h: *Homo sapiens*, Hd: *Hymenolepis diminuta*, Hn: *Hymenolepis nana*, Ht: *Hydatigera taeniaeformis*, Lg: *Lottia gigantean*, Mc: *Mesocestoides corti*, Ml: *Macrostomum lignano*, Of: *Opisthorchis felineus*, Ov: *Opisthorchis viverrini*, Px: *Protopolystoma xenopodis*, Sb: *Schistosoma bovis*, Sc: *Schistosoma curassoni*, Se: *Spirometra erinaceieuropaei*, Sh: *Schistosoma haematobium*, Sj: *Schistosoma japonicum*, Sm: *Schistosoma mansoni*, Sma: *Schistosoma margrebowiei*, Smt: *Schistosoma mattheei*, Sme: *Schmidtea mediterranea*, Sr: *Schistosoma rodhaini*, Ss: *Schitocephalus solidus*, Ta: *Taenia asiatica*, Tm: *Taenia multiceps*, Ts: *Taenia saginata*, Tr: *Trichobilharzia regent*, Tso: *Taenia solium*.

Sixteen HR96s are identified in Rhabditophora *M*. *lignano* and two are found in *S*. *mediterranea*; three HR96s are identified in Monogenea, Cestoda and Trematoda, respectively. Phylogenetic analysis shows that Platyhelminths HR96s are clustered in four different groups: HR96a, HR96b, HR96c and HR96d. HR96a, HR96b and HR96c groups contain only Platyhelminth HR96s, but HR96d group contains both Platyhelminth and Mollusca members. The result suggests that Platyhelminth HR96d is an ancient NR gene (Fig 4). HR96d group only contains Platyhelminth *M*. *lignano* HR96s, but each group of HR96a, HR96b and HR96c contains NR96s from all species of the four classes of Platyhelminths.

**Fig 5:**
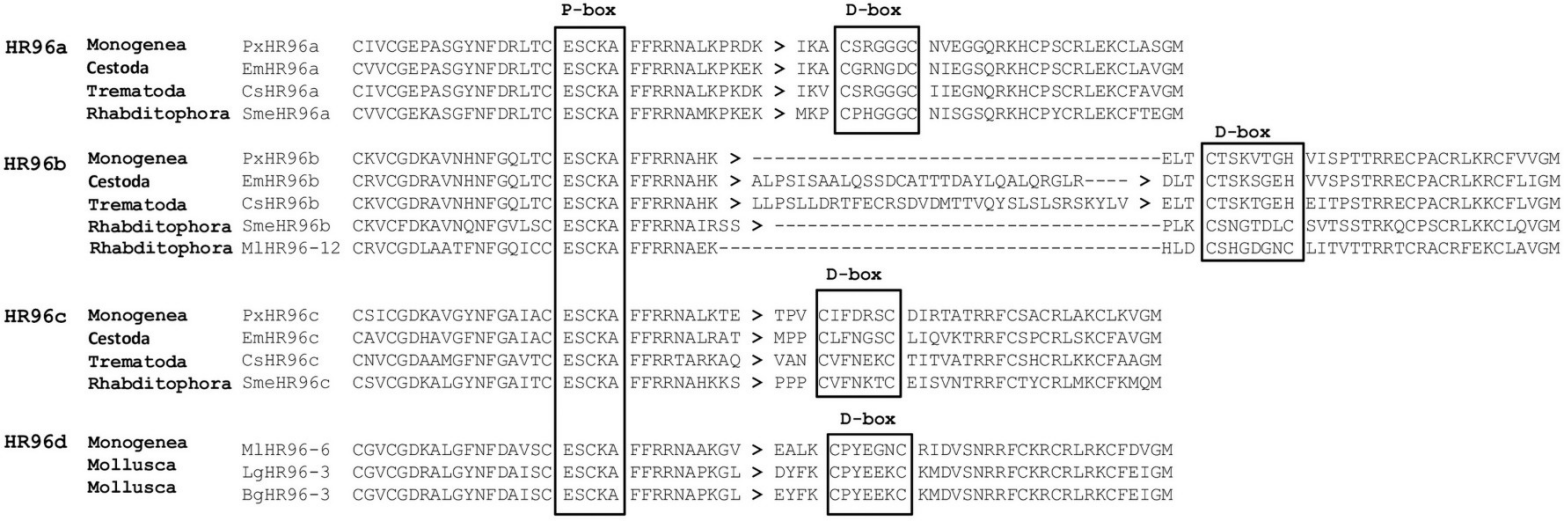
Sequence alignment and intron position of HR96s. The greater-than sign (>) indicates the intron position in DBD region.

Phylogenetic analysis shows that the BPP support values for HR96b and HR96c nodes are lower than 0.8 (Fig 4). The amino acid sequences and intron position of DBD region of HR96s were further analyzed, the result shows the amino acid length is the same within the members each of group of HR96a, HR96c and HR96d, but different in the members among groups. With the exceptionof *M*. *lignano* HR96a members have no intron located in their DBD region and the intron positions of the DBD region is the same in the members of each group but is different among groups. Amino acid length of members and intron position are unusual in Platyhelminth HR96b, most which have an extra exon inserted between the P-box and D-box region that resulted in 2 introns located in this region. Most important, DBD of all parasitic HR96b members have a histidine residue replaced by a second cysteine residue in the D-Box forming a novel zinc finger motif (CHC2). Phylogenetic analysis shows HR96d is an ancient genewhich is supported by sequence analysis, with only members of Platyhelminth HR96d sharing the same DBD amino acid length and intron position with that of Mollusca HR96s (Fig 5). For all sequence alignment of HR96s and intron position see S4 File. This result suggests that there were four HR96s in a common ancestor of Platyhelminths and the ancient HR96 gene (HR96d) was lost in *Schmidtea mediterranea*, Monogenea, Cestoda and Trematoda (Fig 4). HR96 genes were amplified in *M*. *lignano* and each of the four HR96 genes gave birth to a total of sixteen NR96 genes.

#### 3.7.3. RXR

Two RXRs were identified in Rhabditophora, Monogenea, Cestoda and Trematoda, respectively. Phylogenetic analysis shows that parasitic flatworm RXRs are clustered in two different groups: RXR1 group (*Schistosoma* RXR1 orthologues) and RXR2 group (*Schistosoma* RXR2 orthologues). RXR2 group is clustered with RXRs of free-living flatworms, *Drosophila*, Mollusca and human, which suggests that parasitic Platyhelminth RXR2 is an orthologue of free-living flatworms, *Drosophila*, Mollusca and human RXRs. RXR1 group contains only parasitic Platyhelminth RXRs, which suggests that parasitic Platyhelminth RXR2 duplicated after the split of their common ancestor with free-living Platyhelminths. In free-living flatworms, two *M*. *lignano* RXRs are clustered in a group while two *S*. *mediterranea* RXRs are scattered in the phylogenetic tree, this result suggests that RXR duplicated independently in free-living flatworms *M*. *lignano* and *S*. *mediterranea* (S23A Fig). Eventhough the support value of BPP for RXR1 and RXR2 group is 0.79 and 0.75, respectively, the intron position in the DBD of RXRs further supports the phylogenetic analysis (S23B Fig). RXR gene is lost in some of parasitic Platyhelminths after ancient RXR duplicated in a common ancestor of parasitic Platyhelminths: RXR1 was lost in Monogenea *G*. *salaris* and Cestoda *D*. *latum*; RXR2 was lost in Cestoda *S*. *erinaceieuropaei*; both RXR1 and RXR2 were lost in all the analyzed species of Cestoda Cyclophyllidea order.

#### 3.7.4. Coup-TF

One Coup-TF was identified in Cestoda; two are identified in Rhabditophora, Monogenea and Trematoda, respectively. Phylogenetic analysis shows that parasitic flatworms Coup-TFs are clustered in two different groups: Coup-TFI group (*Schistosoma* Coup-TFI orthologues) and Coup-TFII group (*Schistosoma* Coup-TFII orthologues). Coup-TFI group contains members from all three parasitic classes and they are clustered with Coup-TFs of free-living flatworms, *Drosophila*, Mollusca and humans. This result suggests that parasitic Platyhelminth Coup-TFI is an ancient Coup-TF gene and it is the orthologue of free-living flatworms, *Drosophila*, Mollusca and human Coup-TFs. Parasitic Platyhelminth Coup-TFII group contains Monogenea and Trematoda members, which suggests that Coup-TFI duplicated and gave rise to Coup-TFII in a common ancestor of parasitic Platyhelminths. In free-living flatworms, two *M*. *lignano* Coup-TFs and two *S*. *mediterranea* Coup-TFs are scattered in the phylogenetic tree, it suggests *M*. *lignano* and *S*. *mediterranea* Coup-TFs duplicated independently (S24A Fig); analysis of the intron position in DBD of Coup-TFs further supports this result (S24B Fig). Coup-TFI was missing in Cestoda Hymenolepididae family and in Mesocestoididae (*M*. *corti*); Coup-TFII was missing in all analyzed species of Cestoda.

#### 3.7.5. NR3

Four ERRs were identified in Rhabditophora *M*. *lignano*. Phylogenetic analysis shows that all four *M*. *lignano* ERRs form a single group clustered with *Drosophila*, Mollusca and human ERRs. The result demonstrates that *M*. *lignano* ERRs duplicated after a split of *Drosophila*, Mollusca, Platyhelminths and humans (Fig 3B). ERR is missing in Rhabditophora *S*. *mediterranea* and in all analyzed parasitic Platyhelminths.

#### 3.7.6. NR4

Two NR4As were identified in Rhabditophora *M*. *lignano* and *S*. *mediterranea*, respectively; one is found in all analyzed parasitic flatworms. Phylogenetic analysis shows that MlNR4Aa, SmeNR4Aa and parasitic flatworm NR4A are clustered with *Drosophila*, Mollusca and human NR4A; it suggests that MlNR4Aa, SmeNR4Aa and parasitic flatworm NR4A are orthologues of *Drosophila*, Mollusca and human NR4A. MlNR4Ab, SmeNR4Ab are scattered in the phylogenetic tree suggesting that they underwent a duplication after a split of *M*. *lignano* and *S*. *mediterranea* (S25 Fig).

#### 3.7.7. 2DBD-NR

Two 2DBD-NRs were identified in Rhabditophora, *M*. *lignano* and four were identified in *S*. *mediterranea*; three 2DBD-NRs were found in parasitic Platyhelminths including Monogenea, Cestoda and Trematoda, respectively. A phylogenetic tree of 2DBD-NRs was constructed with the amino acid sequence of the second DBD, because the second DBD is more conserved than the first DBD. Phylogenetic analysis shows that parasitic Platyhelminth 2DBD-NRs clustered in three groups: 2DBDa (*Schistosoma mansoni* 2DBDα orthologues), 2DBDb (*S*. *mansoni* 2DBDβ orthologues) and 2DBDg (*S*. *mansoni* 2DBDγ orthologues) groups. Parasitic Platyhelminth 2DBD-NRa and 2DBD-NRb groups contain members of three classes of parasitic flatworms, but 2DBD-NRg group contains 2DBD-NRs of all the four classes of Platyhelminths and members of the Mollusca. This result suggests that Parasitic Platyhelminth 2DBD-NRg is an ancient gene, and 2DBD-NRa and 2DBD-NRb were formed by a second round of duplication. In free living Platyhelminths, both Rhabditophora *M*. *lignano* 2DBD-NRs are clustered in 2DBD-NRg group, but the four *S*. *mediterranea* 2DBD-NRs are clustered with different parasitic helminth 2DBD-NR groups. This result suggests that *M*. *lignano* 2DBD-NR gene underwent duplication after a split of *S*. *mediterranea* and parasitic Platyhelminths. For the four *S*. *mediterranea* 2DBD-NRs, one is clustered in parasitic Platyhelminth 2DBD-NRg group, one is clustered in 2DBD-NRb group and two are clustered with 2DBD-NRa group. Since the two *S*. *mediterranea* 2DBD-NRs (2DBD-NRa1 and 2DBD-NRa2) in 2DBDa group form a polytomy, it suggests that 2DBD-NRa underwent another round of duplication and formed two 2DBD-NRs as a common ancestor of *S*. *mediterranea* and parasitic Platyhelminths and then one 2DBD-NRa was lost in a common ancestor of parasitic Platyhelminths (S26 Fig). The DBD sequence length is different among the members of different 2DBD-NR groups, this further supports the phylogenetic analysis. The phylogenetic analysis of 2DBD-NRs in this study is consistent with the analysis of Platyhelminths and Mollusca 2DBD-NRs [67, 68].

**Fig 6:**
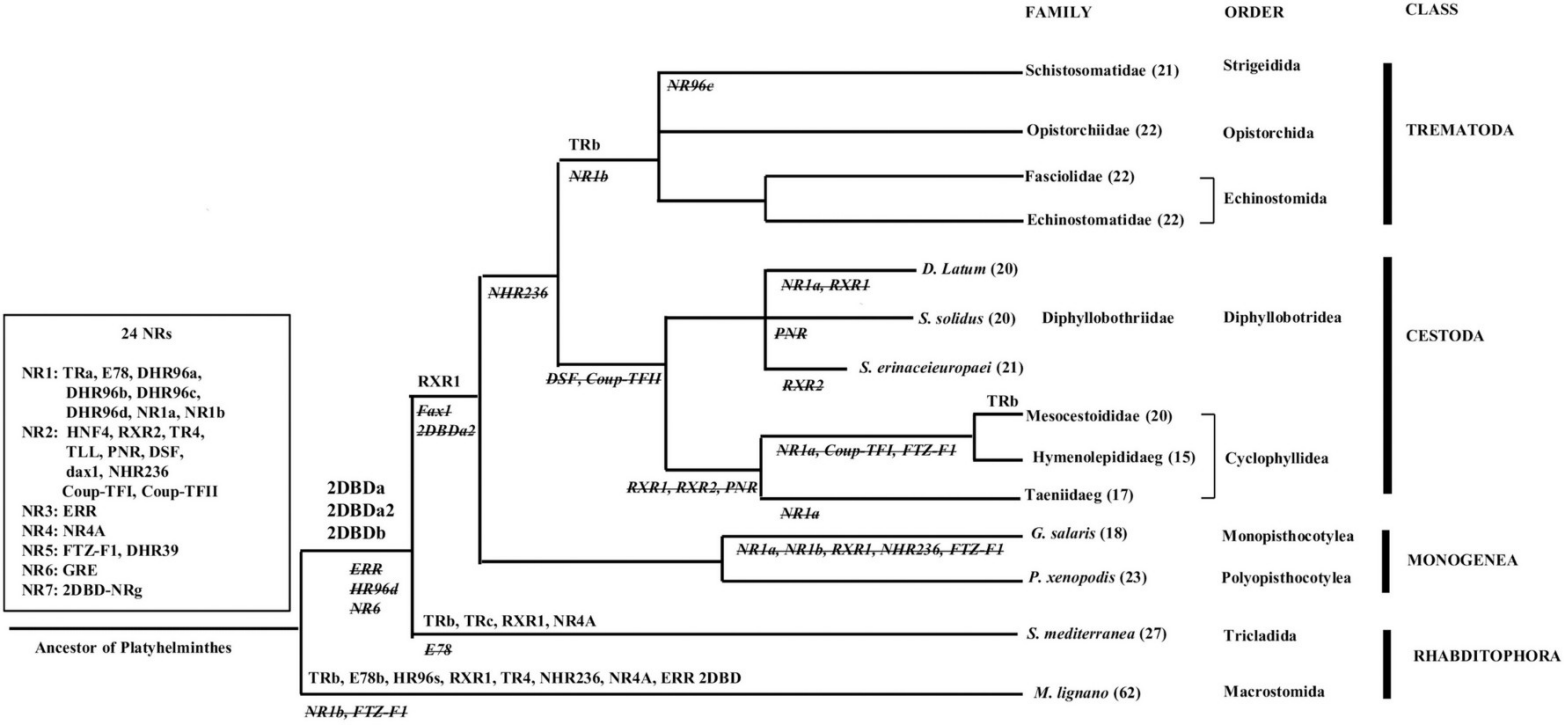
Summary of NR gene gain and loss in Platyhelminths.

The above results suggests that there were at least 24 ancient NRs in a common ancestor of Platyhelminths (Fig 4). There were 8 members from subfamily 1 (TR, E78, DHR96a, DHR96b, DHR96c, DHR96d, NR1a and NR1b); 10 members from subfamily 2 (HNF4, RXR2, TR4, TLL, PNR, DSF, dax1, NHR236, Coup-TFI and Coup-TFII); 1 member from subfamily 3 (ERR); 1 member from subfamily 4 (NR4A); 2 members from subfamily 5 (FTZ-F1 and HR39); 1 member from subfamily 6 (GRF) and 1 member from 2DBD-NRs (2DBD-NRg). Phylogenetic analysis shows that NR gene gain/loss occurred in different Platyhelminth lineages (Fig 6).

A scheme represents the phylogeny of Platyhelminths. The twenty-four ancient NR genes in a common ancestor of Platyhelminths are indicated in square box on the left of the figure. NR gene gained by gene duplication is shown above the branch of different flatworm lineages and the gene lost is shown under the branches (italic and strikethrough). The number behind flatworm species/families in parentheses indicates the number of NRs identified.

## 4. Discussion

In this study, we identified NRs in different species of Platyhelminths and performed sequence and phylogenetic analysis of them. Phylogenetic analysis of the DBD of NRs using DBD sequences was carried out with Bayesian inference and Maximum Likelihood method, Comparison of the two methods, Bayesian inference highly support phylogenetic analysis of NRs using only DBD sequences. (S3 File). This study shows that NRs in Platyhelminths have orthologues in Deuterostomes, arthropods or both, and the NRs in Platyhelminths diverged into two different evolutionary lineages: 1) Gene duplication and lost; 2) NR gene amplification and divergence.

NRs in Rhabditophora *S*. *mediterranea* and parasitic Platyhelminths follow the first evolutionary lineage: gene duplication and lost, the events occurred in different flatworm lineages. For example, fax-1 was lost in a common ancestor of *S*. *mediterranea* and parasitic Platyhelminths, NHR236 was lost in Cestoda and Trematoda, and DSF was lost in Cestoda. In parasitic Platyhelminths, extensive NR gene loss occurred in Cestoda Hymenolepididae and Taeniidae families, e. g. RXR, Coup-TF and FTZ-F1 were retained in most species of Platyhelminths, but they were missing in the cestode families Hymenolepididae and Taeniidae.

Comparison of NR complement in different Platyhelminth families shows that NR complement is most conserved in Trematoda. All species of Trematoda share the same NR complement except that a HR96 member was lost in Strigeidida (*Schistosoma* and *Trichobilharzia*). NR complement in Monogenea and in Cestoda exhibit more differences among different families, in Monogenea, there are 18 NRs in Gyrodactylidae (*G*. *salaris*), but 23 NRs are present in Polyopisthocotylea (*P*. *xenopodis*). In Cestoda Cyclophyllidea, there are 15 NRs in Hymenolepididae family, 17 in Taeniidae family and 20 in Mesocestoididae family. NR gene duplication occurred in different flatworm lineages. For example, 2DBD-NR underwent a second round of duplication in a common ancestor of *S*. *mediterranea* and parasitic Platyhelminths, RXR gene duplicated separately in *S*. *mediterranea* and a common ancestor of parasitic Platyhelminths, and TR duplicated independently in Rhabditophora (*S*. *mediterranea*), Cestoda and Trematoda.

NRs in Rhabditophora *M*. *lignano* follow another evolutionary lineage: Gene amplification and divergence. In *M*. *lignano*, 61 NRs are identified including 16 HR96s and 17 divergent members. This result suggests that NRs in *M*. *lignano* follows an evolutionary lineage with a feature of multiple gene duplication (amplification) and gene divergence. Multiple NR gene duplication was also found in other animals, for example, supplementary NRs (SupNRs) in nematodes and NR1H in Chordate *Cephalochordate amphioxus* [29, 69]. DHR96 (NR1J) plays a role in the response to xenobiotics. The regulation of lipid metabolism and xenobiotics is an ancestral function of members in NR1J group, HR96 links environmental conditions to physiology and development [70]. MlHR96 gene amplification in *M*. *lignano* may result from the adaption to its environment.

NRs in subfamily 3 and 6 were considered lost in Platyhelminths. In this study, we identified four ERRs and a NR6A in Rhabditophora *M*. *lignano*. This is the first known occurrence of NRs in subfamily 3 and 6 in Platyhelminths, but it is still not clear whether NRs from subfamily 3 and 6 are present in other Rhabditophora since the genome data is unavailable. This study also shows that divergent NRs are present in different flatworm lineages suggesting that novel NRs were acquired in different flatworms to adapt to the different living environments.

E75/Rev, DHR3/ROR and EcR/LXR, which are present in Deuterostomia, Arthropods and Lophotrochozoa Mollusca, are missing in Platyhelminths. In insects, E75, HR3 and EcR are directly involved in the control of the ecdysone pathway. E75 acts as a repressor of DHR3 and may through direct interaction [70].

RAR, PPAR, ER, which are missing in Arthropods but retained in Lophotrochozoa Mollusca, are also missing in Platyhelminths. In vertebrates, RARs are known to bind retinoic acid (RA). RA is a morphogen derived from vitamin A, it controls the patterning of the anteroposterior axis and the differentiation of various cell types [71]. Study of Mollusca *Nucella lapillus* RAR (NlRAR) showed that NlRAR binds to NR response elements as a heterodimer with RXR, but it does not bind all-trans retinoic acid or other retinoids [71]. In vertebrates, PPARs form a heterodimer with RXR binding their response elements, and their ligand includes free fatty acids, eicosanoids and Vitamin B3. In Mollusca *N*. *lapillus*, PPAR-responsive pathways is related to tributyltin (TBT) induced imposex [72]. Vertebrate ERs are activated by estradiol (E2) and have important roles in development of the nervous system and secondary sexual traits, but Mollusca ERs cannot bind estrogen, they are constitutively active and retained the ability to regulate their own gene transcription [73].

ERRs are orphan receptors in Chordata and Ecdysozoa, they play important roles in regulation of neurogenesis and metabolism in Chordata and are involved in control of larval growth in Ecdysozoa. The function of Lophotrochozoa ERRs is unknown [73]. In this study, four ERRs are identified in Platyhelminths (MlERRs), phylogenetic analysis shows that these members are closely related ERRs. The further characterization will reveal their role in development of Platyhelminths.

The NR2E6 gene was first identified in the genome of the honeybee *Apis mellifera* and then in other insects, but it was missing in *Drosophila*. The missing of NR2E6 in the major model organisms delayed the identification and functional analysis of this protein. Sometimes NR2E6 is named as PNR-like or PNR, but the true insect homolog of vertebrate PNR is HR51 (NR2E3) [70]. We demonstrated that a novel NR2E member (NHR236, orthologue of nematode *Caenorhabditis elegans* HR236) was present in Cnidaria, Arthropoda, Platyhelminths, Mollusca and Echinodermata [14]. Insect NR2E6 is an orthologue of NHR236 and all of NHR236 orthologues possess an unique P-box sequence (CDGCRG) in the DBD region.

Previously, we isolated a partial cDNA of *S*. *mansoni* HR96b (SmNR96b) [12], this member has a CHC2 zinc finger motif in the second zinc finger of DBD. In this study, we show that all parasitic Platyhelminth SmHR96b orthologues contain this novel motif. Whether the function of this new type of motif in NRs may change the DNA binding properties awaits further study.

## 5. Addendum

A paper on *Macrostomum lignano* NRs was published (Cheng, Y., et al., *Genome-Wide Characterization of the Nuclear Receptor Gene Family in Macrostomum lignano Imply Its Evolutionary Diversification*. Frontiers in Marine Science, 2021. **8**(645)) while this paper was under review. The main differences of identification of *M*. *lignano* NRs between Cheng et al. and this contribution are as follows (for details see S5 File): (1). Cheng, Y., et al. identified 51 NRs in *M*. *lignano*, we identified 61 NRs in this species. (2). Cheng, Y., et al. identified 4 NR1Cs (PPARs) in *M*. *lignano*, we found no evidence for any PPARs in *M*. *lignano*. (3). Cheng, Y., et al. identified 2 NR1Fs (RORs). We identified the same sequences but our phylogenetical analysis showed that they belonged to NR1E (E78) group. (4). Cheng, Y., et al. identified 20 NR1Js (HR96s) in *M*. *lignano* including one sequence without a DBD sequence and 5 members each with an atypical P-box sequence which is different from the typical HR96 P-box sequence. We identified 16 HR96s in *M*. *lignano*, all of them possessed a typical HR96 P-box sequence. We also identified the same 5 members with an atypical P-box sequence that Cheng, Y., et al. identified, but put them into a divergent NR group. (5). Cheng, Y., et al. identified 10 NR3 members in *M*. *lignano* including four members that each possessed a typical ERR P-box sequences and 6 members that each possessed an atypical P-box sequence. We identified the same members but put the six members that possessed an atypical P-box sequence into a divergent NR group. (6). Cheng, Y., et al. identified a NR8 member, we identified the same member but our phylogenetical analysis showed it was a divergent member. (7). Cheng, Y., et al. identified 2 NR0 members. We identified the same members but our phylogenetic analysis showed one of them belong to subfamily 6 and the other one is a divergent member because it possessed an atypical P-box sequence.

## Supporting information

S1 Fig Bayesian phylogenetic tree of *Macrostomum lignano* NRs. The Bayesian tree was constructed with the deduced amino sequences of the DNA binding domain (DBD) with a mix amino acid replacement model + invgamma rates. The PPs values are shown above each branch, branches under the PPs 0.5 are shown as polytomies. The same data set is also tested by ML method using PHYML (v2.4.4) under LG+G+I substitution model (Equilibrium frequencies: Model, Proportion of invariable sites: Estimated (0.158), Number of substitution rate categories: 4, Gamma shape parameter: Estimated (0.956). Support values for the tree are obtained by bootstrapping a 1,000 replicates and bootstrap values above 500 and are indicated below each branch (or after MrBAYES BPPs separated by Slash). Star indicates the node obtained by Bayesian inference which is different from that obtained by ML method. Bg: *Biomphalaria glabrata*, Cg: *Crassostrea gigas*, d: *Drosophila melanogaster*, h: *Homo sapiens*, Lg: *Lottia gigantean*, Ml: *Macrostomum lignano*, Px: *Protopolystoma xenopodis*, Sm: *Schistosoma mansoni*. Red highlighted NRs show *M*. *lignano* NRs.
(TIF)

S2 Fig Bayesian phylogenetic tree of *Schmidtea mediterranea* NRs. Methods for construction of phylogenetic trees see S1 Fig legend. ML model tested as LG+G+I (Equilibrium frequencies: Model, Proportion of invariable sites: Estimated (0.165), Number of substitution rate categories: 4, Gamma shape parameter: Estimated (0.751). Bg: *Biomphalaria glabrata*, Cg: *Crassostrea gigas*, d: *Drosophila melanogaster*, h: *Homo sapiens*, Lg: *Lottia gigantean*, Px: *Protopolystoma xenopodis*, Sm: *Schistosoma mansoni*, Sme: *Schmidtea mediterranea*. Red highlighted NRs show *S*. *mediterranea* NRs.
(TIF)

S3 Fig Bayesian phylogenetic tree of *Protopolystoma xenopodis* NRs. Methods for construction of phylogenetic trees see S1 Fig legend. ML model tested as LG+G+I (Equilibrium frequencies: Model, Proportion of invariable sites: Estimated (0.119), Number of substitution rate categories: 4, Gamma shape parameter: Estimated (0.691). Bg: *Biomphalaria glabrata*, Cg: *Crassostrea gigas*, d: *Drosophila melanogaster*, h: *Homo sapiens*, Lg: *Lottia gigantean*, Px: *Protopolystoma xenopodis*, Sm: *Schistosoma mansoni*. Red highlighted NRs show *P*. *xenopodis* NRs.
(TIF)

S4 Fig Bayesian phylogenetic tree *Gyrodactylus salaris* NRs. Methods for construction of phylogenetic trees see S1 Fig legend. ML model tested as LG+G+I (Equilibrium frequencies: Model, Proportion of invariable sites: Estimated (0.119), Number of substitution rate categories: 4, Gamma shape parameter: Estimated (0.691). Bg: *Biomphalaria glabrata*, Cg: *Crassostrea gigas*, d: *Drosophila melanogaster*, Gs: *Gyrodactylus salaris*, h: *Homo sapiens*, Lg: *Lottia gigantean*, Px: *Protopolystoma xenopodis*, Sm: *Schistosoma mansoni*. Red highlighted NRs show *G*. *salaris* NRs.
(TIF)

S5 Fig Bayesian phylogenetic tree of *Hymenolepis microstoma* NRs. The phylogenetic tree of *H*. *microstoma* NRs represents all analyzed *Hymenolepis* species because of the highly conserved DBD sequences in these species. Methods for construction of phylogenetic trees see S1 Fig legend. ML model tested as LG+G+I (Equilibrium frequencies: Model, Proportion of invariable sites: Estimated (0.092), Number of substitution rate categories: 4, Gamma shape parameter: Estimated (0.709). Bg: *Biomphalaria glabrata*, Cg: *Crassostrea gigas*, d: *Drosophila melanogaster*, h: *Homo sapiens*, Hm: *Hymenolepis microstoma*, Lg: *Lottia gigantean*, Px: *Protopolystoma xenopodis*, Sm: *Schistosoma mansoni*. Red highlighted NRs show *H*. *microstoma* NRs.
(TIF)

S6 Fig Bayesian phylogenetic tree of *Mesocestoides corti* NRs. Methods for construction of phylogenetic trees see S1 Fig legend. ML model tested as LG+G+I (Equilibrium frequencies: Model, Proportion of invariable sites: Estimated (0.073), Number of substitution rate categories: 4, Gamma shape parameter: Estimated (0.782). Bg: *Biomphalaria glabrata*, Cg: *Crassostrea gigas*, d: *Drosophila melanogaster*, h: *Homo sapiens*, Lg: *Lottia gigantean*, Mc: *Mesocestoides corti*, Px: *Protopolystoma xenopodis*, Sm: *Schistosoma mansoni*. Red highlighted NRs show *M*. *corti* NRs.
(TIF)

S7 Fig Bayesian phylogenetic tree of *Echinococcus multilocularis* NRs. Methods for construction of phylogenetic trees see S1 Fig legend. ML model tested as LG+G+I (Equilibrium frequencies: Model, Proportion of invariable sites: Estimated (0.103), Number of substitution rate categories: 4, Gamma shape parameter: Estimated (0.735). Bg: *Biomphalaria glabrata*, Cg: *Crassostrea gigas*, d: *Drosophila melanogaster*, h: *Homo sapiens*, Lg: *Lottia gigantean*, *Em*: *Echinococcus multilocularis*, Px: *Protopolystoma xenopodis*, Sm: *Schistosoma mansoni*. Red highlighted NRs show *E*. *multilocularis* NRs.
(TIF)

S8 Fig Bayesian phylogenetic tree of *Hydatigera taeniaeformis* NRs. Methods for construction of phylogenetic trees see S1 Fig legend. ML model tested as LG+G+I (Equilibrium frequencies: Model, Proportion of invariable sites: Estimated (0.093), Number of substitution rate categories: 4, Gamma shape parameter: Estimated (0.717). Bg: *Biomphalaria glabrata*, Cg: *Crassostrea gigas*, d: *Drosophila melanogaster*, h: *Homo sapiens*, Ht: *Hydatigera taeniaeformis*, Lg: *Lottia gigantean*, Px: *Protopolystoma xenopodis*, Sm: *Schistosoma mansoni*. Red highlighted NRs show *H*. *taeniaeformis* NRs.
(TIF)

S9 Fig Bayesian phylogenetic tree of *Taenia saginata* NRs. Methods for construction of phylogenetic trees see S1 Fig legend. ML model tested as LG+G+I (Equilibrium frequencies: Model, Proportion of invariable sites: Estimated (0.123), Number of substitution rate categories: 4, Gamma shape parameter: Estimated (0.898). Bg: *Biomphalaria glabrata*, Cg: *Crassostrea gigas*, d: *Drosophila melanogaster*, h: *Homo sapiens*, Lg: *Lottia gigantean*, Px: *Protopolystoma xenopodis*, Sm: *Schistosoma mansoni*, Ts: *Taenia saginata*. Red highlighted NRs show *T*. *saginata* NRs.
(TIF)

S10 Fig Bayesian phylogenetic tree of *Schitocephalus solidus* NRs. Methods for construction of phylogenetic trees see S1 Fig legend. ML model tested as LG+G+I (Equilibrium frequencies: Model, Proportion of invariable sites: Estimated (0.129), Number of substitution rate categories: 4, Gamma shape parameter: Estimated (0.744). Bg: *Biomphalaria glabrata*, Cg: *Crassostrea gigas*, d: *Drosophila melanogaster*, h: *Homo sapiens*, Ss: *Schitocephalus solidus*, Lg: *Lottia gigantean*, Px: *Protopolystoma xenopodis*, Sm: *Schistosoma mansoni*. Red highlighted NRs show *T*. *saginata* NRs.
(TIF)

S11 Fig Bayesian phylogenetic tree of *Spirometra erinaceieuropaei* NRs. Methods for construction of phylogenetic trees see S1 Fig legend. ML model tested as LG+G+I (Equilibrium frequencies: Model, Proportion of invariable sites: Estimated (0.129), Number of substitution rate categories: 4, Gamma shape parameter: Estimated (0.701). Bg: *Biomphalaria glabrata*, Cg: *Crassostrea gigas*, d: *Drosophila melanogaster*, h: *Homo sapiens*, Lg: *Lottia gigantean*, Px: *Protopolystoma xenopodis*, Se: *Spirometra erinaceieuropaei*, Sm: *Schistosoma mansoni*. DBD of E78 sequence is partial, not used for construction of the tree. Red highlighted NRs show *S*. *erinaceieuropaei* NRs.
(TIF)

S12 Fig Bayesian phylogenetic tree of *Clonorchis sinensis* NRs. Methods for construction of phylogenetic trees see S1 Fig legend. ML model tested as LG+G+I (Equilibrium frequencies: Model, Proportion of invariable sites: Estimated (0.127), Number of substitution rate categories: 4, Gamma shape parameter: Estimated (0.747). Bg: *Biomphalaria glabrata*, Cg: *Crassostrea gigas*, Cs: *Clonorchis sinensis*, d: *Drosophila melanogaster*, h: *Homo sapiens*, Lg: *Lottia gigantean*, Px: *Protopolystoma xenopodis*, Sm: *Schistosoma mansoni*. Red highlighted NRs show *C*. *sinensis* NRs.
(TIF)

S13 Fig Bayesian phylogenetic tree of *Opisthorchis viverrini* NRs. The phylogenetic tree of *O viverrini* NRs represents *Opisthorchis viverrini* and *O*. *felineus* because of the highly conserved DBD sequences in these two species. Methods for construction of phylogenetic trees see S1 Fig legend. ML model tested as LG+G+I (Equilibrium frequencies: Model, Proportion of invariable sites: Estimated (0.123), Number of substitution rate categories: 4, Gamma shape parameter: Estimated (0.830). Bg: *Biomphalaria glabrata*, Cg: *Crassostrea gigas*, d: *Drosophila melanogaster*, h: *Homo sapiens*, Lg: *Lottia gigantean*, Ov: *Opisthorchis viverrini*, Px: *Protopolystoma xenopodis*, Sm: *Schistosoma mansoni*. Red highlighted NRs show *O*. *viverrini* NRs.
(TIF)

S14 Fig Bayesian phylogenetic tree of *Fasciola hepatica* NRs. Methods for construction of phylogenetic trees see S1 Fig legend. ML model tested as LG+G+I (Equilibrium frequencies: Model, Proportion of invariable sites: Estimated (0.133), Number of substitution rate categories: 4, Gamma shape parameter: Estimated (0.834). Bg: *Biomphalaria glabrata*, Cg: *Crassostrea gigas*, d: *Drosophila melanogaster*, Fh: *Fasciola hepatica*, h: *Homo sapiens*, Lg: *Lottia gigantean*, Px: *Protopolystoma xenopodis*, Sm: *Schistosoma mansoni*. Red highlighted NRs show *F*. *hepatica* NRs.
(TIF)

S15 Fig Bayesian phylogenetic tree of *Echinostoma caproni* NRs. Methods for construction of phylogenetic trees see S1 Fig legend. ML model tested as LG+G+I (Equilibrium frequencies: Model, Proportion of invariable sites: Estimated (0.127), Number of substitution rate categories: 4, Gamma shape parameter: Estimated (0.802). Bg: *Biomphalaria glabrata*, Cg: *Crassostrea gigas*, d: *Drosophila melanogaster*, Eca: *Echinostoma caproni*, h: *Homo sapiens*, Lg: *Lottia gigantean*, Px: *Protopolystoma xenopodis*, Sm: *Schistosoma mansoni*. EcaCoup-TFII sequence is partial and not used for construction of the tree. Red highlighted NRs show *E*. *caproni* NRs.
(TIF)

S16 Fig Bayesian phylogenetic tree of *Schistosoma haematobium* NRs. The phylogenetic tree of *S haematobium* NRs represents all analyzed *Schistosoma* species because of the highly conserved DBD sequences in these species. Methods for construction of phylogenetic trees see S1 Fig legend. ML model tested as LG+G+I (Equilibrium frequencies: Model, Proportion of invariable sites: Estimated (0.119), Number of substitution rate categories: 4, Gamma shape parameter: Estimated (0.714). Bg: *Biomphalaria glabrata*, Cg: *Crassostrea gigas*, d: *Drosophila melanogaster*, h: *Homo sapiens*, Lg: *Lottia gigantean*, Px: *Protopolystoma xenopodis*, Sh: *Schistosoma haematobium*, Sm: *Schistosoma mansoni*. Red highlighted NRs show *S*. *haematobium* NRs.
(TIF)

S17 Fig Bayesian phylogenetic tree of *Trichobilharzia regent* NRs. Methods for construction of phylogenetic trees see S1 Fig legend. ML model tested as LG+G+I (Equilibrium frequencies: Model, Proportion of invariable sites: Estimated (0.093), Number of substitution rate categories: 4, Gamma shape parameter: Estimated (0.709). Bg: *Biomphalaria glabrata*, Cg: *Crassostrea gigas*, d: *Drosophila melanogaster*, h: *Homo sapiens*, Lg: *Lottia gigantean*, Px: *Protopolystoma xenopodis*, Sm: *Schistosoma mansoni*, Tr: *Trichobilharzia regent*. Red highlighted NRs show *T*. *regent* NRs.
(TIF)

S18 Fig Bayesian phylogenetic tree of NR1 DBD sequences. Methods for construction of phylogenetic trees see S1 Fig legend. ML model tested as JTT+G (Equilibrium frequencies: Model, Proportion of invariable sites: Fixed (0.0), Number of substitution rate categories: 4, Gamma shape parameter: Estimated (0.378). Bg: *Biomphalaria glabrata*, d: *Drosophila melanogaster*, Dl: *Dibothriocephalus latus*, Eca: *Echinostoma caproni*, Eg: *Echinococcus granulosus*, Fh: *Fasciola hepatica*, h: *Homo sapiens*, Hd: *Hymenolepis diminuta*, Ht: *Hydatigera taeniaeformis*, Lg: *Lottia gigantean*, Mc: *Mesocestoides corti*, Ml: *Macrostomum lignano*, Ov: *Opisthorchis viverrini*, Px: *Protopolystoma xenopodis*, Se: *Spirometra erinaceieuropaei*, Sm: *Schistosoma mansoni*, Sme: *Schmidtea mediterranea*, Ss: *Schitocephalus solidus*, Tm: *Taenia multiceps*, Tr: *Trichobilharzia regent*. Red highlighted NRs show Platyhelminths NRs.
(TIF)

S19 Fig Bayesian phylogenetic tree of NR1 DBD with LBD. Methods for construction of phylogenetic trees see S1 Fig legend. ML model tested as LG+G+I+F (Equilibrium frequencies: Empirical, Proportion of invariable sites: Estimated (0.029), Number of substitution rate categories: 4, Gamma shape parameter: Estimated (1.348). Bg: *Biomphalaria glabrata*, d: *Drosophila melanogaster*, Eg: *Echinococcus granulosus*, h: *Homo sapiens*, Lg: *Lottia gigantean*, Sme: *Schmidtea mediterranea*. Red highlighted NRs show Platyhelminths NRs.
(TIF)

S20 Fig Bayesian phylogenetic tree of NR1 LBD. Methods for construction of phylogenetic trees see S1 Fig legend. ML model tested as LG+G+I+F (Equilibrium frequencies: Empirical, Proportion of invariable sites: Estimated (0.004), Number of substitution rate categories: 4, Gamma shape parameter: Estimated (1.741). Bg: *Biomphalaria glabrata*, d: *Drosophila melanogaster*, Eg: *Echinococcus granulosus*, h: *Homo sapiens*, Lg: *Lottia gigantean*, Sme: *Schmidtea mediterranea*. Red highlighted NRs show Platyhelminths NRs.
(TIF)

S21 Fig Bayesian phylogenetic tree of Platyhelminth divergent NRs. Methods for construction of phylogenetic trees see S1 Fig legend. ML model tested as JTT+G (Equilibrium frequencies: Model, Proportion of invariable sites: Fixed (0.0), Number of substitution rate categories: 4, Gamma shape parameter: Estimated (0.895). Bg: *Biomphalaria glabrata*, d: *Drosophila melanogaster*, Dl: *Dibothriocephalus latus*, Ec: *Echinococcus Canadensis*, Eg: *Echinococcus granulosus*, Em: *Echinococcus multilocularis*, Gs: *Gyrodactylus salaris*, h: *Homo sapiens*, Hd: *Hymenolepis diminuta*, Hm: Of *H*. *microstoma*, Ht: *Hydatigera taeniaeformis*, Mc: *Mesocestoides corti*, Ml: *Macrostomum lignano*, Sme: *Schmidtea mediterranea*, Ta: *Taenia asiatica*, Tm: *Taenia multiceps*, Ts: *Taenia saginata*, Tr: *Trichobilharzia regent*, Tso: *Taenia solium*.
(TIF)

S22 Fig Bayesian phylogenetic tree of Platyhelminth TRs. Methods for construction of phylogenetic trees see S1 Fig legend. ML model tested as LG+G (Equilibrium frequencies: Model, Proportion of invariable sites: Fixed (0.0), Number of substitution rate categories: 4, Gamma shape parameter: Estimated (0.411). Bg: *Biomphalaria glabrata*, Cs: *Clonorchis sinensis*, d: *Drosophila melanogaster*, Dl: *Dibothriocephalus latus*, Ec: *Echinococcus Canadensis*, Eca: *Echinostoma caproni*, Eg: *Echinococcus granulosus*, Em: *Echinococcus multilocularis*, Fh: *Fasciola hepatica*, Gs: *Gyrodactylus salaris*, h: *Homo sapiens*, Hd: *Hymenolepis diminuta*, Hm: Of *H*. *microstoma*, Hn: *Hymenolepis nana*, Ht: *Hydatigera taeniaeformis*, Lg: *Lottia gigantean*, Mc: *Mesocestoides corti*, Ml: *Macrostomum lignano*, Of: *Opisthorchis felineus*, Ov: *Opisthorchis viverrini*, Px: *Protopolystoma xenopodis*, Sb: *Schistosoma bovis*, Sc: *Schistosoma curassoni*, Se: *Spirometra erinaceieuropaei*, Sh: *Schistosoma haematobium*, Sj: *Schistosoma japonicum*, Sm: *Schistosoma mansoni*, Sma: *Schistosoma margrebowiei*, Smt: *Schistosoma mattheei*, Sme: *Schmidtea mediterranea*, Sr: *Schistosoma rodhaini*, Ss: *Schitocephalus solidus*, Ta: *Taenia asiatica*, Tm: *Taenia multiceps*, Ts: *Taenia saginata*, Tr: *Trichobilharzia regent*, Tso: *Taenia solium*.
(TIF)

S23 Fig Bayesian phylogenetic tree of Platyhelminth RXR. A) Bayesian phylogenetic tree of Platyhelminth RXR. Methods for construction of phylogenetic trees see S1 Fig legend. ML model tested as LG+G+I (Equilibrium frequencies: Model, Proportion of invariable sites: Estimated (0.339), Number of substitution rate categories: 4, Gamma shape parameter: Estimated (1.457). Bg: *Biomphalaria glabrata*, Cs: *Clonorchis sinensis*, d: *Drosophila melanogaster*, Dl: *Dibothriocephalus latus*, Ec: *Echinococcus Canadensis*, Eca: *Echinostoma caproni*, Eg: *Echinococcus granulosus*, Em: *Echinococcus multilocularis*, Fh: *Fasciola hepatica*, Gs: *Gyrodactylus salaris*, h: *Homo sapiens*, Lg: *Lottia gigantean*, Ml: *Macrostomum lignano*, Of: *Opisthorchis felineus*, Ov: *Opisthorchis viverrini*, Px: *Protopolystoma xenopodis*, Sb: *Schistosoma bovis*, Sc: *Schistosoma curassoni*, Se: *Spirometra erinaceieuropaei*, Sh: *Schistosoma haematobium*, Sj: *Schistosoma japonicum*, Sm: *Schistosoma mansoni*, Sma: *Schistosoma margrebowiei*, Smt: *Schistosoma mattheei*, Sme: *Schmidtea mediterranea*, Sr: *Schistosoma rodhaini*, Ss: *Schitocephalus solidus*, Tr: *Trichobilharzia regent*. B) Sequence alignment shows the intron position (red color >) in DBD of RXRs.
(TIF)

S24 Fig Bayesian phylogenetic tree of Platyhelminth Coup-TF. A) Bayesian phylogenetic tree of Platyhelminth Coup-TF. Methods for construction of phylogenetic trees see S1 Fig legend. ML model tested as FLU+G (Equilibrium frequencies: Model, Proportion of invariable sites: Fixed (0.0), Number of substitution rate categories: 4, Gamma shape parameter: Estimated (0.320). Bg: *Biomphalaria glabrata*, Cs: *Clonorchis sinensis*, d: *Drosophila melanogaster*, Dl: *Dibothriocephalus latus*, Ec: *Echinococcus Canadensis*, Eca: *Echinostoma caproni*, Eg: *Echinococcus granulosus*, Em: *Echinococcus multilocularis*, Fh: *Fasciola hepatica*, Gs: *Gyrodactylus salaris*, h: *Homo sapiens*, Lg: *Lottia gigantean*, Ml: *Macrostomum lignano*, Of: *Opisthorchis felineus*, Ov: *Opisthorchis viverrini*, Px: *Protopolystoma xenopodis*, Sb: *Schistosoma bovis*, Sc: *Schistosoma curassoni*, Se: *Spirometra erinaceieuropaei*, Sh: *Schistosoma haematobium*, Sj: *Schistosoma japonicum*, Sm: *Schistosoma mansoni*, Sma: *Schistosoma margrebowiei*, Smt: *Schistosoma mattheei*, Sme: *Schmidtea mediterranea*, Sr: *Schistosoma rodhaini*, Ss: *Schitocephalus solidus*, Ta: *Taenia asiatica*, Tm: *Taenia multiceps*, Ts: *Taenia saginata*, Tr: *Trichobilharzia regent*, Tso: *Taenia solium*. B) Sequence alignment shows the intron position (red color >) in DBD of Coup-TFs.
(TIF)

S25 Fig Bayesian phylogenetic tree of Platyhelminth NR4A. Methods for construction of phylogenetic trees see S1 Fig legend. ML model tested as FLU+G (Equilibrium frequencies: Model, Proportion of invariable sites: Fixed (0.0), Number of substitution rate categories: 4, Gamma shape parameter: Estimated (0.461). Bg: *Biomphalaria glabrata*, d: *Drosophila melanogaster*, Gs: *Gyrodactylus salaris*, h: *Homo sapiens*, Hd: *Hymenolepis diminuta*, Lg: *Lottia gigantean*, Mc: *Mesocestoides corti*, Ml: *Macrostomum lignano*, Sm: *Schistosoma mansoni*, Sme: *Schmidtea mediterranea*.
(TIF)

S26 Fig Bayesian phylogenetic tree of Platyhelminth 2DBD. Methods for construction of phylogenetic trees see S1 Fig legend. ML model tested as JTT+G+I (Equilibrium frequencies: Model, Proportion of invariable sites: Estimated (0.131), Number of substitution rate categories: 4, Gamma shape parameter: Estimated (0.832). Bg: *Biomphalaria glabrata*, Cs: *Clonorchis sinensis*, d: *Drosophila melanogaster*, Dl: *Dibothriocephalus latus*, Em: *Echinococcus multilocularis*, Fh: *Fasciola hepatica*, Gs: *Gyrodactylus salaris*, h: *Homo sapiens*, Hd: *Hymenolepis diminuta*, Hm: *H*. *microstoma*, Lg: *Lottia gigantean*, Mc: *Mesocestoides corti*, Ml: *Macrostomum lignano*, Px: *Protopolystoma xenopodis*, Se: *Spirometra erinaceieuropaei*, Sm: *Schistosoma mansoni*, Sme: *Schmidtea mediterranea*, Ss: *Schitocephalus solidus*, Tr: *Trichobilharzia regent*.
(TIF)

S1 File Lists of GenBank accession number of published NR sequences used in this study. (DOCX)

S2 File DBD sequences of NRs in 33 species of Platyhelminthes. (DOCX)

S3 File Comparison of Bayesian inference and Maximum Likelihood method to analysis of NR DBD amino sequences. (DOCX)

S4 File Sequence alignment of DBD sequence of HR96s and intron in this regions. (DOCX)

S5 File Comparison of Macrostomum lignano NRs identified in Cheng, Y., et al 2021 and this study. (XLSX)

## Abbreviations

2DBD-NR: nuclear receptor with two DBDs
AR: androgen receptor
CAR: constitutive androstane receptor
COUP-TF: chicken ovalbumin upstream promoter transcription factor
DBD: DNA binding domain
DSF: *Drosophila* dissatisfaction gene
E75: *Drosophila* ecdysone-induced protein 75
E78: *Drosophila* ecdysone-induced protein 78
EAR: V-erbA-related gene
EGON: embryonic gonad protein
ER: estrogen receptor
ERR: estrogen related receptor
FTZ-F1: fushi tarazu-factor 1
FXR: farnesoid X receptor
GCNF: germ cell nuclear factor
GR: glucuronide receptor
HNF-4: hepatocyte nuclear factor 4
HR96: *Drosophila* hormone receptor 96
LBD: ligand binding domain
LRH-1: liver receptor homologue 1
LXR: liver X receptor
MR: mineralocorticoid receptor
NGFI-B: nerve growth factor-induced B
NHR236: nematode nuclear receptor 236
NOR1: neuron-derived orphan receptor 1
NR: Nuclear receptor
Nurr1: nuclear receptor related 1
PNR: photoreceptor-specific nuclear receptor
PPAR: peroxisome proliferators-activated receptor
PR: progesterone receptor
PXR: pregnane X receptor
RAR: retinoic acid receptor
Rev-erb: nuclear receptor-related protein coded on the opposite strand of the thyroid hormone receptor gene
ROR: retinoid-related orphan receptor
RXR: retinoid X receptor
SF1: Steroidogenic factor 1
SR: steroid receptor
SupNRs: supplementary nuclear receptors
SVP: seven up
TLL: *Drosophila* tailless gene
TLX: homologue of the *Drosophila* tailless gene
TR: thyroid hormone recepto
TR2/4: testicular receptor 2/4
USP: ultraspiracle
VDR: vitamin D receptor
