# Correction: Identification and evolution of nuclear receptors in Platyhelminths

**DOI:** 10.1371/journal.pone.0315840

**Published:** 2024-12-12

**Authors:** Wenjie Wu, Philip T. LoVerde

There is an error in Table 2. The first two columns in the second page of the table are missing. Please see the correct version of Table 2 here.

**Table 2:** Nuclear receptors identified in Platyhelminths

				**Platyhelminths**			
**CLASS**				**Rhabditophora**		**Monogenea**	
**ORDER**				Macrostomida	Tricladida	Polyopisthocotylea	Monopisthocotylea
**FAMILY**				Macrostomidae	Dugesidae	Polystomatidae	Gyrodactylidae
**Genus/** **Species**		*H*. *sapiens*	*D*. *melanogaster*	*Macrostomum* *lignano*	*Schmidtea* *mediterranea*	*Protopolystoma* *xenopodis*	*Gyrodactylus* *salaris*
NR1A	TR	2		2	3	1	1
NR1B	RAR	3					
NR1C	PPAR	3					
NR1D	Rev-erb/E75	2	1				
NR1E	E78		1	2		1	1
NR1F	ROR	3	1				
NR1H	ECR/LXR/ FXR	3	1				
NR1I	VDR/PXR/CAR	3					
NR1J	DHR96		1	16	3	3	2
NR1a				1	1	1	
NR1b					1	1	
NR2A	HNF4	2	1	1	1	1	1
NR2B	RXR/USP	3	1	2	2	2	1
NR2C/D	TR2/TR4/DHR78	2	1	2	1	1	1
NR2E1/2	TLX/TLL	1	1	1	1	1	1
NR2E3	PNR	1	1	1	1	1	1
NR2E4	DSF		1	1	1	1	1
NR2E5	fax-1		1	1			
NR2E new	NHR236			2	1	1	
NR2F	COUP-TF/SVP	3	1	2	2	2	2
NR3A	ER	2					
NR3B	ERR	3	1	4			
NR3C	MR/PR/AR/GR	4					
NR4A	NGFI-B/DHR38	3	1	2	2	1	1
NR5A	FTZ-F1	2	1		1	1	
NR5B	DHR39		1	1	1	1	1
NR6A	GCNF1	1	1	1			
NR0A/B	Knirps/KNRL/ EGON/DAX1/SHP	2	3				
2DBD-NR	2DBD-NR			2	4	3	3
Other				17	1		1
**Total**	** **	**48**	**21**	**61**	**27**	**23**	**18**

		**Platyhelminths**							
**CLASS**		**Cestoda**						**Trematoda**	
**ORDER**		**Cyclophyllidea**			**Diphyllobothriidea**			**Opistorchida** **Echinostomida**	**Strigeidida**
**FAMILY**		Hymenolepididae	Mesocestoididae	Taeniidae	Diphyllobothriidae				
**Genus/** **Species**		*Hymenolepis*	*Mesocestoides* *corti*	*Echinococcu* *Hydatigera* *Taenia*	*Diphyllobothrium* *latum*	*Schistocephalus* *solidus*	*Spirometra* *erinaceieuropaei*	* *	* *
NR1A	TR	1	2	1	1	1	1	2	2
NR1B	RAR								
NR1C	PPAR								
NR1D	Rev-erb/E75								
NR1E	E78	1	1	1	1	1	1	1	1
NR1F	ROR								
NR1H	ECR/LXR/ FXR								
NR1I	VDR/PXR/CAR								
NR1J	DHR96	3	3	3	3	3	3	3	2
NR1a			1			1	1	1	1
NR1b		1	1	1	1	1	1		
NR2A	HNF4	1	1	1	1	1	1	1	1
NR2B	RXR/USP				1	2	1	2	2
NR2C/D	TR2/TR4/DHR78	1	1	1	1	1	1	1	1
NR2E1/2	TLX/TLL	1	1	1	1	1	1	1	1
NR2E3	PNR				1		1	1	1
NR2E4	DSF							1	1
NR2E5	fax-1								
NR2E new	NHR236								
NR2F	COUP-TF/SVP			1	1	1	1	2	2
NR3A	ER								
NR3B	ERR								
NR3C	MR/PR/AR/GR								
NR4A	NGFI-B/DHR38	1	1	1	1	1	1	1	1
NR5A	FTZ-F1			1	1	1	1	1	1
NR5B	DHR39	1	1	1	1	1	1	1	1
NR6A	GCNF1								
NR0A/B	Knirps/KNRL/ EGON/DAX1/SHP								
2DBD-NR	2DBD-NR	3	3	3	3	3	3	3	3
Other		1	5	1	1	1	1		
**Total**		**15**	**21**	**17**	**19**	**20**	**20**	**22**	**21**
