# Supplementary figures and images for: Identification and evolution of nuclear receptors in Platyhelminths
Source: PLoS One. 2021 Aug 13;16(8):e0250750. doi: 10.1371/journal.pone.0250750 (PMC8363021; doi:10.1371/journal.pone.0250750)

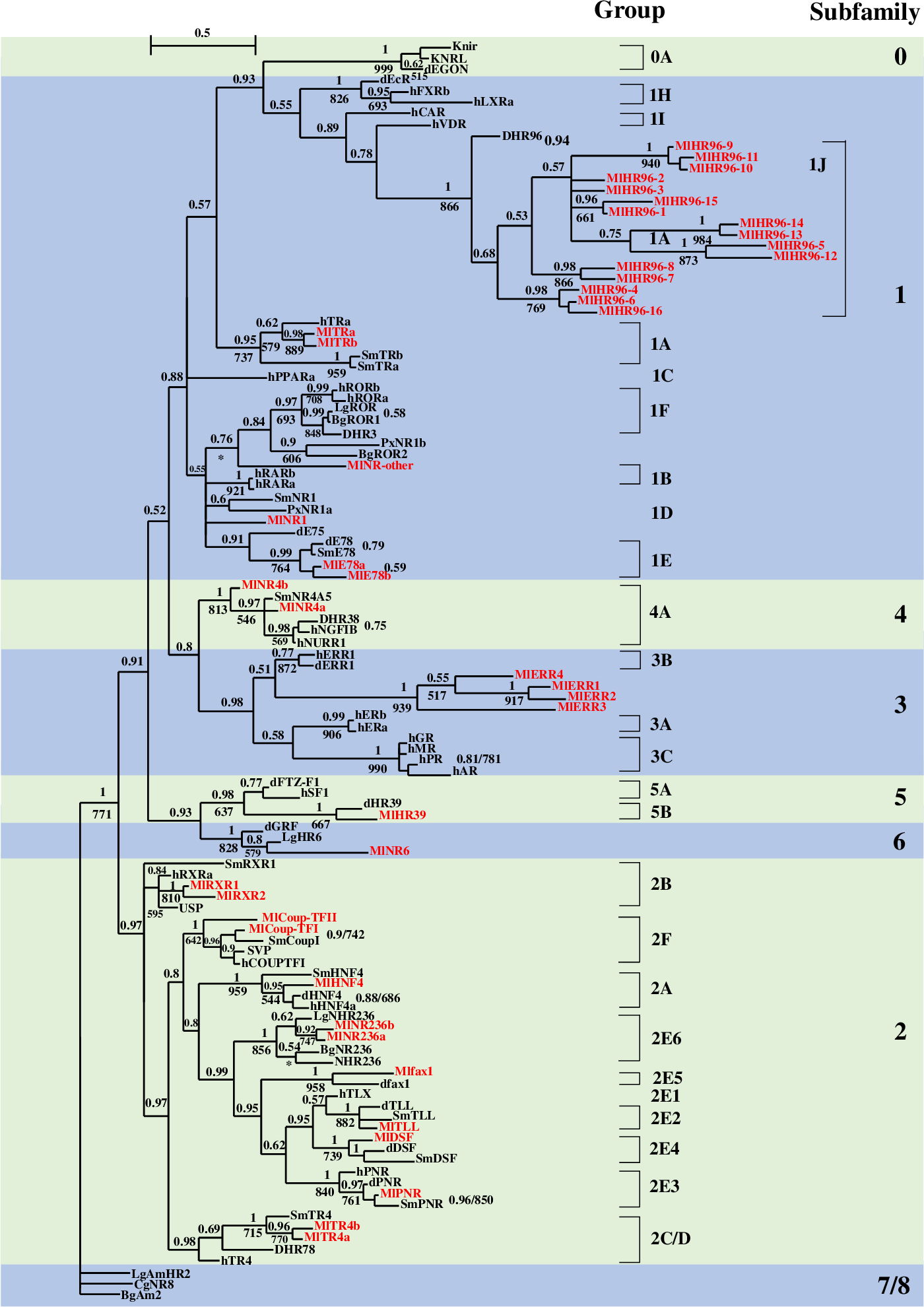

Supplement: S1 Fig — The Bayesian tree was constructed with the deduced amino sequences of the DNA binding domain (DBD) with a mix amino acid replacement model + invgamma rates. The PPs values are shown above each branch, branches under the PPs 0.5 are shown as polytomies. The same data set is also tested by ML method using PHYML (v2.4.4) under LG+G+I substitution model (Equilibrium frequencies: Model, Proportion of invariable sites: Estimated (0.158), Number of substitution rate categories: 4, Gamma shape parameter: Estimated (0.956). Support values for the tree are obtained by bootstrapping a 1,000 replicates and bootstrap values above 500 and are indicated below each branch (or after MrBAYES BPPs separated by Slash). Star indicates the node obtained by Bayesian inference which is different from that obtained by ML method. Bg: Biomphalaria glabrata, Cg: Crassostrea gigas, d: Drosophila melanogaster, h: Homo sapiens, Lg: Lottia gigantean, Ml: Macrostomum lignano, Px: Protopolystoma xenopodis, Sm: Schistosoma mansoni. Red highlighted NRs show M. lignano NRs. (TIF) [file pone.0250750.s001.tif]

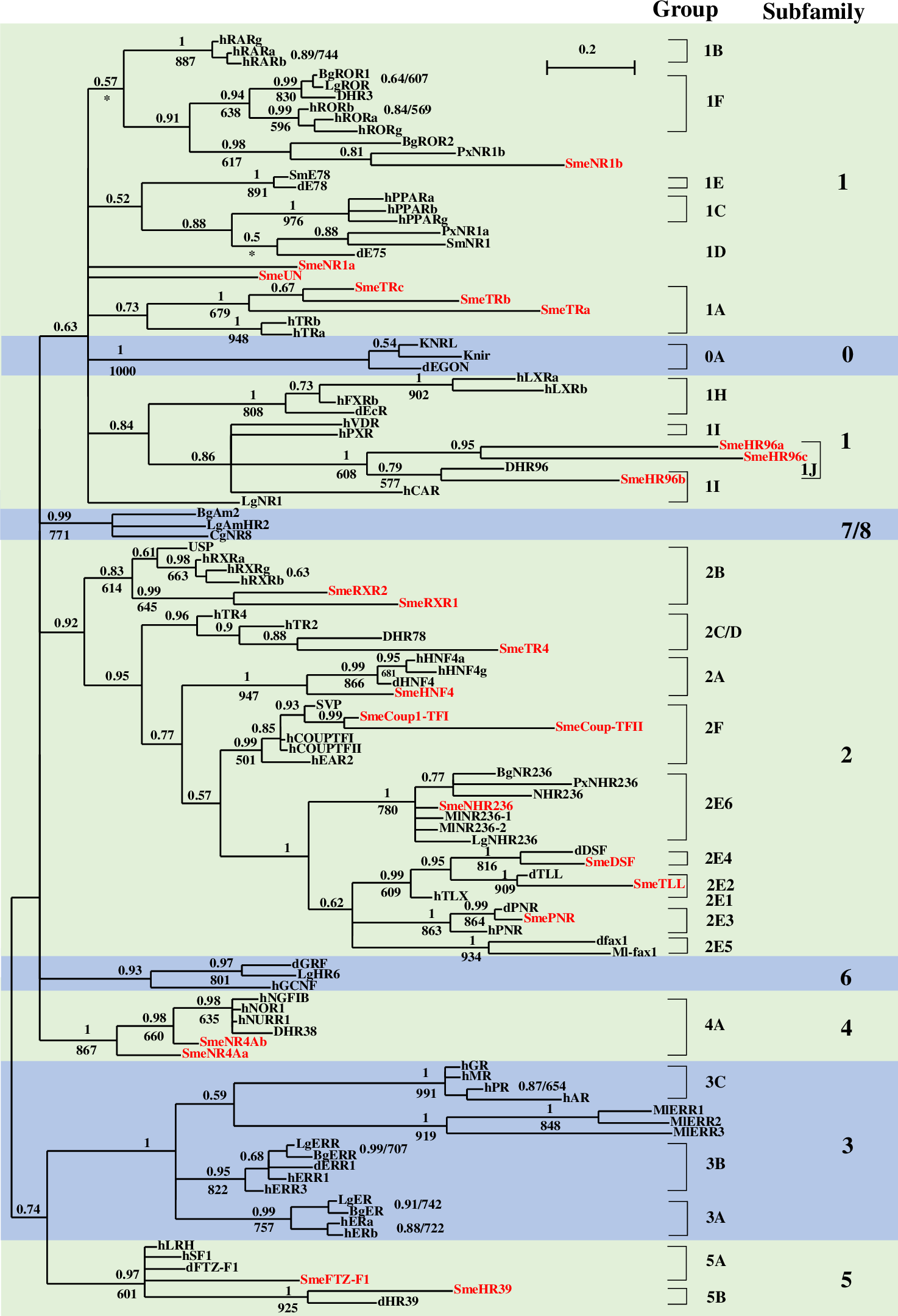

Supplement: S2 Fig — Methods for construction of phylogenetic trees see S1 Fig legend. ML model tested as LG+G+I (Equilibrium frequencies: Model, Proportion of invariable sites: Estimated (0.165), Number of substitution rate categories: 4, Gamma shape parameter: Estimated (0.751). Bg: Biomphalaria glabrata, Cg: Crassostrea gigas, d: Drosophila melanogaster, h: Homo sapiens, Lg: Lottia gigantean, Px: Protopolystoma xenopodis, Sm: Schistosoma mansoni, Sme: Schmidtea mediterranea. Red highlighted NRs show S. mediterranea NRs. (TIF) [file pone.0250750.s002.tif]

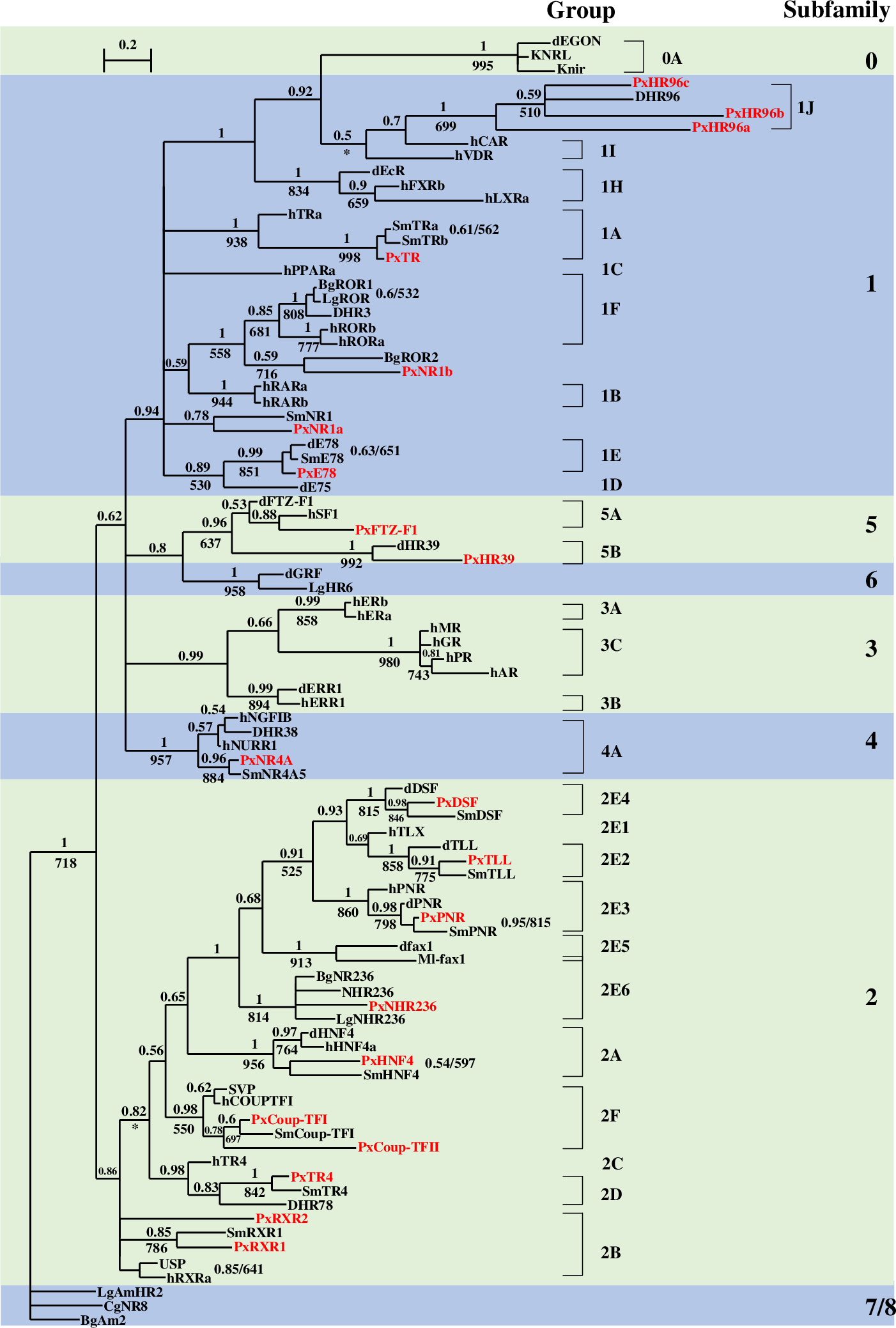

Supplement: S3 Fig — Methods for construction of phylogenetic trees see S1 Fig legend. ML model tested as LG+G+I (Equilibrium frequencies: Model, Proportion of invariable sites: Estimated (0.119), Number of substitution rate categories: 4, Gamma shape parameter: Estimated (0.691). Bg: Biomphalaria glabrata, Cg: Crassostrea gigas, d: Drosophila melanogaster, h: Homo sapiens, Lg: Lottia gigantean, Px: Protopolystoma xenopodis, Sm: Schistosoma mansoni. Red highlighted NRs show P. xenopodis NRs. (TIF) [file pone.0250750.s003.tif]

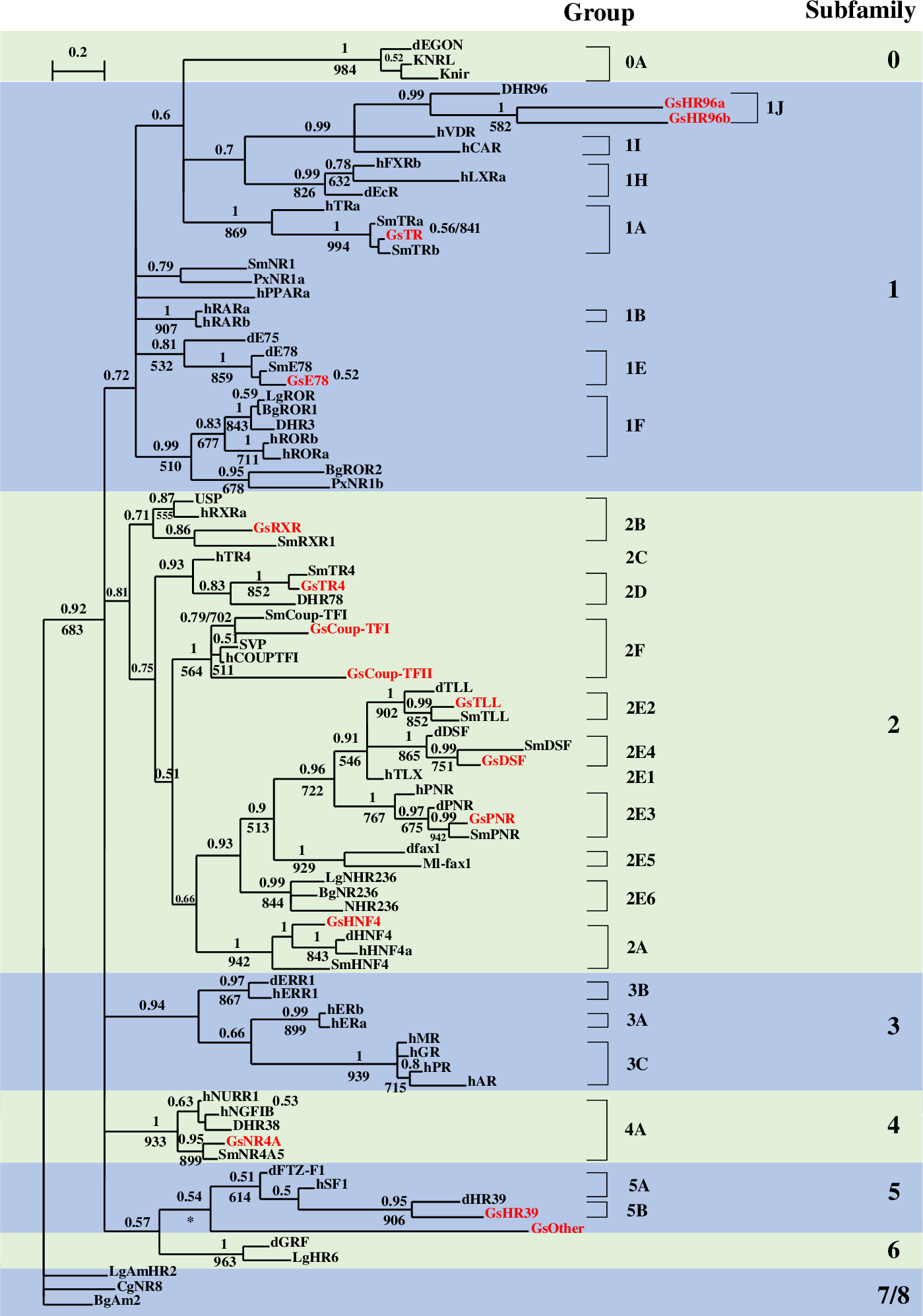

Supplement: S4 Fig — Methods for construction of phylogenetic trees see S1 Fig legend. ML model tested as LG+G+I (Equilibrium frequencies: Model, Proportion of invariable sites: Estimated (0.119), Number of substitution rate categories: 4, Gamma shape parameter: Estimated (0.691). Bg: Biomphalaria glabrata, Cg: Crassostrea gigas, d: Drosophila melanogaster, Gs: Gyrodactylus salaris, h: Homo sapiens, Lg: Lottia gigantean, Px: Protopolystoma xenopodis, Sm: Schistosoma mansoni. Red highlighted NRs show G. salaris NRs. (TIF) [file pone.0250750.s004.tif]

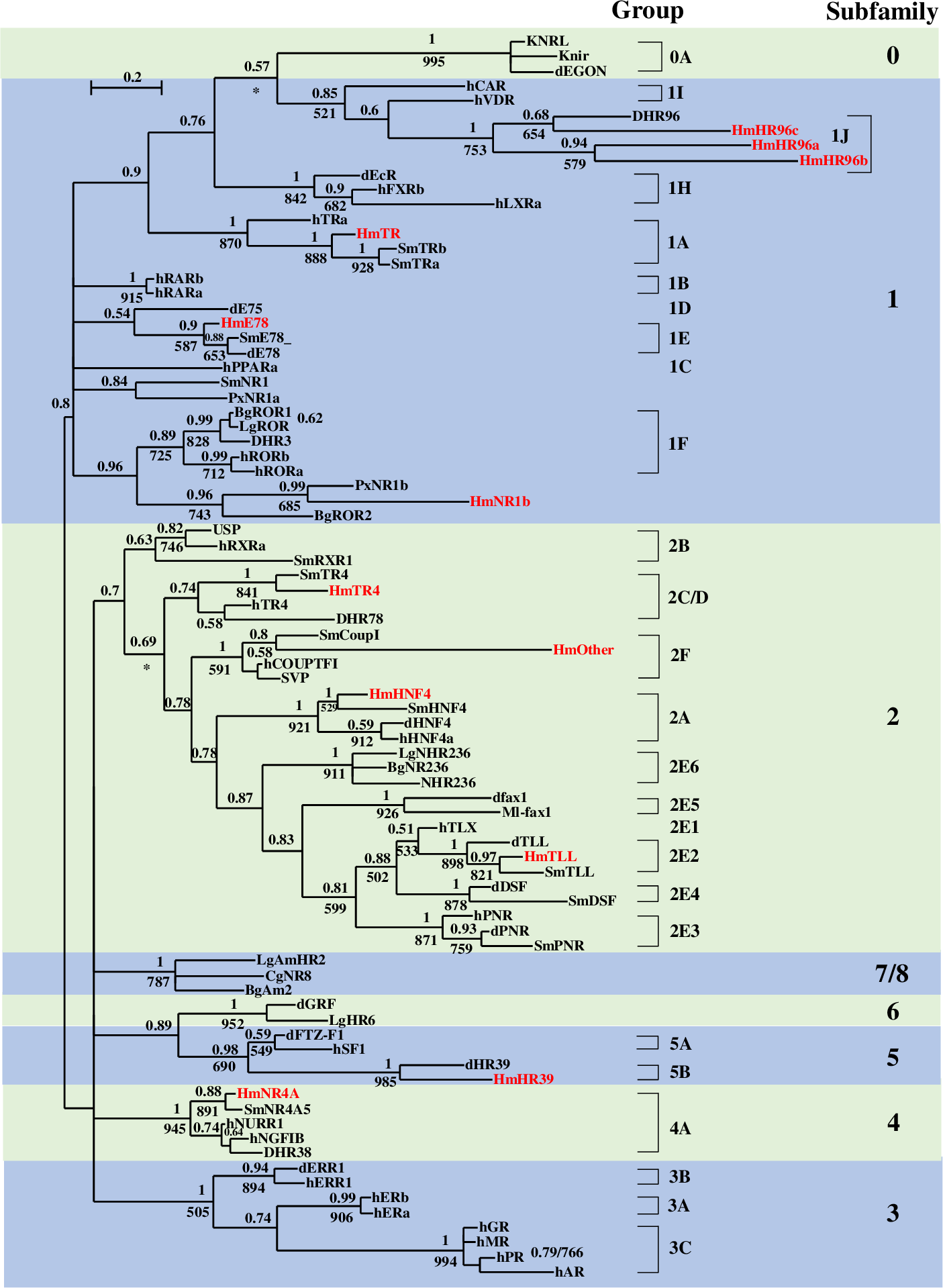

Supplement: S5 Fig — The phylogenetic tree of H. microstoma NRs represents all analyzed Hymenolepis species because of the highly conserved DBD sequences in these species. Methods for construction of phylogenetic trees see S1 Fig legend. ML model tested as LG+G+I (Equilibrium frequencies: Model, Proportion of invariable sites: Estimated (0.092), Number of substitution rate categories: 4, Gamma shape parameter: Estimated (0.709). Bg: Biomphalaria glabrata, Cg: Crassostrea gigas, d: Drosophila melanogaster, h: Homo sapiens, Hm: Hymenolepis microstoma, Lg: Lottia gigantean, Px: Protopolystoma xenopodis, Sm: Schistosoma mansoni. Red highlighted NRs show H. microstoma NRs. (TIF) [file pone.0250750.s005.tif]

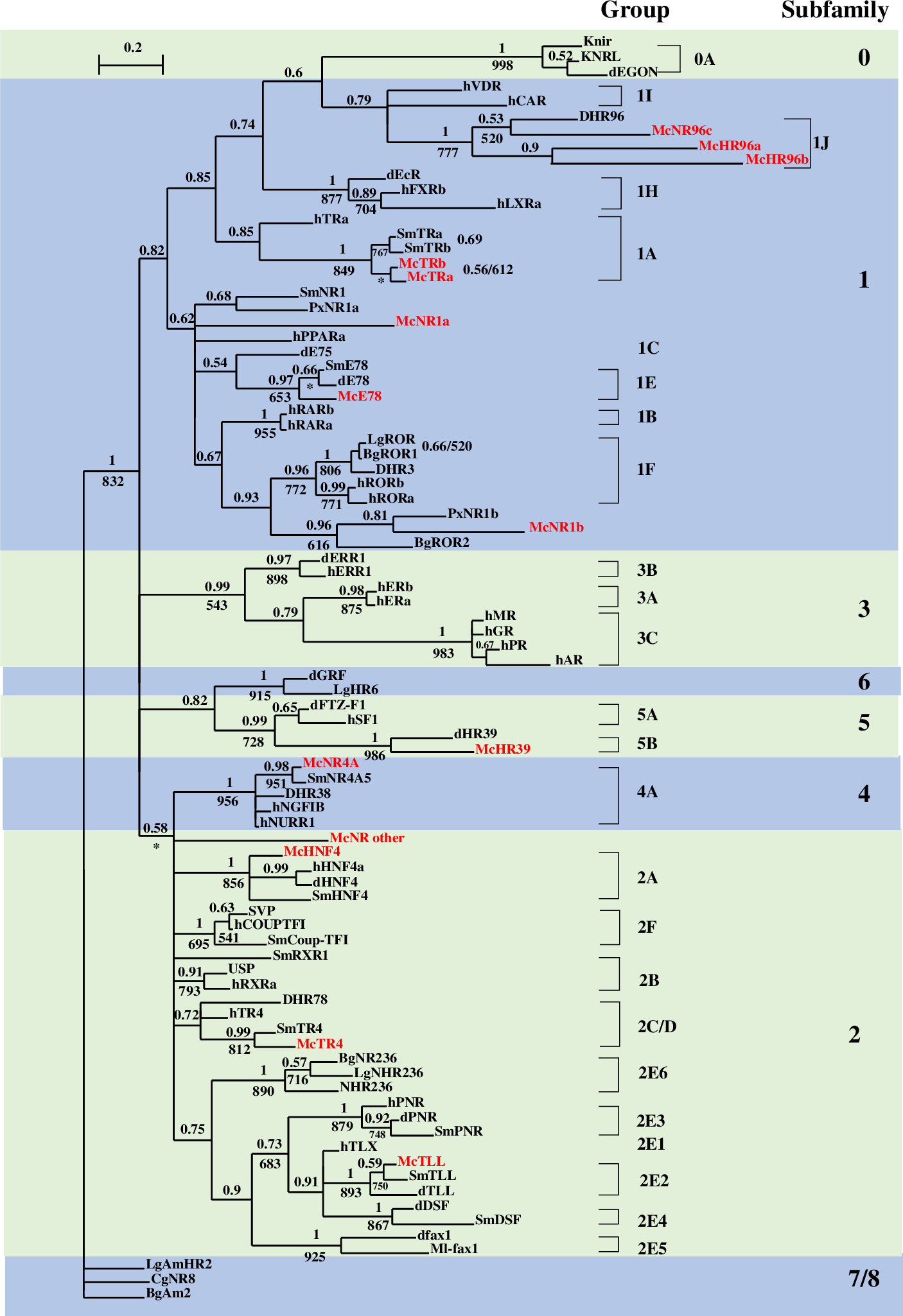

Supplement: S6 Fig — Methods for construction of phylogenetic trees see S1 Fig legend. ML model tested as LG+G+I (Equilibrium frequencies: Model, Proportion of invariable sites: Estimated (0.073), Number of substitution rate categories: 4, Gamma shape parameter: Estimated (0.782). Bg: Biomphalaria glabrata, Cg: Crassostrea gigas, d: Drosophila melanogaster, h: Homo sapiens, Lg: Lottia gigantean, Mc: Mesocestoides corti, Px: Protopolystoma xenopodis, Sm: Schistosoma mansoni. Red highlighted NRs show M. corti NRs. (TIF) [file pone.0250750.s006.tif]

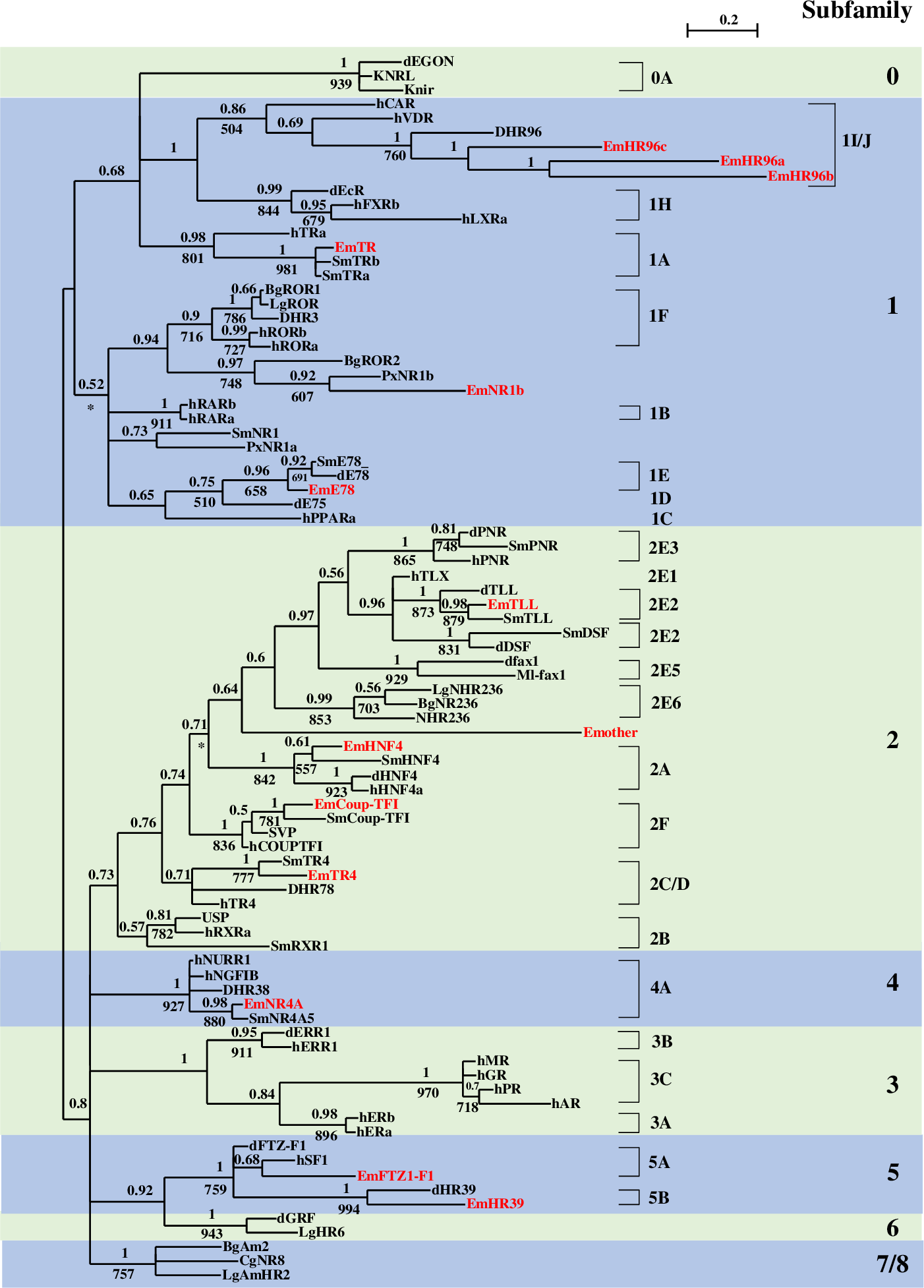

Supplement: S7 Fig — Methods for construction of phylogenetic trees see S1 Fig legend. ML model tested as LG+G+I (Equilibrium frequencies: Model, Proportion of invariable sites: Estimated (0.103), Number of substitution rate categories: 4, Gamma shape parameter: Estimated (0.735). Bg: Biomphalaria glabrata, Cg: Crassostrea gigas, d: Drosophila melanogaster, h: Homo sapiens, Lg: Lottia gigantean, Em: Echinococcus multilocularis, Px: Protopolystoma xenopodis, Sm: Schistosoma mansoni. Red highlighted NRs show E. multilocularis NRs. (TIF) [file pone.0250750.s007.tif]

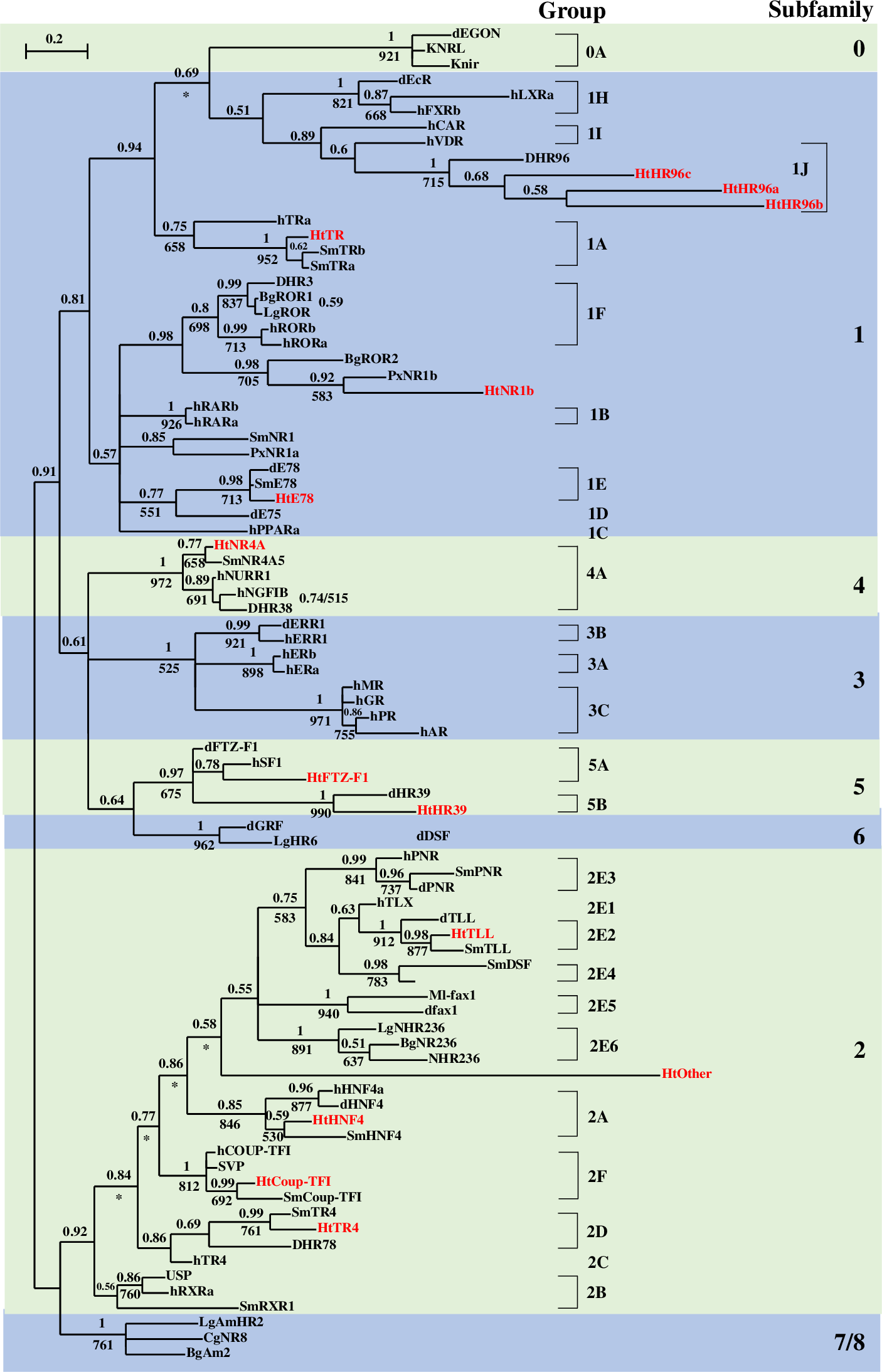

Supplement: S8 Fig — Methods for construction of phylogenetic trees see S1 Fig legend. ML model tested as LG+G+I (Equilibrium frequencies: Model, Proportion of invariable sites: Estimated (0.093), Number of substitution rate categories: 4, Gamma shape parameter: Estimated (0.717). Bg: Biomphalaria glabrata, Cg: Crassostrea gigas, d: Drosophila melanogaster, h: Homo sapiens, Ht: Hydatigera taeniaeformis, Lg: Lottia gigantean, Px: Protopolystoma xenopodis, Sm: Schistosoma mansoni. Red highlighted NRs show H. taeniaeformis NRs. (TIF) [file pone.0250750.s008.tif]

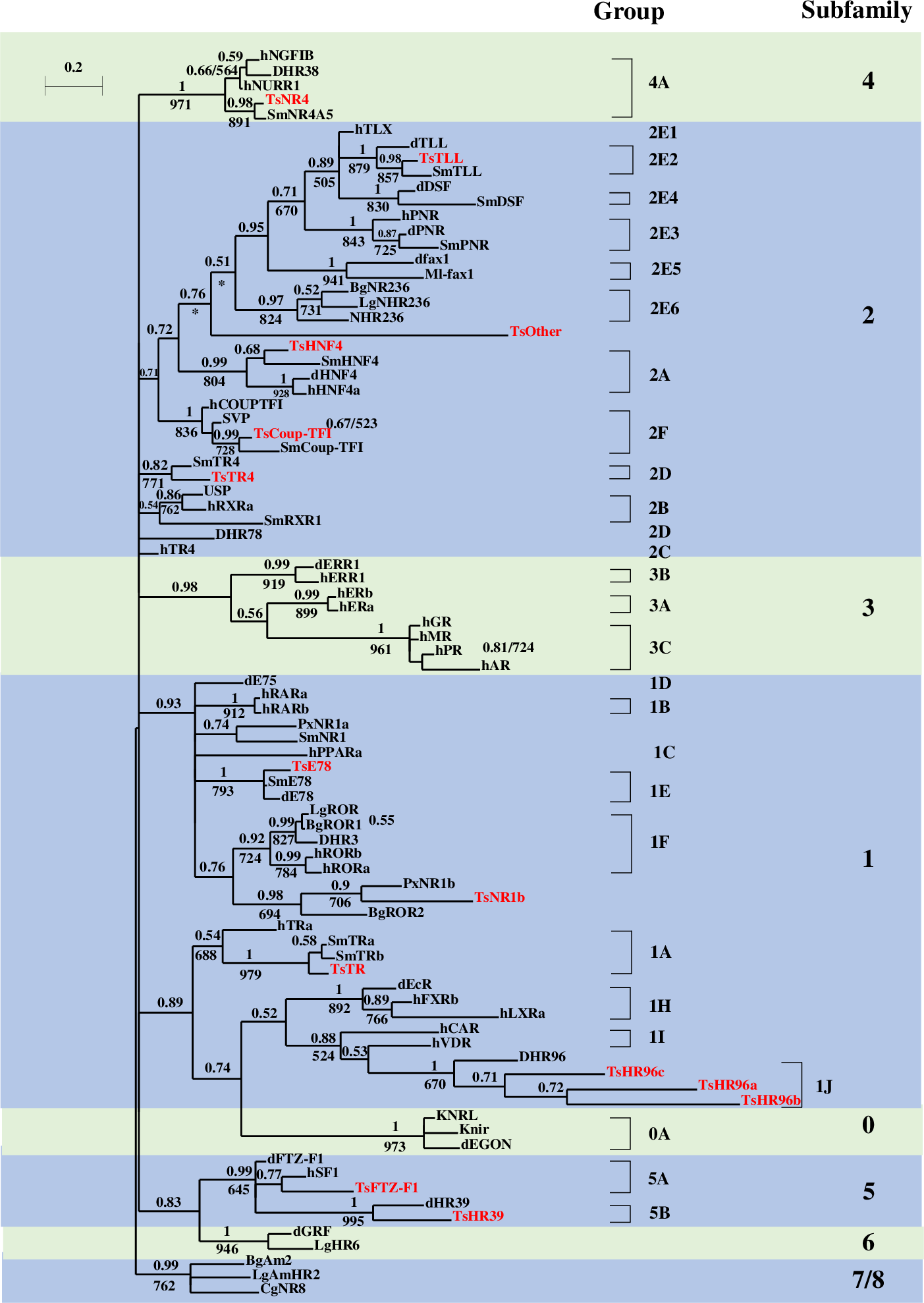

Supplement: S9 Fig — Methods for construction of phylogenetic trees see S1 Fig legend. ML model tested as LG+G+I (Equilibrium frequencies: Model, Proportion of invariable sites: Estimated (0.123), Number of substitution rate categories: 4, Gamma shape parameter: Estimated (0.898). Bg: Biomphalaria glabrata, Cg: Crassostrea gigas, d: Drosophila melanogaster, h: Homo sapiens, Lg: Lottia gigantean, Px: Protopolystoma xenopodis, Sm: Schistosoma mansoni, Ts: Taenia saginata. Red highlighted NRs show T. saginata NRs. (TIF) [file pone.0250750.s009.tif]

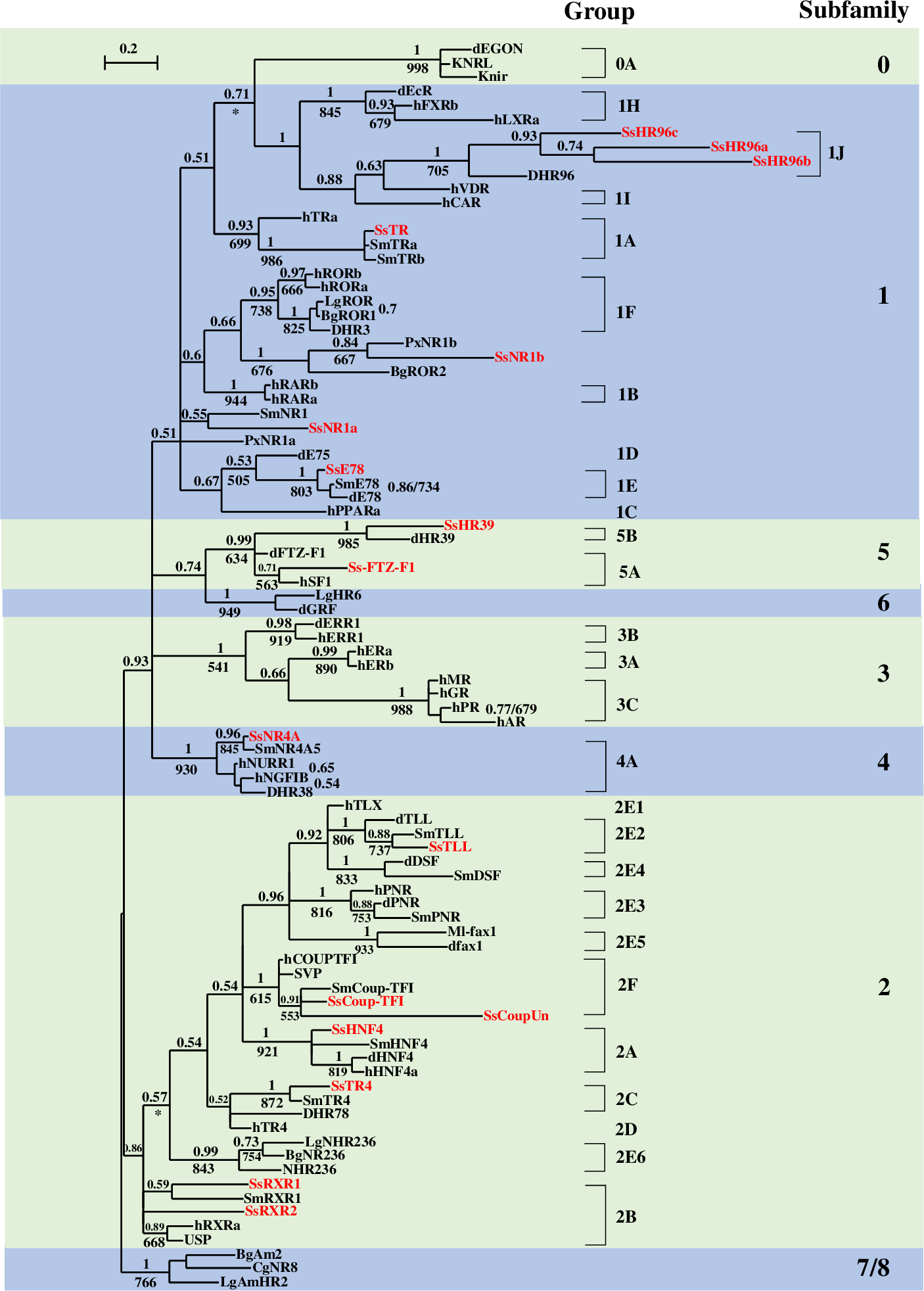

Supplement: S10 Fig — Methods for construction of phylogenetic trees see S1 Fig legend. ML model tested as LG+G+I (Equilibrium frequencies: Model, Proportion of invariable sites: Estimated (0.129), Number of substitution rate categories: 4, Gamma shape parameter: Estimated (0.744). Bg: Biomphalaria glabrata, Cg: Crassostrea gigas, d: Drosophila melanogaster, h: Homo sapiens, Ss: Schitocephalus solidus, Lg: Lottia gigantean, Px: Protopolystoma xenopodis, Sm: Schistosoma mansoni. Red highlighted NRs show T. saginata NRs. (TIF) [file pone.0250750.s010.tif]

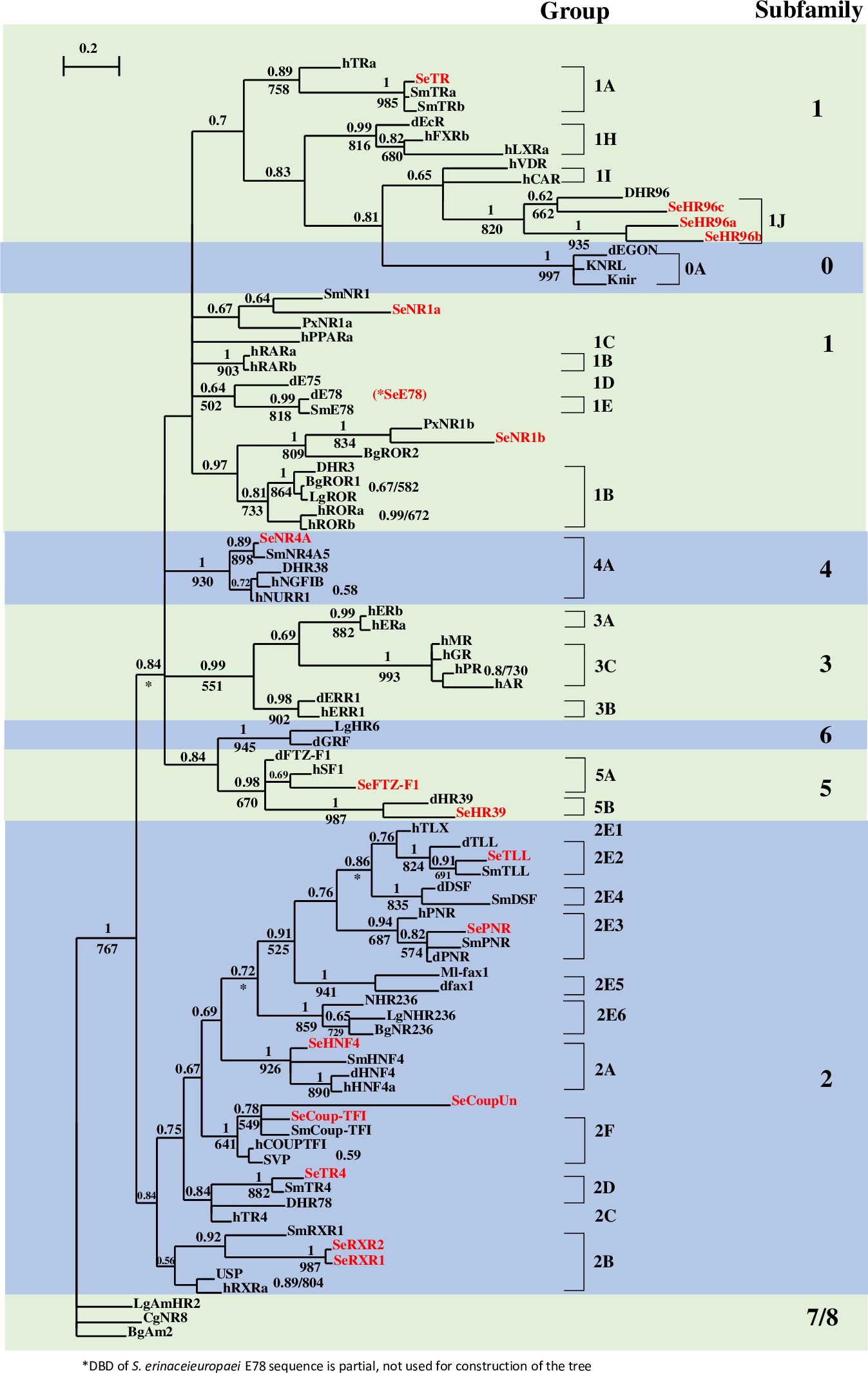

Supplement: S11 Fig — Methods for construction of phylogenetic trees see S1 Fig legend. ML model tested as LG+G+I (Equilibrium frequencies: Model, Proportion of invariable sites: Estimated (0.129), Number of substitution rate categories: 4, Gamma shape parameter: Estimated (0.701). Bg: Biomphalaria glabrata, Cg: Crassostrea gigas, d: Drosophila melanogaster, h: Homo sapiens, Lg: Lottia gigantean, Px: Protopolystoma xenopodis, Se: Spirometra erinaceieuropaei, Sm: Schistosoma mansoni. DBD of E78 sequence is partial, not used for construction of the tree. Red highlighted NRs show S. erinaceieuropaei NRs. (TIF) [file pone.0250750.s011.tif]

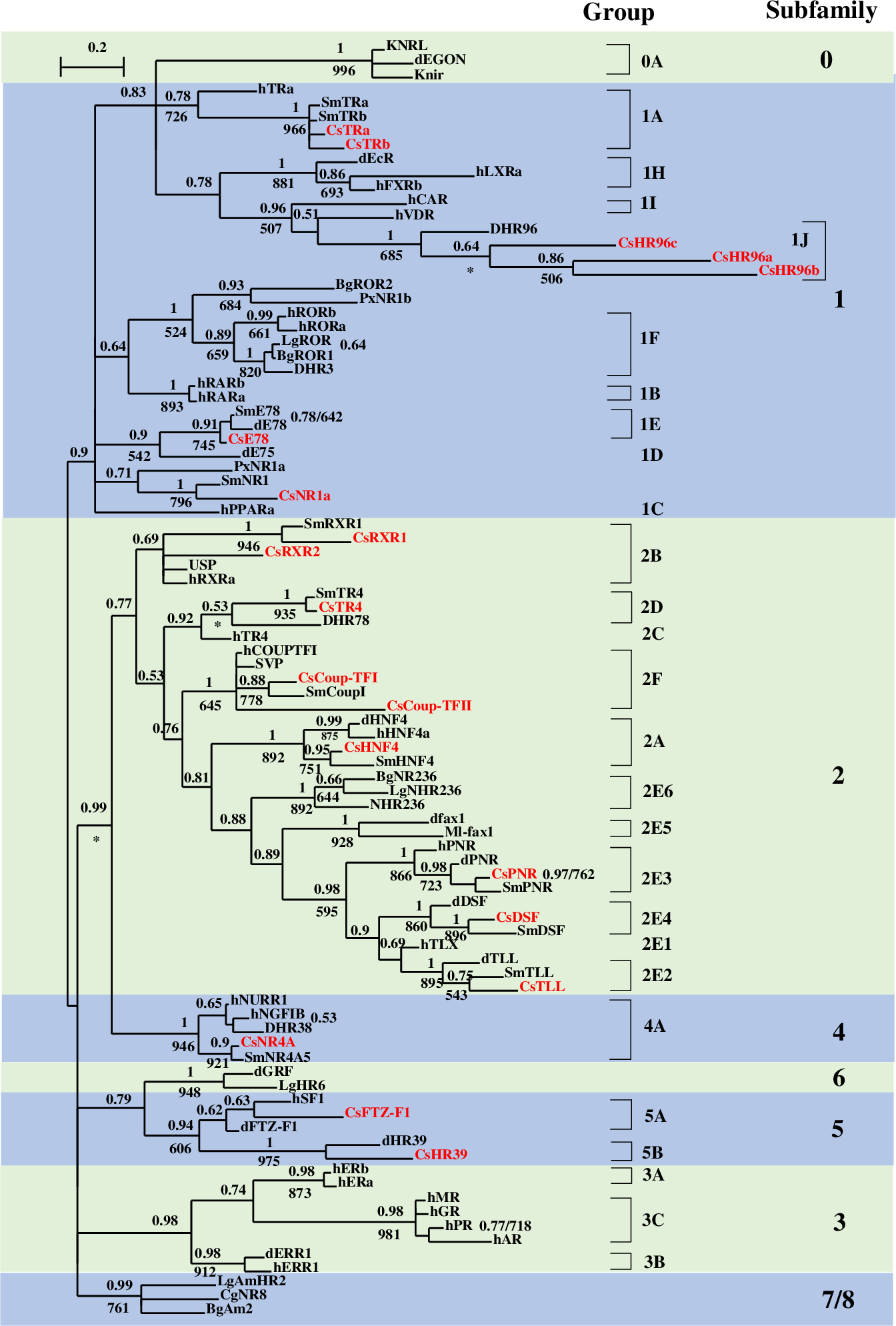

Supplement: S12 Fig — Methods for construction of phylogenetic trees see S1 Fig legend. ML model tested as LG+G+I (Equilibrium frequencies: Model, Proportion of invariable sites: Estimated (0.127), Number of substitution rate categories: 4, Gamma shape parameter: Estimated (0.747). Bg: Biomphalaria glabrata, Cg: Crassostrea gigas, Cs: Clonorchis sinensis, d: Drosophila melanogaster, h: Homo sapiens, Lg: Lottia gigantean, Px: Protopolystoma xenopodis, Sm: Schistosoma mansoni. Red highlighted NRs show C. sinensis NRs. (TIF) [file pone.0250750.s012.tif]

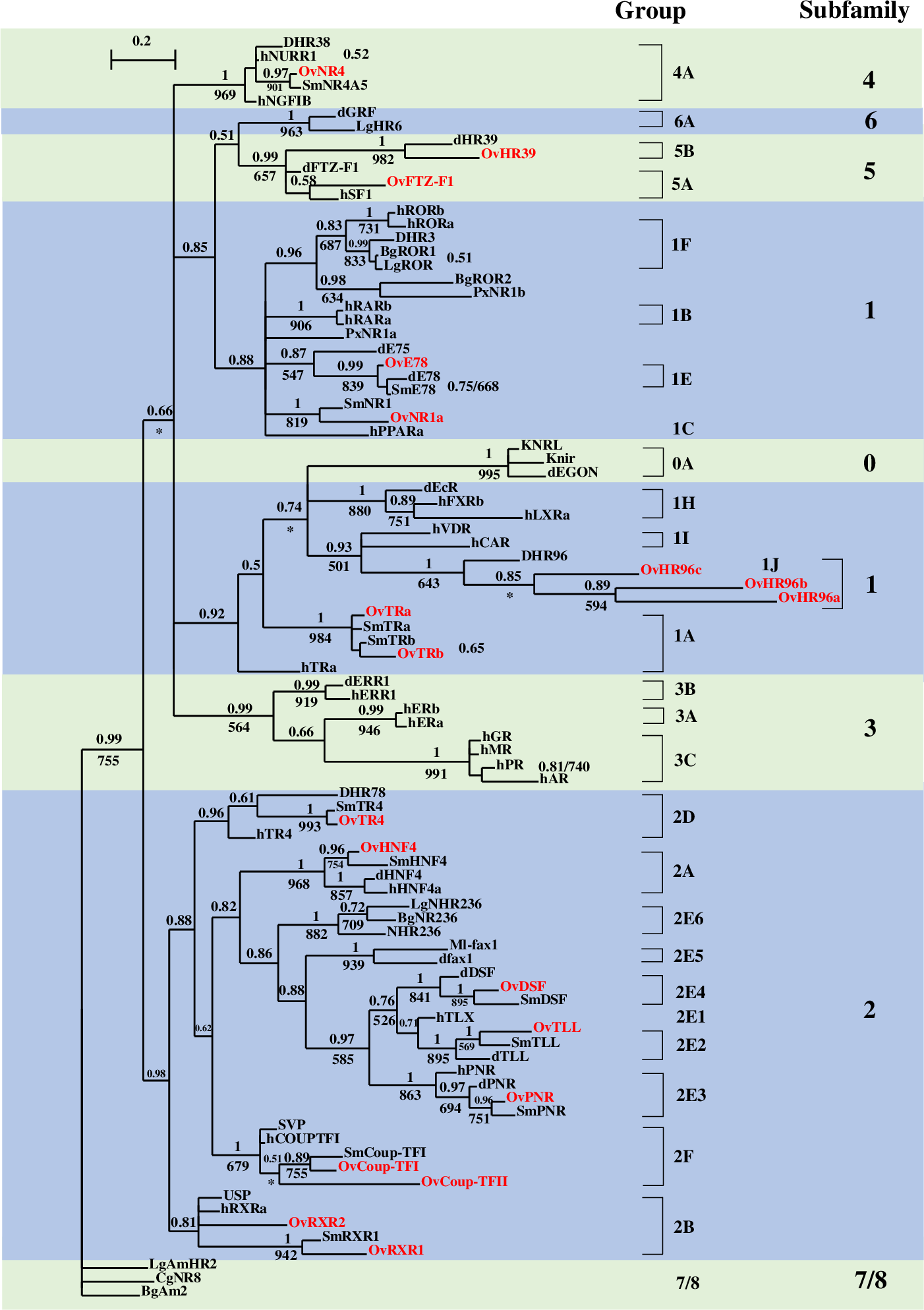

Supplement: S13 Fig — The phylogenetic tree of O viverrini NRs represents Opisthorchis viverrini and O. felineus because of the highly conserved DBD sequences in these two species. Methods for construction of phylogenetic trees see S1 Fig legend. ML model tested as LG+G+I (Equilibrium frequencies: Model, Proportion of invariable sites: Estimated (0.123), Number of substitution rate categories: 4, Gamma shape parameter: Estimated (0.830). Bg: Biomphalaria glabrata, Cg: Crassostrea gigas, d: Drosophila melanogaster, h: Homo sapiens, Lg: Lottia gigantean, Ov: Opisthorchis viverrini, Px: Protopolystoma xenopodis, Sm: Schistosoma mansoni. Red highlighted NRs show O. viverrini NRs. (TIF) [file pone.0250750.s013.tif]

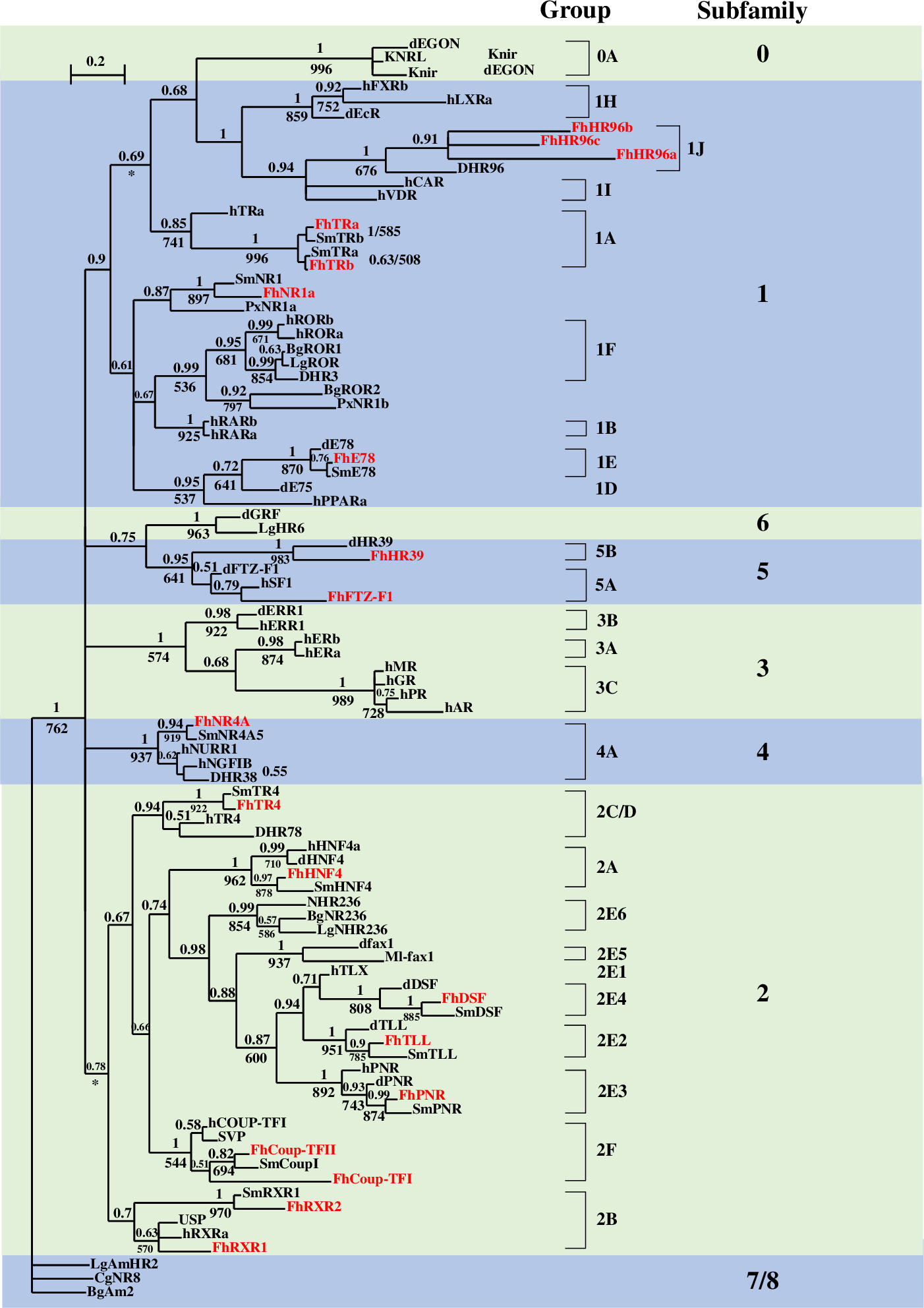

Supplement: S14 Fig — Methods for construction of phylogenetic trees see S1 Fig legend. ML model tested as LG+G+I (Equilibrium frequencies: Model, Proportion of invariable sites: Estimated (0.133), Number of substitution rate categories: 4, Gamma shape parameter: Estimated (0.834). Bg: Biomphalaria glabrata, Cg: Crassostrea gigas, d: Drosophila melanogaster, Fh: Fasciola hepatica, h: Homo sapiens, Lg: Lottia gigantean, Px: Protopolystoma xenopodis, Sm: Schistosoma mansoni. Red highlighted NRs show F. hepatica NRs. (TIF) [file pone.0250750.s014.tif]

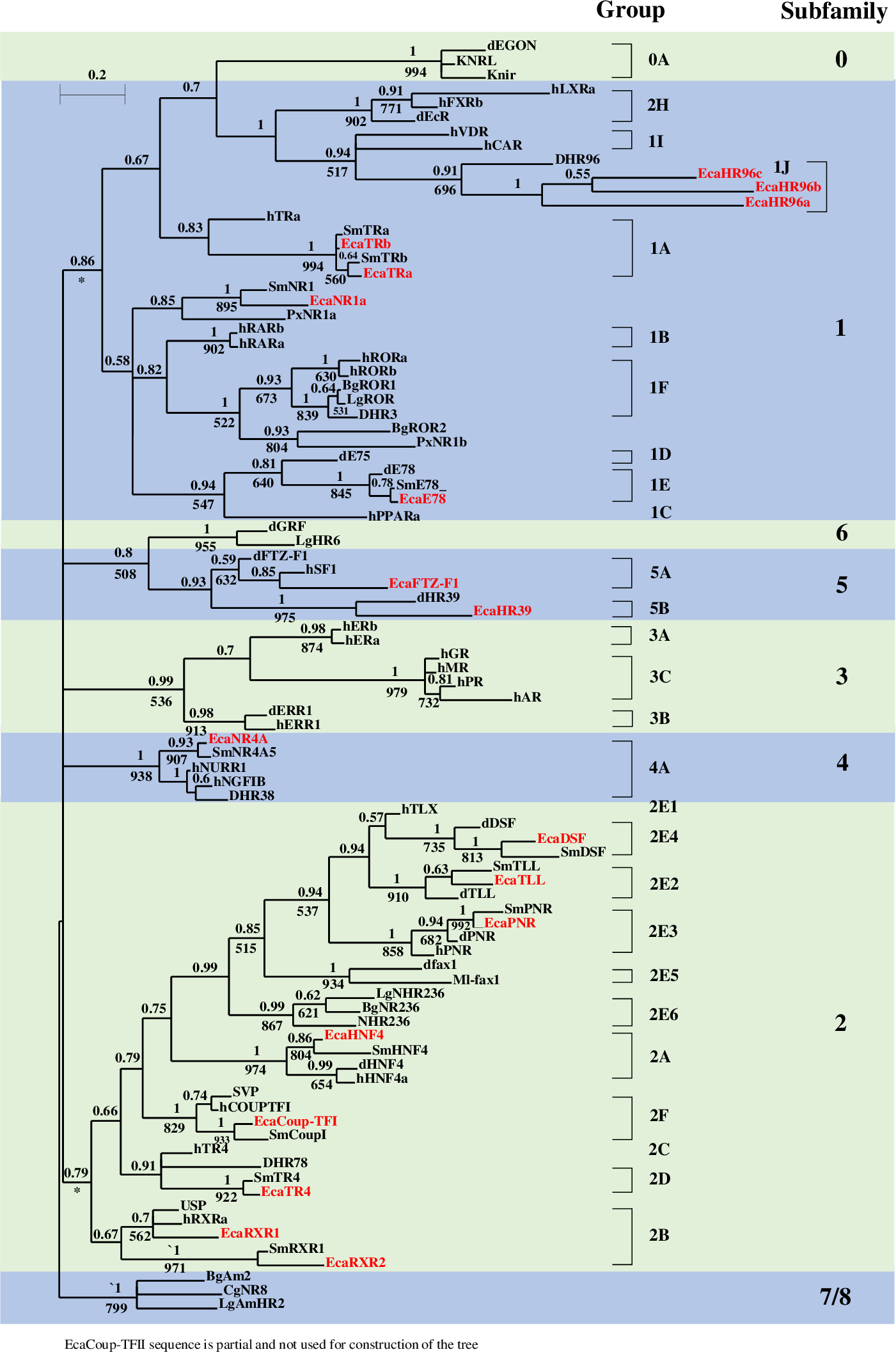

Supplement: S15 Fig — Methods for construction of phylogenetic trees see S1 Fig legend. ML model tested as LG+G+I (Equilibrium frequencies: Model, Proportion of invariable sites: Estimated (0.127), Number of substitution rate categories: 4, Gamma shape parameter: Estimated (0.802). Bg: Biomphalaria glabrata, Cg: Crassostrea gigas, d: Drosophila melanogaster, Eca: Echinostoma caproni, h: Homo sapiens, Lg: Lottia gigantean, Px: Protopolystoma xenopodis, Sm: Schistosoma mansoni. EcaCoup-TFII sequence is partial and not used for construction of the tree. Red highlighted NRs show E. caproni NRs. (TIF) [file pone.0250750.s015.tif]

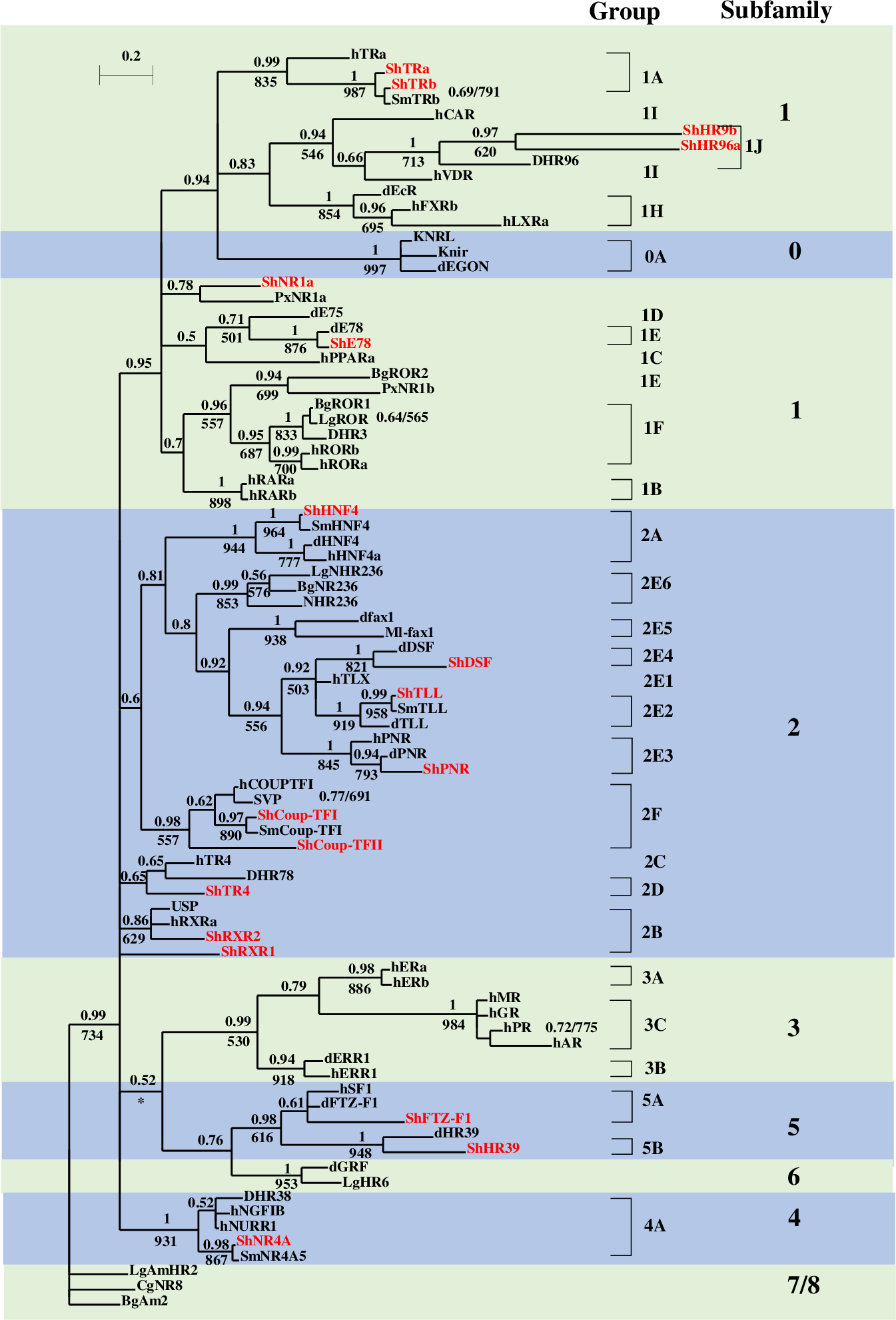

Supplement: S16 Fig — The phylogenetic tree of S haematobium NRs represents all analyzed Schistosoma species because of the highly conserved DBD sequences in these species. Methods for construction of phylogenetic trees see S1 Fig legend. ML model tested as LG+G+I (Equilibrium frequencies: Model, Proportion of invariable sites: Estimated (0.119), Number of substitution rate categories: 4, Gamma shape parameter: Estimated (0.714). Bg: Biomphalaria glabrata, Cg: Crassostrea gigas, d: Drosophila melanogaster, h: Homo sapiens, Lg: Lottia gigantean, Px: Protopolystoma xenopodis, Sh: Schistosoma haematobium, Sm: Schistosoma mansoni. Red highlighted NRs show S. haematobium NRs. (TIF) [file pone.0250750.s016.tif]

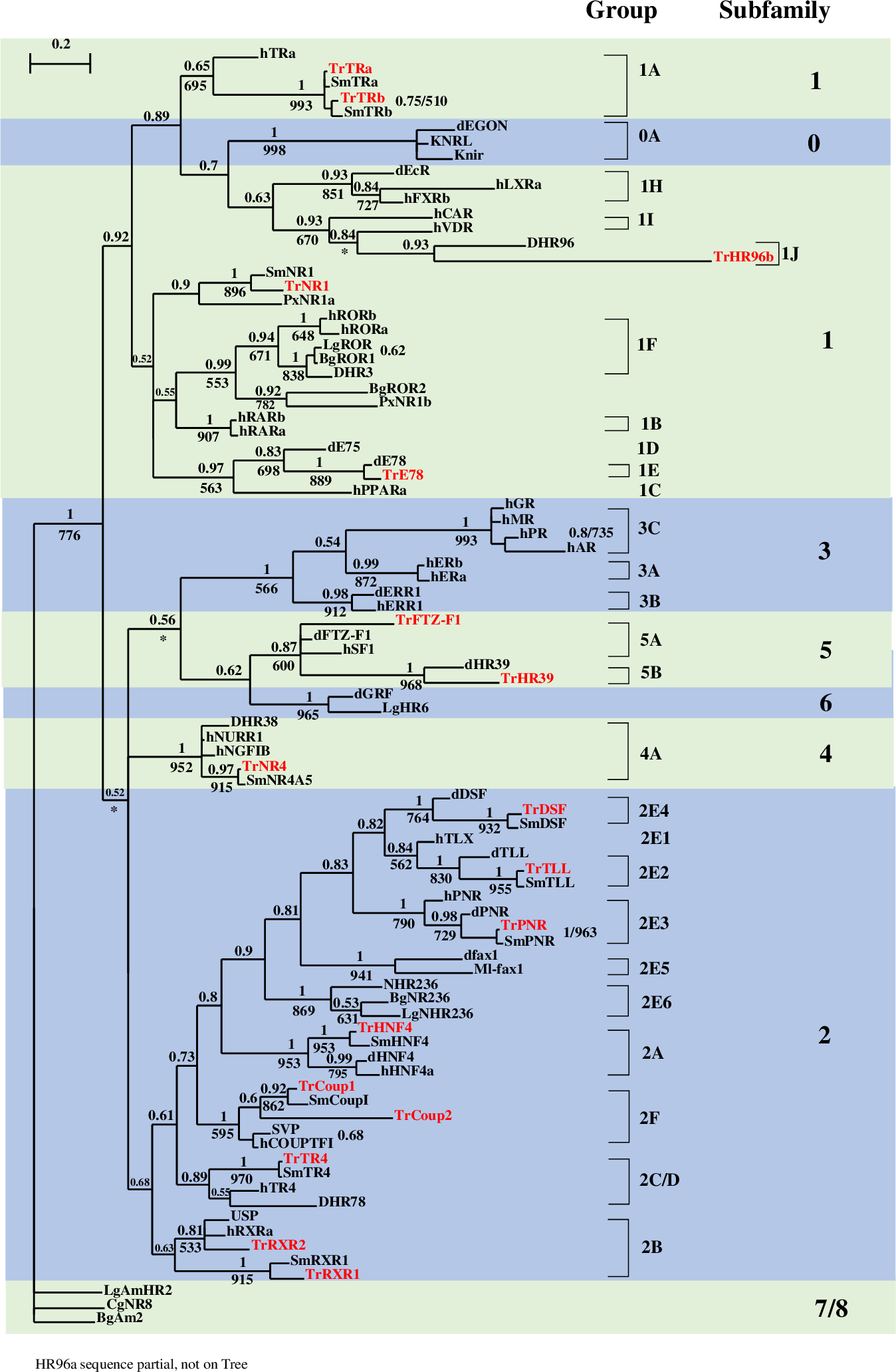

Supplement: S17 Fig — Methods for construction of phylogenetic trees see S1 Fig legend. ML model tested as LG+G+I (Equilibrium frequencies: Model, Proportion of invariable sites: Estimated (0.093), Number of substitution rate categories: 4, Gamma shape parameter: Estimated (0.709). Bg: Biomphalaria glabrata, Cg: Crassostrea gigas, d: Drosophila melanogaster, h: Homo sapiens, Lg: Lottia gigantean, Px: Protopolystoma xenopodis, Sm: Schistosoma mansoni, Tr: Trichobilharzia regent. Red highlighted NRs show T. regent NRs. (TIF) [file pone.0250750.s017.tif]

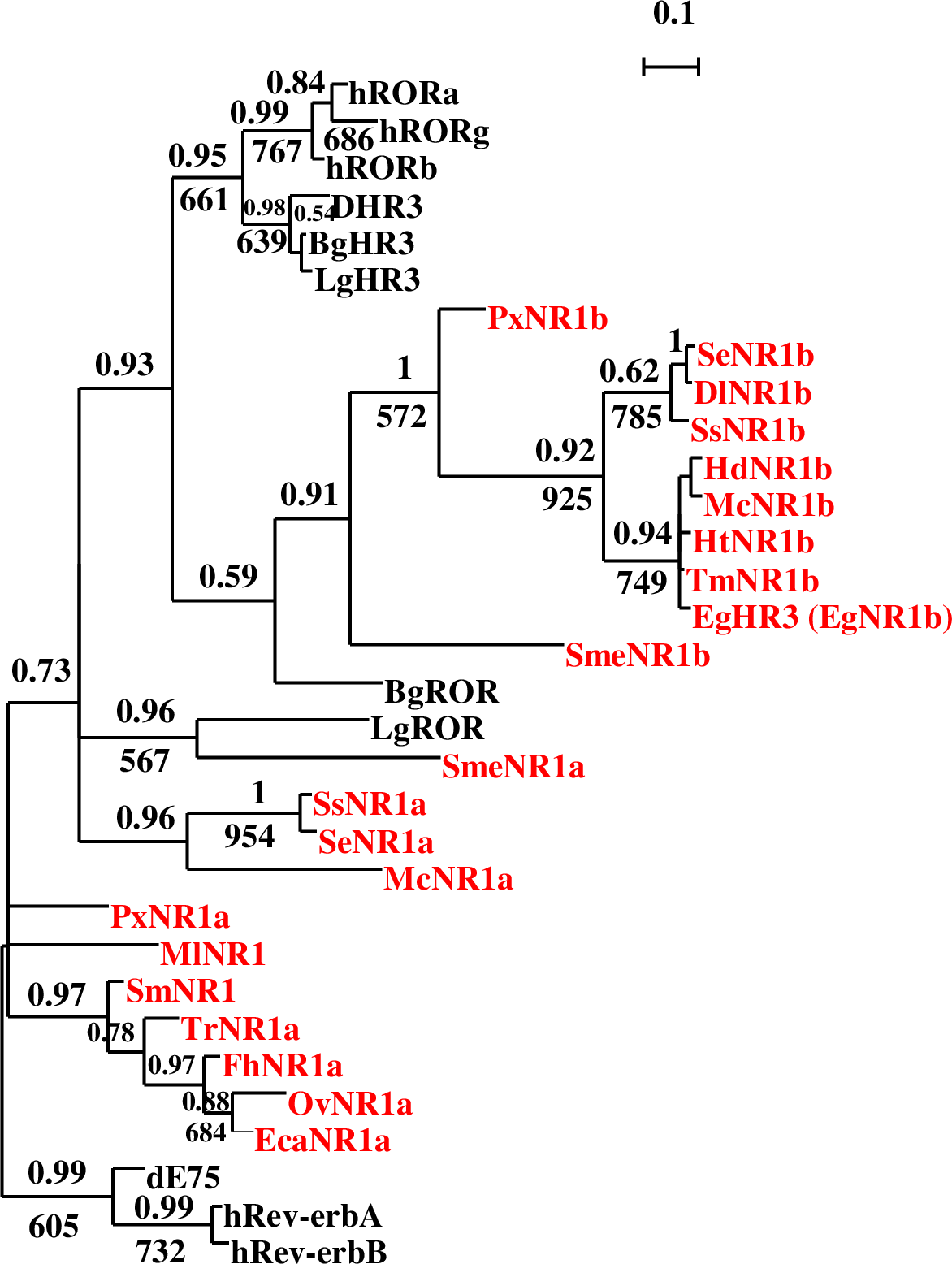

Supplement: S18 Fig — Methods for construction of phylogenetic trees see S1 Fig legend. ML model tested as JTT+G (Equilibrium frequencies: Model, Proportion of invariable sites: Fixed (0.0), Number of substitution rate categories: 4, Gamma shape parameter: Estimated (0.378). Bg: Biomphalaria glabrata, d: Drosophila melanogaster, Dl: Dibothriocephalus latus, Eca: Echinostoma caproni, Eg: Echinococcus granulosus, Fh: Fasciola hepatica, h: Homo sapiens, Hd: Hymenolepis diminuta, Ht: Hydatigera taeniaeformis, Lg: Lottia gigantean, Mc: Mesocestoides corti, Ml: Macrostomum lignano, Ov: Opisthorchis viverrini, Px: Protopolystoma xenopodis, Se: Spirometra erinaceieuropaei, Sm: Schistosoma mansoni, Sme: Schmidtea mediterranea, Ss: Schitocephalus solidus, Tm: Taenia multiceps, Tr: Trichobilharzia regent. Red highlighted NRs show Platyhelminths NRs. (TIF) [file pone.0250750.s018.tif]

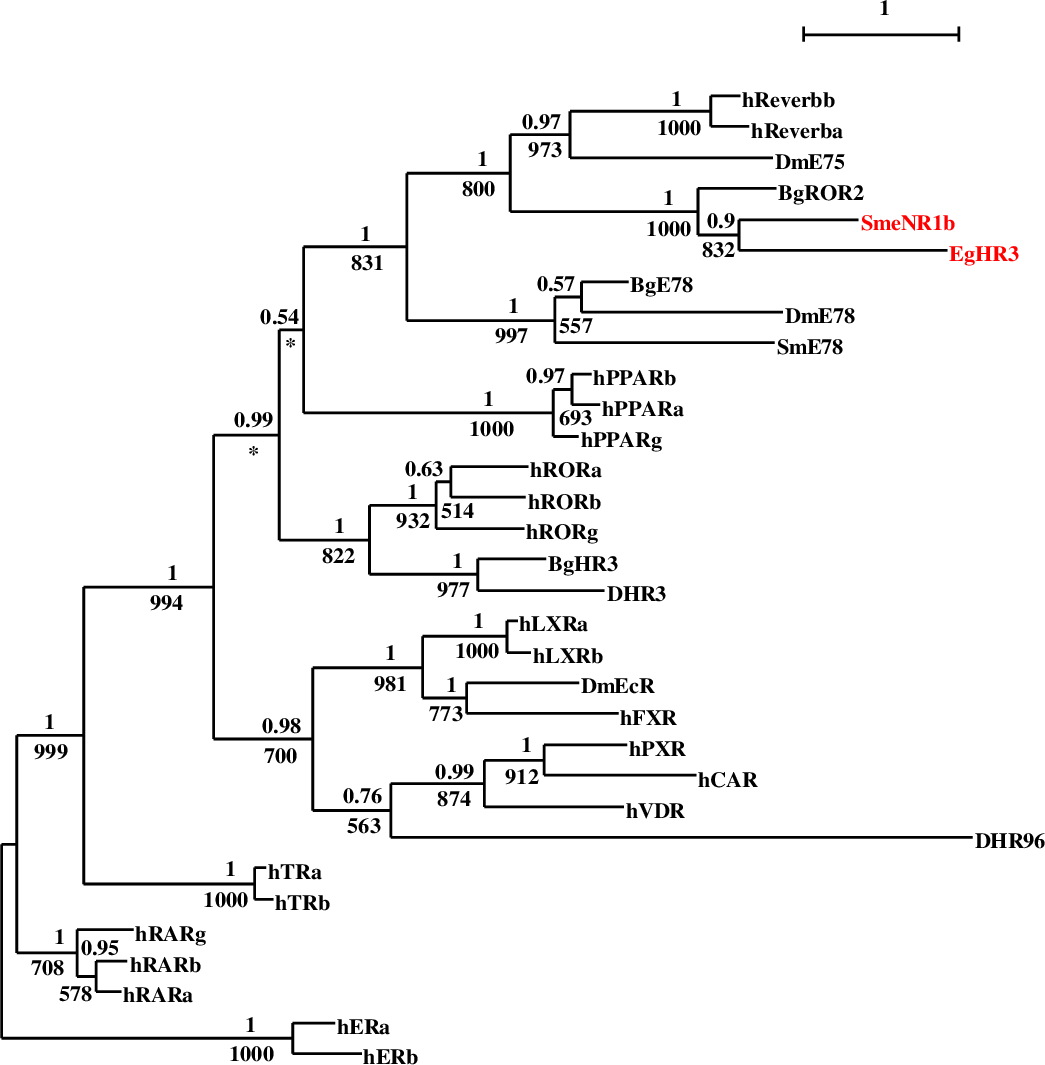

Supplement: S19 Fig — Methods for construction of phylogenetic trees see S1 Fig legend. ML model tested as LG+G+I+F (Equilibrium frequencies: Empirical, Proportion of invariable sites: Estimated (0.029), Number of substitution rate categories: 4, Gamma shape parameter: Estimated (1.348). Bg: Biomphalaria glabrata, d: Drosophila melanogaster, Eg: Echinococcus granulosus, h: Homo sapiens, Lg: Lottia gigantean, Sme: Schmidtea mediterranea. Red highlighted NRs show Platyhelminths NRs. (TIF) [file pone.0250750.s019.tif]

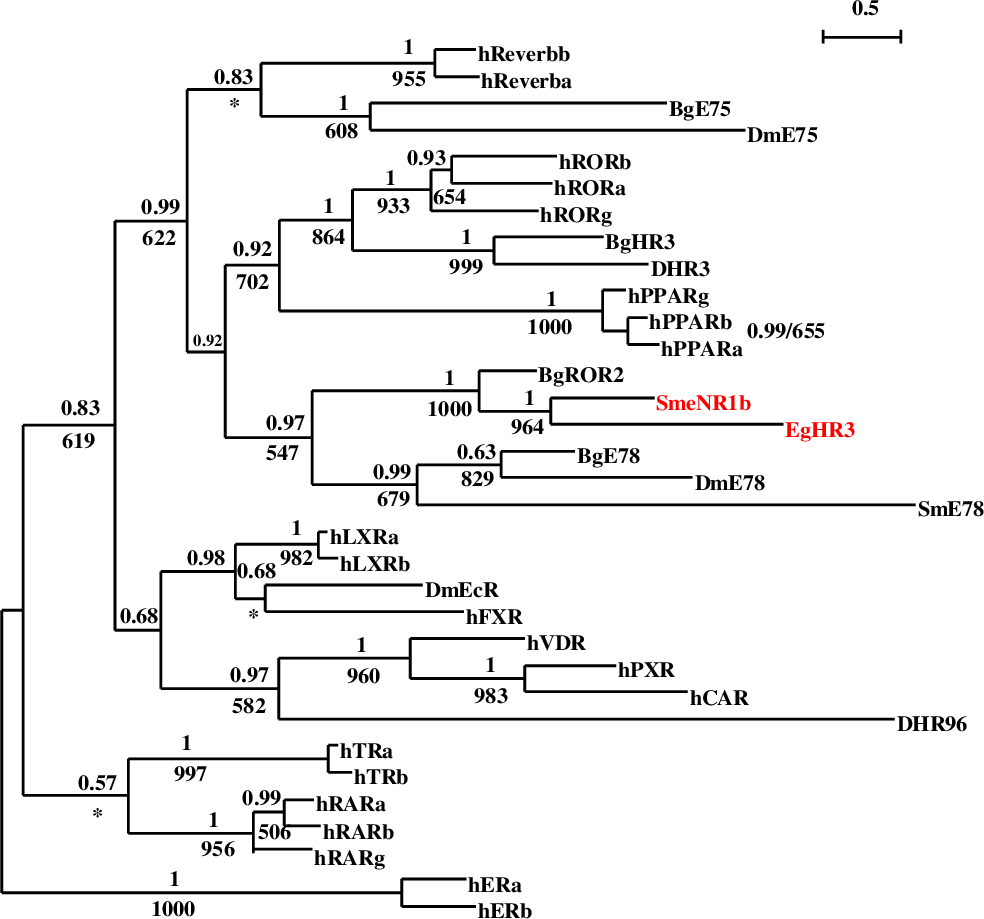

Supplement: S20 Fig — Methods for construction of phylogenetic trees see S1 Fig legend. ML model tested as LG+G+I+F (Equilibrium frequencies: Empirical, Proportion of invariable sites: Estimated (0.004), Number of substitution rate categories: 4, Gamma shape parameter: Estimated (1.741). Bg: Biomphalaria glabrata, d: Drosophila melanogaster, Eg: Echinococcus granulosus, h: Homo sapiens, Lg: Lottia gigantean, Sme: Schmidtea mediterranea. Red highlighted NRs show Platyhelminths NRs. (TIF) [file pone.0250750.s020.tif]

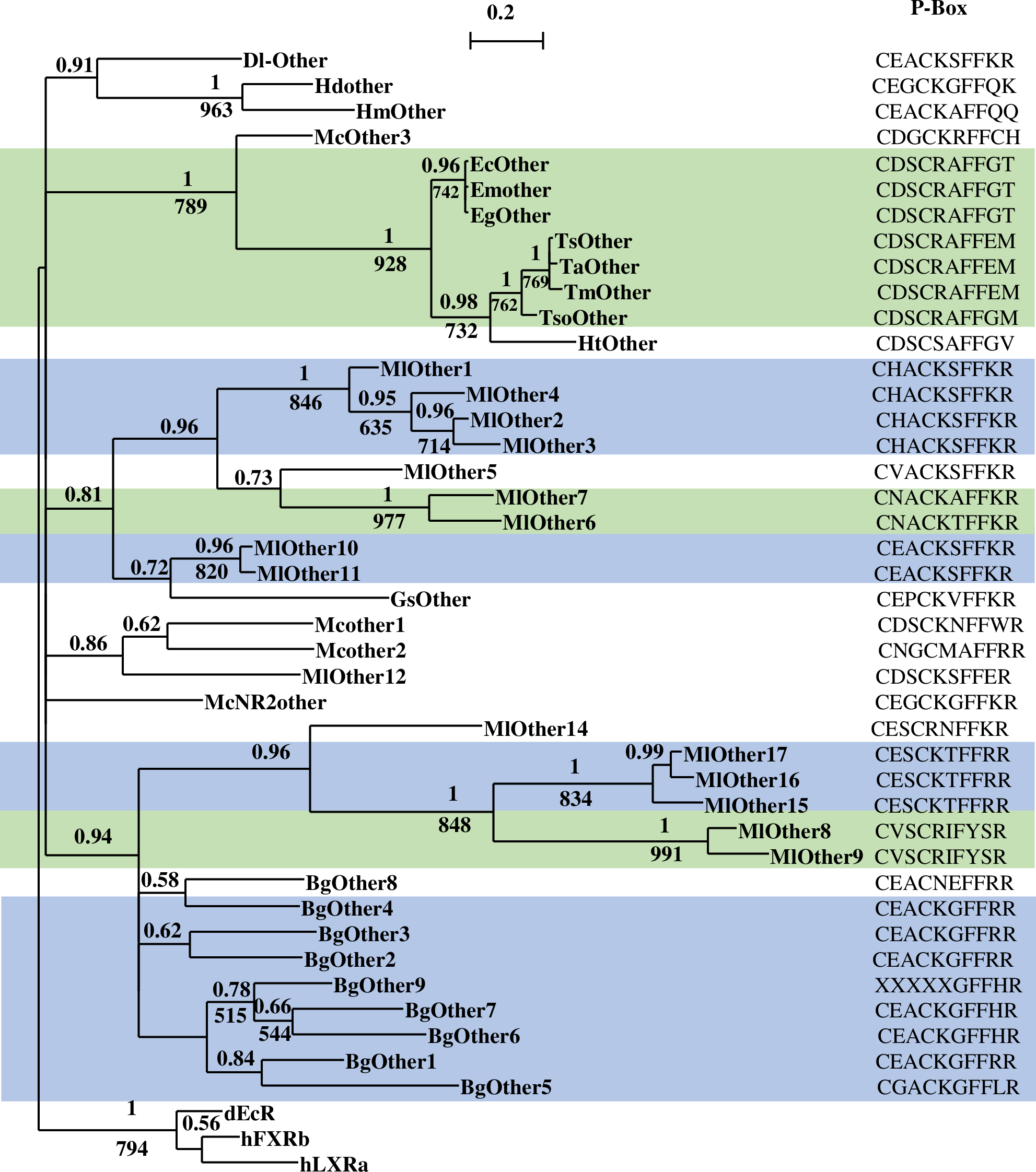

Supplement: S21 Fig — Methods for construction of phylogenetic trees see S1 Fig legend. ML model tested as JTT+G (Equilibrium frequencies: Model, Proportion of invariable sites: Fixed (0.0), Number of substitution rate categories: 4, Gamma shape parameter: Estimated (0.895). Bg: Biomphalaria glabrata, d: Drosophila melanogaster, Dl: Dibothriocephalus latus, Ec: Echinococcus Canadensis, Eg: Echinococcus granulosus, Em: Echinococcus multilocularis, Gs: Gyrodactylus salaris, h: Homo sapiens, Hd: Hymenolepis diminuta, Hm: Of H. microstoma, Ht: Hydatigera taeniaeformis, Mc: Mesocestoides corti, Ml: Macrostomum lignano, Sme: Schmidtea mediterranea, Ta: Taenia asiatica, Tm: Taenia multiceps, Ts: Taenia saginata, Tr: Trichobilharzia regent, Tso: Taenia solium. (TIF) [file pone.0250750.s021.tif]

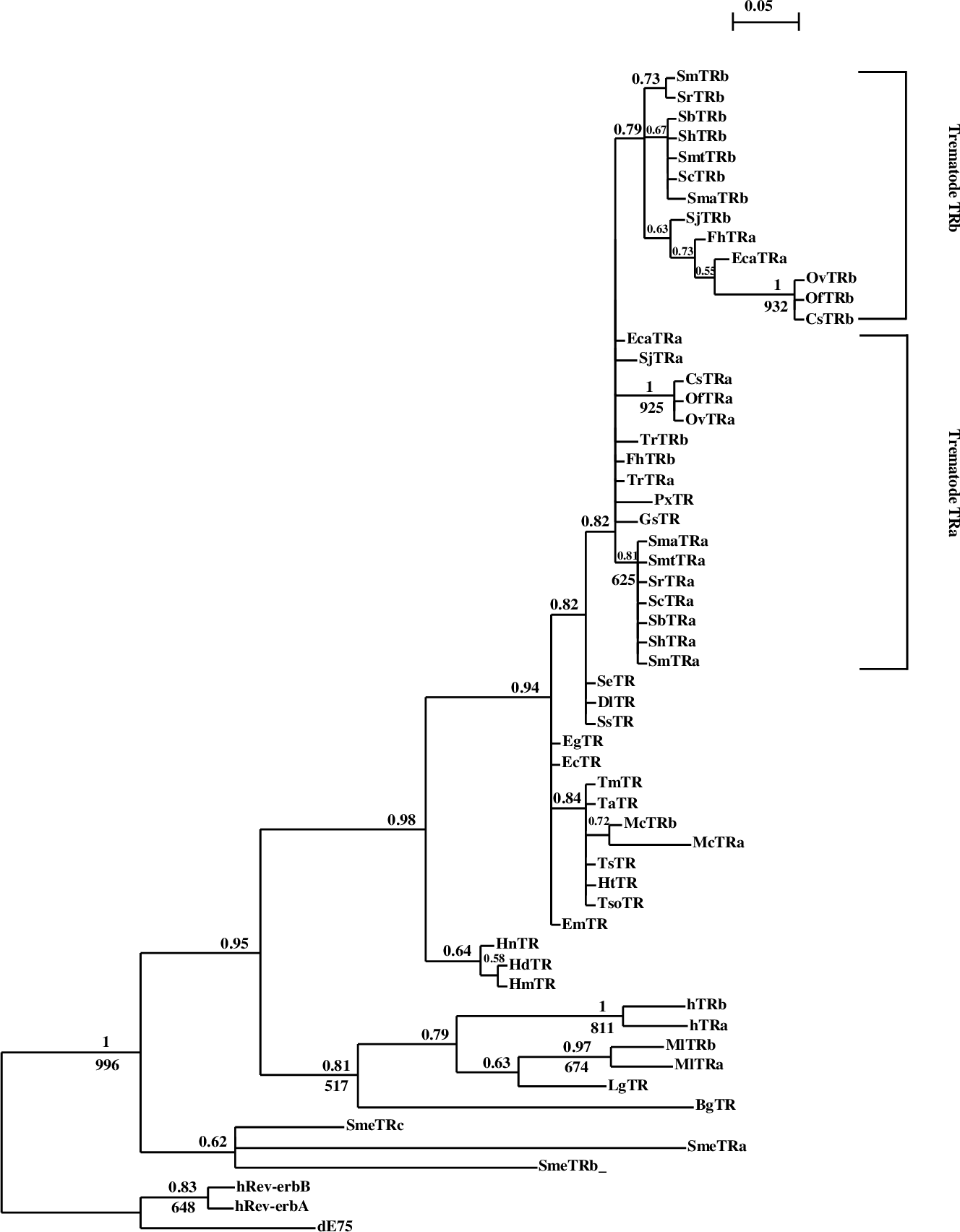

Supplement: S22 Fig — Methods for construction of phylogenetic trees see S1 Fig legend. ML model tested as LG+G (Equilibrium frequencies: Model, Proportion of invariable sites: Fixed (0.0), Number of substitution rate categories: 4, Gamma shape parameter: Estimated (0.411). Bg: Biomphalaria glabrata, Cs: Clonorchis sinensis, d: Drosophila melanogaster, Dl: Dibothriocephalus latus, Ec: Echinococcus Canadensis, Eca: Echinostoma caproni, Eg: Echinococcus granulosus, Em: Echinococcus multilocularis, Fh: Fasciola hepatica, Gs: Gyrodactylus salaris, h: Homo sapiens, Hd: Hymenolepis diminuta, Hm: Of H. microstoma, Hn: Hymenolepis nana, Ht: Hydatigera taeniaeformis, Lg: Lottia gigantean, Mc: Mesocestoides corti, Ml: Macrostomum lignano, Of: Opisthorchis felineus, Ov: Opisthorchis viverrini, Px: Protopolystoma xenopodis, Sb: Schistosoma bovis, Sc: Schistosoma curassoni, Se: Spirometra erinaceieuropaei, Sh: Schistosoma haematobium, Sj: Schistosoma japonicum, Sm: Schistosoma mansoni, Sma: Schistosoma margrebowiei, Smt: Schistosoma mattheei, Sme: Schmidtea mediterranea, Sr: Schistosoma rodhaini, Ss: Schitocephalus solidus, Ta: Taenia asiatica, Tm: Taenia multiceps, Ts: Taenia saginata, Tr: Trichobilharzia regent, Tso: Taenia solium. (TIF) [file pone.0250750.s022.tif]

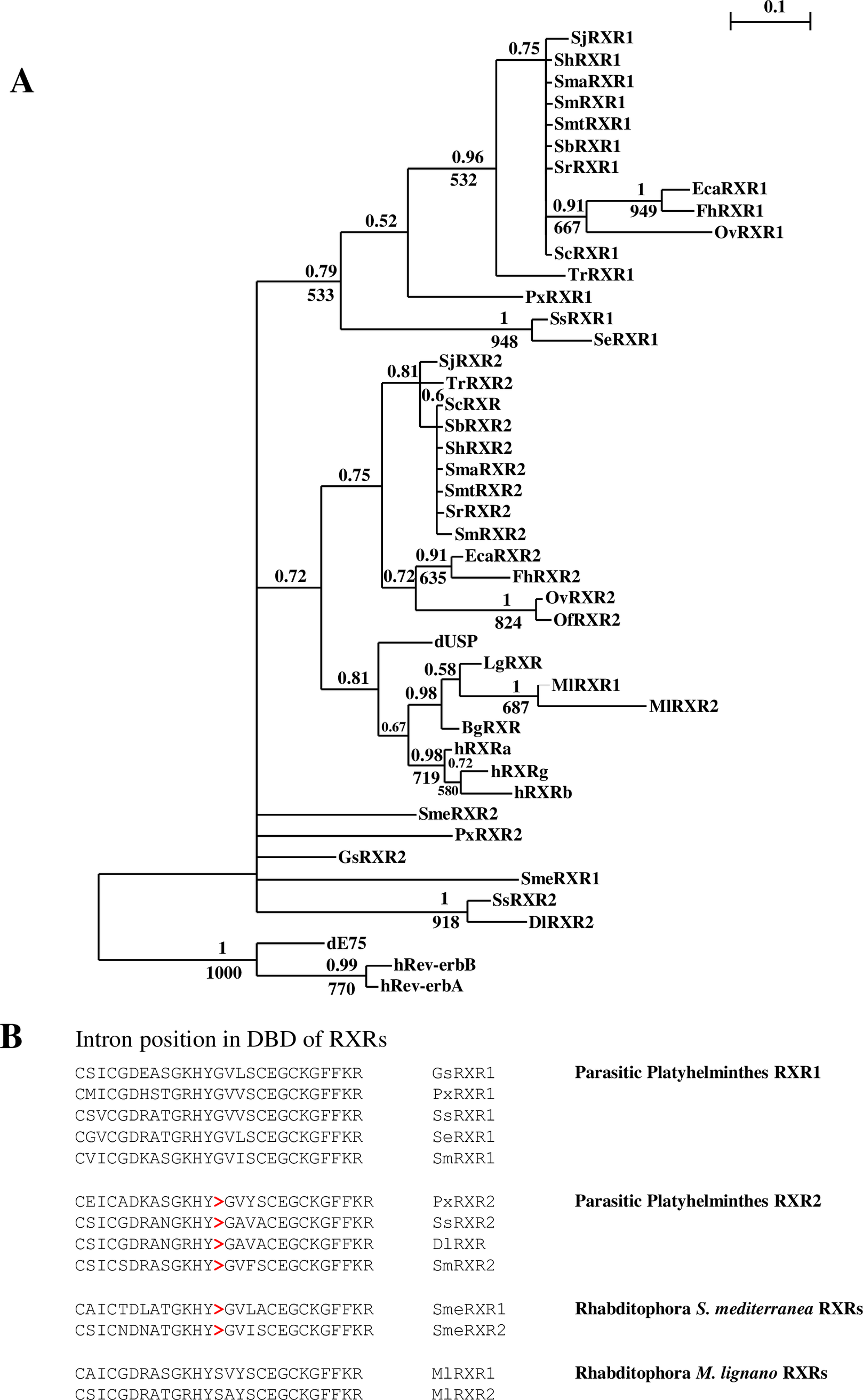

Supplement: S23 Fig — A) Bayesian phylogenetic tree of Platyhelminth RXR. Methods for construction of phylogenetic trees see S1 Fig legend. ML model tested as LG+G+I (Equilibrium frequencies: Model, Proportion of invariable sites: Estimated (0.339), Number of substitution rate categories: 4, Gamma shape parameter: Estimated (1.457). Bg: Biomphalaria glabrata, Cs: Clonorchis sinensis, d: Drosophila melanogaster, Dl: Dibothriocephalus latus, Ec: Echinococcus Canadensis, Eca: Echinostoma caproni, Eg: Echinococcus granulosus, Em: Echinococcus multilocularis, Fh: Fasciola hepatica, Gs: Gyrodactylus salaris, h: Homo sapiens, Lg: Lottia gigantean, Ml: Macrostomum lignano, Of: Opisthorchis felineus, Ov: Opisthorchis viverrini, Px: Protopolystoma xenopodis, Sb: Schistosoma bovis, Sc: Schistosoma curassoni, Se: Spirometra erinaceieuropaei, Sh: Schistosoma haematobium, Sj: Schistosoma japonicum, Sm: Schistosoma mansoni, Sma: Schistosoma margrebowiei, Smt: Schistosoma mattheei, Sme: Schmidtea mediterranea, Sr: Schistosoma rodhaini, Ss: Schitocephalus solidus, Tr: Trichobilharzia regent. B) Sequence alignment shows the intron position (red color >) in DBD of RXRs. (TIF) [file pone.0250750.s023.tif]

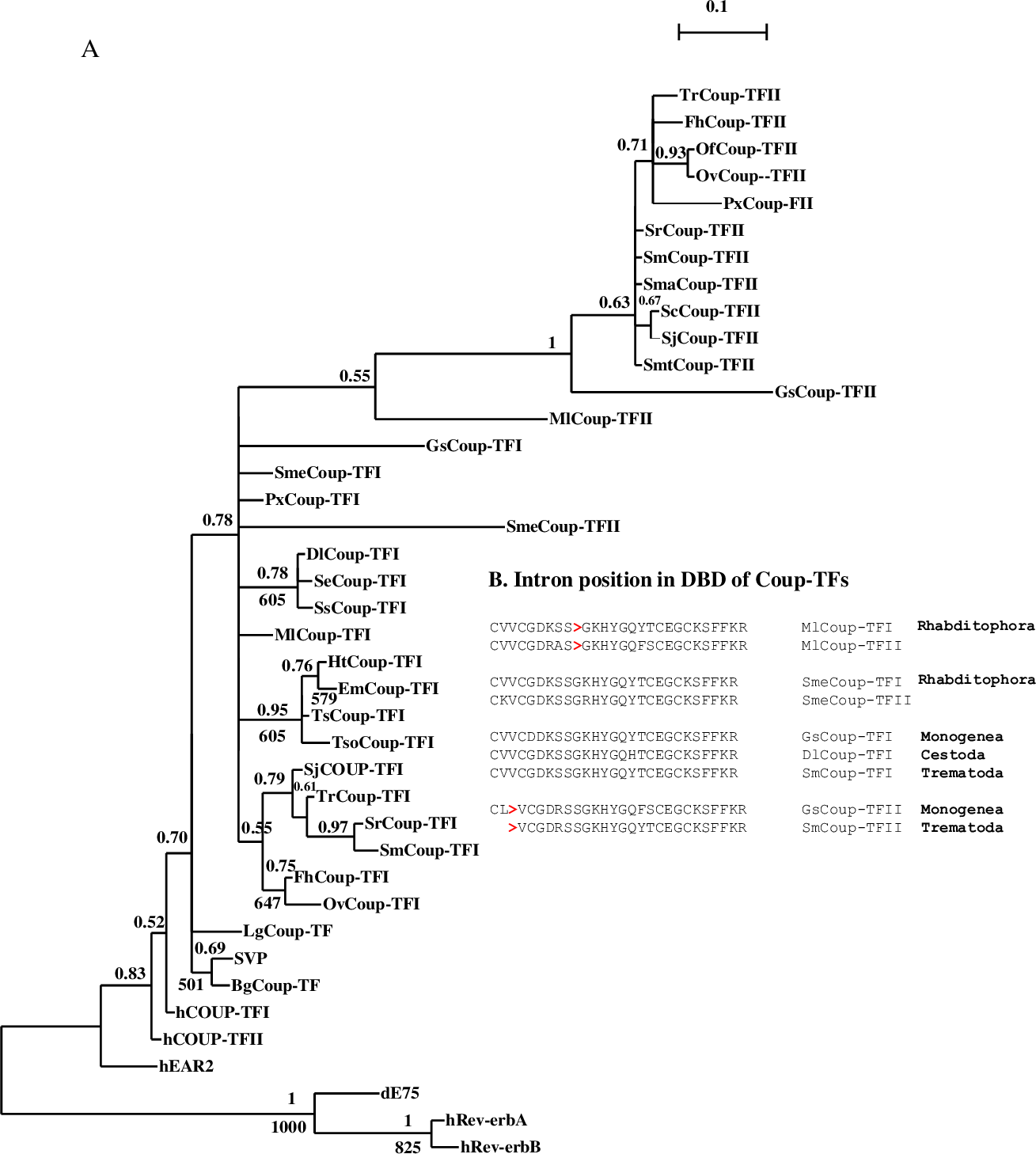

Supplement: S24 Fig — A) Bayesian phylogenetic tree of Platyhelminth Coup-TF. Methods for construction of phylogenetic trees see S1 Fig legend. ML model tested as FLU+G (Equilibrium frequencies: Model, Proportion of invariable sites: Fixed (0.0), Number of substitution rate categories: 4, Gamma shape parameter: Estimated (0.320). Bg: Biomphalaria glabrata, Cs: Clonorchis sinensis, d: Drosophila melanogaster, Dl: Dibothriocephalus latus, Ec: Echinococcus Canadensis, Eca: Echinostoma caproni, Eg: Echinococcus granulosus, Em: Echinococcus multilocularis, Fh: Fasciola hepatica, Gs: Gyrodactylus salaris, h: Homo sapiens, Lg: Lottia gigantean, Ml: Macrostomum lignano, Of: Opisthorchis felineus, Ov: Opisthorchis viverrini, Px: Protopolystoma xenopodis, Sb: Schistosoma bovis, Sc: Schistosoma curassoni, Se: Spirometra erinaceieuropaei, Sh: Schistosoma haematobium, Sj: Schistosoma japonicum, Sm: Schistosoma mansoni, Sma: Schistosoma margrebowiei, Smt: Schistosoma mattheei, Sme: Schmidtea mediterranea, Sr: Schistosoma rodhaini, Ss: Schitocephalus solidus, Ta: Taenia asiatica, Tm: Taenia multiceps, Ts: Taenia saginata, Tr: Trichobilharzia regent, Tso: Taenia solium. B) Sequence alignment shows the intron position (red color >) in DBD of Coup-TFs. (TIF) [file pone.0250750.s024.tif]

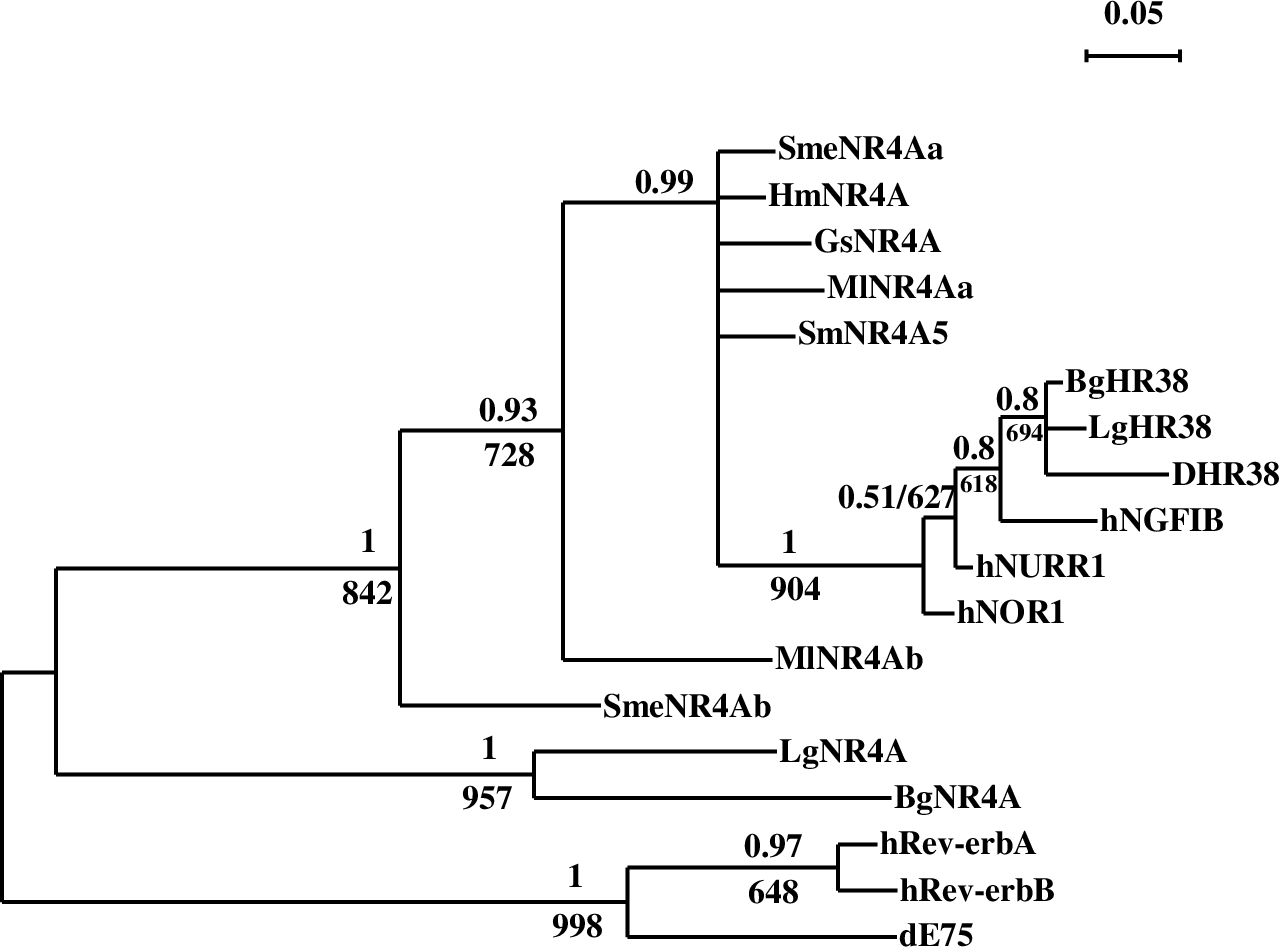

Supplement: S25 Fig — Methods for construction of phylogenetic trees see S1 Fig legend. ML model tested as FLU+G (Equilibrium frequencies: Model, Proportion of invariable sites: Fixed (0.0), Number of substitution rate categories: 4, Gamma shape parameter: Estimated (0.461). Bg: Biomphalaria glabrata, d: Drosophila melanogaster, Gs: Gyrodactylus salaris, h: Homo sapiens, Hd: Hymenolepis diminuta, Lg: Lottia gigantean, Mc: Mesocestoides corti, Ml: Macrostomum lignano, Sm: Schistosoma mansoni, Sme: Schmidtea mediterranea. (TIF) [file pone.0250750.s025.tif]

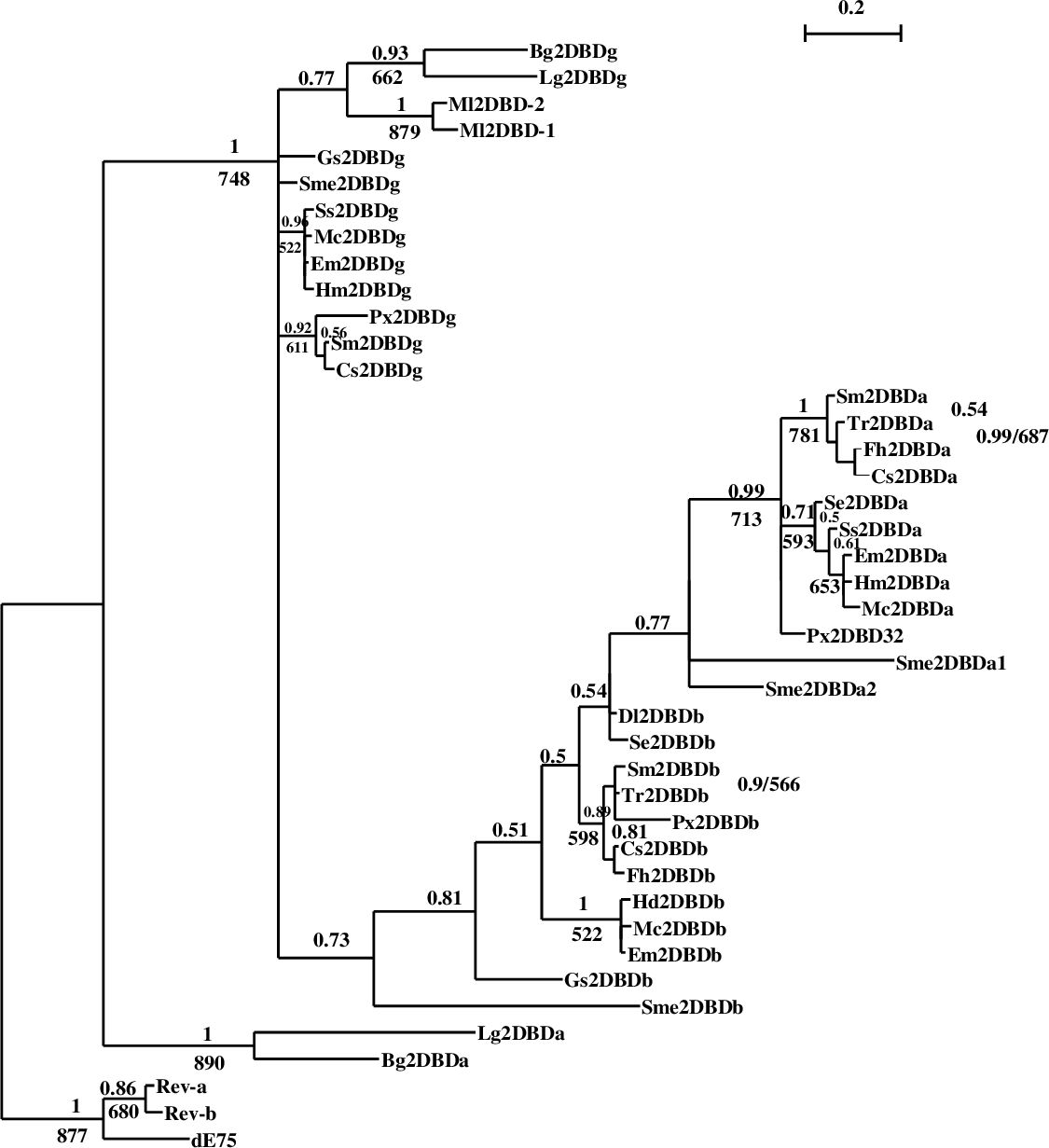

Supplement: S26 Fig — Methods for construction of phylogenetic trees see S1 Fig legend. ML model tested as JTT+G+I (Equilibrium frequencies: Model, Proportion of invariable sites: Estimated (0.131), Number of substitution rate categories: 4, Gamma shape parameter: Estimated (0.832). Bg: Biomphalaria glabrata, Cs: Clonorchis sinensis, d: Drosophila melanogaster, Dl: Dibothriocephalus latus, Em: Echinococcus multilocularis, Fh: Fasciola hepatica, Gs: Gyrodactylus salaris, h: Homo sapiens, Hd: Hymenolepis diminuta, Hm: H. microstoma, Lg: Lottia gigantean, Mc: Mesocestoides corti, Ml: Macrostomum lignano, Px: Protopolystoma xenopodis, Se: Spirometra erinaceieuropaei, Sm: Schistosoma mansoni, Sme: Schmidtea mediterranea, Ss: Schitocephalus solidus, Tr: Trichobilharzia regent. (TIF) [file pone.0250750.s026.tif]
