# Supplementary material for: Identification and evolution of nuclear receptors in Platyhelminths
Source: PLoS One. 2021 Aug 13;16(8):e0250750. doi: 10.1371/journal.pone.0250750 (PMC8363021; doi:10.1371/journal.pone.0250750)
Supplement: S1 File — (DOCX) [file pone.0250750.s027.docx]

**S1 File:** **lists of GenBank accession number of published NR sequences used in this study**

***Drosophila melanogaster***

Knir: X1333

KNRL: X14153

EGON: X16631

EcR: M74078

DHR96: U36792

DHR3: M90806

E75: X51548

HR38: X89246

ERR: NP_729340.1

FTZ-F1: M98393

HR39: L06423

GRF: NP_001259157.1

SVP: M28863

HNF4: U70847

DSF: AF106677

fax1: NM_141390

PNR: NP_611032.2

TLL: M34639

DHR78: U36791

***Homo sapiens***

hPNR: AF121129

Human TRa: X55005

hTRb: X04707

hRARa: X06614

hRARb: X07282

hPPARa: L07592

hRev-erb-a: M24898

hRev-erb-b: L31785

hRORa: U04897

hRORb: Y08639

hLXRa: U22662

hFXR: U68233

hVDR: J03258

hCAR: Z30425

hHNF4a: X76930

hRXRa: X52773

hTR4: U10990

hTLX: Y13276

hPNR: AF121129

hCOUP-TFI: X16155

hERa: X03635

hERb: AB006590

hERRa: X51416

hGR: X03225

hMR: M16801

hPR: X51730

hAR: M20132

hNGFIB: D49728

hNURR1: X75918

hSF1: U76388

***Schistosoma mansoni***

SmNR1: AH013463.2

SmNR4A5: AY688260.1

Sm2DBDα: AY395036

Sm2DBDβ: AY688251

Sm2DBDγ: AY698061

SmDSF: AY688254

SmPNR: AH013463.2

SmHR96a: AY688258

SmHR96-b:AY688259

SmRXR1: AF094759

SmTLL: AY698060

SmTRα: AY395038

SmTRβ: AY395039

SmCoup-TFI: AAW88535.1

SmHNF4: AY688257.1

SmTR4: AY688263.1

LgROR: XP_009062806.1

BgROR1: XP_013094161.1

BgROR2: XP_013077761.1

LgHR6: XP_009056562.1

***Biomphalaria glabrata***

BgNR236: XP_013088107.1

BgNR4A: XP_013085240.1

BgTR: S1 file of: The Nuclear Receptors of Biomphalaria glabrata and Lottia gigantea: Implications for Developing New Model Organisms. Kaur S, et al. PLoS One, 2015 Apr 7;10(4):e0121259. doi: 10.1371/journal.pone.0121259.

***Lottia gigantea***

LgAMHR2: XP_009055459.1

LgNHR236: XM_009058292.1

LgTR, LgNR4A: S1 file of: The Nuclear Receptors of Biomphalaria glabrata and Lottia gigantea: Implications for Developing New Model Organisms. Kaur S, et al. PLoS One, 2015 Apr 7;10(4):e0121259. doi: 10.1371/journal.pone.0121259.

***Crassostrea gigas***

CgNR8: AKG49571.1

***Caenorhabditis elegans***

NHR236: NM_067789
