# Supplementary material for: Identification and evolution of nuclear receptors in Platyhelminths
Source: PLoS One. 2021 Aug 13;16(8):e0250750. doi: 10.1371/journal.pone.0250750 (PMC8363021; doi:10.1371/journal.pone.0250750)
Supplement: S2 File — (DOCX) [file pone.0250750.s028.docx]

**S2 File. DBD sequences of NRs in 33 species of Platyhelminthes**

**1. NRs in *Clonorchis sinensis* (22)(PRJDA72781)**

**>CsTRa** (GenBank_GAA56779)

CVVCGDSATGFHYRAMTCEGCKGFFRRSVQKKLVYTCKFNGRCSVADKQNRNSCQKCRFDRCLSGGM

**>CsTRb** (GenBank_RJW66780.1)

CVVCGDNATGFHYRAMTCEGCKGFFRRSIQKQLVYVCKFQGNCSVSDKHSRNSCQKCRFDRCIRGGM

**>CsE78** (GenBank_RJW61214)

CKVCGDKASGYHYGVISCEGCKGFFRRSIQKQIEYKCLRDGKCLVIRLNRNRCQYCRFRKCIAVGM

>**CsHR96a** (GenBank_RJW63637)

CIVCGEPASGYNFDRLTCESCKAFFRRNALKPKDKIKVCSRGGGCIIEGNQRKHCPSCRLEKCFAVGM

**>CsHR96b** (GenBank_RJW67200.1)

CKVCGDRAVNHNFGQLTCESCKAFFRRNAHKLLPSLLDRTFECRSDVDMTTVQYSLSLSRSKYLVELTCTSKTGEHEITPSTRRECPACRLKKCFLVGM

**>CsNHR96c** (GenBank_RJW67200.1)

CNVCGDAAMGFNFGAVTCESCKAFFRRTARKAQVANCVFNEKCTITVATRRFCSHCRLKKCFAAGM

**>CsNR1a** (GenBank_GAA58146)

CVVCGDKSTGAHYGVFTCEGCKGFFRRAVHRNQTYACARNGSCEVNRALRNKCQHCRFLKCLASGM

**>CsHNF4** (GenBank_GAA47660)

CLICGDRATGKHYGAYSCDGCKGFFRRSVRRNHSYTCRHKRACVITKDKRNQCRFCRLRKCFRVGM

**>CsRXR1** (GenBank_GAA56711)

CVICGDRASGRHYGVVSCEGCKGFFKRTVRKQVQYVCRGSGDCPVDRRKRTRCQACRYDRCILKGM

**>CsRXR2** (GenBank_ RJW63494)

CQVCGDRACGKHYGVFSCEGCKGFFRRTVRRELVYTCRDSQSCQIDRRLRNRCQFCRYHKCLQVGM

**>CsTR4** (GenBank_GAA51438.1)

CRVCGDKASGRHYGVVSCEGCKGFFKRSIRGHVSYVCRSDQNCLVNKAYRNRCQYCRLQKCLLVGM

**>CsTLL** (GenBank_GAA56686)

CRVCKDHSSGKHYGIYACDGCAGFFKRSIRRNRQYACKNRTANGTKLSTAVGGCRVDKSHRNQCRACRLKKCLEVGM

**>CsPNR** (GenBank_GAA35793)

CSVCGDVSSGKHYGILACNGCSGFFKRSVRRKLIYRCQAGTGLCSIDKAHRNQCQACRLKKCLRMGM

**>CsDSF** (WBPS_Clonorchis_sinensis_scf00005)

CGVCGDISSGKHYGIYSCDGCSGFFKRSIHKSRTYTCKAVGHLKGRCPIDRNRRNQCRACRLKKCFQAQM

**>CsCoup-TFI** (GenBank_GAA52025)

CVVCGDKSSGKHYGQHTCEGCKSFFKRSVRRKLTYTCRGTRQCPIDVHHRNQCQYCRFQKCVRAGM

**>CsCoup-TFII** (GenBank_RJW72774)

CG>VCGDRSSGKHYGQFTCEGCKSFFKRSVRKSASYVCRSEGQCPVDAQRRNQCQACRMTRCLLAGM

**>CsNR4A** (GenBank_GAA40423)

CLVCGDSAACQHYGVRTCEGCKGFFKRTIQKNAQYVCLQSKNCVVDKRRRNRCQYCRFQKCLKVGM

**>CsFTZ-F1a** (GenBank_GAA50141.1)

CPICGDRVSGYHYGLPTCESCKGFFKRTVQNKKEYHCTEQGNCVIDRIHRKRCAHCRFQKCLAVGM

**>CsFTZ-F1b** (GenBank_GAA50141.1)

CPICGDKISGYHYGIFCCESCKGFFKRTVQNAKRYACHHPSANSVCEINVSSRKKCPACRFLKCVDKGM

**>Cs2DBDa** (GenBank_GAA27896)

CQVCGELAAGFHHGAYVCEACKKFFMRHSLTDTKPTNICPTGGNCVVAKGSRGKCQICRYRKCLFVGM

CRVCGGRSSGFHFGALTCEGCKGFFRRTEGSAASLVCVGGQNTCTITPRSRNACKSCRFRRCIAAGM

**>Cs2DBDb** (GenBank_GAA48469)

CQICGQPAVGFHHRAYVCEACKKFFMRHTAARYRHAEAGGGNLSDSACPMGGQCRVEGPGRGKCPHCRYRKCLDLGM

CRVCGGPSSGFHFGALTCEGCKGFFRRTVLSNVRLECLSNNDCPITPANRNMCKSCRFQRCLAVGM

**>Cs2DBDg** (scf00241_C_sinensis)

CDICGDVSAGFHCNAYVCEACKKFFIRSSKGDNFSKYTCTKSNACEINKDTRTHCQRCRYQKCLRLGM

CRVCGAKSSGFHFGAITCEGCKGFFRRTINERESQRYTCRNGGNCAVTGATRNNCKSCRYRRCVAVGM

**2. NRs in *Dibothriocephalus latus* (19)(PRJEB1206)**

**>DlTR** (GenBank_VDK41693.1)

CVVCGDNATGFHYRAMTCEGCKGFFRRSVQKKLEYTCKFNGRCSVADKQNRNSCQKCRFDRCIKGGM

**>DlE78** (scaffold:D_latum_Geneva_v1_0_4:DILT_scaffold0034689)

CKVCGDKASGYHYGVVSCEGCKGFFRRSIQKQIEYKCLRDGKCIVIRLNRNRCQYCRFRKCIAVGM

**>DlHRHR96a** (scaffold:D_latum_Geneva_v1_0_4:DILT_scaffold0007202)

FRRNALKPRDK>IKACGRSGDCNIEGSQRKHCPSCRLEKCLAVGM

**>DlHR96b** (scaffold:D_latum_Geneva_v1_0_4:DILT_scaffold0069985)

ELTCTSKTGEHVVSPTTRRECPACRLKRCFLIGM

**>DlHR96c** (scaffold:D_latum_Geneva_v1_0_4:DILT_caffold0021976, scaffold0016777)

CAVCGDHAVGFNFGAIACESCKAFFxxxxxxxMPSCLFSGKCSIQVKTRRFCSPCRLEKCFAVGM

**>DlNR1b** (scaffold:D_latum_Geneva_v1_0_4:DILT_scaffold0004747, scaffold0002345)

CLVCGDTASGVHYGVLSCEGCKGFFRRTLQDKRSQPKQCLRDGLCRITVKSRNNCQPCRLRRCLELGM

**>DlRXR2** (GenBank_VDN14946.1)

CSICGDRANGRHYGAVACEGCKGFFKRSIRKNIKYVCREGENCQMSIRQRNRCQHCRFRRCLLEGM

**>DlCoup-TFI** (scaffold:D_latum_Geneva_v1_0_4:DILT_scaffold0074492)

CVVCGDKSSGKHYGQHTCEGCKSFFKRSVRRKLIYKCRGNRQCPVDMHHRNQCQHCRFQKCLKTGM

**>DlHNF4** (scaffold:D_latum_Geneva_v1_0_4:DILT_scaffold0031848)

GKHYGAYSCDGCKGFFRRSVRRKHTYSCRYNRACVIDKDMRNQCRFCRLKKCFRVGM

**>DlTR4** (scaffold:D_latum_Geneva_v1_0_4:DILT_contig0013139)

RHYGVLSCEGCKGFFKRSIRGHVNYICRTKQRC

**>DlPNR** (scaffold:D_latum_Geneva_v1_0_4:DILT_scaffold0024809)

DKERRNQCQACRLRKCIQMGM

**>DlTLL** (scaffold:D_latum_Geneva_v1_0_4:DILT_contig0001642)

RSIRRNRQYSCKSRNGTEASCRIDKPHRNQCRACRLKKCLDAGM

**>DlNR4A** (scaffold:D_latum_Geneva_v1_0_4:DILT_contig0001883)

RTIQKNAHYVCLQSKNCVVDKRRRNRCQYCRFQKCLKVGM

**>DlFTZ-F1a** (scaffold:D_latum_Geneva_v1_0_4:DILT_scaffold0024754)

CPVCGDKVSGYHYGLPTCESCKGFFKRTVQNKKEYHCTDTGACTIDRVHRKRCAHCRFQKCLSVGM

**>DlFTZ-F1b (DHR39)** (scaffold:D_latum_Geneva_v1_0_4:DILT_scaffold0055138)

CPICGDKISGYHYGIFCCESCKGFFKRTVQNAKRYACHMATPSSLCEITAASRKKCPACRFVKCLEKGM

**>Dl2DBDa** (scaffold0008272, scaffold0009459, scaffold0014679)

CQICGELAAGFHHGAYVCEACKKFFMRQSLSNGKSTTVCPSGGNCVIAKTSRGRCQQCRYKKCLELGM

CRVCGGRSSGFHFGALTCEGCK

**>Dl2DBDb** (scaffold:D_latum_Geneva_v1_0_4:DILT_scaffold0006747, scaffold0007154)

CQVCGQPSVGFHHRAYVCEACKKFFTRHMTMRVRKNKEGAENPEAFAGRELRTVCPMGGRCKIEGAGRGKCPHCRFRKCLDLGM

CRVCGGRSSGFHFGALTCEGCKGFFRRTALSNVKLECLGKHNCCITPANRNMCKSCRFQRCLAVGM

**>Dl2DBDg** (scaffold:D_latum_Geneva_v1_0_4:DILT_scaffold0031486, scaffold0012192, scaffold0033872)

CDICGDVSAGFHCNAYVCEACKKFFIRSSKNENYAKYSCSKVNSCEVNKDTRTHCQRCRYQKCL

CRVCGAQSSGFHFGAITCEGCKGFFRRTISERDNQRYTCRNGGACLITLATRNNCKSCRYRKCLSVGM

**>Dl-other_Coup** (scaffold:D_latum_Geneva_v1_0_4:DILT_scaffold0026769)

VCGDESRGRHYGQYTCEACKSFFKRSVRKGAAYICLRGGACDIDARLRNRCRYCRWKRCLAAGM

**3. NRs in *Echinococcus Canadensis* (17)(** **prjeb8992)**

**>EcTRa** (E.canG7_contigs_6644)

CVVCGDNATGFHYRAMTCEGCKGFFRRSVQKKLEYTCKFNGNCSVGDKQNRNSCQKCRFDRCIKGGM

**>EcE78** (E.canG7_contigs_1334)

CHVCGDKASGYHYGVISCEGCKGFFRRSIQKQIEYKCLREGKCTVVRLNRNRCQYCRFRKCIAVGM

**>EcHR96a** (E.canG7_contigs_5001)

CVVCGEPASGYNFDRLTCESCKAFFRRNALKPKEKIKACGRNGDCNIEGSQRKHCPSCRLEKCLAVGM

**>EcHR96b** (E.canG7_contigs_5605)

CRVCGDRAVNHNFGQLTCESCKAFFRRNAHKALPSISAALQSSDCATTTDAYLQALQRGLRDLTCTSKSGEHVVSPSTRRECPACRLKRCFLIGM

**>EcHR96c** (E.canG7_contigs_8397)

CAVCGDHAVGFNFGAIACESCKAFFRRNALRATTPPCLFNGSCLIQVKTRRFCSPCRLSKCFAVGM

**>EcNR1b** (E.canG7_contigs_6225)

CLVCGDTASGIHYGVLSCEGCKGFFRRALQDKRAHLRKCIRQDRCKISIKTRNHCQPCRLRRCLELGM

**>EcHNF4** (E.canG7_contigs_8260)

CLICGDKATGKHYGAYSCDGCKGFFRRSVRRKHTYSCRYNRTCTIDKDMRNQCRFCRLKKCFRVGM

**>EcTR4** (E.canG7_contigs_6029)

CECLVCGDVASGRHYGVISCEGCKGFFKRSIRGHVNYVCRSNKHCIVNKAFRNRCQYCRMQKCLLVGM

**>EcTLL** (E.canG7_contigs_1025)

CKVCKDHSSGKHYGIYACDGCAGFFKRSIRRNRQYTCKSRNGQESCCRVDKPHRNQCRACRLKKCLEAGM

**>EcCoup-TFI** (E.canG7_contigs_4826)

CVVCGDKSSGKHYGQFTCEGCKSFFKRSVRRQLTYTCRGARQCLIDLHHRNQCQYCRFQKCLRKGM

**>EcNR4A** (E.canG7_contigs_2489)

CLVCGDNAACQHYGVRTCEGCKGFFKRTIQKNAHYVCLQTKNCIVDKRRRNRCQYCRFQKCLKVGM

**>EcFTZ-F1a** (E.canG7_contigs_1421)

CPVCGDKVSGYHYGLQTCESCKGFFKRTVQNKKAYQCTEAGVCIIDRVHRKRCAYCRFKKCLSAGM

**>EcFTZ-F1b** (E.canG7_contigs_3694)

CPICGDKISGYHYGIFCCESCKGFFKRTVQNSKRYACHMATPTSLCDITATSRKKCPACRFVKCLEKGM

**>Ec2DBDa** (E.canG7_contigs_7094)

CQICGELAAGFHHGAYVCEACKKFFMRHSLANGRSTTTCPSGGNCVIAKSSRGRCQQCRYKKCLEVGM

CRVCGGRSSGFHFGALTCEGCKFFRRTEESASRLVCVGGKDNCTITPRSRNVCKACRFRRCLRAGM

**>Ec2DBDb** (E.canG7_contigs_9304)

CQVCGQPSVGFHHRAYVCEACKKFFTRHLTNRIKKEKSTFGEGDSAKSSIQGGETHPDLNVVCPMGGNCKIEGPGRGKCPHCRFRKCLDLGM

CRVCGGPSSGFHFGALTCEGCKGFFRRTVHSNSIPQCLGNQTCRITPSNRNMCKSCRFKKCLEVGM

**>Ec2DBDg** (E.canG7_contigs_5288)

CDICGDVSAGFHCNAYVCEACKKFFIRSSKNENYAKYSCSKVNACEVNKDTRTHCQRCRFYKCLRIGM

CRVCGAQSSGFHFGAITCEGCKGFFRRTISERDNQRYTCRNGGTCLITLATRNNCKSCRYRKCLSVGM

**>EcOther** (E.canG7_contigs_2408)

CAICGATATGKHYGALSCDSCRAFFGTATHLGLRECSGKSNIHDNNHQLRCHSCRLRKCLAVGM

**4. NRs in *Echinococcus granulosus* (17)(** **prjna182977)**

**>EgTR** (EGR_08053.1 scaffold:ASM52419v1)

CVVCGDNATGFHYRAMTCEGCKGFFRRSVQKKLEYTCKFNGNCSVGDKQNRNSCQKCRFDRCIKGGM

**>EgE78** (EGR_08053.1 scaffold:ASM52419v1)

CHVCGDKASGYHYGVISCEGCKGFFRRSIQKQIEYKCLREGKCTVVRLNRNRCQYCRFRKCIAVGM

**>EgHR96a** (EGR_02758.1 scaffold:ASM52419v1)

CVVCGEPASGYNFDRLTCESCKAFFRRNALKPKEKIKACGRNGDCNIEGSQRKHCPSCRLEKCLAVGM

**>EgHR96b** (EgG_scaffold_0002)

CRVCGDRAVNHNFGQLTCESCKAFFRRNAHKALPSISAALQSSDCATTTDAYLQALQRGLRDLTCTSKSGEHVVSPSTRRECPACRLKRCFLIGM

**>EgHR96c** (EGR_08428.1 scaffold:ASM52419v1)

CAVCGDHAVGFNFGAIACESCKAFFRRNALRATMPPCLFNGSCLIQVKTRRFCSPCRLSKCFAVGM

**>EgNR1b** (EGR_04405.1 scaffold:ASM52419v1)

CLVCGDTASGIHYGVLSCEGCKGFFRRALQDKRAHLRKCIRQDRCKISIKTRNHCQPCRLRRCLELGM

**>EgHNF4** (EGR_06875.1 scaffold:ASM52419v1)

CLICGDKATGKHYGAYSCDGCKGFFRRSVRRKHTYSCRYNRTCTIDKDMRNQCRFCRLKKCFRVGM

**>EgCoup-TFI** (EGR_00119.1 scaffold:ASM52419v1)

CVVCGDKSSGKHYGQFTCEGCKSFFKRSVRRQLTYTCRGARQCLIDLHHRNQCQYCRFQKCLRKGM

**>EgTR4** (EGR_01863.1 scaffold:ASM52419v1)

CLVCGDVASGRHYGVISCEGCKGFFKRSIRGHVNYVCRSNKHCIVNKAFRNRCQYCRMQKCLLVGM

**>EgTLL** (EG_S00017 scaffold:ASM52419v1)

CKVCKDHSSGKHYGIYACDGCAGFFKRSIRRNRQYTCKSRNGQESCCRVDKPHRNQCRACRLKKCLEAGM

**>EgNR4A** (EGR_02914.1 scaffold:ASM52419v1)

CLVCGDNAACQHYGVRTCEGCKGFFKRTIQKNAHYVCLQTKNCIVDKRRRNRCQYCRFQKCLKVGM

**>EgFTZ-F1a** (EGR_05526.1 scaffold:ASM52419v1)

CPVCGDKVSGYHYGLQTCESCKGFFKRTVQNKKAYQCTEAGVCIIDRVHRKRCAYCRFKKCLSAGM

**>EgFTZ-F1b** (EG_S00201 scaffold:ASM52419v1)

CPICGDKISGYHYGIFCCESCKGFFKRTVQNSKRYACHMATPTSLCDITATSRKKCPACRFVKCLEKGM

**>Eg2DBDa** (EGR_06786.1 scaffold:ASM52419v1)

CQICGELAAGFHHGAYVCEACKKFFMRHSLANGRSTTTCPSGGNCVIAKSSRGRCQQCRYKKCLEVGM

CRVCGGRSSGFHFGALTCEGCKGFFRRTEESASRLVCVGGKDNCTITPRSRNVCKTCRFRRCLRAGM

**>Eg2DBDgb** (EGR_04794.1 scaffold:ASM52419v1)

CQVCGQPSVGFHHRAYKCEACKKFFTRHLTNRIKKEKSTFGEGDSAKSSTQGGETHPDLNVVCPMGGNCKIEGPGRGKCPHCRFRKCLDLGM

CRVCGGPSSGFHFGALTCEGCKGFFRRTVHSNSIPQCLGNQTCRINRNMCKSCRFKKCLEVGM

**>Eg2DBDg** (EgrG_000240200 scaffold:EGRAN001)

CDICGDVSAGFHCNAYVCEACKKFFIRSSKNENYAKYSCSKVNACEVNKDTRTHCQRCRFYKCLRIGM

CRVCGAQSSGFHFGAITCEGCKGFFRRTISERDNQRYTCRNGGTCLITLATRNNCKSCRYRKCLSVGM

**>EgOther** (EGR_02251.1 scaffold:ASM52419v1)

CAICGATATGKHYGALSCDSCRAFFGTATHLGLRCECSGKSNIHDNNHQLRCHSCRLRKCLAVGM

**5. NRs in *Echinococcus multilocularis* (17)(** **prjeb122)**

**>EmTR** (scaffold:EMULTI002:pathogen_EmW_scaffold_10)

CVVCGDNATGFHYRAMTCEGCKGFFRRSVQKKLEYTCKFNGNCSVGDKQNRNSCQKCRFDRCIKGGM

**>EmE78** (scaffold:EMULTI002:pathogen_EmW_scaffold_02)

CHVCGDKASGYHYGVISCEGCKGFFRRSIQKQIEYKCLREGKCTVVRLNRNRCQYCRFRKCIAVGM

**>EmHR96a** (scaffold:EMULTI002:pathogen_EmW_scaffold_05)

CVVCGEPASGYNFDRLTCESCKAFFRRNALKPKEKIKACGRNGDCNIEGSQRKHCPSCRLEKCLAVGM

**>EmHR96b** (scaffold:EMULTI002:pathogen_EmW_scaffold_02)

CRVCGDRAVNHNFGQLTCESCKAFFRRNAHKALPSISAALQSSDCATTTDAYLQALQRGLRDLTCTSKSGEHVVSPSTRRECPACRLKRCFLIGM

**>EmHR96c** (scaffold:EMULTI002:pathogen_EmW_scaffold_01)

CAVCGDHAVGFNFGAIACESCKAFFRRNALRATMPPCLFNGSCLIQVKTRRFCSPCRLSKCFAVGM

**>EmNR1b** (scaffold:EMULTI002:pathogen_EmW_scaffold_05)

CLVCGDNASGIHYGVLSCEGCKGFFRRALQDKRAHLRKCIRQDRCKISIKTRNHCQPCRLRRCLELGM

**>EmHNF4** (scaffold:EMULTI002:pathogen_EmW_scaffold_06)

CLICGDKATGKHYGAYSCDGCKGFFRRSVRRKHTYSCRYNRTCTIDKDMRNQCRFCRLKKCFRVGM

**>EmTR4** (scaffold:EMULTI002:pathogen_EmW_scaffold_04)

CLVCGDVASGRHYGVISCEGCKGFFKRSIRGHVNYVCRSNKHCIVNKAFRNRCQYCRMQKCLLVGM

**>EmTLL** (scaffold:EMULTI002:pathogen_EmW_scaffold_08)

CKVCKDHSSGKHYGIYACDGCAGFFKRSIRRNRQYTCKSRNGQESCCRVDKPHRNQCRACRLKKCLEAGM

**>EmCoup-TFI** (scaffold:EMULTI002:pathogen_EmW_scaffold_01)

CVVCGDKSSGKHYGQFTCEGCKSFFKRSVRRQLTYTCRGARQCLIDLHHRNQCQYCRFQKCLRKGM

**>EmNR4A** (scaffold:EMULTI002:pathogen_EmW_scaffold_02)

CLVCGDNAACQHYGVRTCEGCKGFFKTIQKNAHYVCLQTKNCIVDKRRRNRCQYCRFQKCLKVGM

**>EmFTZ-F1a** (scaffold:EMULTI002:pathogen_EmW_scaffold_01)

CPVCGDKVSGYHYGLQTCESCKGFFKRTVQNKKAYQCTEAGVCIIDRVHRKRCAYCRFKKCLSAGM

**>EmFTZ-1b** (scaffold:EMULTI002:pathogen_EmW_scaffold_01)

CPICGDKISGYHYGIFCCESCKGFFKRTVQNSKRYACHMATPTSLCDITATSRKKCPACRFVKCLEKGM

**>Em2DBDa** (scaffold:EMULTI002:pathogen_EmW_scaffold_07)

CQICGELAAGFHHGAYVCEACKKFFMRHSLANGRSTTTCPSGGNCVIAKSSRGRCQQCRYKKCLEVGM

CRVCGGRSSGFHFGALTCEGCKGFFRRTEESASRLVCVGGKDNCTITPRSRNVCKACRFRRCLRAGM

**>Em2DBDgb** (scaffold:EMULTI002:pathogen_EmW_scaffold_03)

CQVCGQPSVGFHHRAYVCEACKKFFTRHLTNRIKKEKSTFGEGDSAKSSTQGGETHPDLNVVCPMGGNCKIEGPGRGKCPHCRFRKCLDLGM

CRVCGGPSSGFHFGALTCEGCKGFFRRTVHSNSIPQCLGNQTCRITPSNRNMCKSCRFKKCLEVGM

**>Em2DBDg** (scaffold:EMULTI002:pathogen_EmW_scaffold_04)

CDICGDVSAGFHCNAYVCEACKKFFIRSSKNENYAKYSCSKVNACEVNKDTRTHCQRCRFYKCLRIGM

CRVCGAQSSGFHFGAITCEGCKGFFRRTISERDNQRYTCRNGGTCLITLATRNNCKSCRYRKCLSVGM

**>Emother** (scaffold:EMULTI002:pathogen_EmW_scaffold_03)

CAICGATATGKHYGALSCDSCRAFFGTATHLGLRCECSGKSNIHDNNHQLRCHSCRLRKCLAVGM

**6. NRs in *Echinostoma caproni* (22) (prjeb1207)**

**>EcaTRa** (scaffold:E_caproni_Egypt_v1_5_4:ECPE_scaffold0004705)

CVVCGDNATGFHYRAMTCEGCKGFFRRSIQKKLVYTCKFQGHCSVSDKQNRNSCQKCRFDRCIRGGM

**>EcaTRb** (scaffold:E_caproni_Egypt_v1_5_4:ECPE_scaffold0004705)

CVVCGDNATGFHYRAMTCEGCKGFFRRSVQKKLVYTCKFNGRCSVSDKQNRNSCQKCRFDRCIKGGM

**>EcaE78** (scaffold:E_caproni_Egypt_v1_5_4:ECPE_scaffold0011800, ECPE_scaffold0000836)

CKVCGDKASGYHYGVISCEGCKGFFRRSIQKQIEYKCLRDGKCLVIRLNRNRCQYCRFRKCLAVGM

**>EcaHR96a** (scaffold:E_caproni_Egypt_v1_5_4:ECPE_scaffold0017032, ECPE_scaffold0021492)

CIVCGEPASGYNFDRLTCESCKAFFRRNALKPKDKIKACSRGGGCVIEGNQRKHCPSCRLEKCFNVGM

**>EcaHR96b** scaffold:E_caproni_Egypt_v1_5_4: (ECPE_scaffold0000667)

CKVCNDRAVNHNFGQLTCESCKAFFRRNAHKELTCTLKSGEHEITPTTRRECPACRLKKCFRVGM

**>EcaHR96c** (scaffold:E_caproni_Egypt_v1_5_4:ECPE_scaffold0004630, ECPE_scaffold0043670)

CSVCGDSAVGFNFGAIACESCKAFFRRSAHKAQVTTCLFNERCTIEVATRRFCSHCRLKKCFSVGM

**>EcaNR1a** (scaffold:E_caproni_Egypt_v1_5_4:ECPE_scaffold0008794)

CVVCGDKSSGSHYGVFTCEGCKGFFRRAIQRNQSYVCARTGKCEVNRALRNKCQHCRLLKCLASGM

**>EcaRXR1** (scaffold:E_caproni_Egypt_v1_5_4:ECPE_scaffold0000439)

CVICGDTASGRHYGVISCEGCKGFFKRSVRKQLQFTCRGSGHCPVDRSKRTRCQHCRLEQCLSKGM

**>EcaRXR2** (scaffold:E_caproni_Egypt_v1_5_4:ECPE_scaffold0007324)

CSICGDRASGKHYGVFSCEGCKGFFKRTVRKELIYTCHDNQRCQIDKRLRNRCQYCRYQKCLLVGM

**>EcaTR4** (scaffold:E_caproni_Egypt_v1_5_4:ECPE_scaffold0002381)

CKVCGDKASGKGRHYGVVSCEGCKGFFKRSIRGHVSYACRSDQQCLVNKAFRNRCQYCRLQKCLAVGM

**>EcaPNR** (scaffold:E_caproni_Egypt_v1_5_4:ECPE_scaffold0000245)

CSVCGDVSSGKHYGILACNGCSGFFKRSVRRKLIYRCQAGTGMCVIDKAHRNQCQACRLKKCIRMGM

**>EcaCoup-TFI** (scaffold:E_caproni_Egypt_v1_5_4:ECPE_scaffold0017055)

CVVCGDKSSGKHYGQHTCEGCKSFFKRSVRRKLTYTCRGNRQCPIDIHHRNQCQYCRFQKCVKAGM

**>EcaCoup-TFII** (scaffold:E_caproni_Egypt_v1_5_4:ECPE_scaffold0000968)

SANYVCRSGGQCPVDPQRRNQCQACRMSRCLTAGM

**>EcaTLL** (scaffold:E_caproni_Egypt_v1_5_4:ECPE_scaffold0000151)

CRVCKDHSSGKHYGIYACDGcagffkRSIRRNRQYVCKNRSVVGGKPNPGNCRIDKSHRNQCRACRLRKCLEVGM

**>EcaHNF4** (scaffold:E_caproni_Egypt_v1_5_4:ECPE_scaffold0000417)

GKHYGAFSCDGCKGFFRRSVRRNHSYSCRHKRQCIVTKDKRNQCRYCRLRKCFRVGM

**>EcaDSF** (scaffold:E_caproni_Egypt_v1_5_4:ECPE_scaffold0008450)

CGVCGDHSSGKHYGIYSCDGCSGFFKRSIHKNRAYTCKAVGELKGQCVIDRNRRNQCRACRLKKCFQANM

**>EcaNR4A** (scaffold:E_caproni_Egypt_v1_5_4:ECPE_scaffold0000229, ECPE_scaffold0015305)

CLVCGDSAACQHYGVRTCEGCKGFFKRTIQKNAQYVCLQSKNCVVDKRRRNRCQYCRFQKCLKVGM

**>EcaFTZ-F1a** (scaffold:E_caproni_Egypt_v1_5_4:ECPE_0000392401)

CPVCGDRLSGYHYGLPTCESCKGFFKRTVQNKKEYHCTEQGQCVIDRVHRKRCAFCRFQKCLSVGM

**>EcaFTZ-F1b** (scaffold:E_caproni_Egypt_v1_5_4:ECPE_scaffold0000419)

CPICGDRISGYHYGIFCCESCKGFFKRTVQNAKRYTCHHPNASSLCEINVASRKKCPACRFLKCVDKGM

**>Eca2DBDa** (scaffold:E_caproni_Egypt_v1_5_4:ECPE_scaffold0005536)

CQVCGELAAGFHHGAYVCEACKKFFMRHSLTDTKPTNVCPTGGNCVVAKGSRGKCQICRYRKCLYVGM

CRVCGGRSSGFHFGALTCEGCKGFFRRTEGSAGSLVCVGGQNACTITPRSRNACKSCRFRRCIAAGM

**>Eca2DBDb** (scaffold:E_caproni_Egypt_v1_5_4:ECPE_contig0006292, ECPE_contig0007983, ECPE_scaffold0010896)

CQICGQPAVGFHHRAYVCEACKKFFMRHTAARYRNSEAGGPSPAESVCPMGGQCRVEGPGRGKCPHCRYRKCLDLGM GFFRRTVLSSVRLECLSNNDCPITPANRNMCKSCRFQRCLAVGM

**>Eca2DBDg** (scaffold:E_caproni_Egypt_v1_5_4:ECPE_scaffold0021823, ECPE_scaffold0001297)

CDICGDVSAGFHCNAYVCEACKKFFIRSSKGDNFAKYTCTKSNVCEINKDTRTHCQRCRYQKCLRLGM

CRVCGAKSSGFHFGAITCEGCKGFFRRTINERESQRYTCRNGGNCAVTGATRNNCKSCRYRRCLAVGM

**7. NRs in *Fasciola hepatica* (22)(** **prjeb25283, prjna179522)**

**>FhTRa** (prjeb25283, scaffold:Fasciola_10x_pilon:scaffold10x_919_pilon)

CVVCGDNATGFHYRAMTCEGCKGFFRRSVQKKLVYTCKFNGRCSVSDKQNRNSCQKCRFDRCIKGGM

**>FhTRb** (prjeb25283, scaffold:Fasciola_10x_pilon:scaffold10x_277_pilon)

CVVCGDNATGFHYRAMTCEGCKGFFRRSIQKKLVYTCKFQGRCSVSDKQNRNSCQKCRFDRCIRGGM

**>FhE78** (prjeb25283, scaffold:scaffold:Fasciola_10x_pilon:scaffold10x_798_pilon)

CKVCGDKASGYHYGVISCEGCKGFFRRSIQKQIEYKCLRDGKCLVIRLNRNRCQYCRFRKCLAVGM

**>FhHR96a** (prjeb25283, scaffold:Fasciola_10x_pilon:scaffold10x_1084_pilon)

CIVCGEPASGYNFDRLTCESCKAFFRRNALKPKDKIKACSRGGGCIIEGNQRKHCPSCRLEKCFAVGM

**>FhHR96b** (prjeb25283, scaffold:Fasciola_10x_pilon:scaffold10x_1052_pilon)

CKVCNDRAVNHNFGQLTCESCKAFFRRNAHKDLTCTLKAGEHEITPTTRRECPACRLKKCFRVGM

**>FhHR96c** (prjeb25283, scaffold:Fasciola_10x_pilon:scaffold10x_43_pilon)

CSVCGDSAVGFNFGAIACESCKAFFRRSAHKAQMTSCLFNERCTVDVPTRRFCSHCRLKKCFSVGM

**>FhNR1a** (prjeb25283, scaffold:Fasciola_10x_pilon:scaffold10x_1068_pilon)

CVVCGDKSSGSHYGVTTCEGCKGFFRRAVQRNQSYACARDGKCEVNRTLRNKCQHCRLLKCLACGM

**>FhRXR1** (prjeb25283, scaffold:Fasciola_10x_pilon:scaffold10x_760_pilon)

CAICGDTASGRHYGVISCEGCKGFFKRAVRKQIQFTCRGSGQCPVDRSKRTRCQHCRLEQCLAKGM

**>FhRXR2** (prjeb25283, scaffold:Fasciola_10x_pilon:scaffold10x_1013_pilon)

CSICGDRATGKHYGVFSCEGCKGFFKRTVRKDLVYTCHDNHRCQIDRRLRNRCQYCRYQKCLMVGM

**>FhTR4** (prjeb25283, scaffold:Fasciola_10x_pilon:scaffold10x_147_pilon)

CKVCGDKASGRHYGVVSCEGCKGFFKRSIRGHVSYACRSDQQCLVNKAFRNRCQYCRLQKCLAVGM

**>FhCoup-TFI** (prjeb25283, scaffold:Fasciola_10x_pilon:scaffold10x_2248_pilon)

CVVCGDKSSGKHYGQHTCEGCKSFFKRSVRRKLTYTCRGNRQCPIDIHHRNQCQYCRFQKCVKAGM

**>FhCoup-TFII** (prjeb25283, scaffold:Fasciola_10x_pilon:scaffold10x_1995_pilon)

CQVCGDRSSGKHYGQYTCEGCKSFFKRSVRKSANYVCRSGGQCPVDPQRRNQCQACRMSRCLTAGM

**>FhHNF4** (prjeb25283, scaffold:Fasciola_10x_pilon:scaffold10x_56_pilon)

GKHYGAYSCDGCKGFFRRSVRRNHSYTCRQKRQCVVTKDKRNQCRFCRLRKCFRVGM

**>FhPNR** (prjeb25283, scaffold:Fasciola_10x_pilon:scaffold10x_908_pilon)

CSVCGDVSSGKHYGILACNGCSGFFKRSVRRKLIYRCQAGTGMCVIDKAHRNQCQACRLKKCIGMGM

**>FhDSF** (prjeb25283, scaffold:Fasciola_10x_pilon:scaffold10x_954_pilon)

CGVCGDHSSGKHYGIYSCDGCSGFFKRSIHKNRSYTCKAEGDLKGHCTIDRNRRNQCRACRLKKCFLANM

**>FhTLL** (prjeb25283, scaffold:Fasciola_10x_pilon:scaffold10x_1176_pilon)

CRVCKDHSSGKHYGIYACDGCAGFFKRSIRRNRQYVCKNRSTIGGKSNAGNCRIDKSHRNQCRACRLRKCLEVGM

**>FhNR4A** (prjeb25283, scaffold:Fasciola_10x_pilon:scaffold10x_323_pilon)

CLVCGDSAACQHYGVRTCEGCKGFFKRTIQKNAQYVCLQSKNCVVDKRRRNRCQYCRFQKCLKVGM

**>FhFTZ1-F1a** (prjeb25283, scaffold:Fasciola_10x_pilon:scaffold10x_581_pilon)

CPICGDRVSGYHYGLPTCESCKGFFKRTVQNKKEYHCTEQGQCVIDRVHRKRCAFCRFQKCISVGM

**>FhFTZ-F1b** (prjeb25283, scaffold:Fasciola_10x_pilon:scaffold10x_314_pilon)

CPICGDKISGYHYGIFCCESCKGFFKRTVQNAKRYTCHHPNTSSLCEINVTSRKKCPACRFLKCVDKGM

**>Fh2DBDa** (prjeb25283, scaffold:Fasciola_10x_pilon:scaffold10x_122_pilon)

CQVCGELAAGFHHGAYVCEACKKFFMRHSLTDTKPTNVCPTGGNCVVAKGSRGKCQICRYRKCLYVGM

CRVCGGRSSGFHFGALTCEGCKGFFRRTEGSAGSLVCVGGQNACTITPRSRNACKSCRFRRCIAAGM

**>Fh2DBDb** (prjeb25283, scaffold:Fasciola_10x_pilon:scaffold10x_4_pilon, scaffold:Fasciola_10x_pilon:scaffold10x_284_pilon)

CQICGQPAVGFHHRAYVCEACKKFFMRHTAARYRNSEAGGPSSTESVCPMGGQCRVEGPGRGKCPHCRYRKCLDLGM

CRVCGGPSSGFHFGALTCEGCKGFFRRTVLSSVRLECLSNNDCPITPANRNMCKSCRFQRCLAVGM

**>Fh2DBDg** (prjeb25283, scaffold:Fasciola_10x_pilon:scaffold10x_303_pilon)

CDICGDISAGFHCNAYVCEACKKFFIRSSKGDNFTKYTCTKSNVCEINKDTRTHCQRCRYQKCLRLGM

CRVCGAKSSGFHFGAITCEGCKGFFRRTINERESQRYTCRNGGNCAVTGATRNNCKSCRYRRCLAVGM

**8. NRs in *Gyrodactylus salaris* (18)(** **prjna244375)**

**>GsTR** (scaffold:Gsalaris_v1:scf7180006948568)

CVVCGDNATGFHYRAMTCEGCKGFFRRSVQKKLVYTCKFMGRCSVSDKQNRNSCQKCRFDRCIKGGM

**>GsE78** (scaffold:Gsalaris_v1:scf7180006953175)

CKVCGDKASGYHYGVISCEGCKGFFRRSIQKQIEYKCLREGKCLVVRLNRNRCQFCRFKKCSEAGM

**>GsHR96a** (scaffold:Gsalaris_v1:scf7180006950187)

CVVCGEPASGYNFDRLTCESCKAFFRRNALKPRDKIKKCNRHGQCNIEGSQRKHCPSCRLEKCLAAGM

**>GsHR96b** (scaffold:Gsalaris_v1:scf7180006950516)

CKVCGDRAVNHNFGQLTCESCKAFFRRNAHKELTCTSKTNGHYISPSTRRECPACRLKRCFFVGM

**>GsHNF4** (scaffold:Gsalaris_v1:scf7180006950393)

CLICGDKATGKHYGAQSCDGCKGFFRRSVRKSHQYQCRYNKDCIIDKDKRNQCRFCRLKKCFNVGM

**>GsRXR** (scaffold:Gsalaris_v1:scf7180006952986)

CSICGDEASGKHYGVLSCEGCKGFFKRTIRKDLEYVCRDSNQCKIDKRARNRCQLCRFQKCLSAGM

**>GsTR4**  (scaffold:Gsalaris_v1:scf7180006949311)

CKVCGDKASGRHYGVISCEGCKGFFKRSIRGHVNYACRGEQNCLVNKAYRNRCQFCRLQKCLSVGM

**>GsPNR** (scaffold:Gsalaris_v1:scf7180006950447)

CIVCGDVSSGKHYGILACNGCSGFFKRSVRRKLIYRCQAGTDLCIIDKAHRNQCQSCRYKKCLQMGM

**>GsTLL**  (scaffold:Gsalaris_v1:scf7180006952656)

CRVCRDHSSGKHYGIYACDGCAGFFKRSIRRNRQYTCKSRAGQESSCKIDKSHRNQCRACRLKKCLEAGM

**>GsDSF** (scaffold:Gsalaris_v1:scf7180006947763, scf7180006947525)

CKVCGDRSSGKHYGIFSCDGCSGFFKRSIHKNRIYTCKAAGELKGQCYVDRNHRNQCRACRLDKCFASKM

**>GsCoup-TFI** (scaffold:Gsalaris_v1:scf7180006950759)

CVVCDDKSSGKHYGQYTCEGCKSFFKRSVRRSLTYKCRQNMNCLITLDNRNHCQYCRFQKCLLFGM

**>GsCoup-TFII** (scaffold:Gsalaris_v1:scf7180006953298)

CLVCGDRSSGKHYGQFSCEGCKSFFKRAIRKNSHFVCRSNNQCQIDSQRRNQCQQCRLTRCFLVGM

**>GsNR4A**  (scaffold:Gsalaris_v1:scf7180006951034)

CLVCGDTAACQHYGVRTCEGCKGFFKRTIQKNAHYVCLQNKSCIVDKRRRNRCQYCRFQKCIKVGM

**>GsFTZ-F1b** (maker-scf7180006951109-exonerate_protein2genome-gene-0.17-mRNA-1)

CPICGDRISGYHYGIFCCESCKGFFKRTVQNGKRYSCNIPNDGSQLCEVSVASRKRCPACRFMKGM

**>Gs2DBDa** (scaffold:Gsalaris_v1:scf7180006950798, scf718000695049)

CQVCGESAAGFHHGAYVCEACKKFFMRHSLTDSKLQTACPTGGKCSVTKGNRGKCQQCRFKKCLEIGM

CRVCGGRSSGFHFGALTCEGCKGFFRRTEGSAGSLVCVGGQNACTITPRSRNACKSCRFRRCIAAGM

**>Gs2DBDb**  (scaffold:Gsalaris_v1:scf7180006948306)

CQVCNQPAAGFHHRAYVCEACKKFFMRHATPKTNKANEPAGNLVLTSCPMGGKCRVVGPGRGKCASCRFRKCLDIGM

CRICGGSSSGFHFGAITCEGCKGFFRRTIMSNVKLDCSGSGNCSITPSSRNICKSCRMKKCLDIGM

**>Gs2DBDg** (scaffold:Gsalaris_v1:scf7180006948095)

CEICGDISAGFHCNAYVCEACKKFFVRSSKNDNYSKYICSKINLCEINKDTRTHCQKCRFYKCLKIGM

CRVCGGRSSGFHFGALTCEGCKGFFRRTEGTNTRLVCRSRNACKSCRFKRCLAVGM

**>GsOther** (scaffold:Gsalaris_v1:scf7180006953099)

CVVCGNRSNGVHYNAVTCEPCKVFFKRTVQAKKVYSCKSKNKRCSVQVRGRASCSFCRFFHCLKNGM

**9. NRs in *Hydatigera taeniaeformis* (17)(prjeb534)**

**>HtTR** (scaffold:H_taeniaeformis_Canary_Islands_v1_0_4:TTAC_scaffold0000279)

CVVCGDNATGFHYRAMTCEGCKGFFRRSVQKKLEYTCKFNGNCSVGDKQNRNSCQKCRFERCLKGGM

**>HtE78** (scaffold:H_taeniaeformis_Canary_Islands_v1_0_4:TTAC_contig0001048)

CQVCGDKASGYHYGVISCEGCKGFFRRSIQKQIEYKCLREGKCTVVRLNRNRCQYCRFRKCIAAGM

**>HtHR96a** (scaffold:H_taeniaeformis_Canary_Islands_v1_0_4:TTAC_contig0000078)

CVVCGEPASGYNFDRLTCESCKAFFRRNALKPKEKIKACGRNGDCNIEGSQRKHCPSCRLEKCLAVGM

**>HtHR96b** (scaffold:H_taeniaeformis_Canary_Islands_v1_0_4:TTAC_contig0001019,TTAC_contig0004740)

CRVCGDRAVNHNFGQLTCESCKAFFRRNAHKALPSIIAALRSSGDATTTDAYLQALQRGLRDLTCTSKSGEHVVSPSTRRECPACRLKRCFLIGM

**>HtHR96c** (H_taeniaeformis_Canary_Islands_v1_0_4:TTAC_scaffold0000595)

CAVCGDHAVGFNFGAIACESCKAFFRRNALRATMPPCLFNGSCLIQVKTRRFCSPCRLSKCFAVGM

**>HtNR1b** (scaffold:H_taeniaeformis_Canary_Islands_v1_0_4:TTAC_scaffold0000142)

CQVCGDTASGIHYGVLSCEGCKGFFRRALQDKRAHLRKCIRQDRCKISIKTRNHCQPCRLRRCLELGM

**HtCoup-TFI** (scaffold:H_taeniaeformis_Canary_Islands_v1_0_4:TTAC_contig0000144)

CVVCGDKSSGKHYGQFTCEGCKSFFKRSVRRQLTYTCRGARQCLIDLHHRNQCQYCRFQKCLRMGM

**>HtTR4** (scaffold:H_taeniaeformis_Canary_Islands_v1_0_4:TTAC_contig0000212)

CLVCGDVASGRHYGVISCEGCKGFFKRSIRGHVNYVCRSNKHCIVNKAFRNRCQYCRMQKCLLVGM

**>HtHNF4** (scaffold:H_taeniaeformis_Canary_Islands_v1_0_4:TTAC_contig0000092)

CLICGDKATGKHYGAYSCDGCKGFFRRSVRRKHTYSCRYNRACTIDKDMRNQCRFCRLKKCFRVGM

**>HtTLL** (scaffold:H_taeniaeformis_Canary_Islands_v1_0_4:TTAC_scaffold0000230)

CKVCKDHSSGKHYGIYACDGCAGFFKRSIRRNRQYTCKSRNGQESCCRVDKPHRNQCRACRLKKCLEAGM

**>HtNR4A** (scaffold:H_taeniaeformis_Canary_Islands_v1_0_4:TTAC_scaffold0004217)

CLVCGDNAACQHYGVRTCEGCKGFFKRTIQKNAHYVCLQTKNCIVDKRRRNRCQYCRFQKCLKVGM

**>HtFTZ-F1a** (scaffold:H_taeniaeformis_Canary_Islands_v1_0_4:TTAC_scaffold0000048)

CPVCGDKVSGYHYGLQTCESCKGFFKRTVQNKKSYQCTEAGVCIIDRIHRKRCAYCRFKKCLSVGM

**>HtFTZ-F1b** (scaffold:H_taeniaeformis_Canary_Islands_v1_0_4:TTAC_scaffold0000128)

CPICGDKISGYHYGIFCCESCKGFFKRTVQNSKRYACHMATPSSLCDITATSRKKCPACRFVKCLEKGM

**>Ht2DBDa** (scaffold:H_taeniaeformis_Canary_Islands_v1_0_4:TTAC_contig0002441, TTAC_contig0006749, TTAC_scaffold0003427)

CQICGELAAGFHHGAYVCEACKKFFMRHSLANGRSTTTCPSGGNCVIAKSSRGRCQQCRYKKCLEVGM

CRVCGGRSSGFHFGALTCEGCKGFFRRTEESASRLVCVGGKDNCTITPRSRNVCKACRFRRCLRAGM

**>Ht2DBDb** (scaffold:H_taeniaeformis_Canary_Islands_v1_0_4:TTAC_scaffold0000429)

CQVCGQPSVGFHHRAYVCEACKKFFTRHLTNRIKKDKSTNGEGESAKASTQRVEIYPDLNVVCPMGGNCKIEGPGRGKCPHCRFRKCLDLGM

CRVCGGPSSGFHFGALTCEGCKGFFRRTVHSNSIPQCLGNQTCRITPSNRNMCKSCRFKKCLEVGM

**>Ht2DBDg** (scaffold:H_taeniaeformis_Canary_Islands_v1_0_4:TTAC_contig0000018)

CDICGDVSAGFHCNAYVCEACKKFFIRSSKNENYTKYSCSKVNACEVNKDTRTHCQRCRFYKCLRIGM

CRVCGAQSSGFHFGAITCEGCKGFFRRTISERDNQRYTCRNGGTCLITLATRNNCKSCRYRKCLSVGM

**>HtOther** (scaffold:H_taeniaeformis_Canary_Islands_v1_0_4:TTAC_scaffold0000097)

CAVCGAPATGKRYGAVSCDSCSAFFGVATLLGIQCECSNKPSPSDDAYHLHCHYCMLTKCLAVGM

**10. NRs in *Hymenolepis diminuta* (15)(** **prjeb507)**

**>HdTR** (scaffold:H_diminuta_Denmark_v1_0_4:HDID_contig0002039)

CVVCGDNATGFHYRAMTCEGCKGFFRRTIQKKLTYTCKFNNNCNVMHKQNRNSCQKCRFDKCIEGGM

**>HdE78** (scaffold:H_diminuta_Denmark_v1_0_4:HDID_contig0002597)

CMVCGDKASGYHYGVISCEGCKGFFRRSIQKQIEYKCLRDGNCVVVRLNRNRCQYCRFKKCLKVGM

**>HdHR96a** (scaffold:H_diminuta_Denmark_v1_0_4:HDID_contig0000058)

CVVCEEPASGYNFDRLTCESCKAFFRRNALKPKDKIKACSRNGDCVVVGSQRKHCPSCRLEKCLAVGM

**>HdHR96b** (scaffold:H_diminuta_Denmark_v1_0_4:HDID_scaffold0000005)

CRVCGDRAVNHNFGQLTCESCKAFFRRNAHKALPSISAALQSSGSKSITMEVYIKAIQHGLRDLTCTSKSGEHVVSPSTRRECPACRLKRCFLIGM

**>HdHR96c** (scaffold:H_diminuta_Denmark_v1_0_4:HDID_scaffold0000567)

CAVCGDHAVGFNFGAIACESCKAFFRRNALRASMPSCLFNGSCMIQVKTRRFCSPCRLSKCFAVGM

**>HdNR1b** (scaffold:H_diminuta_Denmark_v1_0_4:HDID_scaffold0000278)

CLVCGDIASGIHYGVLSCEGCKGFFRRALQDKRAHLRKCIRQDCCKISIKTRNHCQPCRLRRCLELGM

**>HdHNF4** (scaffold:H_diminuta_Denmark_v1_0_4:HDID_scaffold0000056)

CLICGDKATGKHYGAYSCDGCKGFFRRSVRRKHTYSCRYNRTCTIDKDMRNQCRFCRLKKCFRVGM

**>HdTR4** (scaffold:H_diminuta_Denmark_v1_0_4:HDID_contig0000166)

CRVCGDVASGRHYGVISCEGCKGFFKRSIRGHVNYVCRSNRKCVVNKAYRNRCQYCRMQKCLDAGM

**>HdTLL** (scaffold:H_diminuta_Denmark_v1_0_4:HDID_scaffold0000245)

CKVCKDHSSGKHYGIYACDGAGFFKRSIRRNRQYTCKSKNGQESSCRVDKPHRNQCRACRLKKCLEAGM

**>HdNR4A** (scaffold scaffold:H_diminuta_Denmark_v1_0_4:HDID_contig0000048)

CLVCGDNAACQHYGVRTCEGCKGFFKRTIQKNAHYVCLQTKNCIVDKRRRNRCQYCRFQKCLKVGM

**>HdFTZ-F1b** (scaffold:H_diminuta_Denmark_v1_0_4:HDID_contig0000719)

CPICGDKISGYHYGIFCCESCKGFFKRTVQNSKRYACHMATPASLCEITATSRKKCPACRFVKCLEKGM

**>Hd2DBDa** (scaffold:H_diminuta_Denmark_v1_0_4:HDID_scaffold0000061)

CQICGELAAGFHHGAYVCEACKKFFMRHSLANGRSTTTCPSGGNCVIVKNSRGRCQQCRYKKCLEVGM

CRVCGGRSSGFHFGALTCEGCKGFFRRTEETASRLVCVGGKDNCTITPRSRNVCKSCRFRRCLRAGM

**>Hd2DBDb** (scaffold:H_diminuta_Denmark_v1_0_4:HDID_contig0001417)

CQVCGQPSVGFHHRAYVCEACKKFFTRHLTNRIKKDKTSVCGDSLGVGDSLKPMSMGGGGNSEIYADLNVVCPMGGNCKIEGPGRGKCPHCRFRKCLDLGM

CRVCGGPSSGFHFGALTCEGCKGFFRRTVNSNSIPQCLGNQTCRITPSNRNMCKSCRFKKCLEVGM

**>Hd2DBDg** (scaffold:H_diminuta_Denmark_v1_0_4:HDID_contig0000655)

CAICGDVSAGFHCNAFVCEACKKFFIRSSKNENFAKYSCSKVNACEVSKDTRTHCQRCRFYKCLSIGM

CRVCGAQSSGFHFGAITCEGCKGFFRRTISERDNQRYTCRNGGTCLVTLATRNNCKSCRYRKCLSVGM

**>Hdother**  (scaffold:H_diminuta_Denmark_v1_0_4:HDID_scaffold0000216)

CVVCGDTNSSRHYGQYACEGCKGFFQKTVTGSLMYELKCNGLCLIEVNTRNRCQYCGFQKCLNVGM

**11. NRs in *Hymenolepis microstoma* (15)(prjeb124)**

**>HmTR** (scaffold:HMN_v3:HMN_06_pilon:16412855:16415733)

CVVCGDNATGFHYRAMTCEGCKGFFRRTIQKKLTYTCKFNNNCNVMHKQNRNSCQKCRFDKCIEGGM

**>HmE78** (scaffold:HMN_v3:HMN_04_pilon:17025213:17034739)

CMVCGDKASGYHYGVISCEGCK>GFFRRSIQKQIEYKCLRDGNCLVVRLNRNRCQYCRFKKCLKVGM

**>HmHR96a** (scaffold:HMN_v3:HMN_05_pilon:12837600:12860115)

CVVCEEPASGYNFDRLTCESCKAFFRRNALKPKDKIKACSRNGDCVVVGSQRKHCPSCRLEKCLAVGM

**>HmHR96b** (scaffold:HMN_v3:HMN_04_pilon:1:25589455:1)

CRVCGDRAVNHNFGQLTCESCKAFFRRNAHKALPSISAVLQSSGTKSITMEVYIKAIQYGLRLTCTSKSGEHVVSPSTRRECPACRLKRCFLIGM

**>HmHR96c** (scaffold:HMN_v3:HMN_05_pilon:12837600:12860115)

CAVCGDHAFGFNFGAIACESCKAFFRRNALRAS>MPPCLFNDSCMIQVKTRRFCSPCRLSKCFAVGM

**>HmNR1b** (scaffold:HMN_v3:HMN_05_pilon:13668616:13688115)

CLVCGDIASGIHYGVLSCEGCKGFFRRALQDKRAHLRKCIRQDCCKISIKTRNHCQPCRLRRCLELGM

**>HmHNF4** (scaffold:HMN_v3:HMN_06_pilon:7637619:7647198)

CLICGDKATGKHYGAYSCDGCKGFFRRSVRRKHTYSCRYNRTCTIDKDMRNQCRFCRLKKCFRVGM

**>HmTR4** (scaffold:HMN_v3:HMN_03_pilon:15450387)

CRVCGDVASGRHYGVISCEGCKGFFKRSIRGHVNYVCRSNRKCVVNKAYRNRCQYCRMQKCLDAGM

**>HmTLL** (scaffold:HMN_v3:HMN_01_pilon:4243845:4252332)

CKVCKDHSSGKHYGIYACDG>CAGFFKRSIRRNRQYTCKSKNGQESSCRVDKPHRNQCRACRLKKCLEAGM

**>HmNR4A** (scaffold:HMN_v3:HMN_04_pilon:10887274:10901789)

CLVCGDNAACQHYGVRTCEGCKGFFK>RTIQKNAHYVCLQTKNCIVDKRRRNRCQYCRFQKCLKVGM

**>HmFTZ-F1b** (scaffold:HMN_v3:HMN_01_pilon:26221131:26233750)

CPICGDKISGYHYGIFCCESCKGFFKRTVQNSKRYACHMATPASLCEITATSRKKCPACRFVKCLEKGM

**>Hm2DBDa** (scaffold:HMN_v3:HMN_05_pilon:3478837:3495186)

CQICGELAAGFHHGAYVCEACKKFFMRHSLANGRSTTTCPSGGNCVIAKNSRGRCQQCRYKKCLEVGM

CRVCGGRSSGFHFGALTCEGCKGFFRRTEETASRLVCVGGKDNCTITPRSRNVCKSCRFRRCLRAGM

**>Hm2DBDgb** (scaffold:HMN_v3:HMN_02_pilon:25725038:25740469)

CQVCGQPSVGFHHRAYVCEACKKFFTRHLTNRIKKDKASVCGDSLVIGDSLKSISMGGGNSDLYADLNVVCPMGGNCKIEGPGRGKCPHCRFRKCLDLGM

CRVCGGPSSGFHFGALTCEGCKGFFRRTVNSSSIPQCLGNQTCRITPSNRNMCKSCRFKKCLEVGM

**>Hm2DBDg** (scaffold:HMN_v3:HMN_03_pilon:16810204:16824059)

CAICGDVSAGFHCNAFVCEACKKFFIRSSKNENFAKYSCSKVNACEVSKDTRTHCQRCRFYKCLSIGM

CRVCGAQSSGFHFGAITCEGCKGFFRRTISERDNQRYTCRNGGTCLVTLATRNNCKSCRYRKCLSVGM

**>HmOther** (scaffold:HMN_v3:HMN_01_pilon:1:43032016)

CAVCGEANSKKYYGQYTCEACKAFFQQTIMGNLLYERRCNGQCTVDVKTRHRCQYCRLQKCLQVGI

**12. NRs in *Hymenolepis nana* (14)(** **prjeb508)**

**>HnTR** (scaffold:H_nana_Japan_v1_5_4:HNAJ_contig0001371)

CVVCGDNATGFHYRAMTCEGCKGFFRRTIQKKLTYTCKFNNNCNVMHKQNRNSCQKCRFDKCIAGGM

**>HnE78** (WBPS:Hymenolepis_nana_prjeb508:HNAJ_0000981901-mRNA-1)

CMVCGDKASGYHYGVISCEGCKGFFRRSIQKQIEYKCLRDGNCLVVRLNRNRCQYCRFKKCLKVGM

**>HnHR96a** (scaffold:H_nana_Japan_v1_5_4:HNAJ_scaffold0000909)

CVVCEEPASGYNFDRLTCESCKAFFRRNALKPKEKIKACSRNGDCVIVGNQRKHCPSCRLEKCLAVGM

**>HnHR96b** (scaffold:H_nana_Japan_v1_5_4:HNAJ_contig0000460)

CRVCGDRAVNHNFGQLTCESCKAFFRRNAHKALPSISAVLQSSGTKSITMEVYIKAIQHGLRDLTCTSKSGEHVVSPSTRRECPACRLKRCFLIGM

**>HnHR96c** (scaffold:H_nana_Japan_v1_5_4:HNAJ_scaffold0000055)

CAVCGDHAFGFNFGAIACESCKAFFRRNALRASMPPCLFNDSCMIQVKTRRFCSPCRLSKCFAVGM

**>HnNR1b** (scaffold:H_nana_Japan_v1_5_4:HNAJ_scaffold0000886)

CLVCGDIASGIHYGVLSCEGCKGFFRRALQDKRAHLRKCIRQDCCKISIKTRNHCQPCRLRRCLELGM

**>HnTLL** (scaffold:H_nana_Japan_v1_5_4:HNAJ_scaffold0000465)

CKVCKDHSSGKHYGIYACDCAGFFKRSIRRNRQYTCKSKNGQDSSCRVDKPHRNQCRACRLKKCLEAGM

**>HnTR4** (scaffold:H_nana_Japan_v1_5_4:HNAJ_contig0006142)

GRHYGVISCEGCKGFFKRSIRGHVNYVCRSNRKCVVNKAYRNRCQYCRMQKCLDAGM

**>HnHNF4** (scaffold:H_nana_Japan_v1_5_4:HNAJ_contig0001913)

CLICGDKATGKHYGAYSCDGCKGFFRRSVRRKHTYSCRYNRTCTIDKDMRNQCRFCRLKKCFRVGM

**>HnNR4A** (scaffold:H_nana_Japan_v1_5_4:HNAJ_scaffold0002453)

CLVCGDNAACQHYGVRTCEGCKGFFKRTIQKNAHYVCLQTKNCIVDKRRRNRCQYCRFQKCLKVGM

**>HnFTZ-F1b** (scaffold:H_nana_Japan_v1_5_4:HNAJ_scaffold0001980)

CPICGDKISGYHYGIFCCESCKGFFKRTVQNSKRYACHMATPASLCEITATSRKKCPACRFVKCLEKGM

**>Hn2DBDa** (scaffold:H_nana_Japan_v1_5_4:HNAJ_scaffold0001235)

CQICGELAAGFHHGAYVCEACKKFFMRHSLANGRSTTTCPSGGNCVIAKNSRGRCQQCRYKKCLEVGM

CRVCGGRSSGFHFGALTCEGCKGFFRRTEETASRLVCVGGKDNCTITPRSRNVCKSCRFRRCLRAGM

**>Hn2DBDb (VDN97648.1, VDO16679.1)**

CQVCGQPSVGFHHRAYVCEACKKFFTRHLTNRIKKDKASACGDSLGIEDPLKSISMGGGNSDMYADLNVVCPMGGNCKIEGPGRGKCPHCRFRKCLDLGM

CRVCGGPSSGFHFGALTCEGCKGFFRRTVNSSSIHQCLGNQTCRITPSNRNMCKSCRFKKCLEVGM

**>Hn2DBDg** (scaffold:H_nana_Japan_v1_5_4:HNAJ_scaffold0000436)

CAICGDVSAGFHCNAFVCEACKKFFIRSSKNENFAKYSCSKVNACEVSKDTRTHCQRCRFYKCLSIGM

CRVCGAQSSGFHFGAITCEGCKGFFRRTISERDNQRYTCRNGGTCLVTLATRNNCKSCRYRKCLSVGM

**13. NRs in *Macrostomum_lignano* (61) (prjna371498, prjna284736)**

**>MlTRa** (prjna371498_ scaffold:Mlig_3_7:scaf390)

CIVCGDAATGYHYRCMTCEGCKGFFRRTIQKSLQYHCKWSGSCQIDKNTRNQCQQCRFAKCLAVGM

**>MlTRb** (prjna371498_ scaffold:Mlig_3_7:scaf73)

CIVCNDAATGYHYRCMTCEGCKGFFRRTIQKNLQYHCKWNESCQIDKNTRNQCQQCRFRKCLAMGM

**>MlE78a** (prjna371498_ scaffold:Mlig_3_7:scaf2913)

CKVCGDKASGYHYGVISCEGCKGFFRRSIQKQIQYKCLRDGRCPVIRVNRNRCQHCRFRKCLSAGM

**>MlE78b** (prjna371498_ scaffold:Mlig_3_7:scaf1687)

CKVCGDKASGYHYGVVSCEGCKGFFRRSIQKQIQYRCLRDGACQVSCFRRNRCQHCRFRKCLAAGM

**>MlHR96-1** (prjna371498_ scaffold:Mlig_3_7:scaf2848)

CHVCGDKALGYNFDAVTCESCKAFFRRNALKKKEQKCNFSGACDVSVNTRKFCSACRLKKCFSVGM

**>MlHR96-2** (prjna371498_ scaffold:Mlig_3_7:scaf528)

CSVCGDKALGFNFDAVTCESCKAFFRRNAVKERVRRCNFDNNCEVTVATRKFCPACRLSKCFRVGM

**>MlHR96-3** (prjna371498_ scaffold:Mlig_3_7:scaf1889)

CTVCGDKALGYNFDAITCESCKAFFRRNALKTKIQKCNFESSCLVSVITRKFCPACRLAKCFKVGM

**>MlHR96-4** (prjna371498_ scaffold:Mlig_3_7:scaf62)

CGVCGDKALGFNFDAVSCESCKAFFRRNAVKGVEAFKCPYSGACSIDVSNRRFCKRCRLAKCFDIGM

**>MlHR96-5** (prjna371498_ scaffold:Mlig_3_7:scaf1047)

CTVCGDLAVNYNFGRVCCESCKHFFRRNSAKTL

**>MlHR96-6** (prjna371498_ scaffold:Mlig_3_7:scaf208)

CGVCGDKALGFNFDAVSCESCKAFFRRNAAKGVEALKCPYEGNCRIDVSNRRFCKRCRLRKCFDVGM

**>MlHR96-7** (prjna371498_ scaffold:Mlig_3_7:scaf3030)

CRVCGDKALGFNFDAISCESCKAFFRRNALKQDVPACMFTQNCSVTVATRRFCTHCRLQKCIQVGM

**>MlHR96-8** (prjna371498_ scaffold:Mlig_3_7:scaf2506)

CTICGDKALGYNFDAISCESCKAFFRRNALKAEIPDCMFSQRCNVTVATRRFCTSCRLKKCLECGM

**>MlHR96-9** (prjna371498_ scaffold:Mlig_3_7:scaf1320)

CAVCGDPASGYNFDRLTCESCKAFFRRNALKAREKIKPCSRGGGCDVSGSQRKHCPTCRLEKCVRVGM

**>MlHR96-10** (prjna371498_ scaffold:Mlig_3_7:scaf2678)

CAVCAEPASGYNFDRLTCESCKAFFRRNALKSREKIKPCSRGGGCDVSGSQRKHCPTCRLEKCLLVGM

**>MlHR96-11** (prjna371498_ scaffold:Mlig_3_7:scaf1335)

CAVCSEPATGYNFDRLTCESCKAFFRRNALKSRERIKPCSRGGGCDVSGPQRKHCPTCRLEKCLRVGM

**>MlHR96-12** (prjna371498_ scaffold:Mlig_3_7:scaf104)

CRVCGDLAATFNFGQICCESCKAFFRRNAEKHLDCSHGDGNCLITVTTRRTCRACRFEKCLAVGM

**>MlHR96-13** (prjna371498_ scaffold:Mlig_3_7:scaf416)

CLVCGCKAENYNFGVISCESCKAFFRRNAHKQTLGEAGFACSFRRGGCEVSLATRKKCPGCRLAKCFAVGM

**>MlHR96-14** (prjna371498_ scaffold:Mlig_3_7:scaf2467)

CLVCGAKAENYNFGVISCESCKAFFRRNAHKKTLGDAGFSCSFQHARCEVTLATRKKCPGCRLAKCFRVGM

**>MlHR96-15** (scaffold:Mlig_3_7:scaf31)

CHVCGDKALGFNFDAVTCESCKAFFRRNAFKNMDMKCNFSGSCSISINTRKFCSPCRLKKCFLIGM

**>MlHR96-16** (prjna371498_ scaffold:Mlig_3_7:scaf197)

CGVCGDKALGFNFDAVSCESCKAFFRRNAGRGVSAFKCPYEGHCVMDVSNRRFCKHCRLRKCFDVGM

**>MlNR1a** (prjna371498_ scaffold:Mlig_3_7:scaf1238)

CVVCGDKSSGYHYGVSSCEGCKGFFRRSIQKSLVYSCQKSDQCEVTRQTRNRCQSCRLRKCIAGGM

**>MlHNF4** (prjna371498_ scaffold:Mlig_3_7:scaf1621)

CGICSDRATGKHYGAYSCDGCKGFFRRSVRKSHQYICRFNRNCTVDRDKRNQCRYCRLDKCFRAGM

**>MlRXR1** (prjna371498_ scaffold:Mlig_3_7:scaf5054)

CAICGDRASGKHYSVYSCEGCKGFFKRTVRKDLKYTCRDERRCCIDKRQRNRCQYCRYMKCLAMGM

**>MlRXR2** (prjna371498_ scaffold:Mlig_3_7:scaf3591)

CSICGDRATGRHYSAYSCEGCKGFFKRTVRKSLRYTCRDSRRCRIDKRQRNRCQYCRYMKCLAAGM

**>MlTR4a** (prjna371498_ scaffold:Mlig_3_7:scaf1351)

CRVCGDRASGRHYGVVSCEGCKGFFKRSIRRKVNYACRSGKQCLVNKAYRNRCQFCRLQKCISTGM

**>MlTR4b** (prjna371498_ scaffold:Mlig_3_7:scaf675)

CRVCGDRASGRHYGVVSCEGCKGFFKRSIRRRVNYSCRSGRQCLVNKTFRNRCQFCRLRKCIGVGM

**>MlPNR** (prjna371498_ scaffold:Mlig_3_7:scaf306)

CVVCGDVSSGKHYGILACNGCSGFFKRSVRRKLIYRCQAGTGTCIIDKAHRNQCQACRLKKCLQMGM

**>MlDSF** (prjna371498_ scaffold:Mlig_3_7:scaf1834)

CKVCGDRSSGKHYGVYSCDGCSGFFKRSIHKGRAYACKAQGDGRDRCPVDKTHRNQCRACRLHKCFEARM

**>MlTLL** (prjna371498_ scaffold:Mlig_3_7:scaf149)

CRVCRDHSSGKHYGIYACDGCAGFFKRSIRRNRQYVCKSRGHGECVIDKTHRNQCRACRLKKCVEAGM

**>Mlfax1** (prjna284736_ scaffold:ML2:uti_cns_0004952)

CLICGDKSYGKHYGVFCCDGCSCFFKRSIRKRMSYTCISGKGTCLIDKARRNWCPFCRLQKCFAVSM

**>MlNR236a** (prjna371498_ scaffold:Mlig_3_7:scaf1501)

CLVCGDRASGKHYGVLSCDGCRGFFKRSVRRSLEYVCKESGDCPVDVARRNQCQACRYRKCLMVRM

**>MlNR236b** (prjna371498_ scaffold:Mlig_3_7:scaf2772)

CLVCGDRASGKHYGVLSCDGCRGFFKRSVRRSLEYVCKEANACPVDVARRNQCQACRYKKCLQVKM

**>MlCoup-TFI** (prjna371498_ scaffold:Mlig_3_7:scaf2996)

CVVCGDKSSGKHYGQYTCEGCKSFFKRSVRRKLSYTCRGNRNCPVDIHHRNQCQYCRFQKCLKSGM

**>MlCoup-TFII** (prjna371498_ scaffold:Mlig_3_7:scaf197)

CVVCGDRASGKHYGQFSCEGCKSFFKRSVRRNLVYSCRGGTPCPVDTQHRNQCQRCRFDKCLALGM

**>MlERR1** (prjna371498_ scaffold:Mlig_3_7:scaf526)

CRVCHRRASGHHYGALTCEACKAFFKRTVNSCLTYFCPVQRSGSRCPVRVPNKRQCPACRWKRCLEASM

**>MlERR2** (prjna284736_ scaffold:ML2:uti_cns_0046130)

CSICFRKATGHHYGAVTCEACKAFFKRTVHSCVTYFCPVQRSGSRCPVPKSSRKRHCPACRWVRCLEARM

**>MlERR3** (prjna371498_ scaffold:Mlig_3_7:scaf1769)

CQVCLNFPQTGRHYGAVSCEACKAFFKRTVQQCLVYQCLASPVLCTELSSKRRSCPKCRFQRCLKAGM

**>MlERR4** (prjna371498_ scaffold:Mlig_3_7:scaf13)

CSICGAPPSGVHYGATTCEACKAFFKRTVHSCLTYSCIRPGNPGQCPVHGNRRRQCAACRWRRCLTVGM

**>MlNR4Aa** (prjna371498_ scaffold:Mlig_3_7:scaf2828)

CLVCGDTAACQHYGVRTCEGCKGFFKRTIQKNAQYVCLHAKNCIVDKRRRNRCQYCRFKKCLSVGM

**>MlNR4Ab** (prjna371498_ scaffold:Mlig_3_7:scaf1702)

CLVCGDHASCQHYGVRTCEGCKGFFKRTIQKNAQYTCQHNNDCVIDKKRRNRCQHCRFRKCLKVGM

**>MlFTZ-TF1b** (prjna371498_ scaffold:Mlig_3_7:scaf303)

CPVCGDKISGYHYGIFSCESCKGFFKRTVQNRKTYVCHRGSQSACEMSLLSRKKCPACRMRKCLEKGM

**>MlNR6** (prjna371498_ scaffold:Mlig_3_7:scaf1573)

CAVCEDRATGLHYGVMTCEGCKGFFKRTVQNRRIYECGFGQGCDVQLQRRNNCQFCRFRKCLDTGM

**>Ml2DBD-1** (prjna371498_ scaffold:Mlig_3_7:scaf155)

CDVCGDTAAGFHCGAFVCEACKKFFIRSTRAGMQGGLGIKYSCSKVGRCDVTKETRTHCQHCRYKKCLMLNM

CRVCGASSSGFHFGAITCEGCKGFFRRTIKERDAGKYICSKGGGCEINQQSRNACKSCRFAKCIRAGM

**>Ml2DBD-2** (prjna371498_ scaffold:Mlig_3_7:scaf3006)

CDVCGDTAAGFHCGAFVCEACKKFFIRSSRAEQQQQLLLLHHGDSAVSSAGGPSGAVKYACSKSGSCDITKETRTHCQFCRYKKCLALRM

CRVCGASSSGFHFGAITCEGCKGFFRRTIKERDSGKYVCSKGGGCEINKSSRNACKSCRFTKCIRAGM

**>MlNR2-other** (prjna371498_ scaffold:Mlig_3_7:scaf2042)

CRVCNDISSGIHYGIEACEGCKGFFKRSIQTPRDYKCAREGTCEINKTSRNVCQYCRLTKCLNAGM

**>MlUn1** (prjna371498_ scaffold scaffold:Mlig_3_7)

CSVCGCDRPAGCHYGAVTCHACKSFFKRTVQHSLAYACMRLARHCALVSERTRACGYCRFRKCLRVGM

**>MlUn2** (prjna371498_ scaffold:Mlig_3_7:scaf2443)

CAVCRCTWPAGSHYGAVTCHACKSFFKRTVQLCLTYSCIRKPHHCSLASERNRGCGACRFRRCLQVGM

**>MlUn3** (prjna371498_ scaffold:Mlig_3_7:scaf759)

CDVCRCTWPAGMTCHACKSFFKRTVQLCLTYRCMQNPRHCSLTFERNRGCGACRFCRCLQVGM

**>MlUn4** (prjna371498_ scaffold:Mlig_3_7:scaf291)

CSVCQCPFPAGNHYGAVTCHACKSFFKRTVQLSLSYNCMRRPRHCSLSSERSRSCGFCRFKKCLEVGM

**>MlUn5** (prjna371498_ scaffold:Mlig_3_7:scaf2137)

CRICGDRGAGAHYGVVTCVACKSFFKRTVQQCLTYRCEHNGSCRIHQATRRLCQACRFQRCLRCGM

**>MlUn6** (prjna284736_ scaffold:ML2:uti_cns_0046130)

CSVCDLRATGVHYGAATCNACKTFFKRTVHSCLTYQCARRLSGASDDQLCPVREPNRKQCPACRWRRCLRANM

**>MlUn7** (prjna284736_ scaffold:ML2:unitig_24121)

CAVCGLTATGVHYGAATCNACKAFFKRTVHSCLTYLCASSTDGQYQPFLQDPKQQSPLCPVNNASRKQCPACRWRRCLMSGM

**>MlUn8** (prjna371498_ scaffold:Mlig_3_7:scaf2717)

CQVCGTGANVRKNFGVPSCVSCRIFYSRNTGKQLVCYQNGNCRIVGPSRKSCAACRFQKCLLAGM

**>MlUn9** (prjna371498_ scaffold:Mlig_3_7:scaf94)

CRICGSTTNVRKNFGVPSCVSCRIFYSRNASKQLVCYQSGNCRIEGASRKSCAACRFDKCTRAGM

**>MlUn10** (prjna371498_ scaffold:Mlig_3_7:scaf1177)

CVVCGDYASGFHYSALTCEACKSFFKRTVQDKHEYTCSGNFDCSVSKSMRKSCQYCRFQKCLVNG

**>MlUn11** (prjna371498_ scaffold:Mlig_3_7:scaf3415)

CSVCGDFASGFHYSALTCEACKSFFKRTVQDKHEYTCSGNFDCAVSKSMRKSCQYCRFQKCLRNGM

**>MlUn12** (prjna371498_ scaffold:Mlig_3_7:scaf4845)

CVVCEDEASGWHYGAVTCDSCKSFFERSVKDRHIYDCHFNRDCDLRLQRRNHCQGCRWDRCLDAGM

**>MlUn13** (prjna284736_ scaffold:ML2:uti_cns_0001803)

CSVCGTNRRVNLHYGVHACESCRNFFKRALANNQQSSVSDVGGSLSPPTRLVCVRGGNQCDVTPNNRSDCKACRLRKCLAVGM

**>MlUn14** (prjna284736_ scaffold:ML2:uti_cns_0005374)

CLVCDLLASGKNFGVATCESCKTFFRRNAKRDTSQLRCLHQNSCNISKEYRRSCTSCRLNKCFAVGM

**>MlUn15** (prjna284736_ scaffold:ML2:uti_cns_0002862)

CCVCELAASGNNFGVATCESCKTFFRRNAKRDPSQIKCLHQNNCNILREYRRSCTACRLRKCFAVGM

**>MlUn16** (prjna284736_ scaffold:ML2:uti_cns_0006443)

CLVCQLAASGNNFGVATCESCKTFFRRNAKREPSQIRCLHYNNCNVLREYRRSCTACRLRKCFAVGM

**14. NRs in *Mesocestoides corti* (21)(** **prjeb510)**

**>McTRa** (scaffold:M_corti_Specht_Voge_v1_0_4:MCOS_contig0001096)

CVVCGDNATGFHYRAMTCEGCKGFFRRSVQKKLKYTCKFNGQCFVGNKQNRNSCQKCRFDRCLKAGM

**>McTRb** (scaffold:M_corti_Specht_Voge_v1_0_4:MCOS_scaffold0000321)

CVVCGDNATGFHYRAMTCEGCKGFFRRSVQKKLEYTCKFNGQCSVGDKQNRNSCQKCRFERCLKGGM

**>McE78** (scaffold:M_corti_Specht_Voge_v1_0_4:MCOS_contig0000103)

CRVCGDKASGYHYGVISCEGCKGFFRRSIQKQIEYKCLRDGKCTVTRMNRNRCQYCRFRLCIDRGM

**>McHR96a** (scaffold:M_corti_Specht_Voge_v1_0_4:MCOS_contig0000017)

CVVCGEPASGYNFDRLTCESCKAFFRRNALKPKEKIKACGRNGDCNIEGSQRKHCPSCRLEKCLAVGM

**>McHR96b** (scaffold:M_corti_Specht_Voge_v1_0_4:MCOS_scaffold0000214)

CRVCGDRAVNHNFGQLTCESCKAFFRRNAHKALPSINAALQSCAPGMPTSDAYVRAVRLGLSELTCTSKSGEHVVSPSTRRECPACRLKRCFLIGM

**>McNR96c** (scaffold:M_corti_Specht_Voge_v1_0_4:MCOS_contig0000141)

CAVCGDHAVGFNFGAIACESCKAFFRRNALRVSMPPCLFNANCLIQVKTRRFCSPCRLAKCFAVGM

**>McNR1b** (scaffold:M_corti_Specht_Voge_v1_0_4:MCOS_scaffold0000051)

CLVCGDTASGIHYGVLSCEGCKGFFRRALQDKRAHLRKCIRQDCCKISLKTRNHCQPCRLRRCLELGM

**>McNR1a** (scaffold:M_corti_Specht_Voge_v1_0_4:MCOS_contig0000225)

CAICGDVATGVHHGVPACEGCKGFFRRSVMRKTPYTCPRNKRCEITPTQRNKCQYCRFVKCTQAGM

**>McHNF4** (scaffold:M_corti_Specht_Voge_v1_0_4:MCOS_contig0000470)

GKHYGAYSCDGCKGFFRRSVRRKHTYSCRYNRACTIDKDMRNQCRFCRLKKCFRVGM

**>McTR4** (scaffold:M_corti_Specht_Voge_v1_0_4:MCOS_scaffold0000091)

CLVCGDVASGRHYGVISCEGCKGFFKRSIRGHVNYVCRSNKHCVVNKAFRNRCQYCRMQKCLMVGM

**>McTLL** (scaffold:M_corti_Specht_Voge_v1_0_4:MCOS_scaffold0000372)

CKVCKDHSSGKHYGIYACDGCAGFFKRSIRRNRQYTCKSRNGSESGCRVDKPHRNQCRACRLKKCLEAGM

**>McNR4A** (scaffold:M_corti_Specht_Voge_v1_0_4:MCOS_scaffold0000146)

CLVCGDNAACQHYGVRTCEGCKGFFKRTIQKNAHYVCLQTKNCIVDKRRRNRCQYCRFQKCLKVGM

**>McFTZ-F1b** (scaffold:M_corti_Specht_Voge_v1_0_4:MCOS_contig0000464)

CPICGDKISGYHYGIFCCESCKGFFKRTVQNAKRYACHMATPSSLCDITATSRKKCPACRFVKCLEKGM

**>Mc2DBDa** (scaffold:M_corti_Specht_Voge_v1_0_4:MCOS_contig0000008)

CQICGELAAGFHHGAYVCEACKKFFMRHSLANGRATTTCPSGGKCVIAKTSRGRCQQCRYKKCLEVGM

CRVCGGRSSGFHFGALTCEGCKGFFRRTEEAAGRLVCVGGKDNCTITPRSRNVCKACRFRRCLRAGM

**>Mc2DBDb** (scaffold:M_corti_Specht_Voge_v1_0_4:MCOS_contig0000673)

CQVCGQPSVGFHHRAYVCEACKKFFTRHLTNRIKKDKPATADGEPSKPTHGDVYADLSVACPMGGNCKIEGPGRGKCPHCRFRKCLDLGM

CRVCGGPSSGFHFGALTCEGCKGFFRRTVHSSSIPQCLGNQTCRITPSNRNMCKSCRFKKCLEVGM

**>Mc2DBDg** (scaffold:M_corti_Specht_Voge_v1_0_4:MCOS_contig0000021)

CDICGDVSAGFHCNAYVCEACKKFFIRSSKNENYTKYSCSKVNACEVNKDTRTHCQRCRFYKCLRIGM

CRVCGAQSSGFHFGAITCEGCKFFRRTISERDNQRYTCRNGGTCIITLATRNNCKSCRYRKCLSVGM

**>McNR2-other** (scaffold:M_corti_Specht_Voge_v1_0_4:MCOS_contig0000268)

CVICGDLATGRHYGAVSCEGCKGFFKRSVRHRLRYICRAQRACPVDIKNRNLCRYCRFHRCIQMGM

**>Mcother-1** (scaffold:M_corti_Specht_Voge_v1_0_4:MCOS_contig0001226)

CVVCGDEASGIHYHALTCDSCKNFFWRSMQKKVEFMCDNDGQCFVGDKQSRSRCQKCRFDRCIESGM

**>Mcother-2** (scaffold:M_corti_Specht_Voge_v1_0_4:MCOS_scaffold0000321)

CVVCGDNATGFHYRAMICNGCMAFFRRLVQNKHQYTCLFNGQCSVDGKQNRNRCKKCRLELCLNRGM

**>Mcother-3** (scaffold:M_corti_Specht_Voge_v1_0_4:MCOS_scaffold0001152)

VCGDNAMGFHYHTMSCDGCKRFFCHSMQGKVGSTCQFYGQCYVVENKNRTRGKKCRLDRCLEIGV

**>McOther-4** (scaffold:M_corti_Specht_Voge_v1_0_4:MCOS_scaffold0001224)

CAICGASASGTHYGVLSCDGCRTFFGKSVRKGVQYVCPGNANCPIDRNQPLKCQYCRFKRCLEAGM

**15. NRs in *Opisthorchis felineus* (22)(** **prjna413383)**

**>OfTRa** (scaffold:ICG_Ofel_1.0:scaff_1637)

CVVCGDSATGFHYRAMTCEGCKGFFRRSVQKKLVYTCKFNGRCSVADKQNRNSCQKCRFDRCLSGGM

**>OfTRb** (scaffold:ICG_Ofel_1.0:scaff_456)

CVVCGDNATGFHYRAMTCEGCKGFFRRSIQKQLVYVCKFQGNCSVSDKHSRNSCQKCRFDRCIRGGM

**>OfE78** (scaffold:ICG_Ofel_1.0:scaff_1466)

CKVCGDKASGYHYGVISCEGCKGFFRRSIQKQIEYKCLRDGKCLVIRLNRNRCQYCRFRKCIAVGM

**>OfHR96a** (scaffold:ICG_Ofel_1.0:scaff_1726)

CIVCGEPASGYNFDRLTCESCKAFFRRNALKPKDKIKVCSRGGGCIIEGNQRKHCPSCRLEKCFAVGM

**>OfNR96b** (scaffold:ICG_Ofel_1.0:scaff_1799)

CKVCGDRAVNHNFGQLTCESCKAFFRRNAHKLPSLLDRTFECRSDIDMTTVQYSLSLSRSKYLVELTCTSKTGEHEITPSTRRECPACRLKKCFLVGM

**>OfHR96c** (scaffold:ICG_Ofel_1.0:scaff_1800)

CNVCGDAAMGFNFGAVTCESCKAFFRRTARKAQVANCVFNEKCTITVATRRFCSHCRLKKCFAAGM

**>OfNR1a** (scaffold:ICG_Ofel_1.0:scaff_1734)

CVVCGDKSTGAHYGVFTCEGCKGFFRRAVHRNQTYACARNGSCEVNRALRNKCQHCRFLKCLASGM

**>OfHNF4** (scaffold:ICG_Ofel_1.0:scaff_9)

CLICGDRATKHYGAYSCDGCKGFFRRSVRRNHSYTCRHKRACVITKDKRNQCRFCRLRKCFRVGM

**>OfRXR1** (scaffold:ICG_Ofel_1.0:scaff_61)

CVICGDRASGRHYGVVSCEGCKGFFKRTVRKQVQYVCRGSGDCPVDRRKRTRCQACRYDRCILKGM

**>OfRXR2** (scaffold:ICG_Ofel_1.0:scaff_158)

CQVCGDRACGKHYGVFSCEGCKGFFRRTVRRELVYTCRDSQSCQIDRRLRNRCQFCRYHKCLQVGM

**>OfTR4** (scaffold:ICG_Ofel_1.0:scaff_271)

CRVCGDKASGRHYGVVSCEGCKGFFKRSIRGHVSYVCRSDQNCLVNKAYRNRCQYCRLQKCLLVGM

**>OfTLL** (scaffold:ICG_Ofel_1.0:scaff_1636)

CRVCKDHSSGKHYGIYACDGCAGFFKRSIRRNRQYACKNRTANGTKLSTAVGGCRVDKSHRNQCRACRLKKCLEVGM

**>OfPNR** (scaffold:ICG_Ofel_1.0:scaff_62)

CSVCGDVSSGKHYGILACNGCSGFFKRSVRRKLIYRCQAGTGLCSIDKAHRNQCQACRLKKCLRMGM

**>OfDSF** (scaffold:ICG_Ofel_1.0:scaff_1794)

CGVCGDISSGKHYGIYSCDGCSGFFKRSIHKSRTYTCKAVGHLKGRCPIDRNRRNQCRACRLKKCFQAQM

**>OfCoup-TFI** (scaffold:ICG_Ofel_1.0:scaff_1277)

CVVCGDKSSGKHYGQHTCEGCKSFFKRSVRRKLTYTCRGTRQCPIDVHHRNQCQYCRFQKCVRAGM

**>OfCoup-TFII** (scaffold:ICG_Ofel_1.0:scaff_1376)

VCGDRSSGKHYGQFTCEGCKSFFKRSVRKSASYVCRSEGQCPVDAQRRNQCQACRMTRCLLAGM

**>OfNR4A** (scaffold:ICG_Ofel_1.0:scaff_412)

CLVCGDSAACQHYGVRTCEGCKGFFKRTIQKNAQYVCLQSKNCVVDKRRRNRCQYCRFQKCLKVGM

**>OfFTZ-F1a** (scaffold:ICG_Ofel_1.0:scaff_403)

CPICGDRVSGYHYGLPTCESCKGFFKRTVQNKKEYHCTEQGNCVIDRIHRKRCAHCRFQKCLAVGM

**>OfFTZ-F1b** (scaffold:ICG_Ofel_1.0:scaff_459)

CPICGDKISGYHYGIFCCESCKGFFKRTVQNAKRYACHHPSANSVCEINVSSRKKCPACRFLKCVDKGM

**>Of2DBDa** (scaffold:ICG_Ofel_1.0:scaff_4)

CQVCGELAAGFHHGAYVCEACK>KFFMRHSLTDTKPTNICPTGGNCVVAKGSRGKCQICRYRKCLFVGM

CRVCGGRSSGFHFGALTCEGCK>GFFRRTEGSAASLVCVGGQNTCTITPRSRNACKSCRFRRCIAAGM

**>Of2DBDb** (scaffold:ICG_Ofel_1.0:scaff_1490)

CQICGQPAVGFHHRAYVCEACKKFFMRHTAARYRHAEAGGGNLSDSACPMGGQCRVEGPGRGKCPHCRYRKCLDLGM

CRVCGGPSSGFHFGALTCEGCKGFFRRTVLSNVRLECLSNNDCPITPANRNMCKSCRFQRCLAVGM

**>Of2DBDg** (scaffold:ICG_Ofel_1.0:scaff_439)

CDICGDVSAGFHCNAYVCEACKKFFIRSSKGDNFSKYTCTKSNACEINKDTRTHCQRCRYQKCLRLGM

CRVCGAKSSGFHFGAITCEGCKGFFRRTINERESQRYTCRNGGNCAVTGATRNNCKSCRYRRCVAVGM

**16. NRs in *Opisthorchis viverrini* (22)(** **prjna222628)**

**>OvTRa** (scaffold:OpiViv1.0:opera_v5_111)

CVVCGDSATGFHYRAMTCEGCKGFFRRSVQKKLVYTCKFNGRCSVADKQNRNSCQKCRFDRCLSGGM

**>OvTRb** (scaffold:OpiViv1.0:scaffold4102)

CVVCGDNATGFHYRAMTCEGCKGFFRRSIQKQLVYVCKFQGNCSVSDKHSRNSCQKCRFDRCIRGGM

**>OvE78** (scaffold:OpiViv1.0:opera_v5_15)

CKVCGDKASGYHYGVISCEGCKGFFRRSIQKQIEYKCLRDGKCLVIRLNRNRCQYCRFRKCIAVGM

**>OvHR96a** (scaffold:OpiViv1.0:opera_v5_2)

CIVCGEPASGYNFDRLTCESCKAFFRRNALKPKDKIKVCSRGGGCIIEGNQRKHCPSCRLEKCFAVGM

**>OvHR96b** (scaffold:OpiViv1.0:opera_v5_296)

CKVCGDRAVNHNFGQLTCESCKAFFRRNAHKLLPSLLDRTFECRSDVDMTTVQYSLSLSRSKYLVELTCTSKTGEHEITPSTRRECPACRLKKCFLVGM

**>OvHR96c** (scaffold:OpiViv1.0:opera_v5_3)

CNVCGDAAMGFNFGAVTCESCKAFFRRTARKAQVANCVFNEKCTITVATRRFCSHCRLKKCFAAGM

**>OvNR1a** (scaffold:OpiViv1.0:opera_v5_64)

CVVCGDKSTGAHYGVFTCEGCKGFFRRAVHRNQTYACARNGSCEVNRALRNKCQHCRFLKCLASGM

**>OvTR4** (scaffold:OpiViv1.0:opera_v5_4)

CRVCGDKASGRHYGVVSCEGCKGFFKRSIRGHVSYVCRSDQNCLVNKAYRNRCQYCRLQKCLLVGM

**>OvRXR1** (scaffold:OpiViv1.0:opera_v5_45)

CVICGDRASGRHYGVVSCEGCKGFFKRTVRKQVQYVCRGSGDCPVDRRKRTRCQACRYDRCILKGM

**>OvRXR2** (scaffold:OpiViv1.0:opera_v5_102)

CQICGDRACGKHYGVFSCEGCKGFFRRTVRRELVYTCRDSQSCQIDRRLRNRCQFCRYHKCLQVGM

**>OvCoup-TFI** (scaffold:OpiViv1.0:opera_v5_82)

CVVCGDKSSGKHYGQHTCEGCKSFFKRSVRRKLTYTCRGTRQCPIDVHHRNQCQYCRFQKCVRAGM

**>OvCoup-TFII** (scaffold:OpiViv1.0:opera_v5_170)

CQVCGDRSSGKHYGQFTCEGCKSFFKRSVRKSASYVCRSEGQCPVDAQRRNQCQACRMTRCLLAGM

**>OvPNR** (scaffold:OpiViv1.0:opera_v5_378)

CSVCGDVSSGKHYGILACNGCSGFFKRSVRRKLIYRCQAGTGLCSIDKAHRNQCQACRLKKCLRMGM

**>OvHNF4** (scaffold:OpiViv1.0:opera_v5_56)

CLICGDRATGKHYGAYSCDGCKGFFRRSVRRNHSYTCRHKRACVITKDKRNQCRFCRLRKCFRVGM

**>OvDSF** (scaffold:OpiViv1.0:opera_v5_84)

CGVCGDISSGKHYGIYSCDGCSGFFKRSIHKSRTYTCKAVGHLKGRCPIDRNRRNQCRACRLKKCFQAQM

**>OvTLL** (scaffold:OpiViv1.0:opera_v5_373)

CRVCKDHSSGKHYGIYACDGcagffRSIRRNRQYACKNRTANGTKLSTAVGGCRVDKSHRNQCRACRLKKCLEVGM

**>OvNR4A** (scaffold:OpiViv1.0:opera_v5_50)

CLVCGDSAACQHYGVRTCEGCKGFFKRTIQKNAQYVCLQSKNCVVDKRRRNRCQYCRFQKCLKVGM

**>OvFTZ1-F1a** (scaffold:OpiViv1.0:opera_v5_162)

CPICGDRVSGYHYGLPTCESCKGFFKRTVQNKKEYHCTEHGNCVIDRIHRKRCAHCRFQKCLAVGM

**>OvFTZ-F1b** (scaffold:OpiViv1.0:opera_v5_52)

CPICGDKISGYHYGIFCCESCKGFFKRTVQNAKRYACHHPSANSVCEINVSSRKKCPACRFLKCVDKGM

**>Ov2DBDa** (scaffold:OpiViv1.0:opera_v5_68)

CQVCGELAAGFHHGAYVCEACKKFFMRHSLTDTKPTNICPTGGNCVVAKGSRGKCQICRYRKCLFVGM

CRVCGGRSSGFHFGALTCEGCKGFFRRTEGSAASLVCVGGQNTCTITPRSRNACKSCRFRRCIAAGM

**>Ov2DBDb** (scaffold:OpiViv1.0:opera_v5_448)

CQICGQPAVGFHHRAYVCEACKKFFMRHTAARYRHAEAGGGNLSDSACPMGGQCRVEGPGRGKCPHCRYRKCLDLGM

CRVCGGPSSGFHFGALTCEGCKGFFRRTVLSNVRLECLSNNDCPITPANRNMCKSCRFQRCLAVGM

**>Ov2DBDg** (scaffold:OpiViv1.0:opera_v5_36)

CDICGDVSAGFHCNAYVCEACKKFFIRSSKGDNFSKYTCTKSNACEINKDTRTHCQRCRYQKCLRLGM

CRVCGAKSSGFHFGAITCEGCKGFFRRTINERESQRYTCRNGGNCAVTGATRNNCKSCRYRRCVAVGM

**17. NRs in *Protopolystoma xenopodis* (23)(** **prjeb1201)**

**>PxTR** (scaffold:P_xenopodis_South_Africa_v1_0_4:PXEA_contig0003395)

CVVCGDKATGFHYRAMTCEGCKGFFRRSVQKRLVYTCKFNGRCSVSDKQNRNSCQKCRFDRCIKGGM

**>PxE78** (scaffold:P_xenopodis_South_Africa_v1_0_4:PXEA_scaffold0003552, scaffold:P_xenopodis_South_Africa_v1_0_4:PXEA_contig0010780)

CKVCGDKASGYHYGVISCEGCKGFFRRSIQKQIEYKCLREGKCLVIRLNRNRCQFCRFKKCLAVGM

**>PxNR1a** (scaffold:P_xenopodis_South_Africa_v1_0_4:PXEA_scaffold0020781, PXEA_contig0133764)

CRVCQDKASGFHYGVATCEGCKGFFRRSIQRRLSYFCSKNGGCEISRTTRNKCQYCRLERCFLVGM

**>PxNR1b** (scaffold:P_xenopodis_South_Africa_v1_0_4:PXEA_contig0016372, PXEA_scaffold0022783)

CMVCGDLASGVHYGVPSCEGCKGFFRRSLQDIGNQARQCTNNRRCLITVATRNRCQYCRLRRCLDLGM

**>PxHR96a** (scaffold scaffold:P_xenopodis_South_Africa_v1_0_4:PXEA_contig0032602, PXEA_contig0118910)

CIVCGEPASGYNFDRLTCESCKAFFRRNALKPRDKIKACSRGGGCNVEGGQRKHCPSCRLEKCLASGM

**>PxHR96b** (scaffold:P_xenopodis_South_Africa_v1_0_4:PXEA_contig0016195, PXEA_contig0023201)

CKVCGDKAVNHNFGQLTCESCKAFFRRNAHKELTCTSKVTGHVISPTTRRECPACRLKRCFVVGM

**>PxHR96c** (scaffold:P_xenopodis_South_Africa_v1_0_4:PXEA_scaffold0008982)

CSICGDKAVGYNFGAIACESCKAFFRRNALKTETPVCIFDRSCDIRTATRRFCSACRLAKCLKVGM

**>PxRXR1** (scaffold:P_xenopodis_South_Africa_v1_0_4:PXEA_contig0041553)

CMICGDHSTGRHYGVVSCEGCKGFFKRTVRRQLVYVCREVGNCLIDKKKRTRCQHCRFQKCLKAGM

**>PxRXR2** (scaffold:P_xenopodis_South_Africa_v1_0_4:PXEA_scaffold0005591, scaffold:P_xenopodis_South_Africa_v1_0_4:PXEA_contig0041993)

CEICADKASGKHYGVYSCEGCKGFFKRTVRKEIQYVCRGDNQCRIDKNSRNKCQSCRLRLCYAVGM

**>PxCoup-TFI** (scaffold:P_xenopodis_South_Africa_v1_0_4:PXEA_contig0015243)

CVVCGDKSSGKHYGQYTCEGCKSFFKRSVRRKLTYTCRGNRQCPIDVHHRNQCQYCRFQKCLQTGM

**>PxCoup-TFII** (scaffold:P_xenopodis_South_Africa_v1_0_4:PXEA_contig0055617)

VCGDRSSGKHYGQYTCEGCKSFFKRSVRKAAAYVCRAGGRCPVDAQRRNQCQACRMARCLAAGM

**>PxDSF** (scaffold:P_xenopodis_South_Africa_v1_0_4:PXEA_contig0013592)

CRVCGDQASGKHYGIYSCDGCSGFFKRSIHKNRSYTCKALGKLKDRCPVDKTHRNQCRACRLKRCFLAQM

**>PxTLL** (scaffold:P_xenopodis_South_Africa_v1_0_4:PXEA_contig0016679)

CRVCKDHSSGKHYGIYACDGCAGFFKRSIRRNRQYGCKARGAPGNMCRIDKSHRNQCRACRLKKCLEAGM

**>PxNHR236** (scaffold:P_xenopodis_South_Africa_v1_0_4:PXEA_scaffold0002890)

CQVCGDRASGRHYGVVSCDGCRGFFKRSVRRDTVYACKEIGSCPVDLIRRNQCQACRFAKCLAVNM

**>PxPNR** (scaffold:P_xenopodis_South_Africa_v1_0_4:PXEA_scaffold0008963)

CVVCGDVSSGKHYGILACNGCSGFFKRSVRRKLIYRCQAGTGYCVIDKAHRNQCQACRLKKCLQMGM

**>PxTR4** (scaffold:P_xenopodis_South_Africa_v1_0_4:PXEA_scaffold0027139)

GRHYGVISCEGCKGFFKRSIRGHVHYACRGDQNCIVNKAYRNRCQFCRLQKCLFVGM

**>PxHNF4** (scaffold:P_xenopodis_South_Africa_v1_0_4:PXEA_contig0006820)

GKHYGAFSCDGCKGFFRRSVRRNHEYVCRFSRSCTITRDRRNQCRCCRLRRCFLMGM

**>PxNR4A** (scaffold:P_xenopodis_South_Africa_v1_0_4:PXEA_scaffold0036509, PXEA_scaffold0039091)

CLVCGDTAACQHYGVRTCEGCKGFFKRTIQKNAQYVCLQSKNCIVDKRRRNRCQYCRFQKCLKVGM

**>PxFTZ-F1a** (scaffold:P_xenopodis_South_Africa_v1_0_4:PXEA_contig0079484)

CPVCGDRVSGYHYGLPTCESCKGFFKRTVQNKKEYQCTENGHCTIDRVHRKRCAYCRFQKCLVVGM

**>PxFTZ-F1b** (scaffold:P_xenopodis_South_Africa_v1_0_4:PXEA_scaffold0039370)

CPICGDKISGYHYGIFCCESCKGFFKRTVQNAKRYSCHMPSSNALCHVSLATRKKCPACRFVKCVNKGM

**>Px2DBDa** (scaffold:P_xenopodis_South_Africa_v1_0_4:PXEA_contig0047645, PXEA_contig0222941, PXEA_contig0021551, PXEA_contig0050034)

CQVCGELAAGFHHGAYVCEACKKFFMRHSLNDSKPTNSCPSAGNCVVAKGSRGKCQQCRYRKCLDVGM

CRVCGGRSSGFHFGALTCEGCKGFFRRTEGTSQRLACVGQDNCAITPRSRNACKGCRFRRCLTAGM

**>**Px**2DBDb** (scaffold:P_xenopodis_South_Africa_v1_0_4:PXEA_contig0055664, PXEA_contig0041119, PXEA_contig0022734)

CQVCGQPAVGFHHRAYVCEACKFFTRHASARLRSASSDNASTSIVTGDTEGLDQSPSASANGGSCNVPASQVMACPMGGGCRVEGPGRGKCPHCRFKKCLDLGM

CRVCSGPSSGFHFGALTCEGCKGFFRRTVLSGVHLECLNNNDCPVTPSNRNMCKACRFNRCLAVGM

**>Px2DBDg** (scaffold:P_xenopodis_South_Africa_v1_0_4:PXEA_contig0052912, PXEA_scaffold0022799, PXEA_scaffold0026460)

CDICGDVSAGYHCNAYVCEACKKFFIRSSKGRSYSKYTCTKASNCDIKKDTRTHCQYCRFQKCLHIGM

CRVCGAKSSGFHFGAITCEGCKGFFRRTVNERDGHRYTCRNGGNCSVTGSTRNNCKSCRYQRCLLVGM

**18. NRs in *Schistocephalus solidus* (20)(** **prjeb527)**

**>SsTR** (scaffold:S_solidus_NST_G2_v1_5_4:SSLN_scaffold0004792)

CVVCGDNATGFHYRAMTCEGCKGFFRRSVQKKLEYTCKFNGRCSVADKQNRNSCQKCRFDRCIKGGM

**>SsE78** (scaffold:S_solidus_NST_G2_v1_5_4:SSLN_scaffold0002163)

CKVCGDKASGYHYGVVSCEGCKGFFRRSIQKQIEYKCLRDGKCIVIRLNRNRCQYCRFRKCIAVGM

**>SsHR96a** (scaffold:S_solidus_NST_G2_v1_5_4:SSLN_scaffold0010768)

CVVCGEPASGYNFDRLTCESCKAFFRRNALKPRDKIKACGRSGDCNIEGSQRKHCPSCRLEKCLAVGM

**>SsHR96b** (scaffold:S_solidus_NST_G2_v1_5_4:SSLN_scaffold0002953, SSLN_scaffold0004774)

CKVCGDRAVNHNFGQLTCESCKAFFRRNAHKELTCTSKTGEHVVSPTTRRECPACRLKRCFLIGM

**>SsHR96c** (scaffold:S_solidus_NST_G2_v1_5_4:SSLN_scaffold0001820)

CAVCGDHAVGYNFGAIACESCKAFFRRNALRPTIPPCLFSGKCSIQVKTRRFCSPCRLEKCFAVGM

**>SsNR1a** (scaffold:S_solidus_NST_G2_v1_5_4:SSLN_scaffold0006738)

CTVCGDKASGTHYGAWTCEGCKGFFRRSVQRKSAYFCVRNNKCDITRTLRNRCQKCRFVKCLRMGM

**>SsNR1b** (scaffold:S_solidus_NST_G2_v1_5_4:SSLN_scaffold0001587)

CLVCGDTASGVHYGVLSCEGCKGFFRRTLQDKRSQPKKCLRDGLCRITIKSRNNCQPCRLRRCVELGM

**>SsRXR1** (scaffold:S_solidus_NST_G2_v1_5_4:SSLN_scaffold0013603)

CSVCGDRATGRHYGVVSCEGCKGFFKRTVRRGAQYVCKETGCCLIDRSQRPRCQYCRFRQCLAAGM

**>SsRXR2** (scaffold:S_solidus_NST_G2_v1_5_4:SSLN_scaffold0000940)

CSICGDRANGKHYGAVACEGCKGFFKRSVRKNIKYLCREGENCQMSVRQRNRCQYCRFHKCLLEGM

**>SsCoup-TFI** (scaffold:S_solidus_NST_G2_v1_5_4:SSLN_scaffold0000564)

CVVCGDKSSGKHYGQHTCEGCKSFFKRSVRRNLIYKCRGNRQCPVDMHHRNQCQHCRFQKCLKTGM

**>SsCoupUn** (scaffold:S_solidus_NST_G2_v1_5_4:SSLN_scaffold0001658)

VCGDESRGRHYGQYACEACKSFFKRSVRKGPAYICLRGGTCDIDARLRNQCRHCRWQRCLAVGM

**>SsHNF4** (scaffold:S_solidus_NST_G2_v1_5_4:SSLN_scaffold0008005)

GHYGAYSCDGCKGFFRRSVRRKHTYSCRYNRACVIDKDMRNQCRFCRLKKCFRVGM

**>SsTR4** (scaffold:S_solidus_NST_G2_v1_5_4:SSLN_scaffold0002139)

CRVCGDRAGRHYGVLSCEGCKGFFKRSIRGHVNYMCRTKQRCIVNKAYRNRCQYCRLQKCLQVGM

**>SsTLL** (scaffold:S_solidus_NST_G2_v1_5_4:SSLN_scaffold0005388)

CKVCKDHSSGKHYGIYACDGCAGFFKRSIRRNRQYSCKSRNGTEASCRIDKPHRNQCRACRLQKCLDAGM

**>SsNR4A** (scaffold:S_solidus_NST_G2_v1_5_4:SSLN_scaffold0000778)

CLVCGDNAACQHYGVRTCEGCKGFFKRTIQKNAHYVCLQSKNCVVDKRRRNRCQYCRFQKCLKVGM

**>SsFTZ1-F1a** (scaffold:S_solidus_NST_G2_v1_5_4:SSLN_scaffold0005013)

CPVCGDKVSGYHYGLPTCESCKGFFKRTVQNKKEYHCTDTGACTIDRVHRKRCAHCRFQKCLSVGM

**>SsFTZ-F1b** (scaffold:S_solidus_NST_G2_v1_5_4:SSLN_scaffold0000283)

CPICGDKISGYHYGIFCCESCKGFFKRTVQNAKRYACHMATPSSLCEITAASRKKCPACRFVKCLEKGM

**>Ss2DBDa** (scaffold:S_solidus_NST_G2_v1_5_4:SSLN_scaffold0003297, SSLN_scaffold0004118)

CQICGELAAGFHHGAYVCEACKKFFMRQSLSNGKSTTVCPSGGNCVIAKTSRGRCQQCRYKKCLELGM

CRVCGGRSSGFHFGALTCEGCKGFFRRTEETASRLVCVGGKDNCSITPRSRNACKACRFRRCLRAGM

**>Ss2DBDb** (scaffold:S_solidus_NST_G2_v1_5_4:SSLN_scaffold0008898, SSLN_scaffold0000795)

CQVCGQPSVGFHHRAYVCEACK>KFFTRHMTMRVRKSKEGTEDTEAFAGRELRTVCPMGGHCKIEGPGRGKCPHCRFRKCLDLGM

GFFRRTAISNVKLECLGKHNCCITPANRNMCKSCRFQRCLAVGM

**>Ss2DBDg** (scaffold:S_solidus_NST_G2_v1_5_4:SSLN_scaffold0003320, SSLN_scaffold0002431)

CDICGDVSAGFHCNAYVCEACK>KFFIRSSKNENYAKYSCSKVNSCEVNKDTRTHCQRCRYQKCLRIGM

CRVCGAQSSGFHFGAITCEGCK>GFFRRTISERDNQRYTCRNGGACLITLATRNNCKSCRYRKCLSVGM

**19. NRs in *Schistosoma mansoni* (21)**

**>SmTRa** (GeneBank: AH013464.2)

CVVCGDNATGFHYRAMTCEGCKGFFRRSVQKKLVYTCKYNGRCSVSDKQNRNSCQKCRFDRCIKGGM

**>SmTRb** (GeneBank: AY688265.1)

CVVCGDNATGFHYRAMTCEGCKGFFRRSVQKKLIYTCKFQGRCSVSDKQNRNSCQKCRFDRCISGGM

**>SmE78** (GeneBank: AY395058.2)

CKVCGDKASGYHYGVISCEGCKGFFRRSIQKQIEYKCLRDGKCLVIRLNRNRCQYCRFRKCLAAGM

**>SmHR96a** (GeneBank: AY688258.1)

CIVCGEPASGYNFDRLTCESCKAFFRRNALKPRDKIKACNRGGGCAIEGNQRKHCPSCRLEKCLAVGM

**>SmHR96b** (GeneBank: AY688259.1)

CKVCGDRAVNHNFGQLTCESCKAFFRRNAHKGLSTLMVTRMLRTNDTSSLIYESNRTAHIKSRGLVELTCTAKSGEHVITPTTRRECPSCRLKQCFRVGM

**>SmNR1** (GeneBank: AH013463.2)

CVVCGDKASGFHYGVSTCEGCKGFFRRAIQRDQSYTCAKNGTCEINKTLRNKCQQCRLLKCIAVGM

**>SmHNF4** (GeneBank: AY688257.1)

CLICEDKATGKHYGAYSCDGCKGFFRRSVRRKHSYTCRHKKNCIVTKDKRNQCRFVGFRKCFRVGM

**>SmRXR1** (GeneBank: AF094759)

CVICGDKASGKHYGVISCEGCKGFFKRTVRKQLVYVCRESGQCPVDRRKRTRCQHCRFEQCLAKGM

**>SmRXR-2** (GeneBank: AF129816.3; AF158102)

CSICSDRASGKHYGVFSCEGCKGFFKRTVRKELTYICRDSQECQIDKRLRNRCQYCRYQKCLRAGM

**>SmTR4** (GeneBank: AY688263.1)

CKVCGDKASGRHYGVVSCEGCKGFFKRSIRGHVSYVCRSEQNCLVNKAYRNRCQYCRLQKCLAVGM

**>SmTLL** (GeneBank: AY698060.1)

CHVCEDHSSGKHYGIYACDGCAGFFKRSIRRNRQYTCKSRGTTIGSKSGIVVCRVDKSHRNQCRACRLTKCLEVGM

**>SmPNR** (GeneBank: AY688261.2)

CIVCGDISSGKHYGILACNGCSGFFKRSVRRKLIYRCQAGNGLCIIDKAHRNQCQACRMKKCIRMGM

**>SmDSF** (GeneBank: AY688254.1)

CGVCGDRSTGKHYGVYSCDGCSGFFKRSIHKNRSYTCKASGNLKGKCTIDRHHRNHCRACRLNKCFLAQM

**>SmCoup-TFI** (GeneBank: AY688252.1)

CVVCGDKSSGKHYGQYTCEGCKSFFKRSVRRQLNYTCRNNKQCPIDINHRNQCQYCRFQKCIKVGM

**>SmCoup-TFII** (GeneBank: AY688253.1)

VCGDRSSGKHYGQYTCEGCKSFFKRSVRKSANYVCRSGGQCPVDAQRRNQCQACRLSRCLLAGM

**>SmNR4A5** (GeneBank: AY688260.1)

CLVCGDNAACQHYGVRTCEGCKGFFK>RTIQKNAQYVCLQAKNCVVDKRRRNRCHYCRFQKCLKVGM

**>SmFTZ-F1a** (GeneBank: AY688256.1)

CPICGDKVSGYHYGLPTCESCKGFFKRTVQNKKEYHCNEQGNCVVDRLHRKRCAYCRFQKCLIVGM

**>SmFTZ-F1b** (GeneBank: AF158103)

CPICGDKISGYHYGIFCCESCKGFFKRTVQNAKRYACHRPNASSRCEINVASRKKCPACRFLKCVDKGM

**>Sm2DBDa** (GeneBank: AH013462.2)

CQVCGELAAGFHHGAYVCEACKKFFMRHSMADTKPTNVCPTGGNCIVAKGSRGKCQICRYRKCLLVGM

CRVCGGRSSGFHFGALTCEGCKGFFRRTEGSSNSLVCVGGQNACTITPRSRNACKSCRFRRCLAAGM

**>Sm2DBDb** (GeneBank: AY688251.2)

CQICGQPAVGFHHRAYVCEACKKFFMRHTAARLRNSEIGSTVSESICPMGGRCRVEGPGRGKCPHCRYRKCLELGM

CRVCSGPSSGFHFGALTCEGCKGFFRRTVLSNVRLECPGNNDCPITPANRNMCKSCRFQRCLAVGM

**>Sm2DBDg** (GeneBank: AY698061.2)

CDICGDVAAGFHCNAYVCEACKKFFIRSSKGENFTKYTCTKSNTCEINKDTRTHCQRCRYQKCIRLGM

CRVCGAKSSGFHFGAITCEGCKGFFRRTINERESQRYTCRNGGNCAVTGATRNNCKSCRYRRCLAVGM

**20. NRs in *Schistosoma mattheei* (20)(** **prjeb523)**

**>SmtTRa** (scaffold:S_mattheei_Denwood_v1_0_4:SMTD_contig0015740)

CVVCGDNATGFHYRAMTCEGCKGFFRRSVQKKLVYTCKYNGRCSVSDKQNRNSCQKCRFDRCIKGGM

**>SmtTRb** (scaffold:S_mattheei_Denwood_v1_0_4:SMTD_contig0006396)

CVVCGDNATGFHYRAMTCEGCKGFFRRSVQKKLVYTCKFQGHCSVSDKQNRNSCQKCRFDRCISGGM

**>SmtE78** (scaffold:S_mattheei_Denwood_v1_0_4:SMTD_scaffold0002842, SMTD_contig0000014)

CKVCGDKASGYHYGVISCEGCKGFFRRSIQKQIEYKCLRDGKCLVIRLNRNRCQYCRFRKCLAAGM

**>SmtHR96a** (scaffold:S_mattheei_Denwood_v1_0_4:SMTD_contig0000060)

CIVCSEPASGYNFDRLTCESCKAFFRRNALKPRDKIKACNRGGGCAIEGNQRKHCPSCRLEKCLAVGM

**>SmtHR96b** (scaffold:S_mattheei_Denwood_v1_0_4:SMTD_contig0002298)

CKVCGDRAVNHNFGQLTCESCKAFFRRNAHKGLSTLMVTRMLRTNDTSSLIYEPNRTAHIKSRGLVLTCTAKSGEHVITPTTRRECPSCRLKQCFRVGM

**>SmtNR1a** (scaffold:S_mattheei_Denwood_v1_0_4:SMTD_contig0000079)

CVVCGDKASGFHYGVSTCEGCKGFFRRAIQRDQSYTCAKNGTCEINKTLRNKCQQCRLLKCIAVGM

**>SmtHNF4** (scaffold:S_mattheei_Denwood_v1_0_4:SMTD_scaffold0000021)

GKHYGAYSCDGCKGFFRRSVRRKHSYTCRHKKNCIVTKDKRNQCRFCRFRKCFRVGM

**>SmtRXR1** (scaffold:S_mattheei_Denwood_v1_0_4:SMTD_scaffold0004265)

CVICGDKASGKHYGVISCEGCKGFFKRTVRKQLVYVCRESGQCPVDRRKRTRCQHCRFEQCLAKGM

**>SmtRXR2** (scaffold:S_mattheei_Denwood_v1_0_4:SMTD_scaffold0002035)

CSICSDRASGKHYGVFSCEGCKGFFKRTVRKELTYICRDNQECQIDKRLRNRCQYCRYQKCLRAGM

**>SmtTR4** (scaffold:S_mattheei_Denwood_v1_0_4:SMTD_scaffold0004936)

CKVCGDKASGRHYGVVSCEGCKGFFKRSIRGHVSYVCRSEQNCLVNKAYRNRCQYCRLQKCLAVGM

**>SmtPNR** (scaffold:S_mattheei_Denwood_v1_0_4:SMTD_scaffold0009457)

CIVCGDISSGKHYGILACNGCSGFFKRSVRRKLIYRCQAGNGLCIIDKAHRNQCQACRMKKCIRMGM

**>SmtDSF** (scaffold:S_mattheei_Denwood_v1_0_4:SMTD_scaffold0010935)

CGVCGDRSTGKHYGVYSCDGCSGFFKRSIHKNRSYTCKASGNLKGKCTIDRHHRNHCRACRLNKCFLAQM

**>SmtCoup-TFI** (scaffold:S_mattheei_Denwood_v1_0_4:SMTD_scaffold0015090)

CVVCGDKSSGKHYGQHTCEGCKSFFKRSVRRQLNYTCRSNKQCPIDIHHRNQCQYCRFQKCIQVGM

**>SmtCoup-TFII** (scaffold:S_mattheei_Denwood_v1_0_4:SMTD_scaffold0001095)

CQVCGDRSSGKHYGQYTCEGCKSFFKRSVRKSANYVCRSGGQCPVDAQRRNQCQACRLSRCLLAGM

**>SmtNR4A** (scaffold:S_mattheei_Denwood_v1_0_4:SMTD_scaffold0023895, SMTD_scaffold0019555)

CLVCGDNAACQHYGVRTCEGCKGFFKRTIQKNAQYVCLQAKNCIVDKRRRNRCQYCRFQKCLKVGM

**>SmtFTZ-F1a** (scaffold:S_mattheei_Denwood_v1_0_4:SMTD_scaffold0019141)

CPICGDKVSGYHYGLPTCESCK>GFFKRTVQNKKEYHCNEQGNCVVDRLHRKRCASCRFQKCLIVGM

**>SmtFTZ-F1b** (scaffold:S_mattheei_Denwood_v1_0_4:SMTD_contig0009106, )

CPICGDKISGYHYGIFCCESCKGFFKRTVQNAKRYACHRPNASSRCEINVASRKKCPACRFLKCVDKGM

**>Smt2DBDa** (scaffold:S_mattheei_Denwood_v1_0_4:SMTD_scaffold0008852, SMTD_scaffold0025118, SMTD_scaffold0018089)

CQVCGELAAGFHHGAYVCEACKKFFMRHSMADTKPTNVCPTGGNCIVAKGSRGKCQICRYRKCLLVGM

CRVCGGRSSGFHFGALTCEGCKGFFRRTEGSSNSLVCVGGQNACTITPRSRNACKSCRFRRCLAAGM

**>Smt2DBDb** (scaffold:S_mattheei_Denwood_v1_0_4:SMTD_scaffold0011126, SMTD_contig0010677, SMTD_scaffold0006005)

CQICGQPAVGFHHRAYVCEACKKFFMRHTAARLRNSEIGSTVSESICPMGGRCRVEGPGRGKCPHCRYRKCLELGM

CRVCSGPSSGFHFGALTCEGCKGFFRRTVLSNVRLECLGNNDCPITPANRNMCKSCRFQRCLAVGM

**>Smt2DBDg** (scaffold:S_mattheei_Denwood_v1_0_4:SMTD_scaffold0015363, SMTD_scaffold0007923, SMTD_contig0015764, SMTD_scaffold0007481)

CDICGDVAAGFHCNAYVCEACKKFFIRSSKGENFTKYTCTKSNTCEINKDTRTHCQRCRYQKCIRLGM

CRVCGAKSSGFHFGAITCEGCKGFFRRTINERESQRYTCRNGGNCAVTGATRNNCKSCRYRRCLAVGM

**21. NRs in *Schistosoma bovis* (21)(** **prjna451066)**

**>SbTRa** (scaffold:ASM395894v1:SBOVIS_1997)

CVVCGDNATGFHYRAMTCEGCKGFFRRSVQKKLVYTCKYNGRCSVSDKQNRNSCQKCRFDRCIKGGM

**>SbTRb** (scaffold:ASM395894v1:SBOVIS_2664)

CVVCGDNATGFHYRAMTCEGCKGFFRRSVQKKLVYTCKFQGHCSVSDKQNRNSCQKCRFDRCISGGM

**>SbE78** (scaffold:ASM395894v1:SBOVIS_1861)

CVVCGDNATGFHYRAMTCEGCKGFFRRSIQKQIEYKCLRDGKCLVIRLNRNRCQYCRFRKCLAAGM

**>SbHR96a** (scaffold:ASM395894v1:SBOVIS_1757)

CIVCSEPASGYNFDRLTCESCKAFFRRNALKPRDKIKACNRGGGCAIEGNQRKHCPSCRLEKCLAVGM

**>SbHR96b** (scaffold:ASM395894v1:SBOVIS_2171, SBOVIS_2902)

CKVCGDRAVNHNFGQLTCESCKAFFRRNAHKGLSTLMVTRMLRTNDTSSLIYEPNRTAHIKSRGLVELTCTAKSGEHVITPTTRRECPSCRLKQCFRVGM

**>SbNR1a** (scaffold:ASM395894v1:SBOVIS_2717)

CVVCGDKASGFHYGVSTCEGCKGFFRRAIQRDQSYTCAKNGTCEINKTLRNKCQQCRLLKCIAVGM

**>SbHNF4** (scaffold:ASM395894v1:SBOVIS_3019)

CLICEDKATGKHYGAYSCDGCKGFFRRSVRRKHSYTCRHKKNCIVTKDKRNQCRFCRFRKCFRVGM

**>SbRXR1** (scaffold:ASM395894v1:SBOVIS_1795)

CVICGDKASGKHYGVISCEGCKGFFKRTVRKQLVYVCRESGQCPVDRRKRTRCQHCRFEQCLAKGM

**>SbRXR2** (scaffold:ASM395894v1:SBOVIS_2015)

CSICSDRASGKHYGVFSCEGCKGFFKRTVRKELTYICRDNQECQIDKRLRNRCQYCRYQKCLRAGM

**>SbTR4** (scaffold:ASM395894v1:SBOVIS_2116)

CKVCGDKASGRHYGVVSCEGCKGFFKRSIRGHVSYVCRSEQNCLVNKAYRNRCQYCRLQKCLAVGM

**>SbTLL** (scaffold:ASM395894v1:SBOVIS_2180)

CHVCEDHSSGKHYGIYACDGCAGFFKRSIRRNRQYTCKSRGTTVGSKSGIVVCRVDKSHRNQCRACRLTKCLEVGM

**>SbPNR** (scaffold:ASM395894v1:SBOVIS_1544)

CIVCGDISSGKHYGILACNGCSGFFKRSVRRKLIYRCQAGNGLCIIDKAHRNQCQACRMKKCIRMGM

**>SbDSF** (scaffold:ASM395894v1:SBOVIS_1300)

CGVCGDRSTGKHYGVYSCDGCSGFFKRSIHKNRSYTCKASGNLKGKCTIDRHHRNHCRACRLNKCFLAQM

**>SbCoup-TFI** (scaffold:ASM395894v1:SBOVIS_2837)

CVVCGDKSSGKHYGQHTCEGCKSFFKRSVRRQLNYTCRSNKQCPIDIHHRNQCQYCRFQKCIQVGM

**>SbCoup-TFII** (scaffold:ASM395894v1:SBOVIS_4059)

VCGDRSSGKHYGQYTCEGCKSFFKRSVRKSANYVCRSGGQCPVDAQRRNQCQACRLSRCLLAGM

**>SbNR4A** (scaffold:ASM395894v1:SBOVIS_2954)

CLVCGDNAACQHYGVRTCEGCKGFFKRTIQKNAQYVCLQAKNCVVDKRRRNRCQYCRFQKCLKVGM

**>SbFTZ-F1a** (scaffold:ASM395894v1:SBOVIS_1652)

CPICGDKVSGYHYGLPTCESCKGFFKRTVQNKKEYHCNEQGNCVVDRLHRKRCAYCRFQKCLIVGM

**>SbFTZ-F1b** (scaffold:ASM395894v1:SBOVIS_3583)

CPICGDKISGYHYGIFCCESCKGFFKRTVQNAKRYACHRPNASSRCEINVASRKKCPACRFLKCVDKGM

**>Sb2DBDa** (scaffold:ASM395894v1:SBOVIS_1701)

CQVCGELAAGFHHGAYVCEACKFFMRHSMADTKPTNVCPTGGNCIVAKGSRGKCQICRYRKCLLVGM

CRVCGGRSSGFHFGALTCEGCKGFFRRTEGSSNSLVCVGGQNACTITPRSRNACKSCRFRRCLAAGM

**>Sb2DBDb** (scaffold:ASM395894v1:SBOVIS_2072)

CQICGQPAVGFHHRAYVCEACKKFFMRHTAARLRNSEIGSTVSESICPMGGRCRVEGPGRGKCPHCRYRKCLELGM

CRVCSGPSSGFHFGALTCEGCKGFFRRTVLSNVRLECLGNNDCPITPANRNMCKSCRFQRCLAVGM

**>Sb2DBDg** (scaffold:ASM395894v1:SBOVIS_1955)

CDICGDVAAGFHCNAYVCEACKKFFIRSSKGENFTKYTCTKSNTCEINKDTRTHCQRCRYQKCIRLGM

CRVCGAKSSGFHFGAITCEGCKGFFRRTINERESQRYTCRNGGNCAVTGATRNNCKSCRYRRCLAVGM

**22. NRs in *Schistosoma curassoni* (21)(prjeb519)**

**>ScTRa** (scaffold:S_curassoni_Dakar_v1_0_4:SCUD_scaffold0000561)

CVVCGDNATGFHYRAMTCEGCKGFFRRSVQKKLVYTCKYNGRCSVSDKQNRNSCQKCRFDRCIKGGM

**>ScTRb** (scaffold:S_curassoni_Dakar_v1_0_4:SCUD_scaffold0006781)

CVVCGDNATGFHYRAMTCEGCKGFFRRSVQKKLVYTCKFQGHCSVSDKQNRNSCQKCRFDRCISGGM

**>ScE78** (scaffold:S_curassoni_Dakar_v1_0_4:SCUD_scaffold0000887, SCUD_scaffold0000676)

CKVCGDKASGYHYGVISCEGCKGFFRRSIQKQIEYKCLRDGKCLVIRLNRNRCQYCRFRKCLAAGM

**>ScHR96a** (scaffold:S_curassoni_Dakar_v1_0_4:SCUD_scaffold0018286)

CIVCSEPASGYNFDRLTCESCKAFFRRNALKPRDK

**>ScHR96b** (scaffold:S_curassoni_Dakar_v1_0_4:SCUD_contig0002662)

CKVCGDRAVNHNFGQLTCESCKAFFRRNAHKGLSTLMVTRMLRTNDTSSLIYEPNRTAHIKSRGLVELTCTAKSGEHVITPTTRRECPSCRLKQCFRVGM

**>ScNR1a** (scaffold:S_curassoni_Dakar_v1_0_4:SCUD_scaffold0006696)

CVVCGDKASGFHYGVSTCEGCKGFFRRAIQRDQSYTCAKNGTCEINKTLRNKCQQCRLLKCIAVGM

**>ScHNF4** (scaffold:S_curassoni_Dakar_v1_0_4:SCUD_contig0001009)

CSICSDRASGKHYGAYSCDGCKGFFRRSVRRKHSYTCRHKKNCTVTKDKRNQCRFCRFRKCFRVGM

**>ScRXR1** (scaffold:S_curassoni_Dakar_v1_0_4:SCUD_scaffold0005787)

CVICGDKASGKHYGVISCeGFFKRTVRKQLVYVCRESGQCPVDRRKRTRCQHCRFEQCLAKGM

**>ScRXR2** (scaffold:S_curassoni_Dakar_v1_0_4:SCUD_scaffold0005454)

CSICSDRASGKHYGVFSCEGCKGFFKRTVRKELTYICRDNQECQIDKRLRNRCQYCRYQKCLRAGM

**>ScTR4** (scaffold:S_curassoni_Dakar_v1_0_4:SCUD_contig0001271)

CKVCGDKASGRHYGVVSCEGCKGFFKRSIRGHVSYVCRSEQNCLVNKAYRNRCQYCRLQKCLAVGM

**>ScTLL** (scaffold:S_curassoni_Dakar_v1_0_4:SCUD_scaffold0009232)

CHVCEDHSSGKHYGIYACDGCAGFFKRSIRRNRQYTCKSRGTXXXXKSGIVVCRVDKSHRNQCRACRLTKCLEVGM

**>ScPNR** (scaffold:S_curassoni_Dakar_v1_0_4:SCUD_scaffold0000250)

CIVCGDISSGKHYGILACNGCSGFFKRSVRRKLIYRCQAGNGLCIIDKAHRNQCQACRMKKCIRMGM

**>ScDSF** (scaffold:S_curassoni_Dakar_v1_0_4:SCUD_scaffold0002566)

CGVCGDRSTGKHYGVYSCDGCSGFFKRSIHKNRSYTCKASGNLKGKCTIDRHHRNHCRACRLNKCFLAQM

**>ScCoup-TFI** (scaffold:S_curassoni_Dakar_v1_0_4:SCUD_scaffold0000257)

CVVCGDKSSGKHYGQHTCEGCKSFFKRSVRRQLNYTCRSNKQCPIDIHHRNQCQYCRFQKCIQVGM

**>ScCoup-TFII** (scaffold:S_curassoni_Dakar_v1_0_4:SCUD_scaffold0000251)

CQVCGDRSSGKHYGQYTCEGCKSFFKRSVRKSANYICRSGGQCPVDAQRRNQCQACRLSRCLLAGM

**>ScNR4A** (scaffold:S_curassoni_Dakar_v1_0_4:SCUD_contig0000854, GenBank_ VDO60662.1)

CLVCGDNAACQHYGVRTCEGCKGFFKRTIQKNAQYVCLQAKNCVVDKRRRNRCQYCRFQKCLKVGM

**>ScFTZ-F1a** (scaffold:S_curassoni_Dakar_v1_0_4:SCUD_scaffold0015416)

CPICGDKVSGYHYGLPTCESCKGFFKRTVQNKKEYHCNEQGNCVVDRLHRKRCAYCRFQKCLIVGM

**>ScFTZ-F1b** (scaffold:S_curassoni_Dakar_v1_0_4:SCUD_scaffold0001406)

CPICGDKISGYHYGIFCCESCKGFFKRTVQNAKRYACHRPNASSRCEINVASRKKCPACRFLKCVDKGM

**>Sc2DBDa** (scaffold:S_curassoni_Dakar_v1_0_4:SCUD_contig0002110, SCUD_scaffold0008574, SCUD_scaffold0014881, GenBank_VDP65226.1)

CQVCGELAAGFHHGAYVCEACKKFFMRHSMADTKPTNVCPTGGNCIVAKGSRGKCQICRYRKCLLVGM

CRVCGGRSSGFHFGALTCEGCKGFFRRTEGSSNSLVCVGGQNACTITPRSRNACKSCRFRRCLAAGM

**>Sc2DBDb** (scaffold:S_curassoni_Dakar_v1_0_4:SCUD_contig0019009, SCUD_scaffold0011476, SCUD_scaffold0002853)

CQICGQPAVGFHHRAYVCEACKKFFMRHTAARLRNSEIGSTVSESICPMGGRCRVEGPGRGKCPHCRYRKCLELGM

CRVCSGPSSGFHFGALTCEGCKGFFRRTVLSNVRLECLGNNDCPITPANRNMCKSCRFQRCLAVGM

**>Sc2DBDg** (scaffold:S_curassoni_Dakar_v1_0_4:SCUD_scaffold0001299, SCUD_contig0000701)

CDICGDVAAGFHCNAYVCEACKKFFIRSSKGENFTKYTCTKSNTCEINKDTRTHCQRCRYQKCIRLGM

CRVCGAKSSGFHFGAITCEGCKGFFRRTINERESQRYTCRNGGNCAVTGATRNNCKSCRYRRCLAVGM

**23. NRs in *Schistosoma haematobium* (21)(** **prjna78265)**

**>ShTRa** (scaffold:SchHae_2.0:scaffold020109)

CVVCGDNATGFHYRAMTCEGCKGFFRRSVQKKLVYTCKYNGRCSVSDKQNRNSCQKCRFDRCIKGGM

**>ShTRb** (scaffold:SchHae_2.0:scaffold020134)

CVVCGDNATGFHYRAMTCEGCKGFFRRSVQKKLVYTCKFQGHCSVSDKQNRNSCQKCRFDRCISGGM

**>ShNR1a** (scaffold:SchHae_2.0:scaffold020105)

CVVCGDKASGFHYGVSTCEGCKGFFRRAIQRDQSYTCAKNGTCEINKTLRNKCQQCRLLKCIAVGM

**>ShE78** (scaffold:SchHae_2.0:scaffold020040)

CKVCGDKASGYHYGVISCEGCKGFFRRSIQKQIEYKCLRDGKCLVIRLNRNRCQYCRFRKCLAAGM

**>ShHR96a** (scaffold:SchHae_2.0:scaffold020138)

CIVCSEPASGYNFDRLTCESCKAFFRRNALKPILQIKACNRGGGCAIEGNQRKHCPSCRLEKCLAVGM

**>ShHR9b** (scaffold:SchHae_2.0:scaffold020153)

CKVCGDRAVNHNFGQLTCESCKAFFRRNAHKELTCTAKSGEHVITPTTRRECPSCRLKQCFRVGM

**>ShHNF4** (scaffold:SchHae_2.0:scaffold020220)

CLICEDKATGKHYGAYSCDGCKGFFRRSVRRKHSYTCRHKKNCIVTKDKRNQCRFCRFRKCFRVGM

**>ShRXR1** (scaffold:SchHae_2.0:scaffold020235)

CVICGDKASGKHYGVISCEGCKGFFKRTVRKQLVYVCRESGQCPVDRRKRTRCQHCRFEQCLAKGM

**>ShRXR2** (scaffold:SchHae_2.0:scaffold020145)

CSICSDRASGKHYVFSCEGCKGFFKRTVRKELTYICRDNQECQIDKRLRNRCQYCRYQKCLRAGM

**>ShTR4** (scaffold:SchHae_2.0:scaffold020089)

CKVCGDKASGRHYGVVSCEGCKGFFKRSIRGHVSYVCRSEQNCLVNKAYRNRCQYCRLQKCLAVGM

**>ShTLL** (scaffold:SchHae_2.0:scaffold020201)

CHVCEDHSSGKHYGIYACDGcagffkRSIRRNRQYTCKSRGTTVGSKSGIVICRVDKSHRNQCRACRLTKCLEVGM

**>ShPNR** (scaffold:SchHae_2.0:scaffold020153)

CIVCGDISSGKHYGILACNGCSGFFKRSVRRKLIYRCQAGNGLCIIDKAHRNQCQACRMKKCIRMGM

**>ShDSF** (scaffold:SchHae_2.0:scaffold020167)

CGVCGDRSTGKHYGVYSCDGCSGFFKRSIHKNRSYTCKASGNLKGKCTIDRHHRNHCRACRLNKCFLAQM

**>ShCoup-TFI** (scaffold:SchHae_2.0:scaffold020070)

CVVCGDKSSGKHYGQHTCEGCKSFFKRSVRRQLNYTCRSNKQCPIDIHHRNQCQYCRFQKCIQVGM

**>ShCoup-TFII** (scaffold:SchHae_2.0:scaffold020173)

CQVCGDRSSGKHYGQYTCEGCKSFFKRSVRKSANYVCRSGGQCPVDAQRRNQCQACRLSRCLLAGM

**>ShNR4A** (scaffold:SchHae_2.0:scaffold020101)

CLVCGDNAACQHYGVRTCEGCKGFFKRTIQKNAQYVCLQAKNCVVDKRRRNRCQYCRFQKCLKVGM

**>ShFTZa** (scaffold:SchHae_2.0:scaffold020139)

CPICGDKVSGYHYGLPTCESCKGFFKRTVQNKKEYHCNEQGNCVVDRLHRKRCAYCRFQKCLIVGM

**>ShFTZb** (scaffold:SchHae_2.0:scaffold020150)

CPICGDKISGYHYGIFCCESCKGFFKRTVQNAKRYACHRPNASSRCEINVASRKKCPACRFLKCVDKGM

**>Sh2DBDa** (scaffold:SchHae_2.0:scaffold020057)

CQVCGELAAGFHHGAYVCEACKKFFMRHSMADTKPTNVCPTGGNCIVAKGSRGKCQICRYRKCLLVGM

CRVCGGRSSGFHFGALTCEGCKGFFRRTEGSSNSLVCVGGQNACTITPRSRNACKSCRFRRCLAAGM

**>Sh2DBDb** (scaffold:SchHae_2.0:scaffold020145)

CQICGQPAVGFHHRAYVCEACKKFFMRHTAARLRNSEIGSTVSESICPMGGRCRVEGPGRGKCPHCRYRKCLELGM

CRVCSGPSSGFHFGALTCEGCKGFFRRTVLSNVRLECLGNNDCPITPANRNMCKSCRFQRCLAVGM

**>Sh2DBDg** (scaffold:SchHae_2.0:scaffold020072)

CDICGDVAAGFHCNAYVCEACKKFFIRSSKGENFTKYTCTKSNTCEINKDTRTHCQRCRYQKCIRLGM

CRVCGAKSSGFHFGAITCEGCKGFFRRTINERESQRYTCRNGGNCAVTGATRNNCKSCRYRRCLAVGM

**24. NRs in *Schistosoma japonicum* (21) (prjea34885)**

**>SjTRa** (scaffold:ASM15177v1:SJC_S000467)

CVVCGDNATGFHYRAMTCEGCKGFFRRSVQKKLIYTCKFNGRCSVSDKQNRNSCQKCRFDRCIKGGM

**>SjTRb** (scaffold:ASM15177v1:SJC_S000165)

CVVCGDNATGFHYRAMTCEGCKGFFRRSIQKKLVYTCKFQGRCSVSDKQNRNSCQKCRFDRCISGGM

**>SjE78** (scaffold:ASM15177v1:SJC_S000145)

CKVCGDKASGYHYGVISCEGCKGFFRRSIQKQIEYKCLRDGKCLVIRLNRNRCQYCRFRKCLAAGM

**>SjHR96a** (scaffold:ASM15177v1:SJC_S018521)

CIVCGEAASGYNFDRLTCESCKAFFRRNALKPRDK

**>SjHR96b** (scaffold:ASM15177v1:SJC_S000093)

GLSTLLETRMLRTNDTSSLLYESNRTSHIRSRGLVELTCTAKSGEHVITPTTRRECPSCRLKQCFRVGM

**>SjNR1a** (scaffold:ASM15177v1:SJC_S000098)

CVVCGDKASGFHYGVSTCEGCKGFFRRAIQRDQSYTCAKNGTCEINKTLRNKCQQCRLLKCIAVGM

**>SjHNF4** (scaffold:ASM15177v1:SJC_S014416)

GKHYGAYSCDGCKGFFRRSVRRKHSYTCRHKRNCIVTKDKRNQCRFCRFRKCFRVGM

**>SjRXR1** (scaffold:ASM15177v1:SJC_S000162)

CVICGDKASGKHYGVISCEGCKGFFKRTVRKQLLYICRESGQCPVDRRKRTRCQHCRFEQCLAKGM

**>SjRXR2** (scaffold:ASM15177v1:SJC_S000005)

CSICSDRASGKHYGVFSCEGCKGFFKRTVRKELSYICRDNQECQIDKRLRNRCQYCRYQKCLRVGM

**>SjTR4** (scaffold:ASM15177v1:SJC_S000620)

CRVCGDKASGRHYGVVSCEGCKGFFKRSIRGHVSYVCRSEQNCLVNKAYRNRCQYCRLQKCLAVGM

**>SjTLL** (scaffold:ASM15177v1:SJC_S004787)

CHVCEDHSSGKHYGIYACDGCAGFFKRSIRRNRQYTCKSRGPTIASKSGIVVCRVDKSHRNQCRACRLTKCLEVGM

**>SjPNR** (scaffold:ASM15177v1:SJC_S027326, GenBank_TNN09046)

CIVCGDISSGKHYGILACNGCSGFFKRSVRRKLIYRCQAGNGLCIIDKAHRNQCQACRLKKCMRMGM

**>SjDSF** (scaffold:ASM15177v1:SJC_S001150)

CGVCGDRSTGKHYGVYSCDGCSGFFKRSIHKNRSYTCKASGNLKGQCTIDRNHRNHCRACRLNKCFLAQM

**>SjCOUP-TFI** (scaffold:ASM15177v1:SJC_S000474)

CVVCGDKSSGKHYGQHTCEGCKSFFKRSVRRKLNYTCRGNRQCPIDIHHRNQCQYCRFQKCLKVGM

**>SjCoup-TFII** (scaffold:ASM15177v1:SJC_S000631)

VCGDRSSGKHYGQYTCEGCKSFFKRSVRKSAYICRSGGQCPVDAQRRNQCQACRLSRCLLAGM

**>SjNR4A** (scaffold:ASM15177v1:SJC_S000436)

CLVCGDNAACQHYGVRTCEGCKGFFKRTIQKNAQYVCLQAKNCVVDKRRRNRCQYCRFQKCLKVGM

**>SjFTZ-F1a** (scaffold:ASM15177v1:SJC_S004111)

CPICGDKVSGYHYGLPTCESCKGFFKRTVQNKKEYRCNEQGNCVVDRLHRKRCAYCRFQKCLNVGM

**>SjFTZ-F1b** (scaffold:ASM15177v1:SJC_S000126)

CPICGDKISGYHYGIFCCESCKGFFKRTVQNAKRYACHRPNASSRCEINVASRKKCPACRFLKCVDKGM

**>Sj2DBDa** (scaffold:ASM15177v1:SJC_S000258)

CQVCGELAAGFHHGAYVCEACKKFFMRHSMADTKPTNVCPTGGNCVVAKGSRGKCQICRYRKCLLVGM

CRVCGGRSSGFHFGALTCEGCKGFFRRTEGSSNSLVCVGGQNTCTITPRSRNACKSCRFRRCLAAGM

**>Sj2DBDb** (scaffold:ASM15177v1:SJC_S000490)

CQICGQPAVGFHHRAYVCEACKKFFMRHTAARLRNSETGSTISESICPMGGRCRVEGPGRGKCPHCRYRKCLELGM

CRVCSGPSSGFHFGALTCEGCKGFFRRTVLSNVRLECLGNNDCPITPANRNMCKSCRFQRCLAVGM

**>Sj2DBDg** (scaffold:ASM15177v1:SJC_S001286, SJC_S004027)

CDICGDVAAGFHCNAYVCEACKKFFIRSSKGDNFSKYTCTKSNTCEINKDTRTHCQRCRYQKCIRLGM

CRVCGAKSSGFHFGAITCEGCKGFFRRTINERESQRYTCRNGGNCTVTGATRNNCKSCRYRRCLAVGM

**25. NRs in *Schistosoma margrebowiei* (21)(prjeb522)**

**>SmaTRa** (scaffold:S_margrebowiei_Zambia_v1_5_4:SMRZ_scaffold0000137)

CVVCGDNATGFHYRAMTCEGCKGFFRRSVQKKLVYTCKYNGRCSVSDKQNRNSCQKCRFDRCIKGGM

**>SmaTRb** (scaffold:S_margrebowiei_Zambia_v1_5_4:SMRZ_contig0001334)

CVVCGDNATGFHYRAMTCEGCKGFFRRSVQKKLIYTCKFQGHCSVSDKQNRNSCQKCRFDRCISGGM

**>SmaE78** (scaffold:S_margrebowiei_Zambia_v1_5_4:SMRZ_contig0001585, SMRZ_contig0001095)

CKVCGDKASGYHYGVISCEGCK>GFFRRSIQKQIEYKCLRDGKCLVIRLNRNRCQYCRFRKCLAAGM

**>SmaHR96a** (scaffold:S_margrebowiei_Zambia_v1_5_4:SMRZ_contig0001321)

CIVCSEPASGYNFDRLTCESCKAFFRRNALKPRDKIKACNRGGGCAIEGNQRKHCPSCRLEKCLAVGM

**>SmaHR96b** (scaffold:S_margrebowiei_Zambia_v1_5_4:SMRZ_scaffold0000790)

CKVCGDRAVNHNFGQLTCESCKAFFRRNAHKGLSTLMVTRMLRTNDTSSLIYEPNRTAHIKSRGLVELTCTAKSGEHVITPTTRRECPSCRLKQCFRVGM

**>SmaNR1a** (scaffold:S_margrebowiei_Zambia_v1_5_4:SMRZ_scaffold0002727)

CVVCGDKASGFHYGVSTCEGCKGFFRRAIQRDQSYTCAKNGTCEINKTLRNKCQQCRLLKCIAVGM

**>SmaPNR** (scaffold:S_margrebowiei_Zambia_v1_5_4:SMRZ_contig0003642)

CIVCGDISSGKHYGILACNGCSGFFKRSVRRKLIYRCQAGNGLCIIDKAHRNQCQACRMKKCIRMGM

**>SmaRXR1** (scaffold:S_margrebowiei_Zambia_v1_5_4:SMRZ_scaffold0000143)

CVICGDKASGKHYGVISCEGCKGFFKRTVRKQLVYVCRESGQCPVDRRKRTRCQHCRFEQCLAKGM

**>SmaRXR2** (scaffold:S_margrebowiei_Zambia_v1_5_4:SMRZ_scaffold0001568)

CSICSDRASGKHYGVFSCEGCKGFFKRTVRKELTYICRDNQECQIDKRLRNRCQYCRYQKCLRAGM

**>SmaHNF4** (scaffold:S_margrebowiei_Zambia_v1_5_4:SMRZ_scaffold0000021)

CLICEDKATGKHYGAYSCDGCKGFFRRSVRRKHSYTCRHKKNCIVTKDKRNQCRFCRFRKCFRVGM

**>SmaDSF** (scaffold:S_margrebowiei_Zambia_v1_5_4:SMRZ_contig0005218)

CGVCGDRSTGKHYGVYSCDGCSGFFKRSIHKNRSYTCKASGNLKGKCTIDRHHRNHCRACRLNKCFLAQM

**>SmaCoup-TFI** (scaffold:S_margrebowiei_Zambia_v1_5_4:SMRZ_contig0000007)

CVVCGDKSSGKHYGQHTCEGCKSFFKRSVRRQLNYTCRSNKQCPIDIHHRNQCQYCRFQKCIQVGM

**>SmaCoup-TFII** (scaffold:S_margrebowiei_Zambia_v1_5_4:SMRZ_scaffold0000142)

VCGDRSSGKHYGQYTCEGCKSFFKRSVRKSANYVCRSGGQCPVDAQRRNQCQACRLSRCLLAGM

**>SmaTR4** (scaffold:S_margrebowiei_Zambia_v1_5_4:SMRZ_contig0000714)

CKVCGDKASGRHYGVVSCEGCKGFFKRSIRGHVSYVCRSEQNCLVNKAYRNRCQYCRLQKCLAVGM

**>SmaTLL** (scaffold:S_margrebowiei_Zambia_v1_5_4:SMRZ_contig0000025)

CHVCEDHSSGKHYGIYACDGCAFFFKRSIRRNRQYTCKSRGTTVGSKSGIVVCRVDKSHRNQCRACRLTKCLEVGM

**>SmaNR4A** (scaffold:S_margrebowiei_Zambia_v1_5_4:SMRZ_scaffold0000035)

CLVCGDNAACQHYGVRTCEGCKGFFKRTIQKNAQYVCLQAKNCVVDKRRRNRCQYCRFQKCLKVGM

**>SmaFTZ-F1a** (scaffold:S_margrebowiei_Zambia_v1_5_4:SMRZ_scaffold0002704)

CPICGDKVSGYHYGLPTCESCKGFFKRTVQNKKEYHCNEQGNCVVDRLHRKRCAYCRFQKCLIVGM

**>SmaFTZ-F1b** (scaffold:S_margrebowiei_Zambia_v1_5_4:SMRZ_scaffold0001594)

CPICGDKISGYHYGIFCCESCKGFFKRTVQNAKRYACHRPNASSRCEINVASRKKCPACRFLKCVDKGM

**>Sma2DBDa** (scaffold:S_margrebowiei_Zambia_v1_5_4:SMRZ_contig0005358, SMRZ_contig0001670)
CQVCGELAAGFHHGAYVCEACKKFFMRHSMADTKPTNVCPTGGNCIVAKGSRGKCQICRYRKCLLVGM

CRVCGGRSSGFHFGALTCEGCKGFFRRTEGSSNSLVCVGGQNACTITPRSRNACKSCRFRRCLAAGM

**>Sma2DBDb** (scaffold:S_margrebowiei_Zambia_v1_5_4:SMRZ_scaffold0000753)

CQICGQPAVGFHHRAYVCEACKKFFMRHTAARLRNSEIGSTVSESICPMGGRCRVEGPGRGKCPHCRYRKCLELGM

CRVCSGPSSGFHFGALTCEGCKGFFRRTVLSNVRLECLGNNDCPITPANRNMCKSCRFQRCLAVGM

**>Sma2DBDg** (scaffold:S_margrebowiei_Zambia_v1_5_4:SMRZ_scaffold0000738, SMRZ_contig0001477)

CDICGDVAAGFHCNAYVCEACKKFFIRSSKGENFTKYTCTKSNTCEINKDTRTHCQRCRYQKCIRLGM

CRVCGAKSSGFHFGAITCEGCKGFFRRTINERESQRYTCRNGGNCAVTGATRNNCKSCRYRRCLAVGM

**26. NRs in *Schistosoma rodhaini* (21)(prjeb526)**

**>SrTRa** (scaffold:S_rodhaini_Burundi_v1_0_4:SROB_scaffold0000351)

CVVCGDNATGFHYRAMTCEGCKGFFRRSVQKKLVYTCKYNGRCSVSDKQNRNSCQKCRFDRCIKGGM

**>SrTRb** (scaffold:S_rodhaini_Burundi_v1_0_4:SROB_scaffold0011579)

CVVCGDNATGFHYRAMTCEGCKGFFRRSVQKKLIYTCKFQGRCSVSDKQNRNSCQKCRFDRCISGGM

**>SrE78** (scaffold:S_rodhaini_Burundi_v1_0_4:SROB_scaffold0007285, SROB_scaffold0001653)

CKVCGDKASGYHYGVISCEGCKGFFRRSIQKQIEYKCXXXXXCLVIRLNRNRCQYCRFRKCLAAGM

**>SrHR96a** (scaffold:S_rodhaini_Burundi_v1_0_4:SROB_scaffold0003668)

CIVCGEPASGYNFDRLTCESCKAFFRRNALKPRDKIKACNRGGGCAIEGNQRKHCPSCRLEKCLAVGM

**>SrHR96b** (scaffold:S_rodhaini_Burundi_v1_0_4:SROB_scaffold0000004)

CKVCGDRAVNHNFGQLTCESCKAFFRRNAHKGLSTLMVTRMLRTNDTSSLIYESNRTAHIKSRGLVELTCTAKSGEHVITPTTRRECPSCRLKQCFRVGM

**>SrNR1a** (scaffold:S_rodhaini_Burundi_v1_0_4:SROB_scaffold0004166)

CVVCGDKASGFHYGVSTCEGCKGFFRRAIQRDQSYTCAKNGTCEINKTLRNKCQQCRLLKCIAVGM

**>SrHNF4** (scaffold:S_rodhaini_Burundi_v1_0_4:SROB_scaffold0007681)

CLICEDKATGKHYGAYSCDGCKGFFRRSVRRKHSYTCRHKKNCIVTKDKRNQCRFCRFRKCFRVGM

**>SrRXR1** (scaffold:S_rodhaini_Burundi_v1_0_4:SROB_scaffold0005290)

CVICGDKASGKHYGVISCEGCKGFFKRTVRKQLVYVCRESGQCPVDRRXXXRCQHCRFEQCLAKGM

**>SrRXR2** (scaffold:S_rodhaini_Burundi_v1_0_4:SROB_scaffold0001261)

GVFSCEGCKGFFKRTVRKELTYICRDNQECQIDKRLRNRCQYCRYQKCLRAGM

**>SrTR4** (scaffold:S_rodhaini_Burundi_v1_0_4:SROB_scaffold0008938)

CKVCGDKASRHYGVVSCEGCKGFFKRSIRGHVSYVCRSEQNCLVNKAYRNRCQYCRLQKCLAVGM

**>SrTLL** (scaffold:S_rodhaini_Burundi_v1_0_4:SROB_scaffold0007309)

CHVCEDHSSGKHYGIYACDGCAGFFKRSIRRNRQYTCKSRGTTIGSKSGIVVCRVDKSHRNQCRACRLTKCLEVGM

**>SrPNR** (scaffold:S_rodhaini_Burundi_v1_0_4:SROB_scaffold0002610)

CIVCGDISSGKHYGILACNGCSGFFKRSVRRKLIYRCQAGNGLCIIDKAHRNQCQACRMKKCIRMGM

**>SrDSF** (scaffold:S_rodhaini_Burundi_v1_0_4:SROB_scaffold0002009)

CVVCGDRSTGKHYGVYSCDGCSGFFKRSIHKNRSYTCKASGNLKGKCTIDRHHRNHCRACRLNKCFLAQM

**>SrCoup-TFI** (scaffold:S_rodhaini_Burundi_v1_0_4:SROB_scaffold0019080)

CVVCGDKSSGKHYGQYTCEGCKSFFKRSVRRKLNYTCRSNKQCPIDINHRNQCQYCRFQKCIKVGM

**>SrCoup-TFII** (scaffold:S_rodhaini_Burundi_v1_0_4:SROB_scaffold0004158)

CVVCGDRSSGKHYGQYTCEGCKSFFKRSVRKSANYVCRSGGQCPVDAQRRNQCQACRLSRCLLAGM

**>SrNR4A** (scaffold:S_rodhaini_Burundi_v1_0_4:SROB_scaffold0005408, SROB_scaffold0000493)

CLVCGDNAACQHYGVRTCEGCKGFFK>RTIQKNAQYVCLQAKNCVVDKRRRNRCQYCRFQKCLKVGM

**>SrFTZ-F1a** (scaffold:S_rodhaini_Burundi_v1_0_4:SROB_contig0002589)

CPICGDKVSGYHYGLPTCESCKGFFKRTVQNKKEYHCNEQGNCVVDRLHRKRCAYCRFQKCLIVGM

**>SrFTZ-F1b** (scaffold:S_rodhaini_Burundi_v1_0_4:SROB_scaffold0001835)

CPICGDKISGYHYGIFCCESCKGFFKRTVQNAKRYACHRPNASSRCEINVASRKKCPACRFLKCVDKGM

**>Sr2DBDa** (scaffold:S_rodhaini_Burundi_v1_0_4:SROB_scaffold0001061, SROB_scaffold0005682)

CQVCGELAAGFHHGAYVCEACKKFFMRHSMADTKPTNVCPTGGNCIVAKGSRGKCQICRYRKCLLVGM

CRVCGGRSSGFHFGALTCEGCKGFFRRTEGSSNSLVCVGGQNACTITPRSRNACKSCRFRRCLAAGM

**>Sr2DBDb** (scaffold:S_rodhaini_Burundi_v1_0_4:SROB_scaffold0002152, SROB_scaffold0007898, SROB_scaffold0011331, SROB_scaffold0004139)

CQICGQPAVGFHHRAYVCEACKKFFMRHTAARLRNSEIGSTVSESICPMGGRCRVEGPGRGKCPHCRYRKCLELGM

CRVCSGPSSGFHFGALTCEGCKGFFRRTVLSNVRLECLGNNDCPITPANRNMCKSCRFQRCLAVGM

**>Sr2DBDg** (scaffold:S_rodhaini_Burundi_v1_0_4:SROB_scaffold0006008, SROB_scaffold0010087, SROB_scaffold0010528)

CDICGDVAAGFHCNAYVCEACKKFFIRSSKGENFTKYTCTKSNTCEINKDTRXXXXQRCRYQKCIRLGM

CRVCGAKSSGFHFGAITCEGCKGFFRRTINERESQRYTCRNGGNCAVTGATRNNCKSCRYRRCLAVGM

**27. NRs in *Schmidtea mediterranea* (27)(prjna12585)**

**>SmeTRa** (scaffold:SmedGD_c1.3:v31.000415)

CVVCEHDASGLHYKALTCEGCKGFFRRTVQQNLTYECKFLKNCDVSRRVKKDVCRSCRWDKCLKTGM

**>SmeTRb** (nhr-12) (scaffold:SmedGD_c1.3:v31.000375)

CKVCGYKASGLHYKVMACEGCKGFFRRSVQKKLKYVCKLNGNCPINSDNRTKCQECRWQLCLNAGM

**>SmeTRc** (nhr-14) (scaffold:SmedGD_c1.3:v31.003447)

CVVCKHNASGFHYRVMACEGCKGFFRRTVQNNLKYSCKFNNNCVIQPHNRAQCQECRWKKCLSVGM

**>SmeHR96a** (nhr-11) (scaffold:SmedGD_c1.3:v31.003273)

CVVCGEKASGFNFDRLTCESCKAFFRRNAMKPKEKMKPCPHGGGCNISGSQRKHCPYCRLEKCFTEGM

**>SmeHR96b** (GenBank: SMU15000579)

CKVCFDKAVNQNFGVLSCESCKAFFRRNAIRSSPLKCSNGTDLCSVTSSTRKQCPSCRLKKCLQVGM

**>SmeHR96c** (nhr-22) (scaffold:SmedGD_c1.3:v31.000522)

CSVCGDKALGYNFGAITCESCKAFFRRNAHKKSPPPCVFNKTCEISVNTRRFCTYCRLMKCFKMQM

**>SmeCoup-TFI** (nhr-23) (scaffold:SmedGD_c1.3:v31.015705)

CVVCGDKSSGKHYGQYTCEGCKSFFKRSVRRNLTYSCRGNHNCPIDIHHRNQCQNCRFQKCLKMGM

**>SmeCoup-TFII** (nhr-7) (scaffold:SmedGD_c1.3:v31.021474)

CKVCGDKSSGRHYGQYTCEGCKSFFKRSVRNQSSYQCYASGDCVIDIQRRNNCQFCRFQKCFASGM

**>SmeNHR236** (nhr-5) (scaffold:SmedGD_c1.3:v31.020556)

CRVCGDRASGKHYGVLSCDGCRGFFKRSVRRNLEYVCKESNVCPVDVARRNQCQACRFKKCLSVKM

**>SmeHNF4** (nhr-8) (scaffold:SmedGD_c1.3:v31.002310)

CLICSDKATGKHYGAFSCDGCKGFFRRSVRKKNNYTCQYNRNCKMDKDKRNQCRYCRLKKCILVGM

**>SmeRXR1** (nhr-16) (scaffold:SmedGD_c1.3:v31.000418)

CAICTDLATGKHYGVLACEGCKGFFKRTIRKELHYTCREKNLCPITERLRNRCQSCRFNKCLDQGM

**>SmeRXR2** (scaffold:SmedGD_c1.3:v31.013694)

CSICNDNATGKHYGVISCEGCKGFFKRTVRNHLIYVCRDFNNCAVDKKMRNRCQSCRFQKCISAGM

**>SmeDSF** (nhr-4) (scaffold:SmedGD_c1.3:v31.016829)

CKVCQDRSSGKHYGIYSCDGCSGFFKRSIHKHRTYSCKVQGEMKNKCPIDKTHRNQCRACRLRKCFDSGM

**>SmeTLL** (nhr-21) (scaffold:SmedGD_c1.3:v31.002266)

CKVCQDHSSGKHYGIYACDGCAGFFKRSIRHSRLYICKNKSIKGDSWIGICKIDKTHRNQCRACRLQKCVDSGM

**>SmeTR4** (nhr-19) (scaffold:SmedGD_c1.3:v31.002714)

CVVCGDYSTGRHYGVLSCEGCKGFFKRSIRNGSRYRCRGSNGCIITKAFRNRCQHCRLQKCLNEGM

**>SmePNR** (nhr-18) (scaffold:SmedGD_c1.3:v31.001384)

CVVCGDVSSGKHYGILACNGCSGFFKRSVRRKLIYRCQAGTGRCTIDKAHRNQCQACRLKKCLLMGM

**>SmeNR4Aa** (nhr-20) (scaffold:SmedGD_c1.3:v31.002657)

CLVCGDTAACQHYGVRTCEGCKGFFKRTIQKNAQYMCLQSKNCIVDKRRRNRCQYCRFQKCLKVGM

**>SmeNR4Ab** (nhr-17) (scaffold:SmedGD_c1.3:v31.011793)

CLVCGDSASCQHYGVKSCEGCKGFFKRTIQKNASATYVCLHNQNCVIDKQNRNRCQFCRYVKCIKVGM

**>SmeFTZ-F1a** (nhr-13) (scaffold:SmedGD_c1.3:v31.003342)

CPVCNDQVSGFHYGVMTCESCKGFFKRTIQNKKEYQCLENSGCSVDINNRRRCAFCRFQKCLSVGM

**>SmeFTZ-F1b** (nhr-9) (scaffold:SmedGD_c1.3:v31.000915)

CPICEDAISGYHYGIFCCESCKGFFKRSVQNHKDYVCHAQQSGGEPCVINKHTRKKCPSCRFQRCIDKGM

**>Sme2DBDa1** (nhr-2) (scaffold:SmedGD_c1.3:v31.002475)

CQVCRESAAGFHHGAYVCEACKKFFMRHNLNSTKFTIPCPTGGQCAQLKTGRIKCQSCRFKKCVSIGM

CRVCGGKSSGFHFGALTCEGCKGFFRRTESTKQHLACITGNNNCCLTGSSSRNLCKSCRFNRCLGVGM

**>Sme2DBDa2** (nhr-3) (scaffold:SmedGD_c1.3:v31.001368)

CQICTKNSVGFHYGAYVCEACKKFFVRHASGVCRLYGSCQEKGKCDLNIDGRGKCQHCRYQRCNEIGM

CQVCGAKSSGLHFGCITCEGCKGFFRRMIKFKGSLVCFNDNKCKLDLKNRSTCKSCRMNRCLSVGM

**>Sme2DBDa3** (nhr-6) (scaffold:SmedGD_c1.3:v31.020066)

CQVCNCLAAGFHHGAYVCEACKKFFKRYSTNQLNSKVVCKKNCDLEKIGRRKCSFCRYQKCLNVGM

CRVCGGKSSGFHFGSLTCEGCKGFFRRAEPLKDKLECVSGKLNCVIDSSHKMPCKWCRYHRCIKVGM

**>Sme2DBD4** (nhr-1) (scaffold:SmedGD_c1.3:v31.000154)

CDVCGDVSAGFHCSAFVCEACKKFFIRSSKGDSYTKYSCTKNNNCEIVKDTRTHCQYCRFQKCLHLGM

CRVCGAQSSGFHFGAITCEGCKGFFRRTINERENQKYTCRNGGNCVINLATRNNCKSCRYKKCLAMGM

**>SmeNR1a** (scaffold:SmedGD_c1.3:v31.010919)

CRICSDTASGIHYGVMTCEGCKGFFQRNYKKINLKCYKNGNCSISKETRRLCQACRFRKCKNQGM

**>SmeNR1b** (nhr-10) (scaffold:SmedGD_c1.3:v31.026178)

CKVCGDVSSGIHYGVQTCEGCKGFFRRALIDDNHKKMHCINNRTCPVLPGTRNRCQYCRLLKCLRLGM

**>SmeNR2Un** (nhr-15) (scaffold:SmedGD_c1.3:v31.011761)

CAVCQDKATGLHYSVMTCEGCKGFFKRSIQAEKAYKCRNNNNCVINKETRNACQACRIRRCFEKGM

**28. NRs in *Spirometra erinaceieuropaei* (21)(prjeb1202)**

**>SeTR** (scaffold:S_erinaceieuropaei_v1_0_4:SPER_scaffold0040350)

CVVCGDNATGFHYRAMTCEGCKGFFRRSVQKKLEYTCKFNGRCSVADKQNRNSCQKCRFDRCIKGGM

**>SeE78** (scaffold:S_erinaceieuropaei_v1_0_4:SPER_contig0053909)

CKVCGDKASGYHYGVVSCEGCK

**>SeHR96a** (caffold:S_erinaceieuropaei_v1_0_4:SPER_scaffold0212654, SPER_scaffold0184453)

CVVCGEPASGYNFDRLTCESCKAFFRRNALKPRDKIKACGRSGDCNIEGSQRKHCPSCRLEKCLAVGM

**>SeHR96b** (scaffold:S_erinaceieuropaei_v1_0_4:SPER_scaffold0159348, SPER_scaffold0039939)

CVVCGEPASGYNFDRLTCESCKAFFRRNALKPRDKELTCTSKTGEHIVSPTTRRECPACRLKRCFLIGM

**>SeHR96c** (scaffold:S_erinaceieuropaei_v1_0_4:SPER_contig0109014, SPER_scaffold0057227)

CAVCGDHAVGYNFGAIACESCKAFFRRNALRATMPSCLFSGKCSIQVKTRRFCSPCRLEKCFAVGM

**>SeNR1a** (scaffold:S_erinaceieuropaei_v1_0_4:SPER_scaffold0078608, SPER_scaffold0001385)

CTVCGDRASGTHYGAWTCEGCKGFFRRSVQRKAAYFCVRNNQCDITRTLRNRCQKCRFVKCLRMGM

**>SeNR1b** (scaffold:S_erinaceieuropaei_v1_0_4:SPER_scaffold0002617, SPER_contig0084304)

CLVCGDTASGVHYGVLSCEGCKGFFRRTLQDKRSQPKQCLRDGLCRITVKSRNNCQPCRLRRCMELGM

**>SeRXR1** (scaffold:S_erinaceieuropaei_v1_0_4:SPER_scaffold0025940)

CGVCGDRATGRHYGVLSCEGCKGFFKRTIRRGAQYVCKEAGCCRIDRSQRPRCQYCRFRQCLAVGM

**>SeHNF4** (scaffold:S_erinaceieuropaei_v1_0_4:SPER_scaffold0002127)

YGAYSCDGCKGFFRRSVRRKHTYSCRYNRSCVIDKDMRNQCRFCRLKKCFRVGM

**>SeCoup-TFI** (scaffold:S_erinaceieuropaei_v1_0_4:SPER_contig0150549)

CVVCGDKSSGKHYGQHTCEGCKSFFKRSVRRKLVYKCRGNRQCPVDMHHRNQCQHCRFQKCLKTGM

**>SeCoupUn** (scaffold:S_erinaceieuropaei_v1_0_4:SPER_scaffold0093776)

VCGDESRGRHYGQYVCEACKSFFKRSVRKGPAYFCLRGGTCAIDARLRNRCRHCRWQRCLSAGM

**>SePNR** (scaffold:S_erinaceieuropaei_v1_0_4:SPER_scaffold0105089, SPER_contig0179980)

CLVCGDVSSGKHYGIFACNGCSGFFKRSVRRRLVYRCQAGTSRCVIDKERRNQCQACRLRKCFQMGM

**>SeTR4** (scaffold:S_erinaceieuropaei_v1_0_4:SPER_contig0061700)

GRHYGVLSCEGCKGFFKRSIRGHVNYICRTKQRCIVNKAYRNRCQYCRLQKCLQVGM

**>SeTLL** (scaffold:S_erinaceieuropaei_v1_0_4:SPER_contig0103798)

CKVCKDHSSGKHYGIYACDGCAGFFKRSIRRNRQYSCKSRNGTEASCRIDKPHRNQCRACRLKKCLDAGM

**>SeNR4A** (scaffold:S_erinaceieuropaei_v1_0_4:SPER_contig0052123, SPER_scaffold0173239)

CLVCGDNAACQHYGVRTCEGCKGFFKRTIQKNAHYVCLQSKNCVVDKRRRNRCQYCRFQKCLKVGM

**>SeFTZ-F1a** (scaffold:S_erinaceieuropaei_v1_0_4:SPER_contig0031144)

CPVCGDKVSGYHYGLPTCESCKGFFKRTVQNKKEYHCTDTGTCTIDRVHRKRCAHCRFQKCLSVGM

**>SeFTZ-F1b** (scaffold:S_erinaceieuropaei_v1_0_4:SPER_scaffold0057587)

CPICGDKISGYHYGIFCCESCKGFFKRTVQNAKRYACHMATPSSLCEITAASRKKCPAC

**>Se2DBDg** (scaffold:S_erinaceieuropaei_v1_0_4:SPER_contig0014964, SPER_contig0069084, SPER_contig0007298)

CDICGDVSAGFHCNAYVCEACKKFFIRSSKNENYAKYSCSKVNACEVNKDTRTHCQRCRYQKCLRIGM

CRVCGAQSSGFHFGAITCEGCKGFFRRTISERDNQRYTCRNGGTCLITLATRNNCKSCRYRKCLSVGM

**>Se2DBDa** (scaffold:S_erinaceieuropaei_v1_0_4:SPER_contig0017083, SPER_contig0105146, SPER_contig0012002)

KFFMRQSLSNGKSTTVCPSGGNCVIAKTSRGRCQQCRYKKCLELGM

CRVCGGRSSGFHFGALTCEGCKGFFRRTEETSSRLVCVGGKDNCAITPRSRNACKACRFRRCLRAGM

**>Se2DBDb** (scaffold:S_erinaceieuropaei_v1_0_4:SPER_contig0064610, SPER_scaffold0176443, SPER_contig0121722)

RGKCPHCRFRKCLDLGM

CRVCGSPSSGFHFGAITCEGCK>GFFRRTALSNVKLECLGKHNCCITPANRNMCKSCRFQRCLAVGM

**29. NRs in *Taenia asiatica* (17) (prjna299871)**

**>TaTR** (scaffold:Taenia_asiatica_TASYD01_v1:Scaffold00070)

CVVCGDNATGFHYRAMTCEGCKGFFRRSVQKKLEYTCKFNGNCSVGDKQNRNSCQKCRFERCLKGGM

**>TaE78** (scaffold:Taenia_asiatica_TASYD01_v1:Scaffold00156:3308)

CQVCGDKASGYHYGVISCEGCKGFFRRSIQKQIEYKCLREGKCTVVRLNRNRCQYCRFRKCIVAGM

**>TaHR96a** (scaffold:Taenia_asiatica_TASYD01_v1:Scaffold00034)

CVVCGEPASGYNFDRLTCESCKAFFRRNALKPKEKIKACGRNGDCNIEGSQRKHCPSCRLEKCLAVGM

**>TaHR96b** (scaffold:Taenia_asiatica_TASYD01_v1:Scaffold00039)

CRVCGDRAVNHNFGQLTCESCKAFFRRNAHKALPSIIAALRSSGDATTTDAYLQALRRGLRDLTCTSKSGEHVVSPSTRRECPACRLKRCFLIGM

**>TaHR96c** (scaffold:Taenia_asiatica_TASYD01_v1:Scaffold00010)

CAVCGDHAVGFNFGAIACESCKAFFRRNALRATMPPCLFNGNCLIQVKTRRFCSPCRLSKCFAVGM

**>TaNR1b** (scaffold:T_asiatica_South_Korea_v1_0_4:TASK_scaffold0000387)

CLVCGDTASGIHYGVLSCEGCKGFFRRALQDKRAHLRKCIRQDRCKISIKTRNHCQPCRLRRCLELGM

**>TaHNF4** (scaffold:Taenia_asiatica_TASYD01_v1:Scaffold00062)

CLICGDKATVLIAFQKGKHYGAYSCDGCKGFFRRSVRRKHTYSCRYNRTCTIDKDMRNQCRFCRLKKCFRVGM

**>TaTR4** (scaffold:T_asiatica_South_Korea_v1_0_4:TASK_scaffold0000334)

CLVCGDVASGRHYGVISCEGCKGFFKRSIRGHVNYVCRSNKHCIVNKAFRNRCQYCRMQKCLLVGM

**>TaTLL** (scaffold:T_asiatica_South_Korea_v1_0_4:TASK_scaffold0000088)

CKVCKDHSSGKHYGIYACDGCAGFFKRSIRRNRQYTCKSKNGQESSCRVDKPHRNQCRACRLKKCLEAGM

**>TaCoup-TFI** (scaffold:Taenia_asiatica_TASYD01_v1:Scaffold00001)

CVVCGDKSSGKHYGQFTCEGCKSFFKRSVRRQLTYTCRGARQCLIDLHHRNQCQYCRFQKCLKMGM

**>TaNR4A** (scaffold:Taenia_asiatica_TASYD01_v1:Scaffold00008)

CLVCGDNAACQHYGVRTCEGCKGFFKRTIQKNAHYVCLQTKNCIVDKRRRNRCQYCRFQKCLKVGM

**>TaFTZ-F1a** (scaffold:T_asiatica_South_Korea_v1_0_4:TASK_contig0000239)

CPVCGDKVSGYHYGLQTCESCKGFFKRTVQNKKSYQCTEAGVCIIDRIHRKRCAYCRFKKCLSVGM

**>TaFTZ-F1b** (scaffold:Taenia_asiatica_TASYD01_v1:Scaffold00001)

CPICGDKISGYHYGIFCCESCKGFFKRTVQNSKRYACHMATPSSLCDITATSRKKCPACRFVKCLEKGM

**>Ta2DBDa** (scaffold:Taenia_asiatica_TASYD01_v1:Scaffold00080)

CQICGELAAGFHHGAYVCEACKKFFMRHSLANGRSTTTCPSGGNCVIAKSSRGRCQQCRYKKCLEVGM

CRVCGGRSSGFHFGALTCEGCKGFFRRTEESASRLVCVGGKDNCTITPRSRNVCKACRFRRCLRAGM

**>Ta2DBDb** (scaffold:Taenia_asiatica_TASYD01_v1:Scaffold00009)

CQVCGQPSVGFHHRAYVCEACKKFFTRHLTNRIKKDKSANGECPMGGNCKIEGPGRGKCPHCRFRKCLDLGM

CRVCGGPSSGFHFGALTCEGCKGFFRRTVHSNSIPQCLGNQTCRITPSNRNMCKSCRFKKCLEVGM

**>Ta2DBDg** (scaffold:Taenia_asiatica_TASYD01_v1:Scaffold00003)

CDICGDVSAGFHCNAYVCEACKKFFIRSSKNENYTKYSCSKVNACEVNKDTRTHCQRCRFYKCLRIGM

CRVCGAQSSGFHFGAITCEGCKGFFRRTISERDNQRYTCRNGGTCLITLATRNNCKSCRYRKCLSVGM

**>TaOther** (scaffold:T_asiatica_South_Korea_v1_0_4:TASK_scaffold0000001)

CTVCGAPATDKHYGAVSCDSCRAFFEMATYLGLQSECSSEPNSCESDHHLRCHSCRLTRCLAVGM

**30. NRs in *Taenia multiceps* (17)(prjna307624)**

**>TmTR** (scaffold:T_multiceps_v2.0:LG2)

CVVCGDNATGFHYRAMTCEGCKGFFRRSVQKKLEYTCKFNGNCSVGDKQNRNSCQKCRFERCLKGGM

**>TmE78** (scaffold:T_multiceps_v2.0:LG1)

CQVCGDKASGYHYGVISCEGCKGFFRRSIQKQIEYKCLREGKCTVVRLNRNRCQYCRFRKCIVAGM

CVVCGEPASGYNFDRLTCESCKAFFRRNALKPKEKIKACGRNGDCNIEGSQRKHCPSCRLEKCLAVGM

**>TmHR96b** (scaffold:T_multiceps_v2.0:LG1)

CRVCGDRAVNHNFGQLTCESCKAFFRRNAHKALPSIIAALRSSGDATTTDAYLQALRRGLRDLTCTSKSGEHVVSPSTRRECPACRLKRCFLIGM

**>TmHR96c** (scaffold:T_multiceps_v2.0:LG1)

CAVCGDHAVGFNFGAIACESCKAFFRRNALRATMPPCLFNGNCLIQVKTRRFCSPCRLSKCFAVGM

**>TmHR96a** (scaffold:T_multiceps_v2.0:LG3)

**>TmNR1b** (scaffold:T_multiceps_v2.0:LG3)

CLVCGDTASGIHYGVLSCEGCKGFFRRALQDKRAHLRKCIRQDRCKISIKTRNHCQPCRLRRCLELGM

**>TmHNF4** (scaffold:T_multiceps_v2.0:LG6)

CLICGDKATGKHYGAYSCDGCKGFFRRSVRRKHTYSCRYNRTCTIDKDMRNQCRFCRLKKCFRVGM

**>TmTR4** (scaffold:T_multiceps_v2.0:LG1)

CLVCGDVASGRHYGVISCEGCKGFFKRSIRGHVNYVCRSNKHCIVNKAFRNRCQYCRMQKCLLVGM

**>TmTLL** (scaffold:T_multiceps_v2.0:LG5)

CKVCKDHSSGKHYGIYACDGCAGFFKRSIRRNRQYTCKSRNGQESCCRVDKPHRNQCRACRLKKCLEAGM

**>TmCoup-TFI** (scaffold:T_multiceps_v2.0:LG1)

CVVCGDKSSGKHYGQFTCEGCKSFFKRSVRRQLTYTCRGARQCLIDLHHRNQCQYCRFQKCLKMGM

**>TmNR4A** (scaffold:T_multiceps_v2.0:LG1)

CLVCGDNAACQHYGVRTCEGCKGFFKRTIQKNAHYVCLQTKNCIVDKRRRRRNRCQYCRFQKCLKVGM

**>TmFTZ-F1a** (scaffold:T_multiceps_v2.0:LG1)

CPVCGDKVSGYHYGLQTCESCKGFFKRTVQNKKSYQCTEAGVCIIDRIHRKRCAYCRFKKCLSVGM

**>TmFTZ-F1b** (scaffold:T_multiceps_v2.0:LG1)

CPICGDKISGYHYGIFCCESCKGFFKRTVQNSKRYACHMATPSSLCDITATSRKKCPACRFVKCLEKGM

**>Tm2DBDa** (scaffold:T_multiceps_v2.0:LG4)

CQICGELAAGFHHGAYVCEACKKFFMRHSLANGRSTTTCPSGGNCVIAKSSRGRCQQCRYKKCLEVGM

CRVCGGRSSGFHFGALTCEGCKGFFRRTEESASRLVCVGGKDNCTITPRSRNVCKACRFRRCLRAGM

**>Tm2DBDb** (scaffold:T_multiceps_v2.0:LG1)

CQVCGQPSVGFHHRAYVCEACKKFFTRHLTNRIKKDKSANGEGDSAKSSTQGGEIYPDLNVVCPMGGNCKIEGPGRGKCPHCRFRKCLDLGM

CRVCGGPSSGFHFGALTCEGCKGFFRRTVHSNSIPQCLGNQTCRITPSNRNMCKSCRFKKCLEVGM

**>Tm2DBDg** (scaffold:T_multiceps_v2.0:LG1)

CDICGDVSAGFHCNAYVCEACKKFFIRSSKNENYTKYSCSKVNACEVNKDTRTHCQRCRFYKCLRIGM

CRVCGAQSSGFHFGAITCEGCKGFFRRTISERDNQRYTCRNGGTCLITLATRNNCKSCRYRKCLSVGM

**>TmOther** (scaffold:T_multiceps_v2.0:LG1)

CTVCGAPATDKHYGAVSCDSCRAFFEMATYLGLQFECSGEPNSCESDHHLRCHSCRLTKCLAVGM

**31. NRs in *Taenia saginata* (17)(prjna71493)**

**>TsTR** (scaffold:ASM169307v2:Scaffold00112)

CVVCGDNATGFHYRAMTCEGCKGFFRRSVQKKLEYTCKFNGNCSVGDKQNRNSCQKCRFERCLKGGM

**>TsE78** (scaffold:ASM169307v2:Scaffold00015)

CQVCGDKASGYHYGVISCEGCKGFFRRSIQKQIEYKCLREGKCTVVRLNRNRCQYCRFRKCIVAGM

**>TsHR96a** (scaffold:ASM169307v2:Scaffold00011)

CVVCGEPASGYNFDRLTCESCKAFFRRNALKPKEKIKACGRNGDCNIEGSQRKHCPSCRLEKCLAVGM

**>TsHR96b** (scaffold:ASM169307v2:Scaffold00018)

CRVCGDRAVNHNFGQLTCESCKAFFRRNAHKALPSIIAALRSSGDATTTDAYLQALRRGLRDLTCTSKSGEHVVSPSTRRECPACRLKRCFLIGM

**>TsHR96c** (scaffold:ASM169307v2:Scaffold00067)

CAVCGDHAVGFNFGAIACESCKAFFRRNALRATMPPCLFNGNCLIQVKTRRFCSPCRLSKCFAVGM

**>TsNR1b** (scaffold:ASM169307v2:Scaffold00048)

CLVCGDTASGIHYGVLSCEGCKGFFRRALQDKRAHLRKCIRQDRCKISIKTRNHCQPCRLRRCLELGM

**>TsHNF4** (scaffold:ASM169307v2:Scaffold00043)

CLICGDKATGKHYGAYSCDGCKGFFRRSVRRKHTYSCRYNRTCTIDKDMRNQCRFCRLKKCFRVGM

**>TsTLL** (scaffold:ASM169307v2:Scaffold00039)

CKVCKDHSSGKHYGIYACDGCAGFFKRSIRRNRQYTCKSRNGQESCCRVDKPHRNQCRACRLKKCLEAGM

**>TsCoup-TFI** (scaffold:ASM169307v2:Scaffold00031)

CVVCGDKSSGKHYGQFTCEGCKSFFKRSVRRQLTYTCRGARQCLIDLHHRNQCQYCRFQKCLKMGM

**>TsTR4** (scaffold:ASM169307v2:Scaffold00001)

CLVCGDVASGRHYGVISCEGCKGFFKRSIRGHVNYVCRSNKHCIVNKAFRNRCQYCRMQKCLLVGM

**>TsNR4A** (scaffold:ASM169307v2:Scaffold00002)

CLVCGDNAACQHYGVRTCEGCKGFFKRTIQKNAHYVCLQTKNCIVDKRRRNRCQYCRFQKCLKVGM

**>TsFTZ-F1a** (scaffold:ASM169307v2:Scaffold00019)

CPVCGDKVSGYHYGLQTCESCKGFFKRTVQNKKSYQCTEAGVCIIDRIHRKRCAYCRFKKCLSVGM

**>TsFTZ-F1b** (scaffold:ASM169307v2:Scaffold00180)

CPICGDKISGYHYGIFCCESCKGFFKRTVQNSKRYACHMATPSSLCDITATSRKKCPACRFVKCLEKGM

**>Ts2DBDa** (scaffold:ASM169307v2:Scaffold00032)

CQICGELAAGFHHGAYVCEACKKFFMRHSLANGRSTTTCPSGGNCVIAKSSRGRCQQCRYKKCLEVGM

CRVCGGRSSGFHFGALTCEGCKGFFRRTEESASRLVCVGGKDNCTITPRSRNVCKACRFRRCLRAGM

**>Ts2DBDb** (scaffold:ASM169307v2:Scaffold00045)

CQVCGQPSVGFHHRAYVCEACKKFFTRHLTNRIKKDKSANGEGDSAKSSIQGGEIYPDLNVVCPMGGNCKIEGPGRGKCPHCRFRKCLDLGM

CRVCGGPSSGFHFGALTCEGCKGFFRRTVHSNSIPQCLGNQTCRITPSNRNMCKSCRFKKCLEVGM

**>Ts2DBDg** (scaffold:ASM169307v2:Scaffold00001)

CDICGDVSAGFHCNAYVCEACKKFFIRSSKNENYTKYSCSKVNACEVNKDTRTHCQRCRFYKCLRIGM

CRVCGAQSSGFHFGAITCEGCKGFFRRTISERDNQRYTCRNGGTCLITLATRNNCKSCRYRKCLSVGM

**>TsOther** (scaffold:ASM169307v2:Scaffold00010)

CTVCGAPATDKHYGAVSCDSCRAFFEMATYLGLQSECSSEPNSCESDHHLRCHSCRLTKCLAVGM

**32. NRs in *Taenia solium* (17)(prjna170813)**

**>TsoTR** (scaffold:Tsolium_Mexico_v1:pathogen_TSM_contig_00578)

CVVCGDNATGFHYRAMTCEGCKGFFRRSVQKKLEYTCKFNGNCSVGDKQNRNSCQKCRFERCLKGGM

**>TsoE78** (scaffold:Tsolium_Mexico_v1:pathogen_TSM_contig_00234)

CQVCGDKASGYHYGVISCEGCKGFFRRSIQKQIEYKCLREGKCTVVRLNRNRCQYCRFRKCIVAGM

**>TsoHR96a** (scaffold:Tsolium_Mexico_v1:pathogen_TSM_contig_00024)

CVVCGEPASGYNFDRLTCESCKAFFRRNALKPKEKIKACGRNGDCNIEGSQRKHCPSCRLEKCLAVGM

**>TsoHR96b** (scaffold:Tsolium_Mexico_v1:pathogen_TSM_contig_00217)

CRVCGDRAVNHNFGQLTCESCKAFFRRNAHKALPSIIAALRSSGDATTTDAYLQALRRGLRDLTCTSKSGEHVVSPSTRRECPACRLKRCFLIGM

**>TsoHR96c** (scaffold:Tsolium_Mexico_v1:pathogen_TSM_contig_00070)

CAVCGDHAVGFNFGAIACESCKAFFRRNALRATMPPCLFNGNCLIQVKTRRFCSPCRLSKCFAVGM

**>TsoNR1b** (scaffold:Tsolium_Mexico_v1:pathogen_TSM_scaffold_00486)

CLVCGDTASGIHYGVLSCEGCKGFFRRALQDKRAHLRKCIRQDRCKISIKTRNHCQPCRLRRCLELGM

**>TsoHNF4** (scaffold:Tsolium_Mexico_v1:pathogen_TSM_contig_00785)

CLICGDKATGKHYGAYSCDGCKGFFRRSVRRKHTYSCRYNRTCTIDKDMRNQCRFCRLKKCFRVGM

**>TsoCoup-TFI** (scaffold:Tsolium_Mexico_v1:pathogen_TSM_contig_02347)

CVVCGDKSSGKHYGQFTCEGCKSFFKRSVRRQLTYTCKGARQCLIDQHHRNQCQYCRFQKCLKMGM

**>TsoTR4** (scaffold:Tsolium_Mexico_v1:pathogen_TSM_contig_00118)

CLVCGDVASGRHYGVISCEGCKGFFKRSIRGHVNYVCRSNKHCIVNKAFRNRCQYCRMQKCLLVGM

**>TsoTLL** (scaffold:Tsolium_Mexico_v1:pathogen_TSM_contig_00249)

CKVCKDHSSGKHYGIYACDGCAGFFKRSIRRNRQYTCKSRNGQESCCRVDKPHRNQCRACRLKKCLEAGM

**>TsoNR4A** (scaffold:Tsolium_Mexico_v1:pathogen_TSM_contig_00138)

CLVCGDNAACQHYGVRTCEGCKGFFKRTIQKNAHYVCLQTKNCIVDKRRRNRCQYCRFQKCLKVGM

**>TsoFTZ-F1a** (scaffold:Tsolium_Mexico_v1:pathogen_TSM_contig_01242)

CPVCGDKVSGYHYGLQTCESCKGFFKRTVQNKKSYQCTEAGVCIIDRIHRKRCAYCRFKKCLSVGM

**>TsoFTZ-F1b** (scaffold:Tsolium_Mexico_v1:pathogen_TSM_contig_00358)

CPICGDKISGYHYGIFCCESCKGFFKRTVQNSKRYACHMATPSSLCDITATSRKKCPACRFVKCLEKGM

**>Tso2DBDa** (scaffold:Tsolium_Mexico_v1:pathogen_TSM_contig_00247)

CQICGELAAGFHHGAYVCEACKKFFMRHSLANGRSTTTCPSGGNCVIAKSSRGRCQQCRYKKCLEVGM

CRVCGGRSSGFHFGALTCEGCKGFFRRTEESASRLVCVGGKDNCTITPRSRNVCKACRFRRCLRAGM

**>Tso2DBDb** (scaffold:Tsolium_Mexico_v1:pathogen_TSM_contig_00072)

CQVCGQPSVGFHHRAYVCEACKKFFTRHLTNRIKKDKSTNGEGDSAKSSTQGGEIYPDLNVVCPMGGNCKIEGPGRGKCPHCRFRKCLDLGM

CRVCGGPSSGFHFGALTCEGCKGFFRRTVHSNSIPQCLGNQTCRITPSNRNMCKSCRFKKCLEVGM

**>Tso2DBDg** (scaffold:Tsolium_Mexico_v1:pathogen_TSM_contig_00008)

CDICGDVSAGFHCNAYVCEACKKFFIRSSKNENYTKYSCSKVNACEVNKDTRTHCQRCRFYKCLRIGM

CRVCGAQSSGFHFGAITCEGCKGFFRRTISERDNQRYTCRNGGTCLITLATRNNCKSCRYRKCLSVGM

**>TsoOther** (scaffold:Tsolium_Mexico_v1:pathogen_TSM_contig_00028)

CKVCGAPATDKHYGAVSCDSCRAFFGMATYLGIQSKCSNEPNSCDNDHHLRCHSCRLTKCLAVGM

**33. NRs in *Trichobilharzia regent* (21)(prjeb4662)**

**>TrTRa** (scaffold:T_regenti_v1_0_4:TRE_scaffold0018554)

CVVCGDNATGFHYRAMTCEGCKGFFRRSVQKKLVYTCKFNGRCSVSDKQNRNSCQKCRFDRCIKGGM

**>TrTRb** (scaffold:T_regenti_v1_0_4:TRE_scaffold0042270)

CVVCGDNATGFHYRAMTCEGCKGFFRRSVQKKLVYTCKFQGRCSVADKQNRNSCQKCRFDRCIKGGM

**>TrE78** (scaffold:T_regenti_v1_0_4:TRE_contig0001566, TRE_scaffold0003880)

CKVCGDKASGYHYGVISCEGCKGFFRRSIQKQIEYKCLRDGKCLVIRLNRNRCQYCRFRKCLAAGM

**>TrHR96a** (scaffold:T_regenti_v1_0_4:TRE_contig0000708)

CIVCGEPASGYNFDRLTCESCKAFFRRNALKPRDK

**>**Tr**HR96b** (scaffold:T_regenti_v1_0_4:TRE_contig0011335, TRE_scaffold0022804)

CKVCGDKAVNHNFXXXXXXXxxxxxxxxxxxELTCTAKSGEHIITPTTRRECPSCRLKQCFRVGM

**>TrNR1a** (scaffold:T_regenti_v1_0_4:TRE_scaffold0003931)

CVVCGDKSSGFHYGVITCEGCKGFFRRAIQGNQSYTCSRDGTCEVNKTLRNKCQQCRLVKCIAVGM

**>TrCoup-TFI** (dna:scaffold scaffold:T_regenti_v1_0_4:TRE_scaffold0032905)

CVVCGDKSSGKHYGQHTCEGCKSFFKRSVRRKLNYTCRGNRQCPIDIHHRNQCQYCRFQKCIKVGM

**>TrCoup-TFII** (scaffold:T_regenti_v1_0_4:TRE_scaffold0019282)

VCGDRSSGKHYGQYTCEGCKSFFKRSVRKSASYICRSAGQCPVDAQRRNQCQACRMSRCLLAGM

**>TrTR4** (scaffold:T_regenti_v1_0_4:TRE_scaffold0014429)

GRHYGVVSCEGCKGFFKRSIRGHVSYVCRSEQNCLVNKAYRNRCQYCRLQKCLAVGM

**>TrRXR1** (scaffold:T_regenti_v1_0_4:TRE_scaffold0045763)

CMICGDKATGKHYGAISCEGCKGFFKRTVRKHLEYVCRVAGQCPVDKRKRTRCQYCRFEQCLAKGM

**>TrRXR2** (scaffold:T_regenti_v1_0_4:TRE_scaffold0015179, TRE_scaffold0016580)

CSICSDRASGKHYGVISCEGCKGFFKRTVRKELTYICRDNQDCQIDKRLRNRCQYCRYQKCLRVGM

**>TrPNR** (scaffold:T_regenti_v1_0_4:TRE_scaffold0001209)

CIVCGDISSGKHYGILACNGCSGFFKRSVRRKLIYRCQAGNGLCIIDKAHRNQCQACRLKKCIRMGM

**>TrTLL** (scaffold:T_regenti_v1_0_4:TRE_scaffold0020872)

CHVCEDHSSGKHYGIYACDGCAGFFKRSIRRNRQYTCKSRGTTKPGMVICRVDKSHRNQCRACRLTKCLEVGM

**>TrHNF4** (scaffold:T_regenti_v1_0_4:TRE_scaffold0018763)

GKHYGAYSCDGCKGFFRRSVRRKHSYTCRHKRSCIVTKDKRNQCRFCRFRKCFRVGM

**>TrDSF** (scaffold:T_regenti_v1_0_4:TRE_scaffold0017076)

CGVCGDRSTGKHYGVYSCDGCSGFFKRSIHKNRTYTCKASGNLKGQCTIDRNRRNHCRACRLNKCFMAQM

**>TrNR4A** (scaffold:T_regenti_v1_0_4:TRE_scaffold0001610, TRE_scaffold0000439)

CLVCGDNAACQHYGVRTCEGCKGFFKRTIQKNAQYVCLQAKNCVVDKRRRNRCQYCRFQKCLKVGM

**>TrFTZ-F1a** (scaffold:T_regenti_v1_0_4:TRE_scaffold0008942)

CPICGDKVSGYHYGLPTCESCKGFFKRTVQNKKEYHCNEKGRCVVDRQHRKRCAYCRFQKCLNVGM

**>TrFTZ-F1b** (scaffold:T_regenti_v1_0_4:TRE_scaffold0071294)

CPICGDKISGYHYGIFCCESCKGFFKRTVQNAKRYACHRPNAASRCEININTRKKCPACRFLKCVDKGM

**>Tr2DBDa** (scaffold:T_regenti_v1_0_4:TRE_scaffold0026560, TRE_scaffold0022260, TRE_scaffold0022927)

CQVCGELAAGFHHGAYVCEACKKFFMRHSMADTKPTNVCPTGGNCIVAKGSRGKCQICRYRK

CRVCGGRSSGFHFGALTCEGCKGFFRRTEGSANSLVCVGGQNACTITPRSRNACKSCRFRRCLTAGM

**>Tr2DBDb** (scaffold:T_regenti_v1_0_4:TRE_scaffold0004177, TRE_scaffold0034172, TRE_scaffold0034172, TRE_scaffold0003842)

CQICGQPAVGFHHRAYVCEACKKFFMRHTAARLRNSEMGSTVTXXXXXMGGRCRVEGPGRGKCPHCRYRKCLDLGM

CRVCSGPSSGFHFGALTCEGCKGFFRRTVLSNVRLECLGNNDCPITPANRNMCKSCRFQRCLAVGM

**>Tr2DBDg** (scaffold:T_regenti_v1_0_4:TRE_scaffold0027404, TRE_scaffold0104990, TRE_scaffold0086818)

CDICGDVAAGFHCNAYVCEACK

CRVCGAKSSGFHFGAITCEGCKGFFRRTINERESQRYTCRNGGNCAVTGATRNNCKSCRYRRCLAVGM
