# Supplementary material for: Identification and evolution of nuclear receptors in Platyhelminths
Source: PLoS One. 2021 Aug 13;16(8):e0250750. doi: 10.1371/journal.pone.0250750 (PMC8363021; doi:10.1371/journal.pone.0250750)
Supplement: S3 File — (DOCX) [file pone.0250750.s029.docx]

**S3 File. Comparison of Bayesian inference and Maximum Likelihood method to analysis of NR DBD amino sequences (ML bootstrap value is shown first and Bayesian posterior probability is shown after slash)**

| Group | Gene | P-Box | *G.*  *salaris* | *P.*  *xenopodis* | *H.*  *microstoma* | *M.*  *corti* | *E.*  *multilocularis* | *T saginata* | *H.*  *taeniaeformis* | *M.*  *corti* |
| --- | --- | --- | --- | --- | --- | --- | --- | --- | --- | --- |
| NR0A | Knr | CEGCKSFFGR | 984/1 | 955/1 | 995/1 | 998/1 | 939/1 | 973/1 | 921/1 | 998/1 |
| NR1A | TR | CEGCKGFFRR | 869/1 | 988/1 | 870/1 | -/0.85 | 801/0.98 | 979/1 | -/0.75  952/1 | 849/1 |
| NR1B | RAR | CEGCKGFFRR | 907/1 | 944/1 | 915/1 | 955/1 | 911/1 | 912/1 | 926/1 | 955/1 |
| NR1E | E78 | CEGCKGFFRR | 859/1 | 851/0.99 | -/0.9 | -/0.97 | -/0.96 | 793/1 | 713/0.98 | -/0.97 |
| NR1F | ROR | CEGCKGFFRR | -/0.83 | -/0.85 | 725/0.89 | 772/0.96 | 716/0.9 | 724/0.92 | -/0.8 | 772/0.96 |
| NR1I | VDR | CEGCKGFFRR | -/0.99 | -/- | -/0.85 | -/0.79 | -/0.86 | -/0.88 | -/0.89 | -/0.79 |
| NR1J | HR96 | CESCKAFFRR | -/0.99 | -/1 | 753/1 | 777/1 | 760/1 | -/1 | 715/1 | 777/1 |
| NR2A | HNF4 | CDGCKGFFRR | 942/1 | 956/1 | 921/1 | 856/1 | 842/1 | 804/0.99 | 846/0.85 | 856/1 |
| NR2B | RXR | CEGCKGFFKR | -/0.71  -/0.86 | -/0.86 | -/0.63 | 793/0.91 | -/0.57 | -/0.54 | -/0.56 | -/0.58 |
| NR2D | TR4 | CEGCKGFFKR | -/0.83 | -/0.83 | -/0.74  841/1 | -/0.72  812/0.99 | -/0.71  777/1 | 771/0.82 | -/0.69  761/0.99 | 812/0.99 |
| NR2E2 | TLL | CDGCAGFFKR | 899/1 | 858/1 | 898/1 | 893/1 | 873/1 | 857/0.98 | 912/1 | 893/1 |
| NR2E3 | PNR | CNGCSGFFKR | 767/1 | 860/1 | 871/1 | 879/1 | 865/1 | 843/1 | 841/0.99 | 879/1 |
| NR2E4 | DSF | CDGCSGFFKR | 898/1 | 851/1 | 878/1 | 867/1 | 831/1 | 830/1 | 783/0.98 | 867/1 |
| NR2E5 | fax1 | CDGCSCFFKR | 929/1 | 913/1 | 926/1 | 925/1 | 929/1 | 941/1 | 940/1 | 925/1 |
| NR2E6 | NHR236 | CDGCRGFFKR | 844/0.99 | 814/1 | 911/1 | 890/1 | 853/0.99 | 824/0.97 | 891/1 | 890/1 |
| NR2F | COUP-TF | CEGCKSFFKR | -/1 | -/0.98 | -/1 | -/1 | 836/1 | 836/1 | 812/1 | -/1 |
| NR3A | ER | CEGCKAFFKR | 899/0.99 | 858/0.99 | 906/0.99 | 875/0.97 | 896/0.98 | 899/0.99 | 898/1 | 875/0.98 |
| NR3B | ERR | CEACKAFFKR | 867/0.97 | 894/0.99 | 894/0.94 | 898/0.97 | 911/0.95 | 919/0.99 | 921/0.99 | 898/0.97 |
| NR3C | MR | CGSCKVFFKR | 939/1 | 980/1 | 994/1 | 983/1 | 896/0.98 | 961/1 | 971/1 | 983/1 |
| NR4A | NR4A | CEGCKGFFKR | 933/1 | 957/1 | 945/1 | 956/1 | 927/1 | 971/1 | 972/1 | 956/1 |
| NR5A | FTZ-F1 | CESCKGFFKR | -/0.51 | -/0.53  -/0.88 | -/0.98 | -/0.65 | 759/1 | -/0.99 | 675/0.97 | -/0.65 |
| NR5B | HR39 | CESCKGFFKR | 906/0.95 | 992/1 | 985/1 | 986/1 | 994/1 | 995/1 | 990/1 | 986/1 |
| NR6 | NR6A | CEGCKGFFKR | 963/1 | 958/1 | 952/1 | 915/1 | 943/1 | 941/1 | 962/1 | 915/1 |
| NR7/8 |  | CEGCKGFFKR | -/0.92 | 718/1 | 787/1 | 832/1 | 757/1 | 762/0.99 | 761/1 | 832/1 |

| **Group** | **Gene** | **P-Box** | ***S.***  ***solidus*** | ***S.***  ***erinaceieuropaei*** | ***O.***  ***viverrini*** | ***E. caproni*** | ***F.***  ***hepatica*** | ***S.***  ***haematobium*** | ***T.***  ***regenti*** | ***M.***  ***lignano*** | ***S. mediterranea*** |
| --- | --- | --- | --- | --- | --- | --- | --- | --- | --- | --- | --- |
| NR0A | Knr | CEGCKSFFGR | 998/1 | 997/1 | 995/1 | 994/1 | 996/1 | 997/1 | 998/1 | 999/1 | 1000/1 |
| NR1A | TR | CEGCKGFFRR | 986/1 | 985/1 | 984/1 | -/0.83 | 741/0.85 | 835/0.99 | -/0.53  993/1 | 737/0.95 | -/0.73 |
| NR1B | RAR | CEGCKGFFRR | 944/1 | 903/1 | 906/1 | 902/1 | 925/1 | 898/1 | 907/1 | 921/1 | 916/1 |
| NR1E | E78 | CEGCKGFFRR | 803/1 | 818/0.99 | 839/0.99 | 845/1 | 870/1 | 876/1 | 889/1 | 764/0.99 | 891/1 |
| NR1F | ROR | CEGCKGFFRR | 738/0.95 | 733/0.81 | -/0.83 | -/0.93 | -/0.95 | -/0.95 | -/0.94 | -/0.97 | 723/0.94 |
| NR1I | VDR | CEGCKGFFRR | -/0.88 | -/0.65 | -/0.93 | -/0.94 | -/0.94 | -/0.94 | -/0.93 | -/0.89 | -/0.86 |
| NR1J | HR96 | CESCKAFFRR | 705/1 | 820/1 | -/1 | -/0.91 | -/1 | 713/1 | -/0.93 | 866/1 | -/1 |
| NR2A | HNF4 | CDGCKGFFRR | 921/1 | 926/1 | 968/1 | 974/1 | 962/1 | 944/1 | 953/1 | 959/1 | 947/1 |
| NR2B | RXR | CEGCKGFFKR | -/0.86 | -/0.56  -/0.92 | -/0.81 | -/0.67  971/1 | -/0.7  970/1 | -/0.95 | -/0.63  915/1  -/0.81 | -/0.84 | -/0.83 |
| NR2D | TR4 | CEGCKGFFKR | 872/1 | -/0.84 | -/0.96 | -/0.91 | -/0.94 | -/0.65 | -/0.89 | -/0.98 | -/0.96 |
| NR2E2 | TLL | CDGCAGFFKR | 806/1 | 824/1 | 895/1 | 910/1 | 951/1 | 919/1 | 830/1 | 882/1 | 909/1 |
| NR2E3 | PNR | CNGCSGFFKR | 816/1 | -/0.94 | 863/1 | 858/1 | 892/1 | 845/1 | 790/1 | 840/1 | 863/1 |
| NR2E4 | DSF | CDGCSGFFKR | 833/1 | 835/1 | 841/1 | 735/1 | 808/1 | 821/1 | 764/1 | 739/1 | 816/1 |
| NR2E5 | fax1 | CDGCSCFFKR | 933/1 | 941/1 | 939/1 | 934/1 | 937/1 | 938/1 | 941/1 | 958/1 | 934/1 |
| NR2E6 | NHR236 | CDGCRGFFKR | 843/0.99 | 859/1 | 882/1 | 867/0.99 | 854/0.99 | 853/0.99 | 869/1 | 856/1 | 780/1 |
| NR2F | COUP-TF | CEGCKSFFKR | -/1 | -/1 | -/1 | 829/1 | -/1 | -/0.98 | -/1 | -/1 | -/0.99 |
| NR3A | ER | CEGCKAFFKR | 890/0.99 | 882/0.99 | 946/0.99 | 874/0.98 | 874/0.98 | 886/0.98 | 872/0.99 | 909/0.99 | 757/0.99 |
| NR3B | ERR | CEACKAFFKR | 919/0.98 | 902/0.98 | 919/0.99 | 913/0.98 | 922/0.98 | 918/0.94 | 912/0.98 | -/0.51* | 822/0.95 |
| NR3C | MR | CGSCKVFFKR | 988/1 | 993/1 | 991/1 | 979/1 | 989/1 | 984/1 | 993/1 | 990/1 | 991/1 |
| NR4 | NR4A | CEGCKGFFKR | 930/1 | 930/1 | 969/1 | 938/1 | 937/1 | 931/1 | 952/1 | 813/1 | **867/1** |
| NR5A | FTZ-F1 | CESCKGFFKR | -/0.99 | -/0.98 | -/0.99 | -/0.93 | -/0.95 | -/0.98 | -/0.87 | -/0.98 | -/0.97 |
| NR5B | HR39 | CESCKGFFKR | 985/1 | 987/1 | 982/1 | 975/1 | 983/1 | 948/1 | 968/1 | -/1 | 925/1 |
| NR6 | NR6A | CEGCKGFFKR | 949/1 | 945/1 | 963/1 | 955/1 | 963/1 | 953/1 | 965/1 | 828/1 | -/0.93 |
| NR7/8 |  | CEGCKGFFKR | 766/1 | 767/1 | 755/0.99 | 799/1 | 762/1 | 734/0.99 | 776/1 | 771/1 | 771/0.99 |

Red letter in P-box sequence shows the amino acid unique to each NR gene/group/subfamily. Dash indicates ML bootstrap value is below 700 (70%). Red number shows ML bootstrap value and Bayesian posterior probability of each Platyhelminth NRs clustered with other Platyhelminth orthologues when Bayesian posterior probability is below 0.8.

* Clustered within NR3 subfamily with BPP 0.51, P-box same as ERR, may be a new group
