# Supplementary material for: Identification and evolution of nuclear receptors in Platyhelminths
Source: PLoS One. 2021 Aug 13;16(8):e0250750. doi: 10.1371/journal.pone.0250750 (PMC8363021; doi:10.1371/journal.pone.0250750)
Supplement: S4 File — (DOCX) [file pone.0250750.s030.docx]

**S4 File. Sequence alignment of DBD sequence of HR96s and intron in this regions (the red color of greater-than sign ( > ) indicates the intron position)**

**HR96a**

**Monogenea**

GsHR96a CVVCGEPASGYNFDRLTCESCKAFFRRNALKPRDK>IKKCNRHGQCNIEGSQRKHCPSCRLEKCLAAGM

PxHR96a CIVCGEPASGYNFDRLTCESCKAFFRRNALKPRDK>IKACSRGGGCNVEGGQRKHCPSCRLEKCLASGM

**Cestoda**

DlHR96a FRRNALKPRDK>IKACGRSGDCNIEGSQRKHCPSCRLEKCLAVGM

EmHR96a CVVCGEPASGYNFDRLTCESCKAFFRRNALKPKEK>IKACGRNGDCNIEGSQRKHCPSCRLEKCLAVGM

EcHR96a CVVCGEPASGYNFDRLTCESCKAFFRRNALKPKEK>IKACGRNGDCNIEGSQRKHCPSCRLEKCLAVGM

EgHR96a CVVCGEPASGYNFDRLTCESCKAFFRRNALKPKEK>IKACGRNGDCNIEGSQRKHCPSCRLEKCLAVGM

HaHR96a CVVCEEPASGYNFDRLTCESCKAFFRRNALKPKEK>IKACSRNGDCVIVGNQRKHCPSCRLEKCLAVGM

HdHR96a CVVCEEPASGYNFDRLTCESCKAFFRRNALKPKDK>IKACSRNGDCVVVGSQRKHCPSCRLEKCLAVGM

HtHR96a CVVCGEPASGYNFDRLTCESCKAFFRRNALKPKEK>IKACGRNGDCNIEGSQRKHCPSCRLEKCLAVGM

HmHR96a CVVCEEPASGYNFDRLTCESCKAFFRRNALKPKDK>IKACSRNGDCVVVGSQRKHCPSCRLEKCLAVGM

McHR96a CVVCGEPASGYNFDRLTCESCKAFFRRNALKPKEK>IKACGRNGDCNIEGSQRKHCPSCRLEKCLAVGM

SeHR96a CVVCGEPASGYNFDRLTCESCKAFFRRNALKPRDK>IKACGRSGDCNIEGSQRKHCPSCRLEKCLAVGM

SsHR96a CVVCGEPASGYNFDRLTCESCKAFFRRNALKPRDK>IKACGRSGDCNIEGSQRKHCPSCRLEKCLAVGM

TaHR96a CVVCGEPASGYNFDRLTCESCKAFFRRNALKPKEK>IKACGRNGDCNIEGSQRKHCPSCRLEKCLAVGM

TmHR96a CVVCGEPASGYNFDRLTCESCKAFFRRNALKPKEK>IKACGRNGDCNIEGSQRKHCPSCRLEKCLAVGM

TsoHR96a CVVCGEPASGYNFDRLTCESCKAFFRRNALKPKEK>IKACGRNGDCNIEGSQRKHCPSCRLEKCLAVGM

TsHR96a CVVCGEPASGYNFDRLTCESCKAFFRRNALKPKEK>IKACGRNGDCNIEGSQRKHCPSCRLEKCLAVGM

**Trematoda**

CsHR96a CIVCGEPASGYNFDRLTCESCKAFFRRNALKPKDK>IKVCSRGGGCIIEGNQRKHCPSCRLEKCFAVGM

EcaHR96a CIVCGEPASGYNFDRLTCESCKAFFRRNALKPKDK>IKACSRGGGCVIEGNQRKHCPSCRLEKCFNVGM

FhHR96a CIVCGEPASGYNFDRLTCESCKAFFRRNALKPKDK>IKACSRGGGCIIEGNQRKHCPSCRLEKCFAVGM

OvHR96a CIVCGEPASGYNFDRLTCESCKAFFRRNALKPKDK>IKVCSRGGGCIIEGNQRKHCPSCRLEKCFAVGM

OfHR96a CIVCGEPASGYNFDRLTCESCKAFFRRNALKPKDK>IKVCSRGGGCIIEGNQRKHCPSCRLEKCFAVGM

SmHR96a CIVCGEPASGYNFDRLTCESCKAFFRRNALKPRDK>IKACNRGGGCAIEGNQRKHCPSCRLEKCLAVGM

SmtHR96a CIVCSEPASGYNFDRLTCESCKAFFRRNALKPRDK>IKACNRGGGCAIEGNQRKHCPSCRLEKCLAVGM

SbHR96a CIVCSEPASGYNFDRLTCESCKAFFRRNALKPRDK>IKACNRGGGCAIEGNQRKHCPSCRLEKCLAVGM

SjHR96a CIVCGEAASGYNFDRLTCESCKAFFRRNALKPRDK>

SmaHR96a CIVCSEPASGYNFDRLTCESCKAFFRRNALKPRDK>IKACNRGGGCAIEGNQRKHCPSCRLEKCLAVGM

SrHR96a CIVCGEPASGYNFDRLTCESCKAFFRRNALKPRDK>IKACNRGGGCAIEGNQRKHCPSCRLEKCLAVGM

ScHR96a CIVCSEPASGYNFDRLTCESCKAFFRRNALKPRDK>

ShHR96a CIVCSEPASGYNFDRLTCESCKAFFRRNALKPILQ>IKACNRGGGCAIEGNQRKHCPSCRLEKCLAVGM

TrHR96a CIVCGEPASGYNFDRLTCESCKAFFRRNALKPRDK>

**Rhabditophora**

SmeHR96a CVVCGEKASGFNFDRLTCESCKAFFRRNAMKPKEK>MKPCPHGGGCNISGSQRKHCPYCRLEKCFTEGM

MlHR96-9 CAVCGDPASGYNFDRLTCESCKAFFRRNALKAREK-IKPCSRGGGCDVSGSQRKHCPTCRLEKCVRVGM (No intron)

MlHR96-10 CAVCAEPASGYNFDRLTCESCKAFFRRNALKSREK-IKPCSRGGGCDVSGSQRKHCPTCRLEKCLLVGM (No intron)

MlHR96-11 CAVCSEPATGYNFDRLTCESCKAFFRRNALKSRER-IKPCSRGGGCDVSGPQRKHCPTCRLEKCLRVGM (No intron)

1

**HR96c**

**Monogenea**

PxHR96c CSICGDKAVGYNFGAIACESCKAFFRRNALKTE>TPVCIFDRSCDIRTATRRFCSACRLAKCLKVGM

**Cestoda**

DlHR96c CAVCGDHAVGFNFGAIACESCKAFFxxxxxxxx>MPSCLFSGKCSIQVKTRRFCSPCRLEKCFAVGM

EcHR96c CAVCGDHAVGFNFGAIACESCKAFFRRNALRAT>TPPCLFNGSCLIQVKTRRFCSPCRLSKCFAVGM

EgHR96c CAVCGDHAVGFNFGAIACESCKAFFRRNALRAT>MPPCLFNGSCLIQVKTRRFCSPCRLSKCFAVGM

HdHR96c CAVCGDHAVGFNFGAIACESCKAFFRRNALRAS>MPSCLFNGSCMIQVKTRRFCSPCRLSKCFAVGM

EmHR96c CAVCGDHAVGFNFGAIACESCKAFFRRNALRAT>MPPCLFNGSCLIQVKTRRFCSPCRLSKCFAVGM

HmHR96c CAVCGDHAFGFNFGAIACESCKAFFRRNALRAS>MPPCLFNDSCMIQVKTRRFCSPCRLSKCFAVGM

HaHR96c CAVCGDHAFGFNFGAIACESCKAFFRRNALRAS>MPPCLFNDSCMIQVKTRRFCSPCRLSKCFAVGM

McNR96c CAVCGDHAVGFNFGAIACESCKAFFRRNALRVS>MPPCLFNANCLIQVKTRRFCSPCRLAKCFAVGM

SeHR96c CAVCGDHAVGYNFGAIACESCKAFFRRNALRAT>MPSCLFSGKCSIQVKTRRFCSPCRLEKCFAVGM

TaHR96c CAVCGDHAVGFNFGAIACESCKAFFRRNALRAT>MPPCLFNGNCLIQVKTRRFCSPCRLSKCFAVGM

TmHR96c CAVCGDHAVGFNFGAIACESCKAFFRRNALRAT>MPPCLFNGNCLIQVKTRRFCSPCRLSKCFAVGM

TsHR96c CAVCGDHAVGFNFGAIACESCKAFFRRNALRAT>MPPCLFNGNCLIQVKTRRFCSPCRLSKCFAVGM

TsoHR96c CAVCGDHAVGFNFGAIACESCKAFFRRNALRAT>MPPCLFNGNCLIQVKTRRFCSPCRLSKCFAVGM

**Trematoda**

CsHR96c CNVCGDAAMGFNFGAVTCESCKAFFRRTARKAQ>VANCVFNEKCTITVATRRFCSHCRLKKCFAAGM

EcaHR96c CSVCGDSAVGFNFGAIACESCKAFFRRSAHKAQ>VTTCLFNERCTIEVATRRFCSHCRLKKCFSVGM

FhHR96c CSVCGDSAVGFNFGAIACESCKAFFRRSAHKAQ>MTSCLFNERCTVDVPTRRFCSHCRLKKCFSVGM

OfHR96c CNVCGDAAMGFNFGAVTCESCKAFFRRTARKAQ>VANCVFNEKCTITVATRRFCSHCRLKKCFAAGM

OvHR96c CNVCGDAAMGFNFGAVTCESCKAFFRRTARKAQ>VANCVFNEKCTITVATRRFCSHCRLKKCFAAGM

SsHR96c CAVCGDHAVGYNFGAIACESCKAFFRRNALRPT>IPPCLFSGKCSIQVKTRRFCSPCRLEKCFAVGM

**Rhabditophora**

SmeHR96c CSVCGDKALGYNFGAITCESCKAFFRRNAHKKS>PPPCVFNKTCEISVNTRRFCTYCRLMKCFKMQM

MlHR96-7 CRVCGDKALGFNFDAISCESCKAFFRRNALKQD>VPACMFTQNCSVTVATRRFCTHCRLQKCIQVGM

MlHR96-8 CTICGDKALGYNFDAISCESCKAFFRRNALKAE>IPDCMFSQRCNVTVATRRFCTSCRLKKCLECGM

**HR96d**

**Rhabditophora**

MlHR96-4 CGVCGDKALGFNFDAVSCESCKAFFRRNAVKGV>EAFKCPYSGACSIDVSNRRFCKRCRLAKCFDIGM

MlHR96-6 CGVCGDKALGFNFDAVSCESCKAFFRRNAAKGV>EALKCPYEGNCRIDVSNRRFCKRCRLRKCFDVGM

MlHR96-16 CGVCGDKALGFNFDAVSCESCKAFFRRNAGRGV>SAFKCPYEGHCVMDVSNRRFCKHCRLRKCFDVGM

**Mollusca**

LgHR96-3 CGVCGDRALGYNFDAISCESCKAFFRRNAPKGL>DYFKCPYEEKCKMDVSNRRFCKRCRLRKCFEIGM

BgHR96-3 CGVCGDRALGYNFDAISCESCKAFFRRNAPKGL>EYFKCPYEEKCKMDVSNRRFCKRCRLRKCFEIGM

2

**HR96b**

**Monogenea**

PxHR96b CKVCGDKAVNHNFGQLTCESCKAFFRRNAHK>------------------------------------ELTCTSKVTGHVISPTTRRECPACRLKRCFVVGM

GsHR96b CKVCGDRAVNHNFGQLTCESCKAFFRRNAHK>------------------------------------ELTCTSKTNGHYISPSTRRECPACRLKRCFFVGM

**Cestoda**

DlHR96b --------------------------------------------------------------------ELTCTSKTGEHVVSPTTRRECPACRLKRCFLIGM

EcHR96b CRVCGDRAVNHNFGQLTCESCKAFFRRNAHK>ALPSISAALQSSDCATTTDAYLQALQRGLR>-----DLTCTSKSGEHVVSPSTRRECPACRLKRCFLIGM

EgHR96b CRVCGDRAVNHNFGQLTCESCKAFFRRNAHK>ALPSISAALQSSDCATTTDAYLQALQRGLR>-----DLTCTSKSGEHVVSPSTRRECPACRLKRCFLIGM

EmHR96b CRVCGDRAVNHNFGQLTCESCKAFFRRNAHK>ALPSISAALQSSDCATTTDAYLQALQRGLR>-----DLTCTSKSGEHVVSPSTRRECPACRLKRCFLIGM

HtHR96b CRVCGDRAVNHNFGQLTCESCKAFFRRNAHK>ALPSIIAALRSSGDATTTDAYLQALQRGLR>-----DLTCTSKSGEHVVSPSTRRECPACRLKRCFLIGM

HdHR96b CRVCGDRAVNHNFGQLTCESCKAFFRRNAHK>ALPSISAALQSSGSKSITMEVYIKAIQHGLR>----DLTCTSKSGEHVVSPSTRRECPACRLKRCFLIGM

HmHR96b CRVCGDRAVNHNFGQLTCESCKAFFRRNAHK>ALPSISAVLQSSGTKSITMEVYIKAIQYGLR>----DLTCTSKSGEHVVSPSTRRECPACRLKRCFLIGM

HaHR96b CRVCGDRAVNHNFGQLTCESCKAFFRRNAHK>ALPSISAVLQSSGTKSITMEVYIKAIQHGLR>----DLTCTSKSGEHVVSPSTRRECPACRLKRCFLIGM

McHR96b CRVCGDRAVNHNFGQLTCESCKAFFRRNAHK>ALPSINAALQSCAPGMPTSDAYVRAVRLGLS>----ELTCTSKSGEHVVSPSTRRECPACRLKRCFLIGM

SeHR96b CVVCGEPASGYNFDRLTCESCKAFFRRNALKPRDK>--------------------------------ELTCTSKTGEHIVSPTTRRECPACRLKRCFLIGM

TaHR96b CRVCGDRAVNHNFGQLTCESCKAFFRRNAHK>ALPSIIAALRSSGDATTTDAYLQALRRGLR>-----DLTCTSKSGEHVVSPSTRRECPACRLKRCFLIGM

TmHR96b CRVCGDRAVNHNFGQLTCESCKAFFRRNAHK>ALPSIIAALRSSGDATTTDAYLQALRRGLR>-----DLTCTSKSGEHVVSPSTRRECPACRLKRCFLIGM

TsHR96b CRVCGDRAVNHNFGQLTCESCKAFFRRNAHK>ALPSIIAALRSSGDATTTDAYLQALRRGLR>-----DLTCTSKSGEHVVSPSTRRECPACRLKRCFLIGM

TsoHR96b CRVCGDRAVNHNFGQLTCESCKAFFRRNAHK>ALPSIIAALRSSGDATTTDAYLQALRRGLR>-----DLTCTSKSGEHVVSPSTRRECPACRLKRCFLIGM

**Trematoda**

CsHR96b CKVCGDRAVNHNFGQLTCESCKAFFRRNAHK>LLPSLLDRTFECRSDVDMTTVQYSLSLSRSKYLV->ELTCTSKTGEHEITPSTRRECPACRLKKCFLVGM

EcaHR96b CKVCNDRAVNHNFGQLTCESCKAFFRRNAHK>------------------------------------ELTCTLKSGEHEITPTTRRECPACRLKKCFRVGM

FhHR96b CKVCNDRAVNHNFGQLTCESCKAFFRRNAHK>------------------------------------DLTCTLKAGEHEITPTTRRECPACRLKKCFRVGM

OfNR96b CKVCGDRAVNHNFGQLTCESCKAFFRRNAHK>LLPSLLDRTFECRSDIDMTTVQYSLSLSRSKYLV->ELTCTSKTGEHEITPSTRRECPACRLKKCFLVGM

OvHR96b CKVCGDRAVNHNFGQLTCESCKAFFRRNAHK>LLPSLLDRTFECRSDVDMTTVQYSLSLSRSKYLV->ELTCTSKTGEHEITPSTRRECPACRLKKCFLVGM

SsHR96b CKVCGDRAVNHNFGQLTCESCKAFFRRNAHK>------------------------------------ELTCTSKTGEHVVSPTTRRECPACRLKRCFLIGM

SmHR96b CKVCGDRAVNHNFGQLTCESCKAFFRRNAHK>GLSTLMVTRMLRTNDTSSLIYESNRTAHIKSRGLV>ELTCTAKSGEHVITPTTRRECPSCRLKQCFRVGM

SmtHR96b CKVCGDRAVNHNFGQLTCESCKAFFRRNAHK>GLSTLMVTRMLRTNDTSSLIYEPNRTAHIKSRGLV>ELTCTAKSGEHVITPTTRRECPSCRLKQCFRVGM

SbHR96b CKVCGDRAVNHNFGQLTCESCKAFFRRNAHK>GLSTLMVTRMLRTNDTSSLIYEPNRTAHIKSRGLV>ELTCTAKSGEHVITPTTRRECPSCRLKQCFRVGM

ScHR96b CKVCGDRAVNHNFGQLTCESCKAFFRRNAHK>GLSTLMVTRMLRTNDTSSLIYEPNRTAHIKSRGLV>ELTCTAKSGEHVITPTTRRECPSCRLKQCFRVGM

ShHR9b CKVCGDRAVNHNFGQLTCESCKAFFRRNAHK>GLSTLMVTRMLRTNDTSSLIYEPNRTAHIKSRGLV>ELTCTAKSGEHVITPTTRRECPSCRLKQCFRVGM

SjHR96b >GLSTLLETRMLRTNDTSSLLYESNRTSHIRSRGLV>ELTCTAKSGEHVITPTTRRECPSCRLKQCFRVGM

SmaHR96b CKVCGDRAVNHNFGQLTCESCKAFFRRNAHK>GLSTLMVTRMLRTNDTSSLIYEPNRTAHIKSRGLV>ELTCTAKSGEHVITPTTRRECPSCRLKQCFRVGM

SrHR96b CKVCGDRAVNHNFGQLTCESCKAFFRRNAHK>GLSTLMVTRMLRTNDTSSLIYESNRTAHIKSRGLV>ELTCTAKSGEHVITPTTRRECPSCRLKQCFRVGM

TrHR96b CKVCGDKAVNHNFXXXXXXX>---------------------------------------------->ELTCTAKSGEHIITPTTRRECPSCRLKQCFRVGM

**Rhabditophora**

SmeHR96b CKVCFDKAVNQNFGVLSCESCKAFFRRNAIRSS>--------------------------------->PLKCSNGTDLCSVTSSTRKQCPSCRLKKCLQVGM

MlHR96-12 CRVCGDLAATFNFGQICCESCKAFFRRNAEK---------------(no intron)-----------HLDCSHGDGNCLITVTTRRTCRACRFEKCLAVGM

MlHR96-13 CLVCGCKAENYNFGVISCESCKAFFRRNAHK---------------(no intron)-----QTLGEAGFACSFRRGGCEVSLATRKKCPGCRLAKCFAVGM

MlHR96-14 CLVCGAKAENYNFGVISCESCKAFFRRNAHK---------------(no intron)-----KTLGDAGFSCSFQHARCEVTLATRKKCPGCRLAKCFRVGM

3
